# Exploring the biomarkers and therapeutic mechanism of kidney-yang deficiency syndrome treated by You-gui pill using systems pharmacology and serum metabonomics†

**DOI:** 10.1039/c7ra12451a

**Published:** 2018-01-03

**Authors:** Ruiqun Chen, Jia Wang, Chengbin Liao, Lei Zhang, Qian Guo, Xiufeng Wang

**Affiliations:** School of Basic Courses, Guangdong Pharmaceutical University Guangzhou 510006 P. R. China wxfsnow8012@126.com +86-20-39352186 +86-20-39352195

## Abstract

In this study, systems pharmacology was used to predict the molecular targets of You-gui pill (YGP) and explore the therapeutic mechanism of Kidney-Yang Deficiency Syndrome (KYDS) treated with YGP. On the basis of this, serum samples from control group, KYDS model group and YGP group rats were studied using ^1^H NMR to verify the results of systems pharmacology from the level of metabonomics. Simultaneously, ^1^H NMR spectra of serum samples were obtained and statistically assessed using pattern recognition analysis. Biochemical analyses of serums were performed *via* radioimmunoassays. Furthermore, histopathological studies were conducted on the pituitary, adrenal, and thyroid glands, and testicles of the control, KYDS and YGP rats. Using systems pharmacology to analyze the active components of YGP, 61 active compounds were finally found. These compounds were likely to have an effect on 3177 target proteins and involve 234 pathways. Using metabonomics to analyze serum from KYDS rats treated with YGP, 22 endogenous biomarkers were found. These biomarkers were mainly involved in 10 metabolic pathways. Combining systems pharmacology and metabonomics, we found that the regulation of KYDS was primarily associated with 19 active compounds of 5 Chinese herbal medicines in YGP. These active compounds mainly had an effect on 8 target proteins, including phosphoenolpyruvate carboxykinase, betaine-homocysteine *s*-methyltransferase 1, alcohol dehydrogenase 1C, *etc.* These target proteins were primarily involved in 6 overlapping pathways, namely aminoacyl-tRNA biosynthesis, glycolysis/gluconeogenesis, glycine, serine and threonine metabolism, valine, leucine and isoleucine biosynthesis, arginine and proline metabolism, and pyruvate metabolism. In addition, there were 4 non-overlapping pathways, respectively alanine, aspartate and glutamate metabolism, d-glutamine and d-glutamate metabolism, ubiquinone and other terpenoid-quinone biosynthesis, and galactose metabolism. In summary, the therapeutic effects of YGP on KYDS were mainly associated with neuroendocrine regulation, energy metabolism, amino acid metabolism, inflammatory responses, apoptosis, oxidative stress and intestinal flora metabolism. What's more, we also found that YGP possessed the potential to protect liver and kidney function. Our study demonstrated that systems pharmacology and metabonomics methods were novel strategies for the exploration of the mechanisms of KYDS and TCM formulas.

## Abstract 2

In this study, systems pharmacology was used to predict the molecular targets of You-gui pills (YGP) and explore the therapeutic mechanism of Kidney-Yang Deficiency Syndrome (KYDS) treated with YGP.

## Graphical abstract

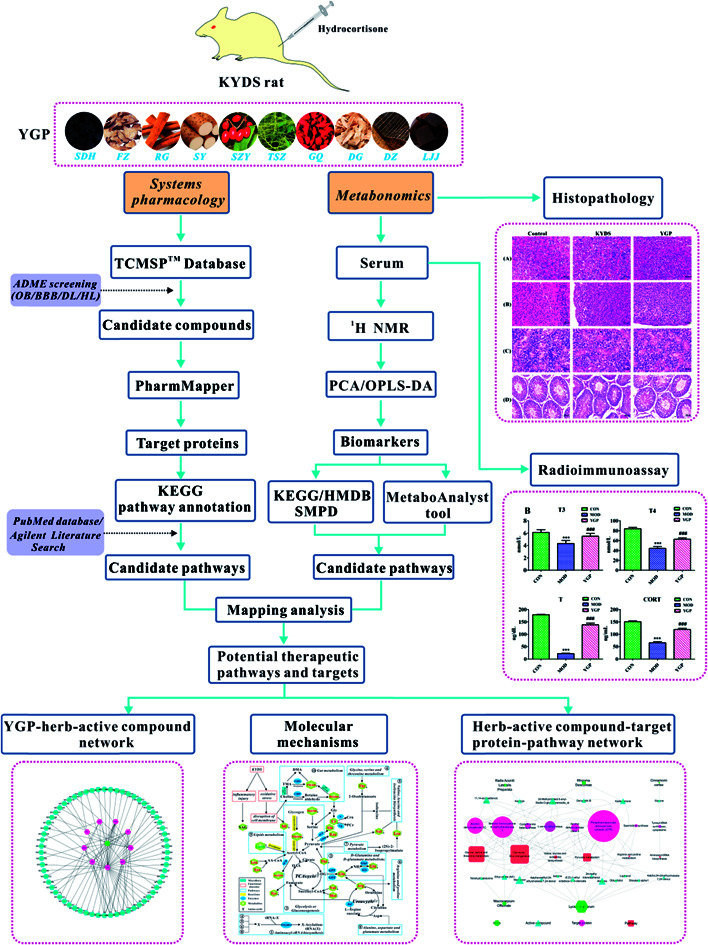

## Introduction

You-gui pill (YGP) is a Traditional Chinese Medicine (TCM) formula consisting of ten herbs: *Radix Rehmanniae Preparata* (Shu-Di-Huang), *Radix Aconiti Lateralis Preparata* (Fu-Zi), *Cinnamomi cortex* (Rou-Gui), *Rhizoma Dioscoreae* (Shan-Yao), *Macrocarpium Officinale* (Shan-Zhu-Yu), *Cuscuta reflexa* (Tu-Si-Zi), *Lycium Barbarum* (Gou-Qi), *Antler glue* (Lu-Jiao-jiao), *Cortex Eucommiae* (Du-Zhong), and *Radix Angelicae Sinensis* (Dang-Gui). They have the effect of improving glucocorticoid-induced symptoms and anti-apoptosis.^1^ Thus, they are used as a classic prescription to treat Kidney-Yang Deficiency Syndrome (KYDS).

‘Kidney-Yang Deficiency Syndrome’, one of the common syndrome patterns in TCM, was recorded first in an early systematic and theoretical monograph existing in China, “Neijing”,^2^ and the characteristics were warm dysfunction and metabolic disorder of body fluid, cold limbs, tinnitus, aversion to cold, *etc*.^3,4^ Studies have shown that the main mechanism of occurrence for KYDS is the hypothalamus-pituitary-target gland (adrenal, thyroid and gonad) axis of varying degrees of functional disorders.^5,6^ However, what metabolites in the body or which metabolic pathways specifically cause the occurrence of KYDS are not yet clear. Besides, which herbs and active ingredients in YGP work on which metabolites, pathways and target proteins also remains poorly understood.

Most complex diseases are not caused by changes in a single gene, but by the disruption of biological networks caused by the dysfunction of many genes or their products. Similarly, many of the different active ingredients of TCM formulas work through multi-target synergies to form a group of complex constituents, and then play a curative role. Treatment using this system may involve very complex dynamic intermolecular interaction networks.^7,8^ Systemic biology^9^ and network pharmacology have emerged in recent years and are used to reveal the molecular mechanisms of the interactions and herbal compatibilities of TCM formulas.^10,11^ Especially, systems pharmacology, as a new emerging field featuring multi-discipline and multi-technique approaches, has made a significant contribution to TCM pharmacological research.^12,13^ This approach combines oral bioavailability prediction, multiple drug target prediction and network analysis to understand the active compounds and therapeutic targets of TCM formulas from the perspective of the whole system.^14–16^

Metabonomics is a powerful tool that allows the assessment of global low-molecular-weight metabolites in a biological system and shows great potential in biomarker discovery.^17^ It can provide important evidence to elucidate the mechanisms of herbal medicines. This is consistent with the holistic approach of traditional Chinese medicine to the treatment of diseases. Compared with other technologies, NMR is widely used in metabonomics research because of the advantages of using less sample, being rapid, quantitative, and noninvasive, showing reproducible results, *etc.*^18,19^

**Fig. 1:**
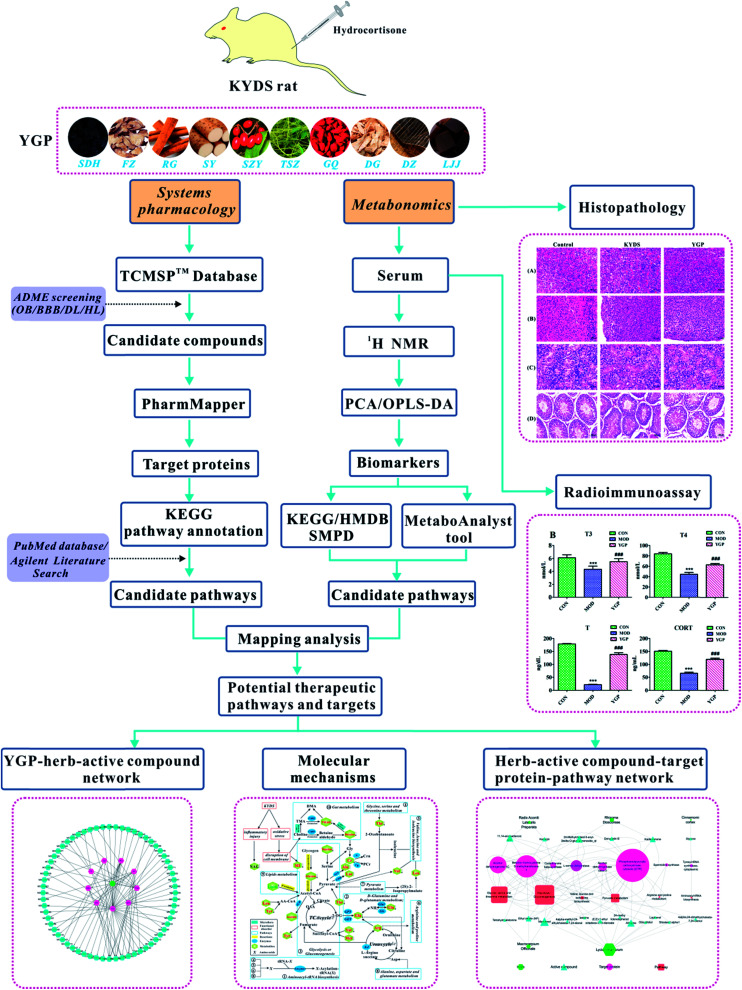
Overall scheme for the processes in this study.

All in all, although metabolic research into the use of different TCM formulas for the treatment of KYDS has a small amount of reports, the study of YGP for the treatment of KYDS still proceeds very slowly. There is an urgent need for a new approach to study the underlying mechanism and biological basis for YGP being used to treat KYDS. In light of this, we use an approach combining systems pharmacology and serum metabonomics to characterize the metabolic pathways of KYDS and evaluate the intervention effects from YGP against KYDS.^20^ The overall scheme for the processes in this study is shown in Fig. 1.

## Materials and methods

### Reagents and instruments

All of the Chinese herbal medicines in this study were purchased from Caizilin Drug Store (Guangzhou, China), and authenticated by Associate Prof. Hongyan Ma (College of Traditional Chinese Medicine of Guangdong Pharmaceutical University). Hydrocortisone injections were got from Tianjin Jinyao Pharmaceutics Co., Ltd (State medicine license no. H14020980, Tianjin, China). A 500 MHz Bruker AVANCE III NMR instrument (Bruker, Swiss) and D_2_O were provided by Guangdong Pharmaceutical University Center Laboratory. 0.9% saline injections were purchased from Guizhou Tiandi Pharmaceutics Co., Ltd (State medicine license No.: H52020069, Guizhou, China). Anhydrous ethanol was provided by Tianjin Baishi Chemical Co., Ltd (Tianjin, China). Sodium dihydrogen phosphate (NaH_2_PO_4_) and disodium hydrogen phosphate (Na_2_HPO_4_) were got from Dragon Technology Co., Ltd (Qingdao, China). The hormone kits were obtained from the Beijing Beitu Dongya Biotechnology Research Institute (Beijing, China). All other reagents were of analytical grade.

### Preparation of YGP

According to the original composition and preparation method of YGP recorded in “Jing Yue Quan Shu”, YGP solution was extracted from SDH (24 g), FZ (6 g), RG (6 g), SY (12 g), SZY (9 g), TSZ (12 g), GQ (12 g), LJJ (12 g), DZ (12 g), and DG (9 g). The specific method of decoction was as follows.^21,28^ Nine of the above kinds of traditional Chinese medicine, all except LJJ, were soaked for 0.5 h in distilled water and boiled for 20 min, followed by simmering for 20 min, and then filtration. Then the residual herbs were soaked in cold distilled water for 1 h and subjected to the same decoction and filtration procedure. The filtrate was mixed, LJJ was added to it, and it was concentrated to 111 mL in a rotary evaporator. Finally, YGP extract solution containing 1.0 g mL^−1^ of crude herb was obtained and the administration dosage for rats was 1.0 mL/100 g.

### Text mining and systems pharmacology analysis

TCMSP^22^ is a very useful systems pharmacology database and analysis platform for users to comprehensively study TCM: it includes the identification of active components, the screening of drug targets, and the generation of compounds-targets-diseases networks, as well as detailed drug pharmacokinetic information involving information about drug-likeness (DL), oral bioavailability (OB), the blood–brain barrier (BBB), drug half-life (HL), *etc.* The ten Chinese herbs included in YGP were uploaded to the TCMSP database (http://lsp.nwu.edu.cn/tcmsp.php) to obtain the compounds contained in each Chinese medicine. OB was undoubtedly one of the most important pharmacokinetic parameters, which revealed the convergence of the ADME process and indicated the capability of a given compound to be delivered into systemic circulation.^23^ Molecules with OB ≧ 30% were retained as candidate bioactive compounds to carry out analysis. The BBB's function is to limit the passage of proteins, and allows the evaluation of potentially diagnostic and therapeutic agents passing into the brain parenchyma. This was critical for us to understand and evaluate the capacity of compounds to enter into the central nervous system. Compounds with a BBB value of >0.3 were considered as strongly penetrating.^24^ DL was used to optimize pharmacokinetic and pharmaceutical properties, such as solubility and chemical stability. A DL ≧ 0.18 was used as a selection criterion for compounds in traditional Chinese herbs.^10^ Drug half-life (*t*_1/2_) was arguably the most important property, as it dictates the timescale over which the compound may elicit therapeutic effects.^25^ Compounds with a HL ≧ 4 were in the slow-elimination group and had longer interaction times. Therefore, compounds with OB ≧ 30%, a BBB value > 0.3, DL ≧ 0.18 and a HL ≧ 4 were selected as active compounds for further analysis.

### Target prediction

The above active compounds were uploaded to the PharmMapper server database ^26^ to predict and analyze the target proteins. This database contains more than 7000 receptor-based pharmacophore models extracted from the DrugBank, PDTD, BindingDB and Target-Bank databases, and predicts potential targets for various compounds *via* reverse pharmacophore matching. In targeting the top 300, a *Z*-score ≧ 0.8 ^27^ was used to screen potential target proteins. PharmMapper is available at http://lilab.ecust.edu.cn/pharmmapper/index.php.

### Pathway annotations

The above target proteins were uploaded to the KEGG database for pathway annotation. In addition, “Kidney-Yang Deficiency Syndrome” and “KYDS” were regarded as search terms to obtain the relevant pathways from the PubMed database and the Agilent Literature Search text mining tool.

### Animal care and experiments

Male Sprague-Dawley (SD) rats (weighing 240 ± 20 g) were provided by Guangdong Provincial Experimental Animals Center (License no. SCXK (Yue) 2008-0002) and kept in SPF-grade experimental animal houses. All rats had free access to food and water under standard conditions of temperature (24 ± 2 °C) and relative humidity (55 ± 5%), and a 12 h light/dark cycle. Animals were acclimatized to the new environment for one week prior to experimentation. After acclimatization, all rats were randomly divided into three groups of 8 rats each as follows: a control group, a KYDS model group, and a YGP-treated group. Rats in the control group were given an intramuscular injection of 0.3 mL of 0.9% saline into a hind limb, once a day, for 22 days in succession. For the KYDS model group, a 2.5 mg/100 g hydrocortisone suspension was injected intramuscularly into a hind limb, once a day, for 22 days in succession. On the 8^th^ day of replication of the KYDS model, the YGP-treated rats were given YGP solution. The administration dosage was 1.0 mL/100 g, once a day, for 15 days in succession.

The rats were sacrificed in batches, with parallel processing on the 23^rd^ day. The levels of triiodothyronine (T_3_), tetraiodothyronine (T_4_), testosterone (T) and corticosterone (CORT) were measured *via* radioimmunoassays. The animal care and experimental procedures were strictly performed in accordance with NIH guidelines (NIH Publication no. 85-23 Rev. 1985) and were approved by the institutional ethical committee (IEC) of Guangdong Pharmaceutical University. Meanwhile, the experimental methods also were conducted according to the principles expressed in the Declaration of Helsinki.

### Collection and preparation of samples

On the 23^rd^ day, plasma was collected from the orbital venous plexus and immediately transferred into tubes, and centrifuged (14 500 rpm) at 4 °C for 10 min. Supernatant samples were collected and stored at −80 °C, flash frozen in liquid nitrogen, until analyses. 300 μL of supernatant was mixed with 180 μL of phosphate buffer solution (0.2 mol L^−1^ Na_2_HPO_4_/NaH_2_PO_4_, pH = 7.4), to minimize chemical shift variation, and 120 μL of D_2_O in a clean EP tube.^28^ After being thoroughly mixed, the mixture was centrifuged (14 500 rpm, 5 min, 4 °C) and the supernatant was taken. 600 μL of supernatant was pipetted into a 5 mm NMR tube. After sacrificing, pituitary, adrenal, thyroid and testicular tissues were quickly taken out and fixed in 10% formalin overnight.

### Biochemical analyses and histopathological studies

The levels of T_3_, T_4_, T and CORT in the serum were measured *via* radioimmunoassays, which were operated strictly according to the manufacturers' instructions on commercial kits. These four hormone indicators were obtained based on a sensitivity index from our previous studies.^29–31^ The tissue samples were embedded in paraffin and 5 μm sections were prepared using a microtome. Then, the pituitary, adrenal, thyroid, and testis sections were stained with hematoxylin and eosin (H&E), and observed under a microscope and photographed.

### NMR data acquisition

^1^H NMR spectra from serums were recorded using a Bruker Avance 500 MHz spectrometer at 298 K. A water-suppressed standard one-dimensional Carr-Purcell-Meiboom-Gill (CPMG) pulse sequence [recycle delay − 90° − (*τ* − 180° − *τ*) *n*-acquisition] was adopted to reduce peak overlap and eliminate interference from macromolecules. 128 transients were collected into 32k data points using a spectral width of 10 kHz with a relation delay of 3 s, and the total echo time (2*nτ*) was 100 ms.^28^

### Data processing and multivariate data analysis

All the ^1^H NMR spectra were phase- and baseline-corrected manually, and then bucketed and automatically integrated with an automation routine in AMIX (Bruker, Germany).^32^ Spectra were calibrated to lactate (d, *δ* = 1.33) and segmented into integrated regions of 0.004 ppm. The region of *δ* 4.7–5.2 was discarded to eliminate variations in water suppression effects. To avoid any significant concentration differences, the integrals of each bucket from the region *δ* 0.5–8.5 were normalized to the total sum of the spectral integrals. The results of the data were saved in Microsoft Excel format and submitted to PCA and OPLS-DA analysis using SIMCA 13.0 software (Umetrics, Sweden). Principal component analysis (PCA) was an unsupervised method used to verify inherent clustering and reduce variables, while orthogonal partial least squares discriminant analysis (OPLS-DA) was a supervised method performed to maximize the distinctions between groups.

### Biomarker identification

For the identification of potential biomarkers, some available biochemical databases, such as HMDB, KEGG, and Chemspider were used to compare the accurate ^1^H NMR information. Moreover, potential biomarkers among this information were further identified by comparing with reference standards.^33,34^ The coefficient diagrams in this study were obtained by introducing the chemical shift values, the loading values of the scores plots, and the coefficient values (|*r*|) of the loading plots into MATLAB (The Mathworks Inc, USA).^35^ |*r*| ≥ 0.707 was used as a criterion for candidate metabolic biomarkers, according to the correlation coefficient threshold (*n* = 8). The colored bars in the figures indicate the degree of difference between both groups. Red represents a significant difference, while blue represents that a difference is not significant.

### Construction of metabolic pathways

The metabolic pathways of endogenous metabolites could be constructed using the MetaboAnalyst tool based on database sources, including KEGG, HMDB and SMPD, to analyze and visualize the metabolic pathways, and facilitate further biological interpretation.^33^ Meanwhile, the metabolites were uploaded and analyzed *via* a metabolite set enrichment analysis overview and over-representation analysis (ORA) in MetaboAnalyst.^36^ Specific pathway analysis algorithms used default values.

### Combining systems pharmacology and metabonomics analyses

The *P*-value < 0.05 pathways, obtained from metabonomics analyses, were mapped to all the pathways of YGP from the systems pharmacology analysis to find the overlapping pathways and non-overlapping pathways. According to the results of systems pharmacology analysis, the overlapping pathways were deduced in reverse to get the corresponding active compounds, target proteins and traditional Chinese medicines, so as to illuminate the therapeutic mechanism of YGP on KYDS.

### Statistical analysis

SPSS 20.0 for Windows was used for the statistical analysis of the biochemical and metabonomics data. Statistically significant differences in mean values were tested using Student's *t*-test, and a *P*-value < 0.05 was considered to indicate a statistical difference.

## Results

### Weight, biochemical parameter results and histological changes

**Fig. 2:**
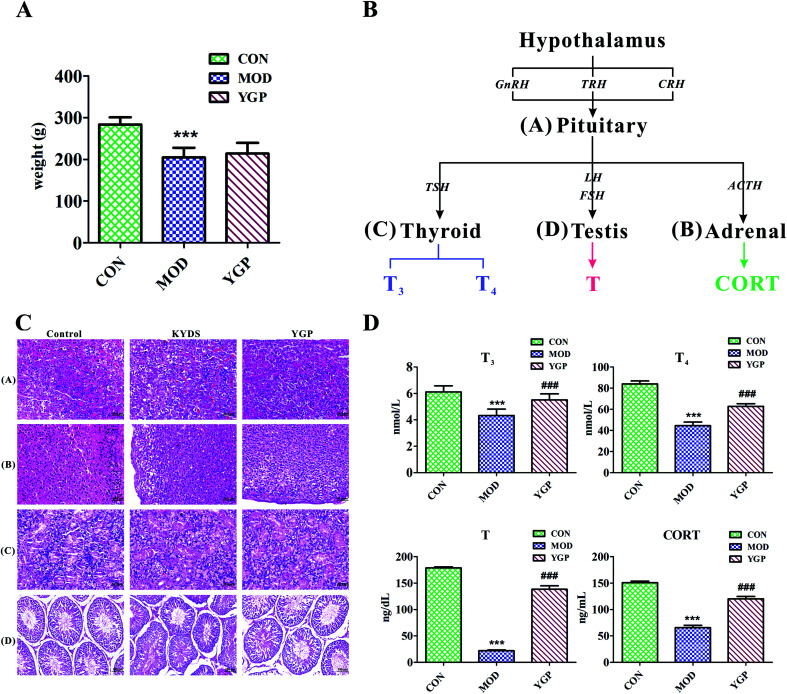
(A) Changes in the body weight index of rats (control group, model group, and YGP-treated group) on day 23; (B) the occurring mechanisms of KYDS-HPT axis and HPA axis disorders; and (C) H&E staining of pituitary, adrenal, thyroid and testicular tissue sections [(A–C): magnification 40×; and (D): magnification 20×]. (D) The biochemical characteristics used for the evaluation of the therapeutic effects of YGP on KYDS. Bar plots represent the mean relative hormone intensities and standard deviations, error bars represent the mean ± SD (Student's *t*-test: * significant difference from control group at *p* < 0.05; ** significant difference from control group at *p* < 0.01; *** significant difference from control group at *p* < 0.001; ^#^ significant difference from model group at *p* < 0.05; ^##^ significant difference from model group at *p* < 0.01; ^###^ significant difference from model group at *p* < 0.001. After treatment with YGP, various levels of T_3_, T_4_, T and CORT in the treatment group returned to the level of the control group) (one-way ANOVA with a Bonferroni correction).

Fig. 2A shows that, at the end of the 23^rd^ day, the weight of SD rats decreased significantly after injection with hydrocortisone, while the weight of KYDS rats increased after the administration of YGP, but the effect was not particularly significant. Moreover, the biochemistry parameters of the control group, KYDS model group and YGP group are summarized in Fig. 2D. Compared with the normal group, the serum levels of T_3_, T_4_, T and CORT in the model group were significantly decreased. T_3_ and T_4_ are groups of iodine-containing tyrosine, and are synthesized in thyroid gland cells using iodine and tyrosine. In this experiment, because of the inhibition of tyrosine metabolism, the production of tyrosine decreased, resulting in decreased levels of T_4_ and T_3_. This was consistent with the 25^th^ pathway in Table 3. The hormones of the neuroendocrine immune system were decreased in the model groups, which meant the neuroendocrine immune system was in a state of inhibition. In addition, KYDS rats also developed a series of behavioral and physiological characteristics, including fatigue, reduced activity, the tendency to cluster, an aversion to cold, chills, a drop in appetite, and a loss of hair. All these results indicated that the KYDS model was successfully established. However, after treatment with YGP, the levels of the four hormones rose significantly and were close to normal levels. The behavioral and physiological characteristics of KYDS rats also improved significantly, and were close to the control group. This was consistent with the results of our earlier studies and literature reports.^35,37^ These results showed that YGP could reverse the biochemical, physiological and whole organism symptoms of KYDS.

In order to further confirm that YGP did have certain therapeutic effects on KYDS, we performed H&E staining on the pituitary, adrenal, thyroid and testicular tissue sections of the normal group, KYDS model group and YGP group. The results of H&E staining showed that the pituitary, adrenal gland, thyroid and testicular tissues showed significant changes between the normal group, the KYDS group and the YGP group (Fig. 2C).

As illustrated in Fig. 2C, it can be seen that the pituitary structures and boundaries of the control group were clear, and eosinophilic cells and basophilic cells were significantly distributed. In the model group, pituitary eosinophils increased, basophils decreased, and some cells showed vacuolar degeneration. While in the YGP treatment group, the pituitary basophils were significantly increased, and the intervals between cells gradually became clear. The adrenal cell structures of the control tissues were clear, and zona glomerulosas and zona fasciculatas could be distinguished. There was no vacuolar phenomenon in zona glomerulosa cells. In the model group, the cell volume became smaller, the arrangement was sparse, the intracellular lipid increased and vacuolization was obvious. The adrenal gland cells in the YGP treatment group were arranged neatly, and the structures of zona glomerulosas and zona fasciculatas were clear, and vacuolization was reduced. The thyroid follicular boundaries of the control group were neat, and the colloid was visible in the follicular cavity. In the model group, the thyroid follicles were atrophic, the number decreased and the follicles became unclear. However, the number of thyroid cells increased in the YGP treatment group, the follicular structures were normal and the boundaries were neat. The seminiferous tubules in the testicular tissue of the control group were intact and arranged closely. Besides, the spermatogonial cells and supporting cells were close to the basement membrane. In the YGP treatment group, the structures of the seminiferous tubules in the testis tissue were more complete, and the spermatogonial cells and the supporting cells were close to the basement membrane. The spermatogenic cells at different levels also were clear and a few cells were deformed, but not seriously. Taken together, these findings indicated that injection with hydrocortisone resulted in changes in the cell structures of pituitary, adrenal, thyroid and testicular tissues. This again demonstrated that the hypothalamic-pituitary-thyroid/gonadal axis (HPT axis) and hypothalamic-pituitary-adrenal axis (HPA axis) were inhibited (Fig. 2B). The HPT axis and the HPA axis were significantly improved after YGP adjustment. Moreover, in the systems pharmacology part, we found that the active ingredients of YGP could act on the GnRH signaling pathway, and other TCM formulas also have been reported in the literature^35^ to treat KYDS through this signaling pathway. This further indicated that YGP indeed acted through the regulation of the HPT axis and HPA axis to treat KYDS, and had a significant effect.

### Active compound screening

**Fig. 3:**
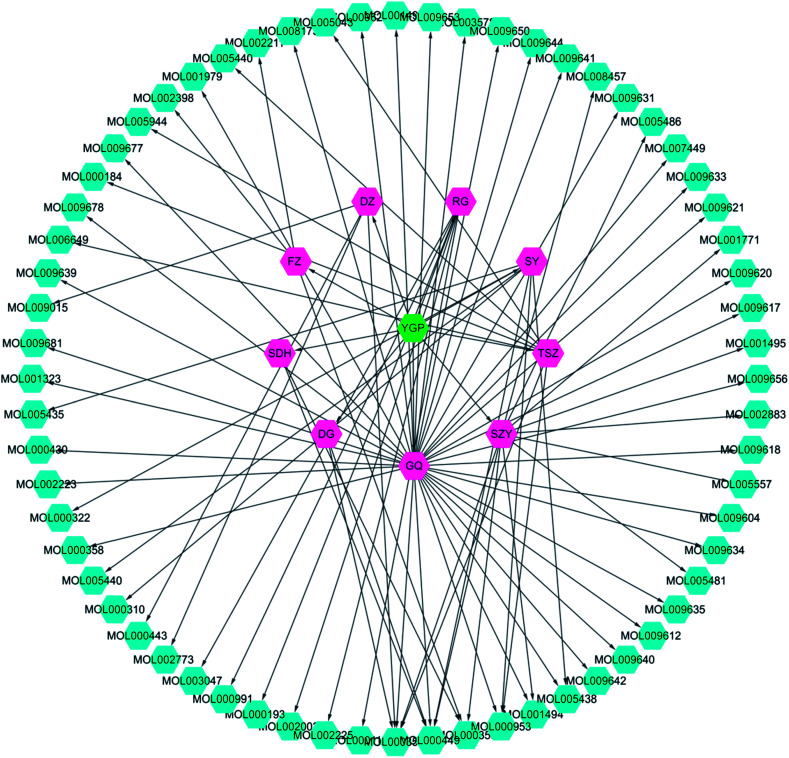
YGP herb-active compound network. This network is constructed using systems pharmacology to analyze nine kinds of Chinese herbs (purple) in YGP (green), with the corresponding 61 active compounds (cyan). YGP: You-gui pills; SDH: *Radix Rehmanniae Preparata*; FZ: *Radix Aconiti Lateralis Preparata*; RG: *Cinnamomi cortex*; SY: *Rhizoma Dioscoreae*; SZY: *Macrocarpium Officinale*; TSZ: *Cuscuta reflexa*; GQ: *Lycium Barbarum*; DZ: *Cortex Eucommiae*; and DG: *Radix Angelicae Sinensis*. Detailed information about the 61 active compounds is available in ESI Table S1.†

Screening results showed that 61 molecules were obtained from 9 herbs; 2 from SDH, 3 from FZ, 7 from SY, 11 from SZY, 7 from TSZ, 35 from GQ, 4 from DZ, 2 from DG and 6 from RG (Fig. 3 and ESI Table 1†). There were 8 active compounds (*e.g.*, sitosterol, stigmasterol, mandenol, *etc.*) widely existing in a variety of TCM components, so there was overlap. Meanwhile, since the TCMSP database did not contain information about *Antler glue* (LJJ), there were no active compounds associated with it to be screened. In addition, there were literature reports that taurine^38^ and betaine^39^ were also important pharmacological components in DG. Hence, even if the BBB and DL values were relatively small, both of these were regarded as active compound targets for further analysis. Similarly, even though 6 compounds (cinnamaldehyde, styrone, (l)-alpha-terpineol, (−)-caryophyllene oxide, (*Z*)-caryophyllene, and [(1*S*)-*endo*]-(−)-borneol) had relatively small DL values, they were retained as active compounds from RG.^40–42^

### Results of target prediction

Screening results showed that 3177 target proteins met the criteria and were likely to be affected by the active compounds (ESI Table S2†).

### Pathways corresponding to the target

The results showed that the 3177 target proteins were involved in 201 pathways. Moreover, the search results manifested 33 pathways associated with KYDS, obtained from the PubMed database and Agilent Literature Search text mining tool. Overall, a total of 234 pathways associated with KYDS were acquired (ESI Table S3†).

### ^1^H NMR spectra of serum samples

**Fig. 4:**
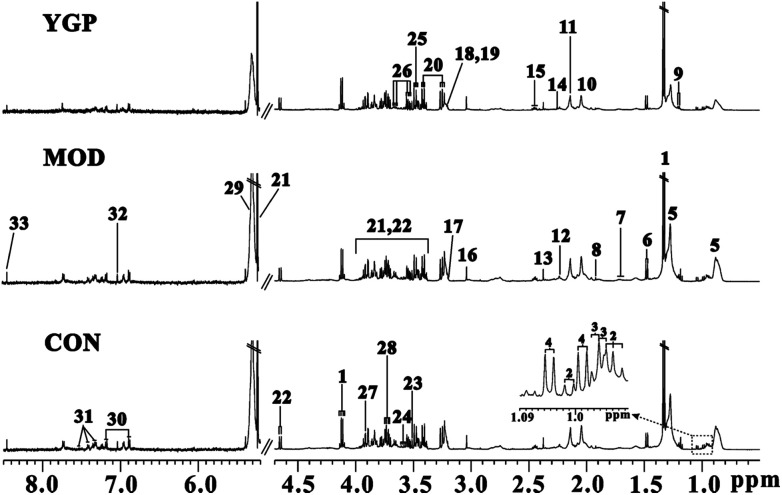
Representative serum (CON: control; MOD: model; and YGP: You-gui pills) ^1^H NMR spectra from the control, model and YGP groups at the end of 23 days. Key: (1) lactate; (2) isoleucine; (3) leucine; (4) valine; (5) lipid; (6) alanine; (7) arginine; (8) acetate; (9) 3-hydroxybutyrate; (10) *N*-acetyl glycoprotein; (11) methionine; (12) *O*-acetylglycoprotein; (13) pyruvate; (14) glutamate; (15) glutamine; (16) creatine; (17) choline; (18) PC; (19) GPC; (20) taurine; (21) α-glucose; (22) β-glucose; (23) glycine; (24) myo-inostiol; (25) threonine; (26) glycerol; (27) TMAO/betaine; (28) alanine; (29) unsaturated lipid; (30) tyrosine; (31) phenylalanine; (32) histidine; and (33) formate.

The peaks in the spectra were assigned using previously reported ^1^H NMR assignments for metabolites, which were obtained from the Biological Magnetic Resonance Data Bank database, HMDB,^43–45^ and the literature (Fig. 4).^18,46–48^

### Results of PCA and OPLS-DA

**Fig. 5:**
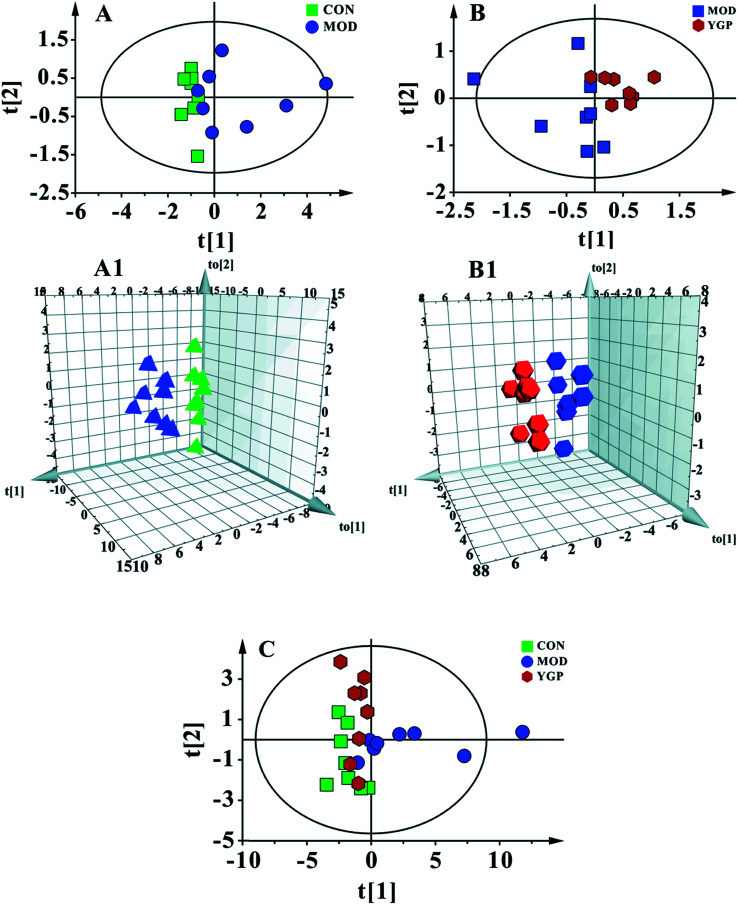
Multivariate analyses of serum ^1^H NMR spectra data at the end of the 23^rd^ day. (A) PCA score plot of the control (green) and KYDS model groups (blue) (R^2^X = 0.97, *Q*^2^ = 0.846) a; and (A1) a 3D plot of OPLS-DA, based on serum metabolites discriminating the control and KYDS model groups (*R*^2^*X* = 0.772, *R*^2^*Y* = 0.987, *Q*^2^ = 0.65). (B) PCA score plot of the KYDS model group (blue) and YGP group (red) (*R*^2^*X* = 0.975, *Q*^2^ = 0.584); and (B1) a 3D plot of OPLS-DA based on serum metabolites discriminating the KYDS model group and YGP group (*R*^2^*X* = 0.787, *R*^2^*Y* = 0.995, *Q*^2^ = 0.809). (C) PCA score plot of serum results from the three groups (*R*^2^*X* = 0.793, *Q*^2^ = 0.606).

Fig. 5C shows a PCA score plot of serum from the control group, KYDS model group and YGP group at the end of the 23^rd^ day. As seen in the figure, the control group, KYDS model group and YGP group could be significantly separated, indicating that there were significant differences between the three groups. Fig. 5A and A1 show a PCA score plot and OPLS-DA 3D plot between the control group and KYDS model group. They are significantly different, indicating a significant difference between the two groups. Additionally, a PCA score plot and 3D plot of OPLS-DA between the KYDS model group and YGP group could also be significantly separated, manifesting that there were significant differences between the two groups (Fig. 5B and B1). Therefore, we did further analysis of these three sets of data to find different biomarkers among the groups.

### Analysis of metabolic patterns and identification of potential biomarkers

**Fig. 6:**
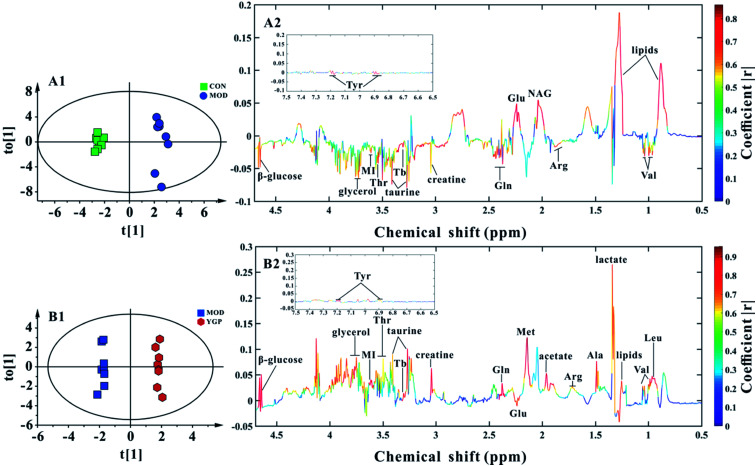
(A1, A2) Score plot and coefficient-coded loading plot for OPLS-DA between the control and KYDS model groups at the end of the 23^rd^ day (*R*^2^*X* = 0.772, *R*^2^*Y* = 0.987, *Q*^2^ = 0.65). (B1, B2) Score plot and coefficient-coded loading plot for OPLS-DA between the KYDS model group and YGP group at the end of the 23^rd^ day (*R*^2^*X* = 0.787, *R*^2^*Y* = 0.995, *Q*^2^ = 0.809). Abbreviations: Tyr: tyrosine; Lipids: VLDL/LDL; Leu: leucine; Val: valine; Ala: alanine; Arg: arginine; NAG: *N*-acetyl glycoprotein; Met: methionine; Glu: glutamate; Gln: glutamine; Thr: threonine; MI: myo-inositol; and Tb: TMAO/betaine.

Finally, 22 biomarkers of significant change were screened (Table 1). As shown in Fig. 6 and Table 1, compared with the control group, the levels of valine, arginine, glutamate, glutamine, creatine, taurine, threonine, myo-inositol, glycerol, TMAO/betaine, tyrosine and β-glucose significantly decreased in the KYDS model group, while lipids and *N*-acetyl glycoprotein increased significantly. Compared to the KYDS model group results, the levels of leucine, valine, lactate, alanine, arginine, acetate, methionine, glutamate, glutamine, creatine, taurine, threonine, myo-inositol, glycerol, TMAO/betaine, tyrosine and β-glucose were significantly increased in the YGP group, whereas lipids were significantly decreased. This showed that the metabolites of KYDS rats had changed significantly, while YGP could reverse the levels of these metabolites to normal conditions.

**Table 1:** Statistical analysis results showing the main endogenous metabolite changes in serum^a^

Metabolite	*δ* ^1^H (ppm) and multiplicity	Variation at the end of the 23^rd^ day	
		KYDS model group *vs.* control group	YGP group *vs.* KYDS model group
HDL	0.84 (m)	—	—
Lipids	0.89 (m), 1.29 (m)	↑**	↓**
Leu	0.95 (d)	—	↑**
Val	0.97 (d),1.02 (d)	↓*	↑**
Lactate	1.33 (d), 4.11 (t)	—	↑**
Ala	1.48 (d)	—	↑**
Arg	1.68 (m)	↓*	↑**
Acetate	1.91 (s)	—	↑**
NAG	2.04 (s)	↑**	—
Met	2.13 (s)	—	↑**
Glu	2.14 (m)	↓**	↑**
Gln	2.41 (m)	↓*	↑**
Creatine	3.04 (s)	↓*	↑**
Taurine	3.25 (t), 3.41 (t)	↓*	↑*
Thr	3.56 (dd)	↓*	↑**
MI	3.63 (dd)	↓*	↑**
Glycerol	3.64 (dd), 3.87 (m)	↓*	↑**
Tb	3.27 (s)	↓**	↑**
Tyr	6.87 (m), 7.17 (m)	↓*	↑**
β-Glucose	4.64 (d)	↓**	↑**

a Lipids: VLDL/LDL; Leu: leucine; Val: valine; Ala: alanine; Arg: arginine; NAG: *N*-acetyl glycoprotein; Met: methionine; Glu: glutamate; Gln: glutamine; Thr: threonine; MI: myo-inositol; Tyr: tyrosine; Tb: TMAO/betaine; s: singlet; d: doublet; t: triplet; and m: multiplet. ‘↑’ and ‘↓’ represent compounds which are up- and down-regulated in the KYDS model group compared with the control group or in the YGP group compared with the KYDS model group. *P < 0.05, **P < 0.01 (one-way ANOVA with a Bonferroni correction).

### Pathway analysis

**Fig. 7:**
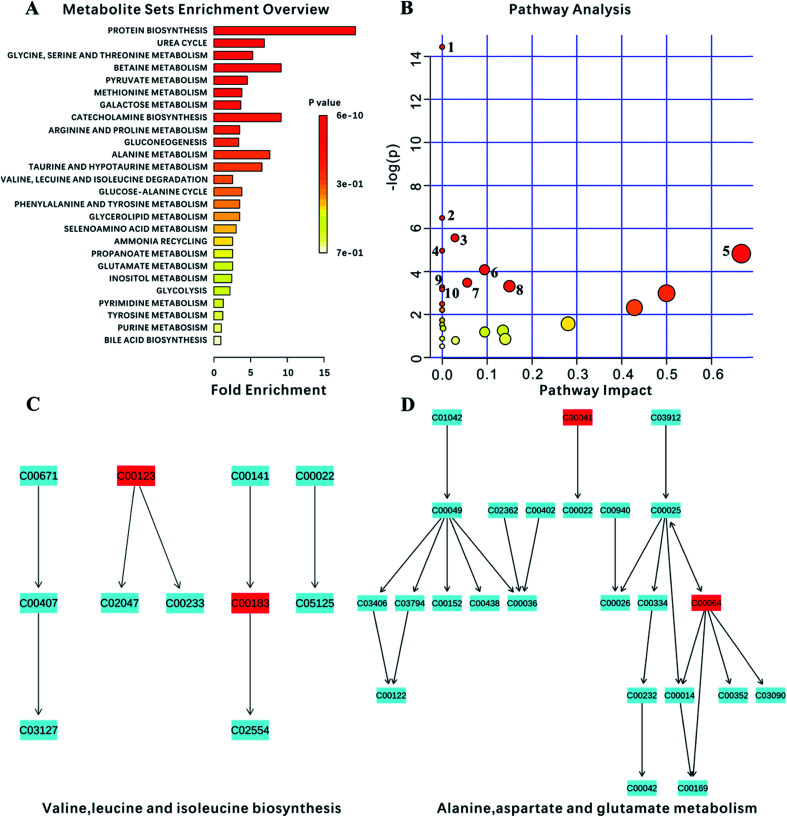
Analysis of metabolism pathways using MetPA for KYDS model rats compared with control rats, as visualized using a bubble plot. The bubble size is proportional to the impact of each pathway and the bubble color denotes the significance, from highest in red to lowest in white; a small *p* value and a big pathway impact factor indicate that the pathway is greatly influenced. The pathways are identified based on over-representation analysis (ORA), using the significantly altered metabolites listed in Table 2 except for lipid metabolites (LDL, VLDL, HDL) and *N*-acetyl glycoprotein.

Ultimately, 18 biomarkers were involved in 27 metabolic pathways. Furthermore, since the program could not recognize the lipids and the membrane metabolites (*e.g.*, LDL, VLDL, HDL and NAG), they were not included in this analysis. *P*-value < 0.05 metabolic pathways were selected as the main impact pathways for YGP adjusted KYDS. There were 10 pathways in total: (1) aminoacyl-tRNA biosynthesis; (2) d-glutamine and d-glutamate metabolism; (3) glycolysis or gluconeogenesis; (4) glycine, serine and threonine metabolism; (5) valine, leucine and isoleucine biosynthesis; (6) arginine and proline metabolism; (7) pyruvate metabolism; (8) alanine, aspartate and glutamate metabolism; (9) ubiquinone and other terpenoid-quinone biosynthesis; and (10) galactose metabolism (Fig. 7, Tables 2 and 3). Simultaneously, due to the impact values of pathway (C) and (D) being >0.1, both of these were regarded as more important pathways.

**Table 2:** Basic annotations of biomarkers

No.	Query	Hit	HMDB	KEGG	Enzymes	Pathways	Transporters
1	Leucine	l-Leucine	HMDB00687	C00123	7	50	1
2	Valine	l-Valine	HMDB00883	C00183	6	52	1
3	Lactate	l-Lactic acid	HMDB00190	C00186	5	16	—
4	Alanine	l-Alanine	HMDB00161	C00041	17	59	4
5	Arginine	l-Arginine	HMDB00517	C00062	25	30	—
6	Acetate	Acetic acid	HMDB00042	C00033	38	20	2
7	Methionine	l-Methionine	HMDB00696	C00073	23	50	1
8	Glutamate	d-Glutamic acid	HMDB03339	C00217	2	—	—
9	Glutamine	l-Glutamine	HMDB00641	C00064	28	53	4
10	Creatine	Creatine	HMDB00064	C00300	9	20	1
11	Taurine	Taurine	HMDB00251	C00245	11	8	—
12	Threonine	l-Threonine	HMDB00167	C00188	8	33	1
13	Myo-inositol	Myo-inositol	HMDB00211	C00137	7	6	—
14	Glycerol	Glycerol	HMDB00131	C00116	41	6	1
15	Betaine	Betaine	HMDB00043	C00719	4	18	—
16	TMAO	Trimethylamine *N*-oxide	HMDB00925	C01104	5	—	—
17	Tyrosine	l-Tyrosine	HMDB00158	C00082	15	17	2
18	Beta-glucose	Beta-d-glucose	HMDB00516	C00221	15	12	—

**Table 3:** Results of pathway analysis using MetPA^a^

No.	Pathway name	Hits	Total	Raw *p*	−log(*p*)	Impact	Details
1	Aminoacyl-tRNA biosynthesis	8	67	5.3584 × 10^−7^	14.439	0.0	KEGG
2	d-Glutamine and d-glutamate metabolism	2	5	0.0015226	6.4874	0.0	KEGG
3	Glycolysis/gluconeogenesis	3	26	0.0038445	5.5611	0.02862	KEGG
4	Glycine, serine and threonine metabolism	3	32	0.0069872	4.9637	0.0	KEGG
5	Valine, leucine and isoleucine biosynthesis	2	11	0.0079994	4.8284	0.66666	KEGG
6	Arginine and proline metabolism	3	44	0.016934	4.0784	0.09426	KEGG
7	Pyruvate metabolism	2	22	0.030903	3.4769	0.05583	KEGG
8	Alanine, aspartate and glutamate metabolism	2	24	0.036368	3.3141	0.14979	KEGG
9	Ubiquinone and other terpenoid-quinone biosynthesis	1	3	0.038051	3.2688	0.0	KEGG
10	Galactose metabolism	2	26	0.042181	3.2688	0.0	KEGG
11	Phenylalanine, tyrosine and tryptophan biosynthesis	1	4	0.050428	2.9872	0.5	KEGG
12	Valine, leucine and isoleucine degradation	2	38	0.083347	2.4847	0.0	KEGG
13	Taurine and hypotaurine metabolism	1	8	0.098447	2.3182	0.42857	KEGG
14	Ascorbate and aldarate metabolism	1	9	0.11009	2.2065	0.0	KEGG
15	Phenylalanine metabolism	1	9	0.11009	2.2065	0.0	KEGG
16	Nitrogen metabolism	1	9	0.11009	2.2065	0.0	KEGG
17	Selenoamino acid metabolism	1	15	0.17701	1.7316	0.0	KEGG
18	Pantothenate and CoA biosynthesis	1	15	0.17701	1.7316	0.0	KEGG
19	Glycerolipid metabolism	1	18	0.20866	1.5671	0.28098	KEGG
20	Pentose phosphate pathway	1	19	0.21895	1.5189	0.0	KEGG
21	Starch and sucrose metabolism	1	23	0.25887	1.3514	0.0021	KEGG
22	Inositol phosphate metabolism	1	26	0.28753	1.2464	0.09464	KEGG
23	Cysteine and methionine metabolism	1	28	0.30606	1.184	0.09464	KEGG
24	Pyrimidine metabolism	1	41	0.41583	0.87749	0.0	KEGG
25	Tyrosine metabolism	1	42	0.41583	0.85908	0.14045	KEGG
26	Primary bile acid biosynthesis	1	46	0.4535	0.79076	0.02976	KEGG
27	Purine metabolism	1	68	0.59365	0.52147	0.0	KEGG

a The “Total” is the number of compounds in the pathway; the “Hits” represents the actual matched number from the user uploaded data; the “Raw *p*” is the original *p* value calculated from enrichment analysis.

### Analysis of the interactions between biomarkers and pathways

**Fig. 8:**
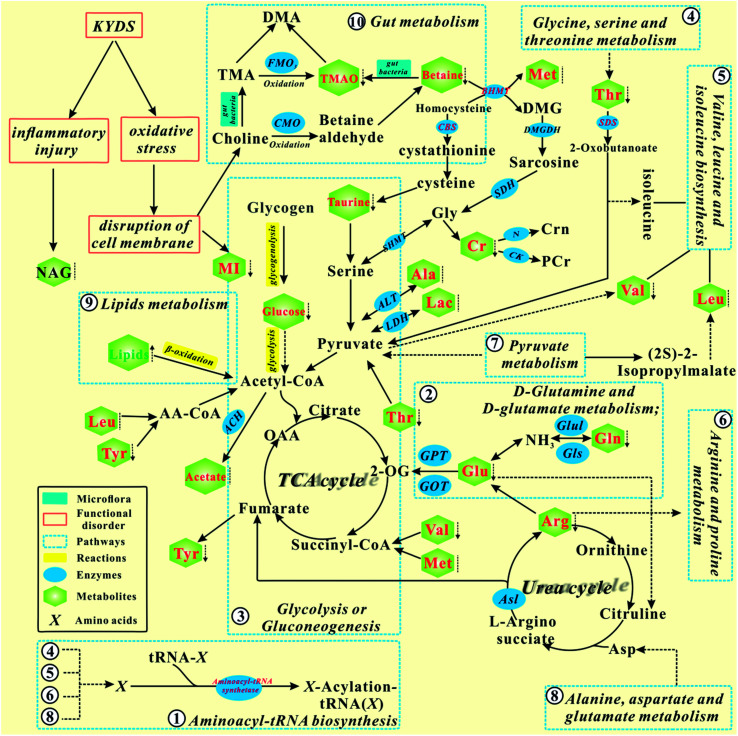
Schematic diagram of the metabolic pathway alterations among the biomarkers associated with KYDS and YGP treatment, according to the KEGG database. ↑ represents a significant elevation in metabolites in KYDS model rats, ↓ indicates a significant reduction, while ⁞ indicates no significant change. A red color indicates increased metabolites after treatment with YGP, a green color shows decreased metabolites, whereas a black color represents no significant change. A solid arrow indicates a single process, while a dotted arrow represents multiple processes.

According to the KEGG database and known biochemical knowledge, the interaction networks between metabolic markers and metabolic pathways were further analyzed. Fig. 8 represents a schematic diagram of the metabolic pathway alterations among the biomarkers associated with KYDS and YGP treatment, according to the KEGG database. It can be seen from the figure that after injection with hydrocortisone, there was an inflammatory reaction and oxidative stress in KYDS rats, leading to destruction of the cell membrane and a variety of metabolic pathway disorders; this eventually led to significant changes in the levels of serum metabolites. To the contrary, YGP could regulate the metabolic changes by reversing most of the imbalanced metabolites and pathways, including aminoacyl-tRNA biosynthesis, glycolysis or gluconeogenesis, gut metabolism, lipid metabolism, *etc.* These pathways were primarily involved in amino acid metabolism, energy metabolism, oxidative stress, gut metabolism and inflammatory responses.

### Combining systems pharmacology and metabonomics to analyze the network regulated by YGP

**Fig. 9:**
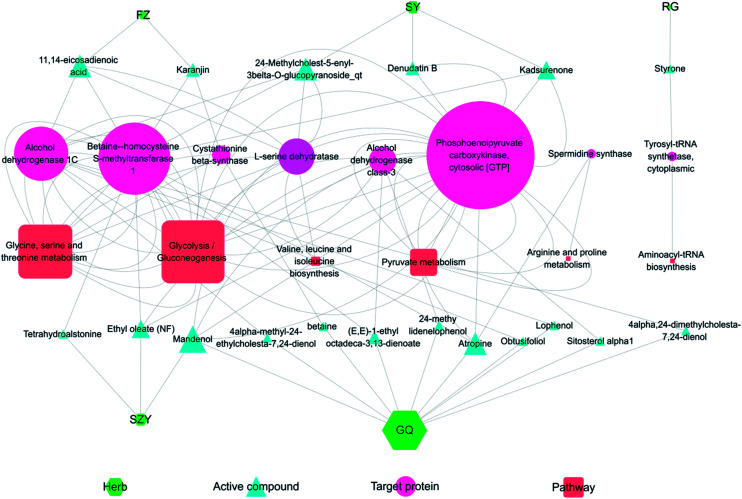
Herb-active compound-target protein pathway network. FZ: *Radix Aconiti Lateralis Preparata*; SY: *Rhizoma Dioscoreae*; RG: *Cinnamomi cortex*; SZY: *Macrocarpium Officinale*; and GQ: *Lycium Barbarum*. The size of the nodes is proportional to their degree value.

Fig. 9 shows the interaction network of the herbal-active compound-target protein pathway in YGP. As illustrated in the figure, there were mainly 5 types of TCM that regulated KYDS directly when using YGP; GQ, SY, SZY, FZ and RG. Taken together, 19 active compounds in 5 herbal medicines were obtained, of which 10 were in GQ, 3 were in SY, 3 were in SZY, 2 were in FZ and 1 was in RG. There were 8 target proteins associated with these active compounds: alcohol dehydrogenase class-3; alcohol dehydrogenase 1C; betaine-homocysteine *s*-methyltransferase 1; phosphoenolpyruvate carboxykinase; tyrosyl-tRNA synthetase; cytoplasmic l-serine dehydratase; spermidine synthase; and cystathionine beta-synthase. 6 pathways were mainly involved: glycolysis/gluconeogenesis; glycine, serine and threonine metabolism; pyruvate metabolism; valine, leucine and isoleucine biosynthesis; arginine and proline metabolism; and aminoacyl-tRNA biosynthesis. The color and shape of the nodes represents the four parts of the herb-active compound-target protein pathway network, and the size of nodes is proportional to their degree. This graph was drawn using the software Cytoscape 3.5.1 (ESI Table S4†).

## Discussion

Ten pathways of *P*-value < 0.05 were mapped to the 234 pathways obtained from systems pharmacology, and 6 pathways were found to be overlapping. These overlapping pathways are aminoacyl-tRNA biosynthesis; glycolysis/gluconeogenesis; glycine, serine and threonine metabolism; valine, leucine and isoleucine biosynthesis; arginine and proline metabolism; and pyruvate metabolism. In addition, there are four non-overlapping pathways, namely: alanine, aspartate and glutamate metabolism; d-glutamine and d-glutamate metabolism; ubiquinone and other terpenoid-quinone biosynthesis, and galactose metabolism.

### Glycolysis/gluconeogenesis

Glycolysis and gluconeogenesis are important sources of energy for the body. Non-glycemic substances (*e.g.*, lactate, glycerol, alanine, glutamate, arginine, threonine, glutamine and valine) supply energy to the body through the gluconeogenesis pathway. Our earlier studies have found that the functions of mitochondria were in disorder under the action of hydrocortisone, which leads to energy metabolism disorder.^35^ Therefore, the cells need to replenish energy for the body through the gluconeogenesis pathway. Phosphoenolpyruvate carboxykinase exists in mitochondria and cytoplasm, and is a rate-limiting enzyme for the gluconeogenesis pathway. It can catalyze oxaloacetic acid (OAA) to form phosphoenolpyruvate and CO_2_ in the gluconeogenesis pathway. The active compounds of four traditional Chinese medicines (*i.e.*, FZ, SY, SZY and GQ) in YGP mainly act on phosphoenolpyruvate carboxykinase to promote gluconeogenesis in the cytoplasm, thus supplementing the energy deficiency of glycolysis. Simultaneously, CORT, as an adrenal cortex hormone, can also increase gluconeogenesis in liver cells. The level of CORT in the serum of KYDS rats regulated using YGP is significantly increased, indicating that YGP can improve gluconeogenesis in rat hepatocytes.

Alcohol dehydrogenase (ADH) plays a rate-limiting role in the metabolic pathway and catalyzes the oxidation of ethanol to acetaldehyde.^49,50^ It is noteworthy that nine active compounds from YGP also affected glycolysis/gluconeogenesis by acting on alcohol dehydrogenase (alcohol dehydrogenase 1C and alcohol dehydrogenase class-3) in this study. We speculate that, owing to TCA cycle disorder, acetates produce acetaldehyde under the action of intestinal flora and enzymes, and then produce ethanol under the action of alcohol dehydrogenase. However, the active compounds in the YGP maybe improve the activity of intestinal flora and act on alcohol dehydrogenase to catalyze the reverse reaction. Ultimately, acetates are converted to acetyl CoA and further generate energy through the TCA cycle.

### Pyruvate metabolism

Pyruvate metabolism is regulated by multiple enzymes in mitochondria, and any abnormality in genes encoding proteins may lead to various diseases such as cancer, heart failure, and neurodegeneration.^51^ Under the action of lactate dehydrogenase (LDH) and alanine aminotransferase (ALT), lactate and valine are converted to pyruvate and pass through pyruvate metabolism into the TCA cycle. Five active compounds (*i.e.*, denudatin B, kadsurenone, ethyloleate (NF), mandenol and atropine) can act on phosphoenolpyruvate carboxykinase to regulate pyruvate metabolism.

### Glycine, serine and threonine metabolism

As seen in Fig. 9, most of the active compounds of the four major traditional Chinese medicines in YGP primarily act on betaine-homocysteine *s*-methyltransferase (BHMT). BHMT plays a crucial role in the cycle pathway and is regarded as an auto-antigen associated with immune reactivity.^52^ Betaine and homocysteine are converted to dimethylglycine (DMG) and methionine under the action of BHMT. DMG is a known feedback inhibitor of BHMT. The accumulation of DMG will inhibit BHMT activity, thereby causing hyperhomocysteinemia, further leading to chronic renal failure (CRF).^53^ On the one hand, the active compounds in YGP may improve the activity of BHMT, and promote DMG to generate sarcosine. Sarcosine then enters the TCA cycle through glycine, serine and threonine metabolism to provide energy for the body. On the other hand, active compounds also can increase the activity of cystathionine-beta-synthase (CBS). CBS can act as a homotetramer to catalyze the conversion of homocysteine to cystathionine. The above two aspects can prevent the occurrence of CRF and improve KYDS to some extent.

Taurine can be synthesized from methionine within the liver and is mainly removed through renal excretion to maintain relative stability in its body content. It can act as a trophic factor in the development of the central nervous system^54,55^ and improve endocrine status to enhance immune and anti-fatigue effects. In addition, there are reports that taurine also possess the ability to maintain the structural integrity of cell membranes,^56^ and possesses antioxidant activity,^57^ anticonvulsant activity,^58^ and osmoregulatory activity.^59^ The taurine content in KYDS rats is decreased, but it shows a significant increase in the YGP group, indicating that YGP has certain therapeutic effects on KYDS.

What's more, threonine can generate 2-oxobutanoate under the action of l-serine dehydratase (SDS) and then enter into the valine, leucine and isoleucine biosynthesis pathway for further metabolism. It can also be metabolized to glycine and acetyl CoA. Glycine plays an important role in metabolic regulation, anti-oxidative reactions, and neurological function.^60^ It can generate serine and enter pyruvate metabolism (Fig. 8). Glycine also can be converted to creatine. Creatine, an important energy storage material, can rapidly re-synthesize ATP to provide energy, which is conducive to the formation of muscle and rapid recovery from fatigue.^61^

### Valine, leucine and isoleucine biosynthesis

Leucine, isoleucine and valine are all branched chain amino acids (BCAAs). Leucine can work with isoleucine and valine to repair muscles, control blood sugar, and provide energy to body tissues. A lack of leucine will lead to similar symptoms to those of hypoglycemia (*e.g.*, fatigue, depression and mental issues). This is consistent with the behavior of KYDS rats.^3,4^ BCAAs have been shown to affect gene expression, protein metabolism, apoptosis and the regeneration of hepatocytes, and insulin resistance.^62^ Furthermore, studies have also shown BCAAs are involved in immunity,^63^ the therapy of metabolic disorders and the induction of antioxidant DNA repair.^64^ 11,14-Eicosadienoic acid and 24-methylcholest-5-enyl-3beta-*O*-glucopyranoside_qt are two active compounds of FZ and SY in YGP, and they can act on l-serine dehydratase to regulate valine, leucine and isoleucine biosynthesis [_qt: the residue of saccharide after desaccharification (*i.e.*, aglycone)]. Thus, active compounds in YGP may improve the liver and muscle damage caused by hydrocortisone, and repair damaged DNA by enhancing the antioxidant capacity.

### Aminoacyl-tRNA biosynthesis

Tyrosyl-tRNA synthetase (TyrRS) is a member of the aminoacyl-tRNA synthetases (AARSs) family. It is also an essential component of the translation machinery in the cytoplasm and catalyzes a two-step reaction to link tyrosine to the 3′-end of its cognate tRNA to generate Tyr-tRNA^Tyr^ as a substrate for the ribosome for protein synthesis.^65,66^ Wei *et al.*^67^ have shown that oxidative stress will induce TyrRS to rapidly translocate from the cytosol to the nucleus to protect against DNA damage. This indicates that SD rats are likely to undergo significant oxidative stress after the injection of hydrocortisone. This induces a large amount of TyrRS from the cytoplasm to the nucleus to prevent DNA damage, which in turn affects TyrRS production and tyrosine metabolism. Our previous studies have also found that KYDS has an important relationship with l-tyrosyl-tRNA (Tyr).^35^ Styrone is one of the main components of RG volatile oil, with anti-oxidation and anti-tumor effects.^68^ Halliwell *et al.*^69^ have reported that the antioxidant compounds in RG have obvious inhibitory effects on free radical damage and metabolic syndrome caused by aging. Moreover, Shobana *et al.*^70^ have also found that these antioxidants are capable of significantly inhibiting the oxidation of fatty acids and lipid peroxidation *in vitro*. Thus, styrone in RG maybe can reduce the oxidative stress response, to prevent cell DNA damage, and affect the aminoacyl-tRNA biosynthesis pathway by regulating the biosynthesis of TyrRS back to normal levels.

### Arginine and proline metabolism

Spermine and spermidine widely exist in eukaryotic cells, and mainly exist in human semen. Spermidine is a polyamine that can be produced by *S*-adenosyl methionine (SAM) under the action of spermidine synthase (SRM). The studies of Eisenberg *et al.* have shown that spermidine not only improves life expectancy by inducing autophagy, but also powerfully inhibits cellular necrosis and oxidative stress in aging mice.^71^ Gupta *et al.*^72^ also have reported that polyamines (spermidine, putrescine) can prevent age-related memory impairment through autophagy dependence. Atropine, an active compound of GQ in YGP, can act on SRM to influence the arginine and proline metabolism pathway. This is likely to demonstrate that atropine can improve the activity of SRM to promote the production of spermidine, and treat oxidative stress, memory loss, and cell necrosis caused by KYDS through improving the body's autophagy.

In addition, studies have also shown that spermine and spermidine can protect DNA from free radical oxidative damage.^73,74^ This also suggests that atropine may improve the activity of SRM, not only by promoting the formation of spermidine, but also by promoting spermine formation. And both of these can protect cell DNA from oxidative stress.

### Alanine, aspartate and glutamate metabolism

Alanine, aspartate and glutamate are all glucogenic amino acids that can be converted into glucose to provide energy for the body through gluconeogenesis and glycolysis pathways. The levels of glutamate in KYDS rats are significantly decreased, while the alanine and glutamate content values increase significantly after YGP administration, which indicates that YGP can promote alanine, aspartate and glutamate metabolism.

### d-Glutamine and d-glutamate metabolism

Glutamine can reversibly generate glutamate in the action of enzymes, whereas glutamate can further generate α-ketoglutarate and participate in the TCA cycle.^75^ Ammonia is a toxic substance in the body. The role of glutamate is to convert ammonia to non-toxic glutamine, thereby preventing ammonia poisoning. When liver and kidney function is impaired, urea synthesis is influenced, and the concentration of blood ammonia increases, resulting in hyperammonemia. Meanwhile, when ammonia enters the brain cells, a large amount of α-ketoglutarate in the brain will be consumed, causing TCA cycle weakening. This ultimately leads to a reduction in ATP, and brain dysfunction. After injection with hydrocortisone, the liver and kidney function of KYDS rats was also likely to be affected. This possibly gives rise to the behavioral characteristics of decreased activity and slowed reaction in KYDS rats.^76^

Betaine decomposes to TMAO under the action of intestinal flora. After treatment with YGP, the increased levels of TMAO and betaine show that the activity and metabolism of intestinal flora is enhanced. As a result, the levels of ammonia from proteins decomposed by tissues and gut flora will rise. The body needs to improve the transformation of d-glutamine and d-glutamate metabolism to maintain the balance of ammonia in the body. Additionally, arginine can also be combined with ornithine to promote the urea cycle, thereby improving the conversion of ammonia into urea.

### Ubiquinone and other terpenoid-quinone biosynthesis

Ubiquinone (UQ), also known as coenzyme Q (CoQ), is a class of liposoluble quinone compounds and hydrogen carriers in respiratory chains. The best known functions of CoQ are to act as a mobile electron carrier in the mitochondrial respiratory chain and serve as a liposoluble antioxidant in cellular membranes.^77^ Its antioxidant function against various types of oxidative stress in cellular membranes, lipids, proteins and DNA is also well recognized.^78,79^ CoQ is an electron carrier in oxidative phosphorylation and is at the center of the electron transport chain. Oxidative phosphorylation is the main way to generate ATP *in vivo*. Glucose, fatty acids, glycerol and amino acids can generate acetyl CoA and re-enter the TCA cycle through a series of reactions. The hydrogen generated from the TCA cycle passes through the respiratory chain and eventually combines with oxygen to produce energy. After injection with hydrocortisone, the mitochondrial function of KYDS rats goes into disorder, which also leads to the inhibition of the TCA cycle and energy metabolism.^35^ This may be due to the disordering of ubiquinone and other terpenoid-quinone biosynthesis, which affects the biosynthesis of CoQ. However, after treatment with YGP, the amino acid and energy metabolism in the KYDS rats was strengthened, leading to the ten amino acids screened in this experiment increasing significantly (Table 1). This indicates that the synthesis pathway of CoQ is enhanced, and the function of mitochondria is restored to a certain degree.

Myo-inostitol (MI) is a component of cell membrane phospholipids.^80^ It has immune and protective functions. If an animal lacks myo-inositol, there will be growth stagnation and hair loss. In the KYDS group, the levels of MI decreased, which may be due to cell membrane damage caused by oxidative stress. Hence, this leads to the emergence of growth retardation, weight loss, a loss of hair, *etc.* in KYDS rats. The levels of MI in the YGP group increase significantly, indicating that the active compounds in YGP are likely to produce anti-oxidative stress, which is beneficial to the repair of cell membranes.

### Galactose metabolism

Galactose is converted into 6-phosphate glucose by various enzymes in liver cells. 6-Phosphate glucose produces glucose under the catalysis of glucose 6-phosphatase. Glucose then enters into the glycolysis pathway, so as to provide energy for the body. However, glucose 6-phosphatase exists only in the liver and kidney. Consequently, it may be due to the role of hydrocortisone that the KYDS rat livers and kidneys are damaged, thereby affecting galactose metabolism. In contrast, YGP can regulate galactose metabolism by restoring the functions of livers and kidneys. Furthermore, 6-phosphate glucose can provide phosphoribose and NADPH through the pentose phosphate pathway. NADPH, as a hydrogen donor, can maintain the reduced state of glutathione to protect some of the –SH–containing proteins or enzymes from oxidants. Phosphoribose is a raw material for nucleic acid biosynthesis *in vivo*. Thus, we speculate that YGP can regulate the glycolysis and pentose phosphate pathways to improve energy and pentose phosphate metabolism.

Physiologically, *N*-acetyl glycoprotein (NAG) is an acute inflammatory mediator and expressed more during inflammation and immune response.^81^ The liver is the main organ for synthetic glycoproteins. Concentrations of NAG significantly increase in KYDS rats, manifesting that hydrocortisone is likely to cause inflammation and immune response, whereas after YGP therapy, the levels of NAG significantly descend, which shows that YGP has the potential to treat inflammation and protect the liver.

Obviously, the active compounds from the different traditional Chinese medicines in YGP are basically different, but most of their target proteins and pathways are the same. Meanwhile, most target proteins appear simultaneously in multiple pathways, suggesting that these target proteins carry out coadjustment and signal transmission between different pathways. Studies have shown that complex diseases are often caused by the accumulation of small defects in many genes, rather than big defects caused by a few genes, so treatment by intervening in a single target does not work.^82^ Zhang *et al.*^83^ have reported that treatment of KYDS using Jinkui Shenqi pills worked through a number of protein regulations of multiple signaling pathways. These signaling pathways include Wnt, chemokine, PPAR, and MAPK signaling pathways, *etc.* Overlapping targets among the 5 herbs manifest that different traditional Chinese medicines in YGP will regulate similar targets to exert synergistic effects. For example, many ingredients including 11,14-eicosadienoic acid, karanjin, kadsurenone, ethyl oleate (NF), tetrahydroalstonine and atropine are involved in mediating the activation of betaine-homocysteine *s*-methyltransferase 1, which may provide synergistic therapeutic effects to benefit patients. This also supports the theory that TCM formulas play a curative effect through synergistic mechanisms of multi-component, multi-target and multi-effect overall regulation.

In this study, we find that only five kinds of Chinese medicine in YGP directly regulate KYDS, and the other five Chinese medicines are not involved in regulation. We believe that this may be due to the limitations of the methods and techniques used in our study. For example, when using the OB index, for some ingredients of traditional Chinese medicine, the OB is very poor, so we can't screen the relevant active compounds. Besides, some of the active compounds contained in traditional Chinese medicine themselves need to play a role in the assistance of the multiple metabolisms of intestinal flora. SDH, mainly containing carbohydrates, amino acids, glycosides and trace elements, can regulate the endocrine system, cardiovascular system and immune system, and has anti-oxidant, anti-fatigue and anti-aging functions, can enhance immunity, promote hematopoiesis, and inhibit tumors.^84,85^ LJJ, a solid adhesive made from the decoction and concentration of antlers, mainly contains animal proteins, a variety of amino acids, peptides, hormones, sugar and a small amount of trace elements, and has the effects of nourishing the liver and kidneys, tonifying Yang and being anti-inflammatory.^86–88^ DZ primarily contains lignans, iridoid, phenols, phenylpropenoids, polysaccharides, sterols and triterpenes, and has anti-oxidation, anti-inflammation, and anti-fatigue functions, and shows hepatorenal protection^89,90^ and hypolipidemic effects.^91,92^ DG chiefly contains volatile oils, organic acids, terpenoids, polysaccharides and flavonoids.^93^ It mainly plays a role in the hematopoietic system, immune system and nervous system, and has anti-tumor, anti-inflammation, anti-oxidation and anti-aging effects.^94^ Furthermore, DG also has certain protective effects on the liver and kidneys.^95,96^ TSZ primarily contains flavonoids, polysaccharides, amino acids, alkaloids and other ingredients.^97^ Studies have shown that extracts of TSZ have a sex hormone-like effect, which can increase serum testosterone (T) content and testicular weight, and slow down anaplasia of testicular tissue.^98^ Therefore, the other five kinds of traditional Chinese medicine in YPG should also play a certain role in the treatment of KYDS and remain to be further studied.

## Conclusions

This study combined systems pharmacology and metabonomics to explore the biomarkers of KYDS treated using YGP. Meanwhile, the active constituents and targets of action of various Chinese herbs in YGP were preliminarily explored. Using systems pharmacology to analyze the active components of YGP, 61 active compounds were finally found. These compounds were likely to have an effect on 3177 target proteins and involve 234 pathways. Using metabonomics to analyze the serum of KYDS rats treated with YGP, 21 endogenous biomarkers were found. These biomarkers were mainly involved in 10 metabolic pathways. Combining systems pharmacology and metabonomics, we found that the regulation of KYDS was primarily associated with 19 active compounds from 5 Chinese herbal medicines in YGP. These active compounds mainly had an effect on 8 target proteins, including phosphoenolpyruvate carboxykinase, betaine-homocysteine *s*-methyltransferase 1, alcohol dehydrogenase 1C, *etc.* These target proteins are primarily involved in 6 overlapping pathways, namely: aminoacyl-tRNA biosynthesis; glycolysis/gluconeogenesis; glycine, serine and threonine metabolism; valine, leucine and isoleucine biosynthesis; arginine and proline metabolism; and pyruvate metabolism. In addition, there are four non-overlapping pathways, namely: alanine, aspartate and glutamate metabolism; d-glutamine and d-glutamate metabolism; ubiquinone and other terpenoid-quinone biosynthesis; and galactose metabolism. Metabolic pathway function analysis showed that the therapeutic effects of YGP on KYDS were mainly associated with neuroendocrine regulation, energy metabolism, amino acid metabolism, inflammatory responses, apoptosis, oxidative stress and intestinal flora metabolism. What's more, we also find that YGP possess the potential to protect liver and kidney function.

In summary, the above results preliminarily explore the pathogenesis of KYDS and the pharmacological mechanism of YGP regulating KYDS. To some extent, it also supports the view that TCM formulas are effective through synergistic mechanisms of multi-component, multi-target and multi-effect regulation. This has a certain guiding effect on an in-depth study of KYDS. Our study demonstrated that systems pharmacology and metabonomics methods are novel strategies for explorations of the mechanisms of KYDS and TCM formulas. In the next phase, we will verify the main pathways and target proteins obtained in this study, hoping to thoroughly elucidate the pathogenesis of KYDS and the pharmacological mechanism for YGP regulating KYDS.

## Conflicts of interest

The authors declare no competing financial interests.

## Supplementary Material

RA-008-C7RA12451A-s001
