# Supplementary material for: Exploring the biomarkers and therapeutic mechanism of kidney-yang deficiency syndrome treated by You-gui pill using systems pharmacology and serum metabonomics
Source: RSC Adv. 2018 Jan 3;8(2):1098–115. doi: 10.1039/c7ra12451a (PMC9077015; doi:10.1039/c7ra12451a)
Supplement: RA-008-C7RA12451A-s001 [file RA-008-C7RA12451A-s001.pdf]

Electronic Supplementary Material (ESI) for RSC Advances.
This journal is © The Royal Society of Chemistry 2017

Exploring the Biomarkers and Therapeutic Mechanism of Kidney-Yang Deficiency Syndrome Treated by You-gui Pill Using the Systems Pharmacology and Serum Metabonomics

Ruiqun Chen, Jia Wang, Chengbin Liao, Lei Zhang, Qian Guo and Xiufeng Wang*

School of Basic Courses, Guangdong Pharmaceutical University, Guangzhou 510006, P. R. China. E-mail: wxfsnow8012@126.com; Fax: +86-20-39352187; Tel: +86-20-39352195

Table S1 The active compounds screened from YGP's nine Chinese herbs by TCMSP

Herb	MOL ID	Molecule Name	OB(%) ≧30	BBB>0.3	DL≧0.18	HL ≧4	Pubchem Cid
SDH (*Radix Rehmanniae Preparata*)	MOL000359	sitosterol	36.91	0.87	0.75	5.37	12303645
	MOL000449	Stigmasterol	43.83	1	0.76	5.57	5280794
FZ (Radix Aconiti Lateralis Preparata)	MOL002211	11,14-eicosadienoic acid	39.99	0.76	0.2	5.6	5282805
	MOL002398	Karanjin	69.56	0.62	0.34	13.15	100633
	MOL000359	sitosterol	36.91	0.87	0.75	5.37	12303645
SY (*Rhizoma Dioscoreae*)	MOL005435	24-Methylcholest-5-enyl-3belta-O-glucopyranoside_qt	37.58	0.96	0.72	4.91	N/A
	MOL005438	campesterol	37.58	0.95	0.71	4.83	5283637
	MOL000953	CLR	43.78	1.13	0.68	4.52	5997
	MOL000310	Denudatin B	61.47	0.35	0.38	7.71	442834
	MOL005440	Isofucosterol	43.78	0.97	0.76	5.18	5281326
	MOL000322	Kadsurenone	54.72	0.52	0.38	9.16	122159
	MOL000449	Stigmasterol	43.83	1	0.76	5.57	5280794
SZY (*Macrocarpium Officinale*)	MOL005481	2,6,10,14,18-pentamethylicosa-2,6,10,14,18-pentaene	33.4	1.99	0.24	6.05	5366013
	MOL005486	3,4-Dehydrolycopen-16-al	46.64	0.6	0.49	4.29	5316458
	MOL000358	beta-sitosterol	36.91	0.99	0.75	5.36	222284
	MOL001495	Ethyl linolenate	46.1	1.12	0.2	6.2	6371716
	MOL002883	Ethyl oleate (NF)	32.4	1.1	0.19	4.85	5363269
	MOL005557	lanosta-8,24-dien-3-ol,3-acetate	44.3	1.31	0.82	7.21	N/A
	MOL001494	Mandenol	42	1.14	0.19	5.39	5282184
	MOL001771	poriferast-5-en-3beta-ol	36.91	1.14	0.75	5.07	457801
	MOL000359	sitosterol	36.91	0.87	0.75	5.37	12303645
	MOL000449	Stigmasterol	43.83	1	0.76	5.57	5280794
	MOL008457	Tetrahydroalstonine	32.42	0.33	0.81	10.5	N/A

						5	
**TSZ (*Cuscuta reflexa*)**	MOL000358	beta-sitosterol	36.91	0.99	0.75	5.36	222284
	MOL005043	campest-5-en-3beta-ol	37.58	0.94	0.71	4.43	173183
	MOL000953	CLR	37.87	1.13	0.68	4.52	5997
	MOL005440	Isofucosterol	43.78	0.97	0.76	5.18	5281326
	MOL005944	matrine	63.77	1.52	0.25	6.69	N/A
	MOL000184	NSC63551	39.25	1.22	0.76	5.78	5356634
	MOL006649	sophranol	55.42	0.68	0.28	4.94	N/A
**GQ (*Lycium Barbarum*)**	MOL009612	(24R)-4alpha-Methyl-24-ethylcholesta-7,25-dien-3beta-ylacetate	46.36	1	0.84	8.25	N/A
	MOL009656	(E,E)-1-ethyl octadeca-3,13-dienoate	42	1.11	0.19	5.47	N/A
	MOL009604	14b-pregnane	34.78	1.95	0.34	4.57	N/A
	MOL009617	24-ethylcholest-22-enol	37.09	0.99	0.75	5.32	N/A
	MOL009618	24-ethylcholesta-5,22-dienol	43.83	0.84	0.76	5.76	N/A
	MOL009620	24-methyl-31-norlanost-9(11)-enol	38	0.93	0.75	5.49	5319735
	MOL009621	24-methylenelanost-8-enol	42.37	1.18	0.77	5.43	634628
	MOL007449	24-methylidenelophenol	44.19	1	0.75	5.1	5283640
	MOL009631	31-norcyclolaudenol	38.68	0.94	0.81	4.88	N/A
	MOL009633	31-norlanost-9(11)-enol	38.35	1.03	0.72	5.37	N/A
	MOL009634	31-norlanosterol	42.2	1.03	0.73	5.27	N/A
	MOL009635	4,24-methyllophenol	37.83	1.1	0.75	4.9	N/A
	MOL009642	4alpha-methyl-24-ethylcholesta-7,24-dienol	42.3	1.08	0.78	5.83	N/A
	MOL009640	4alpha,14alpha,24-trimethylcholesta-8,24-dienol	38.91	1.06	0.76	6.67	N/A
	MOL009641	4alpha,24-dimethylcholesta-7,24-dienol	42.65	0.98	0.75	5.44	N/A
	MOL009644	6-Fluoroindole-7-Dehydrocholesterol	43.73	0.54	0.72	5.1	N/A
	MOL009650	Atropine	42.16	0.39	0.19	5.27	637577
	MOL000358	beta-sitosterol	36.91	0.99	0.75	5.36	222284
	MOL005438	campesterol	37.58	0.95	0.71	4.83	N/A
	MOL000953	CLR	37.87	1.13	0.68	4.52	5997
	MOL003578	Cycloartenol	38.69	1.33	0.78	5	N/A
	MOL009653	Cycloeucalenol	39.73	1.04	0.79	5.01	N/A
	MOL008173	daucosterol_qt	36.91	0.85	0.75	5.6	N/A
	MOL001495	Ethyl linolenate	46.1	1.12	0.2	6.2	6371716
	MOL009622	Fucosterol	43.78	1.01	0.76	5.44	5281328
	MOL001979	LAN	42.12	1.18	0.75	5.84	246983
	MOL009677	lanost-8-en-3beta-ol	34.23	1.25	0.74	5.48	440560

	MOL009678	lanost-8-enol	34.23	1.12	0.74	6.41	N/A
	MOL009639	Lophenol	38.13	1.1	0.71	4.76	160482
	MOL001494	Mandenol	42	1.14	0.19	5.39	5282184
	MOL009681	Obtusifoliol	42.55	1.25	0.76	5.91	65252
	MOL001323	Sitosterol alpha1	43.28	0.97	0.78	5.64	9548595
	MOL000449	Stigmasterol	43.83	1	0.76	5.57	5280794
	MOL002223	TAU	24.37	-2.75	0.01	N/A	4068592
	MOL000430	Betaine	40.92	-1.31	0.01	10.78	248
**DZ (*Cortex Eucommiae*)**	MOL009015	(-)-Tabernemontanine	58.67	0.36	0.61	19.73	N/A
	MOL002773	beta-carotene	37.18	1.52	0.58	4.36	5280489
	MOL000358	beta-sitosterol	36.91	0.99	0.75	5.36	222284
	MOL000443	Erythraline	49.18	0.55	0.55	11.11	N/A
**DG (*Radix Angelicae Sinensis*)**	MOL000358	beta-sitosterol	36.91	0.99	0.75	5.36	222284
	MOL000449	Stigmasterol	43.83	1	0.76	5.57	5280794
**RG (*Cinnamomi cortex*)**	MOL000991	Cinnamaldehyde	31.99	1.48	0.02	4.73	637511
	MOL002225	Styrone	38.35	1.09	0.02	4.38	5315892
	MOL000118	(L)-alpha-Terpineol	48.8	1.72	0.03	11.35	443162
	MOL002003	(-)-Caryophyllene oxide	32.67	1.76	0.13	6.51	N/A
	MOL000193	(Z)-caryophyllene	30.29	2.15	0.09	8	6429301
	MOL003047	[(1S)-endo]-(-)-Borneol	83.54	1.43	0.05	11.33	1201518
_qt: the residue of saccharide after desaccharify (i.e., aglycone)							

Table S2 Potential target proteins predicted by PharmMapper

Active compound	Fit	Norm Fit	z-score	Name of target proteins	Uniprot ID
**(L)-alpha-Terpineol**	2.981	0.5962	1.57626	Corticosteroid 11-beta-dehydrogenase isozyme 1	P28845
	2.993	0.4276	1.42131	Beta-secretase 1	BACE1_HUMAN
	2.936	0.5872	1.29723	Mitogen-activated protein kinase 14	Q16539
	2.944	0.5887	1.03352	Tyrosine-protein phosphatase non-receptor type 1	PTN1_HUMAN
	2.823	0.5646	0.813401	Carbonic anhydrase 2	CAH2_HUMAN
**(Z)-caryophyllene**	4.118	0.5883	2.94197	Retinoic acid receptor gamma	RARG_HU

					MAN
	3.934	0.5619	2.49172	Retinoic acid receptor RXR-alpha	RXRA_HUMAN
	3.885	0.5549	2.37535	Peroxisome proliferator-activated receptor gamma	PPARG_HUMAN
	3.664	0.7329	2.2187	Alpha-tocopherol transfer protein	P49638
	3.597	0.5994	2.10954	Cell division protein kinase 2	P24941
	3.987	0.5696	1.78163	Sulfotransferase family cytosolic 2B member 1	O00204
	4.163	0.4625	1.75578	Retinoic acid receptor beta	RARB_HUMAN
	3.654	0.6091	1.72878	Glutathione S-transferase A1	P08263
	3.671	0.4589	1.52394	Caspase-3	CASP3_HUMAN
	3.627	0.6045	1.46724	Adenosine kinase	P55263
	3.42	0.5701	1.39578	NAD(P)H dehydrogenase [quinone] 1	NQO1_HUMAN
	3.447	0.4925	1.29212	Protein farnesyltransferase/geranylgeranyltransferase type-1 subunit alpha	FNTA_HUMAN
	3.881	0.4851	1.23549	Glucocorticoid receptor	GCR_HUMAN
	3.659	0.5228	1.18167	Fatty acid-binding protein, heart	FABPH_HUMAN
	3.317	0.5529	1.01258	Nuclear receptor subfamily 1 group I member 3	NR1I3_HUMAN
	3.589	0.5127	1.00472	Gastrotropin	FABP6_HUMAN
	3.422	0.5703	0.975928	Glutathione-requiring prostaglandin D synthase	PTGD2_HUMAN
	3.504	0.4381	0.942729	Angiopoietin-1 receptor	TIE2_HUMAN
	3.395	0.5658	0.918916	E3 ubiquitin-protein ligase Mdm2	MDM2_HUMAN
	3.293	0.8233	0.875285	Vitamin D-binding protein	VTDB_HUMAN
	3.33	0.4757	0.852318	Cyclin-A2	CCNA2_HUMAN
**[(1S)-endo]-(-)-Borneol**	4.34	0.7233	1.67192	Cell division protein kinase 2	P24941
	4.144	0.5921	1.50468	Collagenase 3	MMP13_HUMAN

	4.33	0.7217	1.41921	Nuclear receptor subfamily 1 group I member 3	NR1I3_HUMAN
	3.977	0.9943	1.36361	Liver carboxylesterase 1	EST1_HUMAN
	3.913	0.6522	1.34751	cAMP-dependent protein kinase catalytic subunit alpha	P00517
	4.481	0.5602	1.32274	Proto-oncogene tyrosine-protein kinase ABL1	P00519
	5.096	0.5096	1.32022	Nuclear receptor ROR-alpha	RORA_HUMAN
	4	0.8	1.3172	Dipeptidyl peptidase 4	DPP4_HUMAN
	3.835	0.9587	1.24744	Estradiol 17-beta-dehydrogenase 1	P14061
	3.915	0.7829	1.24525	Aldo-keto reductase family 1 member C3	AK1C3_HUMAN
	4	0.8	1.23863	Aldo-keto reductase family 1 member C1	Q04828
	3.926	0.7852	1.22668	Catenin alpha-1	CTNA1_HUMAN
	4.041	0.5772	1.21034	cAMP-dependent protein kinase, alpha-catalytic subunit	P00517
	3.779	0.7558	1.20684	Vascular endothelial growth factor receptor 2	P35968
	3.908	0.977	1.19304	Androgen receptor	ANDR_HUMAN
	3.797	0.9494	1.19084	Dual specificity protein phosphatase 6	DUS6_HUMAN
	4.471	0.5589	1.17696	Caspase-3	CASP3_HUMAN
	4.36	0.545	1.17218	Interleukin-2	IL2_HUMAN
	4.296	0.6137	1.15784	Retinoic acid receptor gamma	RARG_HUMAN
	4.44	0.6342	1.14059	Corticosteroid 11-beta-dehydrogenase isozyme 1	DHI1_HUMAN
	4.171	0.5959	1.07931	Peroxisome proliferator-activated receptor gamma	PPARG_HUMAN
	4	0.8	1.07737	Alpha-tocopherol transfer protein	P49638
	4.07	0.5815	1.06542	Bile acid receptor	NR1H4_HUMAN

	4.494	0.5617	1.05571	Ferrochelatase, mitochondrial	P22830
	4.039	0.6732	1.0546	NAD(P)H dehydrogenase [quinone] 1	NQO1_HUMAN
	4.235	0.7058	1.04732	Glutathione S-transferase A1	P08263
	3.985	0.7971	1.00616	Kinesin-like protein KIF11	KIF11_HUMAN
	4.295	0.6136	1.00537	B-Raf proto-oncogene serine/threonine-protein kinase	BRAF1_HUMAN
	4.292	0.7153	1.00433	Glutathione-requiring prostaglandin D synthase	PTGD2_HUMAN
	3.677	0.7355	0.999554	Serine/threonine-protein kinase PAK 7	PAK7_HUMAN
	3.769	0.7539	0.993826	Estrogen receptor	ESR1_HUMAN
	4.44	0.555	0.990837	Retinoic acid receptor alpha	RARA_HUMAN
	4.13	0.6883	0.914888	Serum albumin	ALBU_HUMAN
	4.467	0.6381	0.867713	Sulfotransferase family cytosolic 2B member 1	O00204
	4.408	0.6297	0.861558	Glutathione S-transferase P	GSTP1_HUMAN
	3.995	0.5707	0.826918	Mitogen-activated protein kinase 1	MK01_HUMAN
	3.731	0.6218	0.825349	Aldose reductase	ALDR_HUMAN
	4.408	0.6298	0.818552	Gastrotropin	FABP6_HUMAN
	4.04	0.5772	0.805515	Retinoic acid receptor RXR-alpha	RXRA_HUMAN
	4.034	0.5043	0.80213	Stromelysin-1	MMP3_HUMAN
**24-methylidenelophenol**	4.46	0.8921	2.44713	Estradiol 17-beta-dehydrogenase 1	P14061
	4.28	0.7134	2.20276	Trafficking protein particle complex subunit 3	O43617
	4.336	0.6194	2.1858	Cytochrome P450 2C8	CP2C8_HUMAN
	3.793	0.6321	2.15051	Ganglioside GM2 activator	P17900
	3.869	0.7738	2.09768	Bile acid receptor	NR1H4_HUMAN

	4.197	0.8394	2.07024	SEC14-like protein 2	O76054
	3.684	0.9209	2.06568	Nuclear receptor ROR-alpha	RORA_HUMAN
	3.638	0.7276	1.93886	Alcohol dehydrogenase 1C	P00326
	3.688	0.922	1.91665	Progesterone receptor	PRGR_HUMAN
	3.905	0.7811	1.90802	Calmodulin	CALM_HUMAN
	4.293	0.477	1.8773	Retinoic acid receptor gamma	RARG_HUMAN
	3.616	0.904	1.81349	Mineralocorticoid receptor	MCR_HUMAN
	3.808	0.6346	1.80746	Oxysterols receptor LXR-beta	NR1H2_HUMAN
	3.915	0.7829	1.76398	Corticosteroid 11-beta-dehydrogenase isozyme 1	DHI1_HUMAN
	5.258	0.6572	1.74528	Retinol-binding protein 4	RET4_HUMAN
	3.8	0.4222	1.73201	Phosphatidylinositol 3-kinase regulatory subunit alpha	P85A_HUMAN
	4.387	0.6268	1.71441	Sulfotransferase family cytosolic 2B member 1	O00204
	4.063	0.5804	1.69981	Lanosterol synthase	ERG7_HUMAN
	3.845	0.5492	1.62223	72 kDa type IV collagenase	MMP2_HUMAN
	4.036	0.6727	1.60704	Glutathione-requiring prostaglandin D synthase	PTGD2_HUMAN
	4.325	0.7209	1.57376	Sex hormone-binding globulin	SHBG_HUMAN
	3.628	0.7257	1.5702	Aldo-keto reductase family 1 member C1	Q04828
	4.744	0.5271	1.49297	Dual specificity mitogen-activated protein kinase kinase 1	MP2K1_HUMAN
	3.739	0.7479	1.49189	Tyrosine-protein phosphatase non-receptor type 11	PTN11_HUMAN
	4.106	0.5132	1.46498	Ferrochelatase, mitochondrial	P22830
	3.711	0.6185	1.43814	Oxysterols receptor LXR-alpha	NR1H3_HUMAN
	3.525	0.705	1.40421	cAMP-dependent protein kinase catalytic subunit alpha	P00517

	3.945	0.4931	1.3943	Caspase-3	CASP3_HUMAN
	3.827	0.6378	1.38493	Tyrosine-protein kinase SYK	KSYK_HUMAN
	3.46	0.692	1.36976	Glutathione S-transferase P	GSTP1_HUMAN
	3.42	0.855	1.27706	Collagenase 3	MMP13_HUMAN
	4.067	0.5809	1.26404	Gastrotropin	FABP6_HUMAN
	3.437	0.6874	1.25802	Dipeptidyl peptidase 4	DPP4_HUMAN
	3.991	0.5701	1.25734	Estrogen sulfotransferase	ST1E1_HUMAN
	3.681	0.5258	1.24592	Interleukin-2	IL2_HUMAN
	4.219	0.4219	1.19237	Vitamin D3 receptor	VDR_HUMAN
	3.904	0.488	1.15581	Retinoic acid receptor alpha	RARA_HUMAN
	4.21	0.4677	1.12278	Retinoic acid receptor beta	RARB_HUMAN
	5.789	0.5789	1.11325	Cellular retinoic acid-binding protein 2	P29373
	3.679	0.6132	1.09797	Glutathione S-transferase A1	P08263
	3.703	0.4628	1.07395	Proto-oncogene tyrosine-protein kinase ABL1	P00519
	3.588	0.7177	1.07101	Wiskott-Aldrich syndrome protein	WASP_HUMAN
	3.743	0.6239	1.04988	Adenosine kinase	P55263
	3.457	0.5762	1.0104	Flavin reductase	P30043
	3.558	0.8894	0.992235	Vitamin D-binding protein	VTDB_HUMAN
	3.937	0.3028	0.988948	Endothelial protein C receptor	EPCR_HUMAN
	3.784	0.6306	0.979143	Alcohol dehydrogenase class-3	ADHX_HUMAN
	4.072	0.3702	0.971697	Serine/threonine-protein phosphatase PP1-gamma catalytic subunit	P36873
	3.298	0.6596	0.967547	Steroid hormone receptor ERR1	P11474

	3.994	0.5705	0.965136	Fatty acid-binding protein, brain	FABP7_HUMAN
	3.689	0.4099	0.839516	Growth factor receptor-bound protein 2	GRB2_HUMAN
	3.715	0.5307	0.816262	Hepatocyte nuclear factor 4-gamma	Q14541
**beta-carotene**	7.764	0.9705	3.50164	Retinol-binding protein 4	RET4_HUMAN
	5.157	0.6446	2.6582	Caspase-3	CASP3_HUMAN
	4.565	0.7609	2.51984	ADAM 17	ADA17_HUMAN
	5.238	0.403	2.19371	Endothelial protein C receptor	EPCR_HUMAN
	4.697	0.671	2.12558	Retinoic acid receptor gamma	RARG_HUMAN
	4.822	0.6889	2.12104	Cytochrome P450 2C8	CP2C8_HUMAN
	4.481	0.7468	2.03589	Aldose reductase	ALDR_HUMAN
	4.736	0.7894	1.94415	Glutathione-requiring prostaglandin D synthase	PTGD2_HUMAN
	4.678	0.5848	1.92629	Proto-oncogene tyrosine-protein kinase ABL1	P00519
	4.185	0.837	1.79232	Bile acid receptor	NR1H4_HUMAN
	4.552	0.7587	1.79058	Trafficking protein particle complex subunit 3	O43617
	5.225	0.5806	1.74393	Retinoic acid receptor beta	RARB_HUMAN
	5.175	0.6469	1.68983	Glucocorticoid receptor	GCR_HUMAN
	4	0.6667	1.62991	Peroxisome proliferator-activated receptor delta	PPARD_HUMAN
	4.903	0.7004	1.56835	Fatty acid-binding protein, brain	FABP7_HUMAN
	4.523	0.7538	1.55198	Carbonic anhydrase 2	CAH2_HUMAN
	4.353	0.7255	1.45942	[Pyruvate dehydrogenase [lipoamide]] kinase isozyme 2, mitochondrial	PDK2_HUMAN
	4	0.5714	1.45272	3-hydroxy-3-methylglutaryl-	HMDH_HU

				coenzyme A reductase	MAN
	4.532	0.6474	1.40545	Hepatocyte nuclear factor 4-gamma	Q14541
	5.145	0.5717	1.29648	Dual specificity mitogen-activated protein kinase kinase 1	MP2K1_HU MAN
	4.534	0.6477	1.27572	Fatty acid-binding protein, heart	FABPH_HU MAN
	4.713	0.5892	1.26275	Basic fibroblast growth factor receptor 1	FGFR1_HU MAN
	6.752	0.6752	1.26104	Cellular retinoic acid-binding protein 2	P29373
	4.465	0.6378	1.25022	Estrogen sulfotransferase	ST1E1_HU MAN
	4.197	0.8394	1.20654	SEC14-like protein 2	O76054
	3.731	0.9329	1.17688	Nuclear receptor ROR-alpha	RORA_HU MAN
	4.017	0.6696	1.15771	Mitogen-activated protein kinase 10	P53779
	4	0.5714	1.12362	72 kDa type IV collagenase	MMP2_HU MAN
	4	0.8	1.11593	Tyrosine-protein phosphatase non-receptor type 11	PTN11_HU MAN
	3.975	0.7951	1.09521	Calmodulin	CALM_HU MAN
	3.87	0.645	1.08324	Poly [ADP-ribose] polymerase 1	P09874
	3.697	0.7393	1.02593	E3 ubiquitin-protein ligase Mdm2	MDM2_HU MAN
	4.624	0.4204	1.02151	Phosphatidylcholine transfer protein	Q9UKL6
	4.169	0.6948	1.0159	Adenosine kinase	P55263
	3.718	0.7437	0.99698	Dipeptidyl peptidase 4	DPP4_HUM AN
	4.106	0.5866	0.974521	Lanosterol synthase	ERG7_HU MAN
	3.938	0.6563	0.969188	Nuclear receptor subfamily 1 group I member 3	NR1I3_HU MAN
	3.644	0.9111	0.965844	Cell division protein kinase 2	P24941
	3.732	0.7465	0.959513	cAMP-dependent protein kinase catalytic subunit alpha	P00517
	4.164	0.4627	0.955637	Serine/threonine-protein phosphatase PP1-gamma	P36873

				catalytic subunit	
	3.836	0.6394	0.950185	Flavin reductase	P30043
	3.744	0.7488	0.935588	Vascular endothelial growth factor receptor 2	P35968
	3.886	0.6476	0.907668	Oxysterols receptor LXR-alpha	NR1H3_HUMAN
	3.654	0.609	0.894117	Ganglioside GM2 activator	P17900
	3.811	0.4764	0.886762	Vitamin D3 receptor	VDR_HUMAN
	3.815	0.6359	0.870472	Receptor tyrosine-protein kinase erbB-4	ERBB4_HUMAN
	4.811	0.4811	0.858882	Retinoic acid receptor RXR-beta	RXRB_HUMAN
	3.622	0.9056	0.851415	Mitogen-activated protein kinase 14	Q16539
	3.854	0.7707	0.838113	Prothrombin	THRB_HUMAN
	3.966	0.6611	0.82961	Glutathione S-transferase A1	P08263
	4.408	0.551	0.82659	Histamine N-methyltransferase	P50135
	3.78	0.63	0.826106	Tyrosine-protein phosphatase non-receptor type 1	PTN1_HUMAN
	3.668	0.7337	0.821063	Aldo-keto reductase family 1 member C1	Q04828
	3.628	0.7256	0.813768	Glutathione S-transferase P	GSTP1_HUMAN
	3.862	0.5517	0.80421	Interleukin-2	IL2_HUMAN
**betaine**	3.438	0.3438	0.63133	Sulfotransferase family cytosolic 2B member 1	O00204
	2.645	0.5291	0.540883	Complement component C8 gamma chain	P07360
	2.734	0.3417	0.414054	Ornithine aminotransferase, mitochondrial	OAT_HUMAN
	2.691	0.299	0.30178	Bifunctional heparan sulfate N-deacetylase/N-sulfotransferase 1	P52848
	2.656	0.332	0.0310912	Pyruvate kinase isozymes R/L	KPYR_HUMAN
	2.622	0.3746	-0.0211379	Cystathionine beta-synthase	P35520
	2.7	0.27	-	Nuclear receptor ROR-alpha	RORA_HU

			0.0669725		MAN
	2.827	0.1885	-0.406964	GTPase HRas	RASH_HUMAN
	2.254	0.2049	-0.979351	Glutathione S-transferase P	GSTP1_HUMAN
	2.129	0.3042	-1.11439	Heat shock 70 kDa protein 1	P08107
	2.285	0.2285	-1.15144	Eukaryotic translation initiation factor 4E	P06730
	2.236	0.559	-1.20921	Proto-oncogene tyrosine-protein kinase Src	SRC_HUMAN
	2.101	0.4202	-1.53595	Tyrosine-protein phosphatase non-receptor type 1	PTN1_HUMAN
**beta-sitosterol**	4.634	0.9268	2.81101	Estradiol 17-beta-dehydrogenase 1	P14061
	4.544	0.9087	2.78011	SEC14-like protein 2	O76054
	3.822	0.9555	2.29261	Progesterone receptor	PRGR_HUMAN
	3.777	0.9443	2.16035	Mineralocorticoid receptor	MCR_HUMAN
	3.657	0.9143	2.00799	Nuclear receptor ROR-alpha	RORA_HUMAN
	4.145	0.6909	1.97081	Trafficking protein particle complex subunit 3	O43617
	4.18	0.5972	1.9412	Lanosterol synthase	ERG7_HUMAN
	3.707	0.9268	1.93272	Collagenase 3	MMP13_HUMAN
	3.734	0.7468	1.9228	Cell division protein kinase 2	P24941
	4.19	0.5238	1.89654	Caspase-3	CASP3_HUMAN
	3.928	0.6547	1.8857	Nuclear receptor subfamily 1 group I member 3	NR1I3_HUMAN
	4.46	0.6371	1.85349	Sulfotransferase family cytosolic 2B member 1	O00204
	3.71	0.742	1.73461	Bile acid receptor	NR1H4_HUMAN
	3.734	0.7467	1.66682	Androgen receptor	ANDR_HUMAN
	4.177	0.5967	1.66366	Hepatocyte nuclear factor 4-gamma	Q14541

	3.755	0.4173	1.64121	Phosphatidylinositol 3-kinase regulatory subunit alpha	P85A_HUMAN
	4.192	0.5241	1.62492	Ferrochelatase, mitochondrial	P22830
	4.239	0.5299	1.62305	Retinoic acid receptor gamma	RARG_HUMAN
	4.019	0.6698	1.59619	Glutathione-requiring prostaglandin D synthase	PTGD2_HUMAN
	4.322	0.7204	1.59286	Sex hormone-binding globulin	SHBG_HUMAN
	3.903	0.6506	1.5715	Glutathione S-transferase A1	P08263
	5.046	0.6307	1.5483	Retinol-binding protein 4	RET4_HUMAN
	3.75	0.6251	1.54055	Oxysterols receptor LXR-alpha	NR1H3_HUMAN
	3.946	0.5637	1.47763	Cytochrome P450 2C8	CP2C8_HUMAN
	4.703	0.5226	1.47021	Dual specificity mitogen-activated protein kinase kinase 1	MP2K1_HUMAN
	3.707	0.7413	1.46111	Calmodulin	CALM_HUMAN
	4.169	0.5955	1.44663	Gastrotropin	FABP6_HUMAN
	3.482	0.5804	1.4284	Peroxisome proliferator-activated receptor delta	PPARD_HUMAN
	4.187	0.3221	1.42386	Endothelial protein C receptor	EPCR_HUMAN
	3.669	0.7337	1.41053	Prothrombin	THRB_HUMAN
	3.74	0.7481	1.39333	Corticosteroid 11-beta-dehydrogenase isozyme 1	DHI1_HUMAN
	3.661	0.6101	1.37026	NAD(P)H dehydrogenase [quinone] 1	NQO1_HUMAN
	3.71	0.6184	1.35393	Ephrin type-B receptor 4	EPHB4_HUMAN
	3.884	0.6473	1.34656	Adenosine kinase	P55263
	3.482	0.6964	1.33394	Dipeptidyl peptidase 4	DPP4_HUMAN
	3.679	0.5256	1.32281	72 kDa type IV collagenase	MMP2_HUMAN
	3.66	0.7319	1.31952	Tyrosine-protein phosphatase non-receptor type 11	PTN11_HUMAN

	3.485	0.268	1.28339	Amine oxidase [flavin-containing] A	AOFA_HUMAN
	3.586	0.4482	1.25577	Vitamin D3 receptor	VDR_HUMAN
	3.753	0.6256	1.24744	Tyrosine-protein kinase SYK	KSYK_HUMAN
	3.648	0.608	1.19157	Oxysterols receptor LXR-beta	NR1H2_HUMAN
	3.302	0.8254	1.15543	Bile salt sulfotransferase	Q06520
	3.96	0.5657	1.1333	Mitogen-activated protein kinase 10	MK10_HUMAN
	3.512	0.4389	1.12762	Heat shock protein HSP 90-beta	P08238
	3.642	0.5203	1.03581	Insulin-like growth factor IA	P01343
	3.561	0.5087	1.01689	Interleukin-2	IL2_HUMAN
	3.768	0.4186	1.0084	Growth factor receptor-bound protein 2	GRB2_HUMAN
	3.966	0.4407	1.00788	Angiopoietin-1 receptor	TIE2_HUMAN
	3.651	0.4564	0.98817	Proto-oncogene tyrosine-protein kinase ABL1	P00519
	3.619	0.517	0.985734	Tyrosine-protein kinase ZAP-70	ZAP70_HUMAN
	5.533	0.5533	0.961073	Cellular retinoic acid-binding protein 2	P29373
	3.433	0.5721	0.95347	Flavin reductase	P30043
	3.74	0.4156	0.889179	Mast/stem cell growth factor receptor	KIT_HUMAN
	3.949	0.5642	0.874001	Lactoylglutathione lyase	LGUL_HUMAN
	3.507	0.8767	0.872717	Vitamin D-binding protein	VTDB_HUMAN
	3.313	0.8282	0.863641	Estrogen receptor	ESR1_HUMAN
	3.598	0.514	0.831692	Protein kinase C theta type	KPCT_HUMAN
	3.398	0.5664	0.831259	Peroxisome proliferator-activated receptor alpha	PPARA_HUMAN
	3.362	0.6725	0.82951	Alpha-tocopherol transfer protein	P49638
**campest-5-en-3beta-ol**	4.633	0.9266	2.6347	Estradiol 17-beta-dehydrogenase 1	P14061

	3.91	0.9775	2.48791	Nuclear receptor ROR-alpha	RORA_HUMAN
	4.455	0.891	2.43423	SEC14-like protein 2	O76054
	4.533	0.5666	2.13007	Retinoic acid receptor alpha	RARA_HUMAN
	3.822	0.9556	2.10957	Progesterone receptor	PRGR_HUMAN
	3.779	0.7557	2.05075	Glutathione S-transferase P	GSTP1_HUMAN
	3.782	0.9454	2.03093	Mineralocorticoid receptor	MCR_HUMAN
	4.236	0.706	1.84385	Glutathione-requiring prostaglandin D synthase	PTGD2_HUMAN
	3.711	0.7421	1.79628	Histone deacetylase 8	Q9BY41
	4.13	0.6884	1.79281	Trafficking protein particle complex subunit 3	O43617
	4.493	0.6419	1.76859	Sulfotransferase family cytosolic 2B member 1	O00204
	3.734	0.7468	1.74688	Dipeptidyl peptidase 4	DPP4_HUMAN
	4.193	0.5241	1.74677	Caspase-3	CASP3_HUMAN
	3.769	0.7537	1.71744	Bile acid receptor	NR1H4_HUMAN
	4.427	0.7379	1.62111	Sex hormone-binding globulin	SHBG_HUMAN
	3.613	0.9034	1.57831	Collagenase 3	MMP13_HUMAN
	5.13	0.6413	1.5231	Retinol-binding protein 4	RET4_HUMAN
	4.168	0.5954	1.50574	Hepatocyte nuclear factor 4-gamma	Q14541
	4.196	0.5245	1.50278	Ferrochelatase, mitochondrial	P22830
	4.09	0.4545	1.50266	Growth factor receptor-bound protein 2	GRB2_HUMAN
	4.239	0.5299	1.49282	Retinoic acid receptor gamma	RARG_HUMAN
	3.649	0.7297	1.48718	Aldo-keto reductase family 1 member C1	Q04828
	4.81	0.5345	1.48229	Dual specificity mitogen-activated protein kinase kinase 1	MP2K1_HUMAN

	3.839	0.7678	1.462	Corticosteroid 11-beta-dehydrogenase isozyme 1	DHI1_HUMAN
	3.861	0.6435	1.44545	Tyrosine-protein kinase ITK/TSK	Q08881
	3.796	0.5423	1.41753	72 kDa type IV collagenase	MMP2_HUMAN
	3.716	0.4129	1.39375	Phosphatidylinositol 3-kinase regulatory subunit alpha	P85A_HUMAN
	3.749	0.6249	1.38744	Oxysterols receptor LXR-alpha	NR1H3_HUMAN
	3.771	0.6284	1.37998	Tyrosine-protein kinase JAK3	JAK3_HUMAN
	3.859	0.5512	1.32582	Tyrosine-protein kinase ZAP-70	ZAP70_HUMAN
	3.85	0.6417	1.32008	Glutathione S-transferase A1	P08263
	3.491	0.8727	1.31714	Mitogen-activated protein kinase 14	Q16539
	4.152	0.5932	1.2944	Gastrotropin	FABP6_HUMAN
	3.805	0.6342	1.28252	Proto-oncogene tyrosine-protein kinase LCK	LCK_HUMAN
	3.921	0.6536	1.27855	Adenosine kinase	P55263
	3.906	0.5579	1.26882	Cytochrome P450 2C8	CP2C8_HUMAN
	3.655	0.6091	1.23257	NAD(P)H dehydrogenase [quinone] 1	NQO1_HUMAN
	3.657	0.7314	1.19477	Calmodulin	CALM_HUMAN
	3.663	0.7326	1.17804	Tyrosine-protein phosphatase non-receptor type 11	PTN11_HUMAN
	3.48	0.696	1.15836	cAMP-dependent protein kinase catalytic subunit alpha	P00517
	3.932	0.3574	1.14261	Caspase-1	CASP1_HUMAN
	3.584	0.448	1.12456	Vitamin D3 receptor	VDR_HUMAN
	3.665	0.6108	1.09111	Oxysterols receptor LXR-beta	NR1H2_HUMAN
	3.801	0.543	1.08122	Protein kinase C theta type	KPCT_HUMAN
	4.047	0.3113	1.06413	Endothelial protein C receptor	EPCR_HU

					MAN
	3.302	0.8255	1.03312	Bile salt sulfotransferase	Q06520
	3.739	0.4674	1.03005	Proto-oncogene tyrosine-protein kinase ABL1	P00519
	4.19	0.4656	1.00265	Retinoic acid receptor beta	RARB_HUMAN
	5.634	0.5634	0.946069	Cellular retinoic acid-binding protein 2	P29373
	3.702	0.617	0.91224	E3 ubiquitin-protein ligase Mdm2	MDM2_HUMAN
	3.552	0.7103	0.859482	Wiskott-Aldrich syndrome protein	WASP_HUMAN
	3.541	0.5058	0.859013	Interleukin-2	IL2_HUMAN
	3.299	0.6598	0.850569	Steroid hormone receptor ERR1	P11474
	3.324	0.2557	0.803257	Amine oxidase [flavin-containing] A	AOFA_HUMAN
**CLR**	4.634	0.9268	2.6817	Estradiol 17-beta-dehydrogenase 1	P14061
	5.986	0.7482	2.46445	Retinol-binding protein 4	RET4_HUMAN
	3.867	0.9667	2.42975	Nuclear receptor ROR-alpha	RORA_HUMAN
	4.395	0.7325	2.33376	Trafficking protein particle complex subunit 3	O43617
	3.873	0.9683	2.19799	Collagenase 3	MMP13_HUMAN
	3.822	0.9554	2.16179	Progesterone receptor	PRGR_HUMAN
	3.761	0.9402	2.02802	Mineralocorticoid receptor	MCR_HUMAN
	4.424	0.632	1.99686	Hepatocyte nuclear factor 4-gamma	Q14541
	3.797	0.7595	1.82899	Bile acid receptor	NR1H4_HUMAN
	4.112	0.8224	1.82075	SEC14-like protein 2	O76054
	4.487	0.641	1.79521	Sulfotransferase family cytosolic 2B member 1	O00204
	3.889	0.6481	1.74525	Oxysterols receptor LXR-alpha	NR1H3_HUMAN
	3.929	0.7859	1.70132	Corticosteroid 11-beta-	DHI1_HUM

				dehydrogenase isozyme 1	AN
	3.651	0.7302	1.65235	Steroid hormone receptor ERR1	P11474
	4.09	0.5843	1.64625	Cytochrome P450 2C8	CP2C8_HUMAN
	4.416	0.736	1.63884	Sex hormone-binding globulin	SHBG_HUMAN
	4.049	0.5784	1.59364	Lanosterol synthase	ERG7_HUMAN
	3.736	0.7471	1.58158	Androgen receptor	ANDR_HUMAN
	3.578	0.8944	1.57217	Mitogen-activated protein kinase 14	Q16539
	4.196	0.5245	1.53766	Ferrochelatase, mitochondrial	P22830
	3.999	0.6664	1.47217	Adenosine kinase	P55263
	3.726	0.414	1.46183	Phosphatidylinositol 3-kinase regulatory subunit alpha	P85A_HUMAN
	3.763	0.7525	1.4518	Tyrosine-protein phosphatase non-receptor type 11	PTN11_HUMAN
	6.317	0.6317	1.43777	Cellular retinoic acid-binding protein 2	P29373
	3.591	0.7181	1.4024	Aldo-keto reductase family 1 member C1	Q04828
	3.648	0.608	1.3615	Oxysterols receptor LXR-beta	NR1H2_HUMAN
	3.69	0.7381	1.34813	Prothrombin	THRB_HUMAN
	4.156	0.5937	1.33471	Gastrotropin	FABP6_HUMAN
	3.535	0.707	1.33292	cAMP-dependent protein kinase catalytic subunit alpha	P00517
	3.625	0.7251	1.31328	Alpha-tocopherol transfer protein	P49638
	3.706	0.5295	1.28768	72 kDa type IV collagenase	MMP2_HUMAN
	4.608	0.512	1.2647	Dual specificity mitogen-activated protein kinase kinase 1	MP2K1_HUMAN
	3.654	0.7308	1.23132	Calmodulin	CALM_HUMAN
	3.777	0.5395	1.22742	Retinoic acid receptor gamma	RARG_HUMAN
	3.443	0.6885	1.22254	Glutathione S-transferase P	GSTP1_HUMAN

	3.839	0.4799	1.10739	Caspase-3	CASP3_HUMAN
	3.795	0.6325	1.1043	Glutathione-requiring prostaglandin D synthase	PTGD2_HUMAN
	3.638	0.9095	1.08919	Vitamin D-binding protein	VTDB_HUMAN
	3.71	0.6184	1.07791	Glutathione S-transferase A1	P08263
	3.302	0.8255	1.07168	Bile salt sulfotransferase	Q06520
	4.211	0.4679	1.06192	Retinoic acid receptor beta	RARB_HUMAN
	3.403	0.6805	1.05043	Histone deacetylase 8	Q9BY41
	3.513	0.4392	1.04021	Heat shock protein HSP 90-beta	P08238
	3.572	0.5103	1.02575	Basic fibroblast growth factor receptor 1	FGFR1_HUMAN
	3.634	0.6056	0.992895	Tyrosine-protein kinase ITK/TSK	Q08881
	4.14	0.3764	0.975245	Phosphatidylcholine transfer protein	Q9UKL6
	3.489	0.4361	0.967962	Vitamin D3 receptor	VDR_HUMAN
	3.791	0.4212	0.958933	Growth factor receptor-bound protein 2	GRB2_HUMAN
	3.559	0.5932	0.954278	Tyrosine-protein kinase JAK3	JAK3_HUMAN
	3.472	0.5787	0.951397	Flavin reductase	P30043
	3.803	0.4754	0.906898	Retinoic acid receptor alpha	RARA_HUMAN
	3.923	0.3017	0.895779	Endothelial protein C receptor	EPCR_HUMAN
	3.46	0.5766	0.885193	Peroxisome proliferator-activated receptor alpha	PPARA_HUMAN
	3.531	0.5044	0.874946	Interleukin-2	IL2_HUMAN
	3.664	0.5234	0.871111	Protein kinase C theta type	KPCT_HUMAN
	3.582	0.5118	0.827724	Tyrosine-protein kinase ZAP-70	ZAP70_HUMAN
	3.432	0.572	0.824609	Receptor tyrosine-protein kinase erbB-4	ERBB4_HUMAN
	3.556	0.5926	0.821566	Proto-oncogene tyrosine-protein kinase LCK	LCK_HUMAN

	3.976	0.4969	0.821468	Histamine N-methyltransferase	P50135
	3.576	0.5109	0.812765	Insulin-like growth factor IA	P01343
	3.95	0.5643	0.802897	Lactoylglutathione lyase	LGUL_HUMAN
**Erythraline**	3.656	0.5223	4.55052	Peptidyl-prolyl cis-trans isomerase FKBP1A	FKB1A_HUMAN
	3.175	0.6351	3.16491	Glycogen synthase kinase-3 beta	P49841
	2.959	0.4932	2.4533	Peroxisome proliferator-activated receptor gamma	PPARG_HUMAN
	2.856	0.4759	2.15158	Fatty acid-binding protein, adipocyte	FABP4_HUMAN
	2.922	0.4869	2.09862	Prothrombin	THRB_HUMAN
	2.85	0.57	2.05281	Neutrophil collagenase	MMP8_HUMAN
	2.946	0.5893	1.99344	Mitogen-activated protein kinase 14	Q16539
	3.44	0.4301	1.96935	Dipeptidase 1	DPEP1_HUMAN
	2.979	0.4964	1.95581	Peroxisome proliferator-activated receptor alpha	PPARA_HUMAN
	2.84	0.71	1.93947	cAMP-specific 3,5-cyclic phosphodiesterase 4D	PDE4D_HUMAN
	2.912	0.3641	1.92086	Signal transducer and activator of transcription 1-alpha/beta	STAT1_HUMAN
	2.784	0.928	1.88023	Glutathione S-transferase P	GSTP1_HUMAN
	2.804	0.5609	1.66234	Serine protease hepsin	P05981
	2.982	0.2711	1.6096	Beta-secretase 1	P56817
	2.923	0.5846	1.60178	Sorbitol dehydrogenase	DHSO_HUMAN
	3.197	0.3997	1.52061	Glucokinase	HXK4_HUMAN
	2.713	0.6782	1.44664	Proto-oncogene tyrosine-protein kinase LCK	LCK_HUMAN
	2.691	0.6728	1.42423	Proto-oncogene tyrosine-protein kinase Src	SRC_HUMAN
	2.793	0.4655	1.35128	Stromelysin-1	MMP3_HUMAN
	2.747	0.5495	1.3291	Fibroblast growth factor receptor 2	P21802

	2.82	0.564	1.2905	Transthyretin	TTHY_HUMAN
	2.9	0.3222	1.25559	Glutathione S-transferase Mu 2	GSTM2_HUMAN
	2.631	0.6577	1.24139	NONE	NONE
	2.801	0.5601	1.22729	Tyrosine-protein phosphatase non-receptor type 1	PTN1_HUMAN
	2.762	0.3069	1.14704	Bcl-2-like protein 1	Q07817
	2.912	0.4161	1.11992	Tyrosine-protein kinase JAK2	JAK2_HUMAN
	2.782	0.3975	1.06167	3-phosphoinositide-dependent protein kinase 1	PDPK1_HUMAN
	2.734	0.4557	1.05779	Leukocyte elastase	ELNE_HUMAN
	2.58	0.8599	1.00837	Carbonic anhydrase 2	CAH2_HUMAN
	2.691	0.2691	1.00069	Tryptase beta-2	TRYB2_HUMAN
	2.864	0.5728	0.976676	Estradiol 17-beta-dehydrogenase 1	P14061
	3.119	0.3899	0.921363	Fatty acid-binding protein, heart	FABPH_HUMAN
	2.549	0.5098	0.839082	Cathepsin S	CATS_HUMAN
	2.668	0.4446	0.830901	Heat shock protein HSP 90-alpha	HS90A_HUMAN
**Isofucosterol**	4.634	0.9267	2.65166	Estradiol 17-beta-dehydrogenase 1	P14061
	3.951	0.9878	2.60536	Nuclear receptor ROR-alpha	RORA_HUMAN
	3.884	0.7768	2.25273	Histone deacetylase 8	Q9BY41
	4.46	0.4955	2.23755	Growth factor receptor-bound protein 2	GRB2_HUMAN
	4.345	0.869	2.23745	SEC14-like protein 2	O76054
	4.299	0.7166	2.12255	Trafficking protein particle complex subunit 3	O43617
	3.822	0.9555	2.12132	Progesterone receptor	PRGR_HUMAN
	3.776	0.7551	2.05405	Glutathione S-transferase P	GSTP1_HUMAN
	4.208	0.7013	2.05079	Glutathione S-transferase A1	P08263

	3.783	0.9458	2.04174	Mineralocorticoid receptor	MCR_HUMAN
	4.447	0.6353	2.00914	Hepatocyte nuclear factor 4-gamma	Q14541
	4.22	0.6028	1.87813	Lanosterol synthase	ERG7_HUMAN
	3.829	0.7659	1.86807	Bile acid receptor	NR1H4_HUMAN
	4.746	0.4746	1.84442	Vitamin D3 receptor	VDR_HUMAN
	3.892	0.7783	1.81799	Prothrombin	THRB_HUMAN
	4.485	0.6408	1.76791	Sulfotransferase family cytosolic 2B member 1	O00204
	4.399	0.6284	1.75439	Mitogen-activated protein kinase 10	MK10_HUMAN
	3.953	0.7906	1.71983	Corticosteroid 11-beta-dehydrogenase isozyme 1	DHI1_HUMAN
	4.447	0.7411	1.66043	Sex hormone-binding globulin	SHBG_HUMAN
	3.85	0.7701	1.64747	Calmodulin	CALM_HUMAN
	4.231	0.5289	1.62091	Retinoic acid receptor alpha	RARA_HUMAN
	3.866	0.6444	1.60149	Nuclear receptor subfamily 1 group I member 3	NR1I3_HUMAN
	3.743	0.7486	1.56607	Androgen receptor	ANDR_HUMAN
	4.057	0.5795	1.55642	Cytochrome P450 2C8	CP2C8_HUMAN
	5.131	0.6413	1.53532	Retinol-binding protein 4	RET4_HUMAN
	4.239	0.5299	1.50291	Retinoic acid receptor gamma	RARG_HUMAN
	4.182	0.5228	1.49037	Ferrochelatase, mitochondrial	P22830
	3.722	0.4136	1.41697	Phosphatidylinositol 3-kinase regulatory subunit alpha	P85A_HUMAN
	4.789	0.4789	1.3769	Retinoic acid receptor RXR-beta	RXRB_HUMAN
	3.504	0.8759	1.34393	Collagenase 3	MMP13_HUMAN

	3.755	0.6258	1.31953	Ephrin type-B receptor 4	EPHB4_HUMAN
	4.655	0.5173	1.30256	Dual specificity mitogen-activated protein kinase kinase 1	MP2K1_HUMAN
	3.735	0.9337	1.28737	Vitamin D-binding protein	VTDB_HUMAN
	3.918	0.653	1.28233	Adenosine kinase	P55263
	3.494	0.6988	1.23897	Dipeptidyl peptidase 4	DPP4_HUMAN
	4.105	0.5865	1.23012	Gastrotropin	FABP6_HUMAN
	3.506	0.7013	1.2275	cAMP-dependent protein kinase catalytic subunit alpha	P00517
	3.68	0.736	1.22571	Tyrosine-protein phosphatase non-receptor type 11	PTN11_HUMAN
	3.496	0.6992	1.16441	Aldo-keto reductase family 1 member C1	Q04828
	3.643	0.6071	1.15899	Oxysterols receptor LXR-alpha	NR1H3_HUMAN
	3.803	0.4754	1.15209	Proto-oncogene tyrosine-protein kinase ABL1	P00519
	3.682	0.6137	1.13523	Oxysterols receptor LXR-beta	NR1H2_HUMAN
	3.58	0.5967	1.11689	Receptor tyrosine-protein kinase erbB-4	ERBB4_HUMAN
	3.823	0.4778	1.04702	Caspase-3	CASP3_HUMAN
	3.302	0.8254	1.03785	Bile salt sulfotransferase	Q06520
	3.577	0.511	1.02172	72 kDa type IV collagenase	MMP2_HUMAN
	3.751	0.6252	1.0187	E3 ubiquitin-protein ligase Mdm2	MDM2_HUMAN
	3.637	0.6062	0.953957	Proto-oncogene tyrosine-protein kinase LCK	LCK_HUMAN
	3.39	0.2608	0.952463	Amine oxidase [flavin-containing] A	AOFA_HUMAN
	3.771	0.419	0.941721	Serine/threonine-protein phosphatase PP1-gamma catalytic subunit	P36873
	3.962	0.3048	0.934209	Endothelial protein C receptor	EPCR_HUMAN

	4.124	0.3749	0.928225	Phosphatidylcholine transfer protein	Q9UKL6
	4.125	0.4583	0.921475	Retinoic acid receptor beta	RARB_HUMAN
	3.806	0.6344	0.91398	Alcohol dehydrogenase class-3	ADHX_HUMAN
	3.552	0.5074	0.886505	Interleukin-2	IL2_HUMAN
	3.681	0.6134	0.876165	Glutathione-requiring prostaglandin D synthase	PTGD2_HUMAN
	3.453	0.5756	0.867518	Tyrosine-protein phosphatase non-receptor type 1	PTN1_HUMAN
	3.612	0.516	0.856572	Tyrosine-protein kinase ZAP-70	ZAP70_HUMAN
	3.609	0.6014	0.830198	Tyrosine-protein kinase SYK	KSYK_HUMAN
	3.367	0.5611	0.825552	Alpha-1-antitrypsin	A1AT_HUMAN
	3.992	0.499	0.82216	Histamine N-methyltransferase	P50135
	3.757	0.4174	0.814298	Mast/stem cell growth factor receptor	KIT_HUMAN
**NSC63551**	5.436	0.7765	3.60171	Gastrotropin	FABP6_HUMAN
	5.131	0.6414	3.26482	Ferrochelatase, mitochondrial	P22830
	3.902	0.9755	2.7743	Nuclear receptor ROR-alpha	RORA_HUMAN
	4.332	0.8664	2.46918	SEC14-like protein 2	O76054
	3.896	0.7793	2.29431	Bile acid receptor	NR1H4_HUMAN
	4.148	0.6913	2.07499	Trafficking protein particle complex subunit 3	O43617
	3.897	0.7794	2.01976	Calmodulin	CALM_HUMAN
	3.949	0.7897	1.96569	Corticosteroid 11-beta-dehydrogenase isozyme 1	DHI1_HUMAN
	3.806	0.6343	1.87628	Glutathione S-transferase P	GSTP1_HUMAN
	3.669	0.7337	1.86833	cAMP-dependent protein kinase catalytic subunit alpha	P00517
	3.891	0.7782	1.80556	Mineralocorticoid receptor	MCR_HUMAN

	4.361	0.623	1.78467	Sulfotransferase family cytosolic 2B member 1	O00204
	3.813	0.6354	1.7724	Histone deacetylase 8	HDAC8_HUMAN
	4.034	0.8068	1.76751	Estradiol 17-beta-dehydrogenase 1	P14061
	3.923	0.6538	1.71	Glutathione S-transferase A1	P08263
	3.736	0.4152	1.70037	Phosphatidylinositol 3-kinase regulatory subunit alpha	P85A_HUMAN
	4.013	0.5733	1.69641	Cytochrome P450 2C8	CP2C8_HUMAN
	4.458	0.4458	1.63648	Vitamin D3 receptor	VDR_HUMAN
	3.857	0.4821	1.60171	Stromelysin-1	MMP3_HUMAN
	3.654	0.7307	1.56665	Alpha-tocopherol transfer protein	P49638
	4.688	0.5209	1.52721	Dual specificity mitogen-activated protein kinase kinase 1	MP2K1_HUMAN
	3.678	0.613	1.42012	Nuclear receptor subfamily 1 group I member 3	NR1I3_HUMAN
	3.864	0.6439	1.39892	Adenosine kinase	P55263
	3.675	0.6125	1.34285	Retinol-binding protein 4	RET4_HUMAN
	3.574	0.5957	1.32464	Receptor tyrosine-protein kinase erbB-4	ERBB4_HUMAN
	3.669	0.5242	1.31682	Interleukin-2	IL2_HUMAN
	4.042	0.311	1.26288	Endothelial protein C receptor	EPCR_HUMAN
	3.713	0.6189	1.25438	Tyrosine-protein kinase SYK	KSYK_HUMAN
	3.871	0.4839	1.25358	Phospholipase A2, membrane associated	P14555
	3.587	0.7174	1.24446	Tyrosine-protein phosphatase non-receptor type 11	PTN11_HUMAN
	3.955	0.4944	1.23939	Retinoic acid receptor gamma	RARG_HUMAN
	3.624	0.6041	1.23049	Oxysterols receptor LXR-beta	NR1H2_HUMAN
	3.573	0.5104	1.19972	72 kDa type IV collagenase	MMP2_HUMAN

	3.597	0.8991	1.1992	Vitamin D-binding protein	VTDB_HUMAN
	3.59	0.5983	1.19139	Ephrin type-B receptor 4	EPHB4_HUMAN
	3.712	0.464	1.18011	Proto-oncogene tyrosine-protein kinase ABL1	P00519
	3.855	0.4283	1.15846	Mast/stem cell growth factor receptor	KIT_HUMAN
	4.411	0.4411	1.09084	Retinoic acid receptor RXR-beta	RXRB_HUMAN
	3.339	0.8347	1.08709	Progesterone receptor	PRGR_HUMAN
	3.689	0.6148	1.07659	Glutathione-requiring prostaglandin D synthase	PTGD2_HUMAN
	3.877	0.5538	1.06993	Mitogen-activated protein kinase 10	MK10_HUMAN
	3.435	0.4294	1.04332	Heat shock protein HSP 90-beta	P08238
	3.764	0.6273	1.0381	Alcohol dehydrogenase class-3	ADHX_HUMAN
	3.428	0.5713	1.03701	Flavin reductase	P30043
	3.934	0.562	0.961004	Fatty acid-binding protein, brain	FABP7_HUMAN
	3.153	0.6306	0.956741	Vascular endothelial growth factor receptor 2	P35968
	4.016	0.4463	0.936628	Retinoic acid receptor beta	RARB_HUMAN
	3.299	0.6599	0.929918	Aldo-keto reductase family 1 member C1	Q04828
	4.003	0.5003	0.913873	Glucocorticoid receptor	GCR_HUMAN
	3.775	0.5394	0.873458	Fatty acid-binding protein, heart	FABPH_HUMAN
	3.216	0.6431	0.859073	Steroid hormone receptor ERR1	P11474
	3.566	0.5944	0.848125	E3 ubiquitin-protein ligase Mdm2	MDM2_HUMAN
	3.661	0.523	0.805448	Hepatocyte nuclear factor 4-gamma	Q14541
**Obtusifoliol**	3.972	0.9929	2.62787	Nuclear receptor ROR-alpha	RORA_HUMAN
	4.594	0.9188	2.5183	Estradiol 17-beta-dehydrogenase 1	P14061

	4.412	0.8823	2.31528	SEC14-like protein 2	O76054
	4.184	0.5977	2.14142	Renin	RENI_HUMAN
	3.893	0.7786	1.99556	Bile acid receptor	NR1H4_HUMAN
	4.277	0.611	1.94212	Lanosterol synthase	ERG7_HUMAN
	4.294	0.7157	1.91753	Glutathione-requiring prostaglandin D synthase	PTGD2_HUMAN
	3.739	0.9348	1.89042	Progesterone receptor	PRGR_HUMAN
	3.71	0.9276	1.88444	Mineralocorticoid receptor	MCR_HUMAN
	3.695	0.6159	1.77162	Ganglioside GM2 activator	P17900
	3.913	0.6521	1.72348	NAD(P)H dehydrogenase [quinone] 1	NQO1_HUMAN
	3.596	0.7191	1.67914	Alcohol dehydrogenase 1C	P00326
	4.345	0.5432	1.63216	Retinoic acid receptor gamma	RARG_HUMAN
	4.109	0.5871	1.62172	Cytochrome P450 2C8	CP2C8_HUMAN
	3.673	0.7347	1.61881	Dipeptidyl peptidase 4	DPP4_HUMAN
	3.908	0.7817	1.5999	Corticosteroid 11-beta-dehydrogenase isozyme 1	DHI1_HUMAN
	4.655	0.5819	1.55038	Fatty acid-binding protein, heart	FABPH_HUMAN
	4.006	0.6677	1.54501	Trafficking protein particle complex subunit 3	O43617
	3.867	0.5525	1.5402	72 kDa type IV collagenase	MMP2_HUMAN
	3.723	0.7445	1.52077	Androgen receptor	ANDR_HUMAN
	4.341	0.6202	1.51462	Sulfotransferase family cytosolic 2B member 1	O00204
	3.72	0.62	1.50842	Flavin reductase	P30043
	4.855	0.5394	1.49867	Dual specificity mitogen-activated protein kinase kinase 1	MP2K1_HUMAN
	3.773	0.7547	1.45696	Calmodulin	CALM_HUMAN
	3.542	0.8855	1.43208	Collagenase 3	MMP13_H

					UMAN
	5.081	0.6351	1.43099	Retinol-binding protein 4	RET4_HUMAN
	3.893	0.6488	1.39653	Glutathione S-transferase A1	P08263
	4.265	0.3281	1.39396	Endothelial protein C receptor	EPCR_HUMAN
	4.201	0.6002	1.38946	Mitogen-activated protein kinase 10	MK10_HUMAN
	4.247	0.7078	1.34136	Sex hormone-binding globulin	SHBG_HUMAN
	3.645	0.6075	1.33217	Oxysterols receptor LXR-beta	NR1H2_HUMAN
	4.092	0.5115	1.32201	Ferrochelatase, mitochondrial	P22830
	3.516	0.7031	1.30997	Histone deacetylase 8	Q9BY41
	4.416	0.4907	1.28631	Retinoic acid receptor beta	RARB_HUMAN
	3.886	0.4858	1.28396	Proto-oncogene tyrosine-protein kinase ABL1	P00519
	3.702	0.6171	1.28347	Oxysterols receptor LXR-alpha	NR1H3_HUMAN
	3.604	0.6007	1.28125	Poly [ADP-ribose] polymerase 1	P09874
	3.747	0.5353	1.25532	Interleukin-2	IL2_HUMAN
	3.612	0.7225	1.25156	Alpha-tocopherol transfer protein	P49638
	3.628	0.4535	1.2191	Vitamin D3 receptor	VDR_HUMAN
	3.452	0.6903	1.21899	Glutathione S-transferase P	GSTP1_HUMAN
	4.021	0.5745	1.18766	Estrogen sulfotransferase	ST1E1_HUMAN
	3.64	0.7281	1.18033	Prothrombin	THRB_HUMAN
	3.353	0.8382	1.17235	Bile salt sulfotransferase	Q06520
	3.594	0.5991	1.16369	Peroxisome proliferator-activated receptor alpha	PPARA_HUMAN
	3.636	0.7271	1.1214	Tyrosine-protein phosphatase non-receptor type 11	PTN11_HUMAN
	4.164	0.5948	1.11138	Fatty acid-binding protein, brain	FABP7_HUMAN
	3.391	0.8478	1.09738	Mitogen-activated protein kinase 14	Q16539

	4.184	0.523	1.08325	Histamine N-methyltransferase	P50135
	3.432	0.6864	1.0629	cAMP-dependent protein kinase catalytic subunit alpha	P00517
	3.829	0.4254	1.05615	Cathepsin B	CATB_HUMAN
	4.007	0.5724	1.05564	Gastrotropin	FABP6_HUMAN
	3.809	0.6348	1.05438	Adenosine kinase	P55263
	3.44	0.688	1.05412	Aldo-keto reductase family 1 member C1	Q04828
	3.805	0.4228	0.995466	Serine/threonine-protein phosphatase PP1-gamma catalytic subunit	P36873
	4.186	0.3806	0.991787	Phosphatidylcholine transfer protein	Q9UKL6
	3.52	0.5029	0.9355	Serine/threonine-protein kinase 6	O14965
	3.556	0.508	0.919515	Neutrophil gelatinase-associated lipocalin	NGAL_HUMAN
	3.703	0.6172	0.91503	E3 ubiquitin-protein ligase Mdm2	MDM2_HUMAN
	3.607	0.6011	0.899894	Tyrosine-protein kinase ITK/TSK	Q08881
	3.543	0.5904	0.889269	Tyrosine-protein kinase JAK3	JAK3_HUMAN
	3.524	0.5034	0.883855	Macrophage metalloelastase	MMP12_HUMAN
	3.544	0.8861	0.828745	Vitamin D-binding protein	VTDB_HUMAN
	4.348	0.4348	0.823816	Retinoic acid receptor RXR-beta	RXRB_HUMAN
	3.834	0.4792	0.805951	Peptidyl-prolyl cis-trans isomerase FKBP1A	FKB1A_HUMAN
	3.454	0.3838	0.803381	Phosphatidylinositol 3-kinase regulatory subunit alpha	P85A_HUMAN
**poriferast-5-en-3beta-ol**	4.634	0.9268	2.66419	Estradiol 17-beta-dehydrogenase 1	P14061
	3.928	0.982	2.55949	Nuclear receptor ROR-alpha	RORA_HUMAN
	4.486	0.8971	2.52015	SEC14-like protein 2	O76054
	4.331	0.7219	2.19431	Trafficking protein particle complex subunit 3	O43617

	3.821	0.9553	2.13142	Progesterone receptor	PRGR_HUMAN
	3.738	0.9344	1.95901	Mineralocorticoid receptor	MCR_HUMAN
	3.856	0.7711	1.94294	Bile acid receptor	NR1H4_HUMAN
	4.251	0.5314	1.88147	Caspase-3	CASP3_HUMAN
	3.767	0.7534	1.86062	Cell division protein kinase 2	P24941
	4.344	0.6206	1.838	Hepatocyte nuclear factor 4-gamma	Q14541
	4.495	0.6421	1.7927	Sulfotransferase family cytosolic 2B member 1	O00204
	3.964	0.7928	1.75502	Corticosteroid 11-beta-dehydrogenase isozyme 1	DHI1_HUMAN
	4.29	0.5363	1.73331	Retinoic acid receptor alpha	RARA_HUMAN
	4.447	0.7411	1.66958	Sex hormone-binding globulin	SHBG_HUMAN
	3.851	0.6419	1.63641	Oxysterols receptor LXR-alpha	NR1H3_HUMAN
	3.709	0.7418	1.63463	Aldo-keto reductase family 1 member C1	Q04828
	3.806	0.7612	1.61372	Prothrombin	THRB_HUMAN
	5.19	0.6487	1.60679	Retinol-binding protein 4	RET4_HUMAN
	3.634	0.7269	1.592	Steroid hormone receptor ERR1	P11474
	4.239	0.5299	1.51214	Retinoic acid receptor gamma	RARG_HUMAN
	4.022	0.5746	1.50164	Cytochrome P450 2C8	CP2C8_HUMAN
	4.176	0.522	1.48988	Ferrochelatase, mitochondrial	P22830
	3.813	0.9532	1.48792	Vitamin D-binding protein	VTDB_HUMAN
	3.747	0.4163	1.48603	Phosphatidylinositol 3-kinase regulatory subunit alpha	P85A_HUMAN
	3.964	0.6606	1.45773	E3 ubiquitin-protein ligase Mdm2	MDM2_HUMAN
	4.408	0.551	1.44548	Fatty acid-binding protein, brain	FABP7_HUMAN

	3.888	0.648	1.41596	Glutathione S-transferase A1	P08263
	4.024	0.4472	1.39511	Growth factor receptor-bound protein 2	GRB2_HUMAN
	3.676	0.4594	1.36618	Heat shock protein HSP 90-beta	P08238
	4.262	0.4735	1.35583	Angiopoietin-1 receptor	TIE2_HUMAN
	3.64	0.6066	1.33438	Flavin reductase	P30043
	3.852	0.642	1.32934	Tyrosine-protein kinase SYK	KSYK_HUMAN
	4.365	0.4365	1.3199	Vitamin D3 receptor	VDR_HUMAN
	3.488	0.6977	1.31166	Glutathione S-transferase P	GSTP1_HUMAN
	3.695	0.739	1.30117	Calmodulin	CALM_HUMAN
	4.682	0.4682	1.25793	Retinoic acid receptor RXR-beta	RXRB_HUMAN
	3.49	0.6981	1.24682	Histone deacetylase 8	Q9BY41
	3.695	0.6159	1.20798	Ephrin type-B receptor 4	EPHB4_HUMAN
	3.872	0.6454	1.20163	Adenosine kinase	P55263
	3.665	0.733	1.20146	Tyrosine-protein phosphatase non-receptor type 11	PTN11_HUMAN
	3.411	0.5685	1.19271	cGMP-specific 3,5-cyclic phosphodiesterase	PDE5A_HUMAN
	4.559	0.5066	1.1925	Dual specificity mitogen-activated protein kinase kinase 1	MP2K1_HUMAN
	3.571	0.5951	1.17807	Tyrosine-protein phosphatase non-receptor type 1	PTN1_HUMAN
	3.61	0.6017	1.16035	NAD(P)H dehydrogenase [quinone] 1	NQO1_HUMAN
	3.867	0.4297	1.14125	Serine/threonine-protein phosphatase PP1-gamma catalytic subunit	P36873
	3.444	0.6888	1.13978	Dipeptidyl peptidase 4	DPP4_HUMAN
	3.824	0.6373	1.13692	Glutathione-requiring prostaglandin D synthase	PTGD2_HUMAN
	4.024	0.5749	1.11152	Gastrotropin	FABP6_HUMAN
	3.62	0.5172	1.10902	72 kDa type IV collagenase	MMP2_HU

					MAN
	3.771	0.4713	1.10317	Proto-oncogene tyrosine-protein kinase ABL1	P00519
	3.757	0.6262	1.08505	[Pyruvate dehydrogenase [lipoamide]] kinase isozyme 2, mitochondrial	PDK2_HUMAN
	3.686	0.6143	1.0832	Tyrosine-protein kinase ITK/TSK	Q08881
	3.639	0.6065	1.05309	Oxysterols receptor LXR-beta	NR1H2_HUMAN
	3.679	0.6132	1.04731	Proto-oncogene tyrosine-protein kinase LCK	LCK_HUMAN
	3.55	0.5072	1.04655	Protein S100-A9	S10A9_HUMAN
	3.302	0.8254	1.04604	Bile salt sulfotransferase	Q06520
	3.538	0.5897	1.04238	Peroxisome proliferator-activated receptor alpha	PPARA_HUMAN
	3.851	0.6418	1.00654	Alcohol dehydrogenase class-3	ADHX_HUMAN
	5.686	0.5686	0.99712	Cellular retinoic acid-binding protein 2	P29373
	3.971	0.3054	0.957404	Endothelial protein C receptor	EPCR_HUMAN
	4.042	0.5052	0.945873	Basic fibroblast growth factor receptor 1	FGFR1_HUMAN
	4.049	0.5061	0.91633	Histamine N-methyltransferase	P50135
	3.627	0.5182	0.894068	Tyrosine-protein kinase ZAP-70	ZAP70_HUMAN
	3.384	0.5639	0.868562	Alpha-1-antitrypsin	A1AT_HUMAN
	3.578	0.5111	0.865114	Protein farnesyltransferase/geranylgeranyltransferase type-1 subunit alpha	FNTA_HUMAN
	3.53	0.5043	0.851831	Interleukin-2	IL2_HUMAN
	3.587	0.5125	0.813116	Insulin-like growth factor IA	P01343
**sitosterol**	5.121	0.6401	3.20438	Ferrochelatase, mitochondrial	P22830
	4.427	0.6325	2.72337	Insulin-like growth factor IA	P01343
	4.381	0.8763	2.52749	SEC14-like protein 2	O76054
	4.211	0.5264	2.22582	Interleukin-2	IL2_HUMAN

	3.687	0.9218	2.16304	Nuclear receptor ROR-alpha	RORA_HUMAN
	3.919	0.7838	2.13568	Prothrombin	THRB_HUMAN
	4.17	0.695	2.08289	Trafficking protein particle complex subunit 3	O43617
	4.249	0.7081	2.07326	Glutathione-requiring prostaglandin D synthase	PTGD2_HUMAN
	3.813	0.7626	2.05484	Bile acid receptor	NR1H4_HUMAN
	3.861	0.6434	2.0494	Alpha-1-antitrypsin	A1AT_HUMAN
	4.49	0.6415	2.02	Gastrotropin	FABP6_HUMAN
	3.673	0.9183	1.96802	Progesterone receptor	PRGR_HUMAN
	4.48	0.6399	1.93919	Sulfotransferase family cytosolic 2B member 1	O00204
	4.124	0.8248	1.907	Estradiol 17-beta-dehydrogenase 1	P14061
	3.843	0.6405	1.90688	Receptor tyrosine-protein kinase erbB-4	ERBB4_HUMAN
	3.909	0.7818	1.83933	Corticosteroid 11-beta-dehydrogenase isozyme 1	DHI1_HUMAN
	3.888	0.7775	1.76958	Mineralocorticoid receptor	MCR_HUMAN
	4.048	0.5783	1.75316	Lanosterol synthase	ERG7_HUMAN
	4.24	0.53	1.679	Retinoic acid receptor gamma	RARG_HUMAN
	5.116	0.6396	1.66911	Retinol-binding protein 4	RET4_HUMAN
	3.704	0.4116	1.58897	Phosphatidylinositol 3-kinase regulatory subunit alpha	P85A_HUMAN
	3.729	0.7459	1.58238	Calmodulin	CALM_HUMAN
	3.561	0.7121	1.5703	Dipeptidyl peptidase 4	DPP4_HUMAN
	4.09	0.5112	1.55896	Retinoic acid receptor alpha	RARA_HUMAN
	3.763	0.5375	1.52807	Basic fibroblast growth factor receptor 1	FGFR1_HUMAN

	3.929	0.5612	1.50408	Cytochrome P450 2C8	CP2C8_HUMAN
	4.66	0.5178	1.46249	Dual specificity mitogen-activated protein kinase kinase 1	MP2K1_HUMAN
	4.029	0.5755	1.45301	Hepatocyte nuclear factor 4-gamma	Q14541
	3.633	0.519	1.43679	Protein S100-A9	S10A9_HUMAN
	3.676	0.7352	1.42554	Tyrosine-protein phosphatase non-receptor type 11	PTN11_HUMAN
	3.655	0.5222	1.33179	72 kDa type IV collagenase	MMP2_HUMAN
	3.63	0.605	1.33008	Oxysterols receptor LXR-alpha	NR1H3_HUMAN
	3.664	0.458	1.25324	Vitamin D3 receptor	VDR_HUMAN
	3.694	0.6157	1.25218	Proto-oncogene tyrosine-protein kinase LCK	LCK_HUMAN
	3.515	0.5858	1.22715	Flavin reductase	P30043
	3.709	0.5298	1.22255	Tyrosine-protein kinase ZAP-70	ZAP70_HUMAN
	3.842	0.4269	1.2177	Growth factor receptor-bound protein 2	GRB2_HUMAN
	3.715	0.5307	1.21268	S-methyl-5-thioadenosine phosphorylase	Q13126
	4.016	0.3089	1.1902	Endothelial protein C receptor	EPCR_HUMAN
	3.957	0.5654	1.18183	Mitogen-activated protein kinase 10	MK10_HUMAN
	4.5	0.45	1.17529	Retinoic acid receptor RXR-beta	RXRB_HUMAN
	3.599	0.8998	1.17168	Vitamin D-binding protein	VTDB_HUMAN
	3.416	0.6833	1.16852	Aldo-keto reductase family 1 member C1	Q04828
	3.425	0.685	1.1605	Serine/threonine-protein kinase Chk1	CHK1_HUMAN
	3.632	0.6053	1.15061	Tyrosine-protein kinase ITK/TSK	Q08881
	3.656	0.6093	1.12014	Glutathione S-transferase A1	P08263
	3.555	0.5925	1.0495	Oxysterols receptor LXR-beta	NR1H2_HU

					MAN
	3.319	0.6638	0.997923	Histone deacetylase 8	Q9BY41
	3.673	0.6121	0.97914	Adenosine kinase	P55263
	3.53	0.5043	0.959589	Peptidyl-prolyl cis-trans isomerase FKBP1A	FKB1A_HUMAN
	3.406	0.4258	0.95368	Heat shock protein HSP 90-beta	P08238
	5.462	0.5462	0.941554	Cellular retinoic acid-binding protein 2	P29373
	3.739	0.4154	0.936788	Mast/stem cell growth factor receptor	KIT_HUMAN
	3.266	0.6533	0.921289	Glutathione S-transferase P	GSTP1_HUMAN
	3.907	0.5581	0.890712	Fatty acid-binding protein, brain	FABP7_HUMAN
	3.362	0.6725	0.88766	Alpha-tocopherol transfer protein	P49638
	3.968	0.4409	0.844198	Retinoic acid receptor beta	RARB_HUMAN
	3.215	0.643	0.835375	Steroid hormone receptor ERR1	P11474
**Sitosterol alpha1**	5.255	0.6568	3.8521	Caspase-3	CASP3_HUMAN
	4.461	0.8923	2.51297	SEC14-like protein 2	O76054
	3.88	0.97	2.4967	Nuclear receptor ROR-alpha	RORA_HUMAN
	4.482	0.8964	2.42226	Estradiol 17-beta-dehydrogenase 1	P14061
	3.959	0.7917	2.23976	Bile acid receptor	NR1H4_HUMAN
	3.996	0.666	2.01424	Oxysterols receptor LXR-alpha	NR1H3_HUMAN
	4.238	0.6054	1.96241	Lanosterol synthase	ERG7_HUMAN
	4.172	0.6953	1.93613	Trafficking protein particle complex subunit 3	O43617
	3.91	0.782	1.93504	Prothrombin	THRB_HUMAN
	3.639	0.7279	1.87016	Alcohol dehydrogenase 1C	P00326
	3.688	0.922	1.84446	Progesterone receptor	PRGR_HUMAN
	4.293	0.477	1.81927	Retinoic acid receptor gamma	RARG_HUMAN
	3.609	0.9023	1.7445	Mineralocorticoid receptor	MCR_HUM

					AN
	3.631	0.6052	1.69563	Ganglioside GM2 activator	P17900
	4.405	0.6293	1.68923	Sulfotransferase family cytosolic 2B member 1	O00204
	3.78	0.63	1.68624	Oxysterols receptor LXR-beta	NR1H2_HUMAN
	3.627	0.9067	1.68152	Collagenase 3	MMP13_HUMAN
	3.895	0.779	1.65578	Corticosteroid 11-beta-dehydrogenase isozyme 1	DHI1_HUMAN
	4.376	0.7294	1.59812	Sex hormone-binding globulin	SHBG_HUMAN
	5.133	0.6416	1.56585	Retinol-binding protein 4	RET4_HUMAN
	4.013	0.5733	1.52759	Cytochrome P450 2C8	CP2C8_HUMAN
	3.9	0.6501	1.48761	Glutathione S-transferase A1	P08263
	4.129	0.5161	1.45101	Ferrochelatase, mitochondrial	P22830
	3.767	0.6279	1.40147	Ephrin type-B receptor 4	EPHB4_HUMAN
	4.692	0.5213	1.38177	Dual specificity mitogen-activated protein kinase kinase 1	MP2K1_HUMAN
	3.67	0.7341	1.2997	Calmodulin	CALM_HUMAN
	3.632	0.4035	1.2748	Phosphatidylinositol 3-kinase regulatory subunit alpha	P85A_HUMAN
	4.312	0.392	1.27077	Serine/threonine-protein phosphatase PP1-gamma catalytic subunit	P36873
	4.092	0.5845	1.25402	Gastrotropin	FABP6_HUMAN
	3.594	0.5991	1.21135	Receptor tyrosine-protein kinase erbB-4	ERBB4_HUMAN
	3.645	0.5208	1.20044	72 kDa type IV collagenase	MMP2_HUMAN
	3.639	0.7278	1.19798	Tyrosine-protein phosphatase non-receptor type 11	PTN11_HUMAN
	3.845	0.6409	1.19509	Adenosine kinase	P55263
	3.968	0.5668	1.17009	Estrogen sulfotransferase	ST1E1_HUMAN
	3.475	0.695	1.1607	Vascular endothelial growth	P35968

				factor receptor 2	
	3.66	0.5229	1.15217	Interleukin-2	IL2_HUMAN
	3.483	0.4353	1.13477	Vitamin D3 receptor	VDR_HUMAN
	4.056	0.312	1.13408	Endothelial protein C receptor	EPCR_HUMAN
	3.583	0.5118	1.11358	Tyrosine-protein phosphatase non-receptor type 1	PTN1_HUMAN
	3.776	0.6293	1.0944	Glutathione-requiring prostaglandin D synthase	PTGD2_HUMAN
	3.872	0.6453	1.08942	Alcohol dehydrogenase class-3	ADHX_HUMAN
	4.19	0.3809	1.06769	Phosphatidylcholine transfer protein	Q9UKL6
	3.678	0.5254	1.04027	Tyrosine-protein kinase ZAP-70	ZAP70_HUMAN
	3.486	0.581	1.01999	Flavin reductase	P30043
	3.669	0.4586	0.963685	Proto-oncogene tyrosine-protein kinase ABL1	P00519
	3.373	0.6746	0.961981	Aldo-keto reductase family 1 member C1	Q04828
	4.399	0.4399	0.94639	Retinoic acid receptor RXR-beta	RXRB_HUMAN
	3.893	0.4866	0.946157	Phospholipase A2, membrane associated	P14555
	4.112	0.4569	0.942471	Retinoic acid receptor beta	RARB_HUMAN
	5.542	0.5542	0.909472	Cellular retinoic acid-binding protein 2	P29373
	3.507	0.5845	0.870473	Tyrosine-protein kinase JAK3	JAK3_HUMAN
	3.646	0.5209	0.863476	Protein kinase C theta type	KPCT_HUMAN
	3.291	0.6583	0.863127	Glutathione S-transferase P	GSTP1_HUMAN
	3.409	0.4261	0.848344	Heat shock protein HSP 90-beta	P08238
	3.835	0.5478	0.835931	Fatty acid-binding protein, heart	FABPH_HUMAN
	3.547	0.5911	0.831621	Proto-oncogene tyrosine-protein kinase LCK	LCK_HUMAN

**Stigmasterol**	4.632	0.9263	2.84641	Estradiol 17-beta-dehydrogenase 1	P14061
	3.938	0.9844	2.77644	Nuclear receptor ROR-alpha	RORA_HUMAN
	4.324	0.8647	2.37981	SEC14-like protein 2	O76054
	3.821	0.9552	2.31655	Progesterone receptor	PRGR_HUMAN
	3.805	0.7611	2.24111	Histone deacetylase 8	Q9BY41
	3.783	0.9457	2.19264	Mineralocorticoid receptor	MCR_HUMAN
	3.888	0.7775	2.189	Bile acid receptor	NR1H4_HUMAN
	3.903	0.7806	1.94992	Calmodulin	CALM_HUMAN
	4.499	0.6428	1.94304	Sulfotransferase family cytosolic 2B member 1	O00204
	4.108	0.6846	1.92572	Trafficking protein particle complex subunit 3	O43617
	4.707	0.3621	1.89176	Serine/threonine-protein phosphatase PP1-gamma catalytic subunit	P36873
	5.26	0.6575	1.81388	Retinol-binding protein 4	RET4_HUMAN
	4.449	0.7414	1.808	Sex hormone-binding globulin	SHBG_HUMAN
	4.092	0.6821	1.75273	Glutathione-requiring prostaglandin D synthase	PTGD2_HUMAN
	4.561	0.4561	1.73209	Vitamin D3 receptor	VDR_HUMAN
	3.781	0.4201	1.72564	Phosphatidylinositol 3-kinase regulatory subunit alpha	P85A_HUMAN
	3.856	0.7713	1.67532	Corticosteroid 11-beta-dehydrogenase isozyme 1	DHI1_HUMAN
	4.239	0.5299	1.64811	Retinoic acid receptor gamma	RARG_HUMAN
	4.181	0.5226	1.6309	Ferrochelatase, mitochondrial	P22830
	3.908	0.6513	1.60464	Glutathione S-transferase A1	P08263
	4.021	0.5027	1.58771	Caspase-3	CASP3_HUMAN
	3.776	0.6293	1.56502	Nuclear receptor subfamily 1 group I member 3	NR1I3_HUMAN

	3.745	0.6241	1.54939	Oxysterols receptor LXR-alpha	NR1H3_HUMAN
	3.926	0.6543	1.45402	Adenosine kinase	P55263
	3.662	0.6103	1.44808	Receptor tyrosine-protein kinase erbB-4	ERBB4_HUMAN
	4.664	0.5182	1.44763	Dual specificity mitogen-activated protein kinase kinase 1	MP2K1_HUMAN
	3.911	0.5587	1.43229	Cytochrome P450 2C8	CP2C8_HUMAN
	3.459	0.8647	1.37873	Collagenase 3	MMP13_HUMAN
	4.111	0.5874	1.37544	Gastrotropin	FABP6_HUMAN
	3.967	0.4959	1.30422	Retinoic acid receptor alpha	RARA_HUMAN
	3.654	0.6089	1.2541	Ephrin type-B receptor 4	EPHB4_HUMAN
	3.439	0.6878	1.24944	Dipeptidyl peptidase 4	DPP4_HUMAN
	3.302	0.8254	1.1623	Bile salt sulfotransferase	Q06520
	4.087	0.5838	1.1543	Fatty acid-binding protein, brain	FABP7_HUMAN
	3.583	0.7165	1.15355	Tyrosine-protein phosphatase non-receptor type 11	PTN11_HUMAN
	3.857	0.6428	1.15061	Alcohol dehydrogenase class-3	ADHX_HUMAN
	3.721	0.4651	1.13261	Proto-oncogene tyrosine-protein kinase ABL1	P00519
	3.537	0.5053	1.06557	72 kDa type IV collagenase	MMP2_HUMAN
	3.574	0.5956	1.0443	Oxysterols receptor LXR-beta	NR1H2_HUMAN
	5.602	0.5602	1.03363	Cellular retinoic acid-binding protein 2	P29373
	3.477	0.5795	1.01206	NAD(P)H dehydrogenase [quinone] 1	NQO1_HUMAN
	3.554	0.8885	1.00516	Vitamin D-binding protein	VTDB_HUMAN
	3.546	0.5066	0.999333	Interleukin-2	IL2_HUMAN
	3.876	0.5537	0.998069	Fatty acid-binding protein, heart	FABPH_HU

					MAN
	3.666	0.611	0.986386	E3 ubiquitin-protein ligase Mdm2	MDM2_HUMAN
	3.662	0.5231	0.982385	Lanosterol synthase	ERG7_HUMAN
	3.271	0.6541	0.90964	Steroid hormone receptor ERR1	P11474
	3.867	0.2975	0.904763	Endothelial protein C receptor	EPCR_HUMAN
	3.345	0.5574	0.896149	Alpha-1-antitrypsin	A1AT_HUMAN
	3.948	0.564	0.888853	Lactoylglutathione lyase	LGUL_HUMAN
	3.731	0.533	0.871034	Hepatocyte nuclear factor 4-gamma	Q14541
	3.307	0.8268	0.856349	Estrogen receptor	ESR1_HUMAN
	3.711	0.4123	0.85467	Mast/stem cell growth factor receptor	KIT_HUMAN
	3.264	0.6527	0.853726	Glutathione S-transferase P	GSTP1_HUMAN
	3.241	0.6481	0.845931	Hepatocyte growth factor receptor	P08581
	3.362	0.6725	0.837723	Alpha-tocopherol transfer protein	P49638
	3.459	0.5765	0.834082	Tyrosine-protein kinase JAK3	JAK3_HUMAN
	3.979	0.4421	0.832431	Retinoic acid receptor beta	RARB_HUMAN
	3.534	0.5049	0.825941	Insulin-like growth factor IA	P01343
	3.661	0.4067	0.809102	Growth factor receptor-bound protein 2	GRB2_HUMAN
**Styrone**	2.964	0.5928	1.73486	Tyrosyl-tRNA synthetase, cytoplasmic	P54577
	2.871	0.9569	1.51599	Peptidyl-prolyl cis-trans isomerase A	P62937
	2.949	0.5898	1.45586	Mitogen-activated protein kinase 14	Q16539
	2.851	0.9504	1.38383	Carbonic anhydrase 2	CAH2_HUMAN
	2.867	0.7168	1.21795	Peroxisome proliferator-activated receptor gamma	PPARG_HUMAN
	2.819	0.5638	1.14256	Phenylethanolamine N-	PNMT_HU

				methyltransferase	MAN
	2.825	0.7063	1.03529	Caspase-7	CASP7_HUMAN
	2.816	0.5631	0.947914	Estrogen-related receptor gamma	ERR3_HUMAN
	2.916	0.486	0.938374	cAMP-specific 3,5-cyclic phosphodiesterase 4B	PDE4B_HUMAN
	2.901	0.5803	0.931302	Tyrosine-protein phosphatase non-receptor type 1	PTN1_HUMAN
	2.952	0.3691	0.886157	Hepatocyte growth factor receptor	P08581
	2.973	0.2973	0.834793	SEC14-like protein 2	O76054
**TAU**	3.591	0.7182	3.35928	Ornithine carbamoyltransferase, mitochondrial	P00480
	3.565	0.4456	2.42422	Glucose-6-phosphate isomerase	G6PI_HUMAN
	3.492	0.3492	1.83432	Nucleoside diphosphate kinase B	NDKB_HUMAN
	3.376	0.3751	1.62613	Non-secretory ribonuclease	P10153
	2.856	0.5712	1.18522	Cytidine deaminase	CDD_HUMAN
	3.338	0.4172	1.03044	Farnesyl pyrophosphate synthetase	FPPS_HUMAN
	2.907	0.3633	0.991099	Tryptase beta-2	TRYB2_HUMAN
**Tetrahydroalstonine**	4.674	0.779	3.41078	Tyrosine-protein kinase JAK3	JAK3_HUMAN
	4.531	0.7552	2.95243	Tyrosine-protein kinase ITK/TSK	Q08881
	4.614	0.769	2.9428	Proto-oncogene tyrosine-protein kinase LCK	LCK_HUMAN
	4.645	0.6635	2.93531	MAP kinase-activated protein kinase 2	P49137
	4.97	0.497	2.80511	Retinoic acid receptor gamma	RARG_HUMAN
	3.887	0.9718	2.51831	Nuclear receptor ROR-alpha	RORA_HUMAN
	4.44	0.6343	2.49331	Tyrosine-protein kinase ZAP-70	ZAP70_HUMAN
	3.924	0.7848	2.43113	Histone deacetylase 8	Q9BY41
	4.641	0.663	2.3769	Glycogen synthase kinase-3 beta	GSK3B_HU

					MAN
	3.814	0.9535	2.32028	Kinesin-like protein KIF11	KIF11_HUMAN
	4.421	0.6316	2.23013	Protein kinase C theta type	KPCT_HUMAN
	3.784	0.7569	2.21557	Coagulation factor X	FA10_HUMAN
	4.21	0.7016	2.15074	Hepatocyte growth factor receptor	MET_HUMAN
	3.85	0.6417	2.11458	Insulin-like growth factor IA	P01343
	4.103	0.5861	2.09965	Protein farnesyltransferase/geranylgeranyltransferase type-1 subunit alpha	FNTA_HUMAN
	3.911	0.7822	2.02202	Sex hormone-binding globulin	SHBG_HUMAN
	3.844	0.7688	1.99036	cAMP-specific 3,5-cyclic phosphodiesterase 4D	PDE4D_HUMAN
	3.978	0.7956	1.9753	Tyrosine-protein phosphatase non-receptor type 11	PTN11_HUMAN
	3.677	0.7354	1.94033	Prothrombin	THRB_HUMAN
	3.759	0.3759	1.92614	Tryptase beta-2	TRYB2_HUMAN
	3.962	0.566	1.8384	Macrophage metalloelastase	MMP12_HUMAN
	3.952	0.7904	1.82477	Wiskott-Aldrich syndrome protein	WASP_HUMAN
	3.647	0.7294	1.77299	Glutathione-requiring prostaglandin D synthase	PTGD2_HUMAN
	3.622	0.9054	1.63459	Progesterone receptor	PRGR_HUMAN
	3.666	0.7332	1.60761	Aldo-keto reductase family 1 member C1	Q04828
	3.863	0.5519	1.59632	Basic fibroblast growth factor receptor 1	FGFR1_HUMAN
	3.957	0.5653	1.58555	S-methyl-5-thioadenosine phosphorylase	Q13126
	3.557	0.8891	1.57389	Estrogen-related receptor gamma	P62508
	3.883	0.4853	1.55481	Betaine--homocysteine S-methyltransferase 1	Q93088
	3.486	0.8714	1.51128	Mitogen-activated protein kinase	Q16539

				14	
	3.582	0.7164	1.51061	Cell division protein kinase 2	P24941
	3.679	0.7358	1.46036	Estradiol 17-beta-dehydrogenase 1	P14061
	3.493	0.8733	1.44922	Transthyretin	TTHY_HUMAN
	3.548	0.8871	1.38542	Estrogen receptor	ESR1_HUMAN
	3.765	0.4706	1.36522	Interleukin-2	IL2_HUMAN
	3.748	0.7495	1.34611	Corticosteroid 11-beta-dehydrogenase isozyme 1	DHI1_HUMAN
	3.821	0.6368	1.33439	Glutathione S-transferase A1	P08263
	4.287	0.5359	1.29901	Histamine N-methyltransferase	P50135
	3.916	0.7832	1.27758	Bile salt sulfotransferase	Q06520
	3.393	0.6786	1.2709	Placenta growth factor	PLGF_HUMAN
	3.939	0.5627	1.22705	Thyroid hormone receptor alpha	P10827
	3.988	0.4985	1.22642	Ferrochelatase, mitochondrial	P22830
	3.634	0.7269	1.2078	Serum albumin	ALBU_HUMAN
	3.822	0.546	1.20706	Lanosterol synthase	ERG7_HUMAN
	3.558	0.5083	1.19496	Phospholipase A2, membrane associated	P14555
	3.66	0.915	1.19097	Vitamin D-binding protein	VTDB_HUMAN
	3.543	0.5905	1.1798	Flavin reductase	P30043
	3.812	0.5445	1.17967	Tyrosine-protein kinase JAK2	JAK2_HUMAN
	3.509	0.3898	1.15806	Bcl-2-like protein 1	Q07817
	4.022	0.5746	1.14224	Gastrotropin	FABP6_HUMAN
	3.55	0.4437	1.139	Vitamin D3 receptor	VDR_HUMAN
	3.37	0.5616	1.10238	Ganglioside GM2 activator	P17900
	3.389	0.6778	1.07907	Thyroid hormone receptor beta	P10828
	3.212	0.8029	1.0644	Alcohol dehydrogenase 1B	ADH1B_HUMAN
	3.786	0.4207	1.05492	Leukotriene A-4 hydrolase	LKHA4_HUMAN

	3.697	0.6161	1.05221	Trafficking protein particle complex subunit 3	O43617
	3.844	0.6406	1.04194	Alcohol dehydrogenase class-3	ADHX_HUMAN
	3.957	0.5652	1.01522	Sulfotransferase family cytosolic 2B member 1	O00204
	3.443	0.5738	0.993557	Oxysterols receptor LXR-beta	NR1H2_HUMAN
	3.417	0.5695	0.992296	Stromelysin-1	MMP3_HUMAN
	3.275	0.8188	0.976097	cAMP-specific 3,5-cyclic phosphodiesterase 4B	PDE4B_HUMAN
	4.394	0.6277	0.976064	Aldo-keto reductase family 1 member C2	AK1C2_HUMAN
	3.227	0.8067	0.954514	TGF-beta receptor type-1	TGFR1_HUMAN
	3.305	0.3672	0.949071	Peptidyl-prolyl cis-trans isomerase FKBP1B	FKB1B_HUMAN
	3.113	0.5188	0.930028	T-cell surface glycoprotein CD1a	CD1A_HUMAN
	3.278	0.4097	0.915544	Heme oxygenase 1	HMOX1_HUMAN
	3.649	0.4054	0.87923	Neutrophil gelatinase-associated lipocalin	NGAL_HUMAN
	3.636	0.606	0.859209	E3 ubiquitin-protein ligase Mdm2	MDM2_HUMAN
	3.358	0.5597	0.8517	Leukocyte elastase	ELNE_HUMAN
	3.629	0.5185	0.832818	Cytochrome P450 2C8	CP2C8_HUMAN
	3.263	0.8157	0.827019	ADAM 17	ADA17_HUMAN
	3.417	0.5695	0.819597	Glutathione S-transferase P	GSTP1_HUMAN
	3.735	0.4668	0.810916	Angiopoietin-1 receptor	TIE2_HUMAN
	3.446	0.6892	0.809909	Calmodulin	CALM_HUMAN
**(24R)-4alpha-Methyl-24-ethylcholesta-7,25-dien-3beta-ylacetate**	4.442	0.7403	2.92685	Beta-secretase 1	P56817
	3.941	0.9853	2.5689	Nuclear receptor ROR-alpha	RORA_HUMAN

	4.253	0.8507	2.03324	SEC14-like protein 2	O76054
	3.805	0.7609	1.9437	cAMP-dependent protein kinase catalytic subunit alpha	P00517
	3.985	0.6642	1.91316	Oxysterols receptor LXR-alpha	NR1H3_HUMAN
	4.231	0.7051	1.8706	Adenosine kinase	P55263
	5.231	0.5812	1.8368	Cellular retinoic acid-binding protein 2	P29373
	3.804	0.7609	1.81006	Bile acid receptor	NR1H4_HUMAN
	3.891	0.7781	1.80896	Prothrombin	THRB_HUMAN
	4.402	0.5503	1.80879	Ferrochelatase, mitochondrial	P22830
	4.005	0.5721	1.79922	72 kDa type IV collagenase	MMP2_HUMAN
	3.958	0.6596	1.7924	Nuclear receptor subfamily 1 group I member 3	NR1I3_HUMAN
	4.079	0.6799	1.77314	Glutathione S-transferase A1	P08263
	4.101	0.6834	1.7313	Trafficking protein particle complex subunit 3	O43617
	4.4	0.5501	1.72687	Retinoic acid receptor gamma	RARG_HUMAN
	4.133	0.8265	1.70788	Estradiol 17-beta-dehydrogenase 1	P14061
	3.641	0.6069	1.6638	Ganglioside GM2 activator	P17900
	4.096	0.5119	1.66053	Proto-oncogene tyrosine-protein kinase ABL1	P00519
	3.649	0.7298	1.6604	Histone deacetylase 8	Q9BY41
	4.112	0.5874	1.63974	Cytochrome P450 2C8	CP2C8_HUMAN
	3.803	0.6339	1.60737	Receptor tyrosine-protein kinase erbB-4	ERBB4_HUMAN
	3.833	0.7665	1.60678	Calmodulin	CALM_HUMAN
	4.218	0.5272	1.57533	Retinoic acid receptor alpha	RARA_HUMAN
	3.739	0.6232	1.57039	Flavin reductase	P30043
	4.245	0.6065	1.552	Estrogen sulfotransferase	ST1E1_HUMAN
	4.361	0.623	1.50483	Fatty acid-binding protein, heart	FABPH_HUMAN

	3.972	0.4965	1.50191	Interleukin-2	IL2_HUMAN
	3.814	0.7628	1.45233	Wiskott-Aldrich syndrome protein	WASP_HUMAN
	3.898	0.6497	1.4064	Tyrosine-protein kinase SYK	KSYK_HUMAN
	3.554	0.7108	1.38642	Dipeptidyl peptidase 4	DPP4_HUMAN
	3.879	0.5541	1.38571	Insulin-like growth factor IA	P01343
	4.209	0.6013	1.37265	Gastrotropin	FABP6_HUMAN
	3.584	0.7167	1.37084	Aldo-keto reductase family 1 member C1	Q04828
	4.468	0.4964	1.36491	Retinoic acid receptor beta	RARB_HUMAN
	3.781	0.7561	1.34796	Corticosteroid 11-beta-dehydrogenase isozyme 1	DHI1_HUMAN
	4.217	0.6025	1.34408	Sulfotransferase family cytosolic 2B member 1	O00204
	3.418	0.8545	1.32019	Mineralocorticoid receptor	MCR_HUMAN
	4.698	0.522	1.31963	Dual specificity mitogen-activated protein kinase kinase 1	MP2K1_HUMAN
	3.63	0.7261	1.30436	Alpha-tocopherol transfer protein	P49638
	3.646	0.4557	1.30415	Heat shock protein HSP 90-beta	P08238
	4.281	0.6115	1.30087	Fatty acid-binding protein, brain	FABP7_HUMAN
	3.77	0.4713	1.28629	Vitamin D3 receptor	VDR_HUMAN
	3.701	0.7403	1.28244	Tyrosine-protein phosphatase non-receptor type 11	PTN11_HUMAN
	3.423	0.8558	1.24071	Cell division protein kinase 2	P24941
	3.904	0.7809	1.19286	Bile salt sulfotransferase	Q06520
	3.491	0.6982	1.17877	cAMP-specific 3,5-cyclic phosphodiesterase 4D	PDE4D_HUMAN
	3.617	0.7235	1.14736	Sorbitol dehydrogenase	DHSO_HUMAN
	3.531	0.3923	1.14058	Bcl-2-like protein 1	Q07817
	3.831	0.6385	1.13966	Glutathione-requiring prostaglandin D synthase	PTGD2_HUMAN
	3.412	0.6823	1.129	Steroid hormone receptor ERR1	P11474

	4.258	0.3871	1.10509	Phosphatidylcholine transfer protein	Q9UKL6
	3.652	0.6086	1.0957	Retinol-binding protein 4	RET4_HUMAN
	4.175	0.5219	1.0804	Histamine N-methyltransferase	P50135
	3.875	0.6458	1.03813	Alcohol dehydrogenase class-3	ADHX_HUMAN
	3.6	0.5142	1.03653	Neutrophil gelatinase-associated lipocalin	NGAL_HUMAN
	3.806	0.4229	1.01131	Serine/threonine-protein phosphatase PP1-gamma catalytic subunit	P36873
	3.849	0.5498	0.95095	Hepatocyte nuclear factor 4-gamma	Q14541
	4.451	0.4451	0.9502	Retinoic acid receptor RXR-beta	RXRB_HUMAN
	3.337	0.6674	0.947228	Glutathione S-transferase P	GSTP1_HUMAN
	3.707	0.6178	0.937133	E3 ubiquitin-protein ligase Mdm2	MDM2_HUMAN
	3.556	0.5927	0.932974	Tyrosine-protein kinase JAK3	JAK3_HUMAN
	3.678	0.613	0.924107	[Pyruvate dehydrogenase [lipoamide]] kinase isozyme 2, mitochondrial	PDK2_HUMAN
	3.958	0.3045	0.919847	Endothelial protein C receptor	EPCR_HUMAN
	4.13	0.5163	0.909869	Glucocorticoid receptor	GCR_HUMAN
	3.72	0.4133	0.873498	Leukotriene A-4 hydrolase	LKHA4_HUMAN
	3.254	0.6509	0.826951	Ephrin type-B receptor 4	EPHB4_HUMAN
**11,14-eicosadienoic acid**	4.636	0.4636	2.75318	Retinoic acid receptor gamma	RARG_HUMAN
	4.534	0.5667	2.60253	Retinoic acid receptor RXR-alpha	P19793
	4.419	0.5524	2.4917	Fatty acid-binding protein, brain	FABP7_HUMAN
	4.781	0.683	2.37677	Aldo-keto reductase family 1 member C2	AK1C2_HUMAN

	4.955	0.3812	2.05142	Vitamin D3 receptor	VDR_HUMAN
	3.415	0.5691	2.05106	Coagulation factor X	FA10_HUMAN
	4.791	0.3993	1.89034	Alpha-tocopherol transfer protein	P49638
	3.446	0.5743	1.82616	Flavin reductase	P30043
	3.361	0.6722	1.75821	E3 ubiquitin-protein ligase Mdm2	MDM2_HUMAN
	3.314	0.6627	1.63403	Alcohol dehydrogenase 1C	P00326
	3.329	0.3699	1.59647	Peptidyl-prolyl cis-trans isomerase FKBP1B	FKB1B_HUMAN
	3.355	0.6709	1.56896	Lanosterol synthase	ERG7_HUMAN
	3.429	0.4899	1.47307	Protein S100-A9	S10A9_HUMAN
	3.362	0.6725	1.1951	Sorbitol dehydrogenase	DHSO_HUMAN
	3.342	0.4178	1.14507	Bile acid receptor	Q96RI1
	3.171	0.5285	1.0923	Peroxisome proliferator-activated receptor delta	PPARD_HUMAN
	3.175	0.7938	1.08315	Collagenase 3	MMP13_HUMAN
	3.318	0.553	1.04098	Histone deacetylase 8	HDAC8_HUMAN
	3.39	0.339	1.01809	Ornithine aminotransferase, mitochondrial	OAT_HUMAN
	3.352	0.4189	1.00138	Betaine--homocysteine S-methyltransferase 1	Q93088
	3.192	0.2455	0.996543	Amine oxidase [flavin-containing] A	AOFA_HUMAN
	3.433	0.4904	0.927657	L-serine dehydratase	P20132
	3.35	0.4785	0.88826	Insulin-like growth factor IA	P01343
	3.177	0.6355	0.885145	Epoxide hydrolase 2	HYES_HUMAN
	3.122	0.5204	0.870546	Thyroid hormone receptor beta-2	P37243
	3.145	0.7864	0.865488	Progesterone receptor	PRGR_HUMAN
	3.408	0.3787	0.850232	Interleukin-2	IL2_HUMAN
	3.105	0.6209	0.840729	Macrophage metalloelastase	MMP12_HUMAN

	3.439	0.3439	0.828436	Histamine N-methyltransferase	P50135
	3.135	0.5225	0.822941	Serine hydroxymethyltransferase, cytosolic	GLYC_HUMAN
	2.994	0.9979	0.808337	Mitogen-activated protein kinase 1	MK01_HUMAN
**24-ethylcholest-22-enol**	4.216	0.8432	2.38525	Mineralocorticoid receptor	MCR_HUMAN
	3.828	0.9569	2.36463	Progesterone receptor	PRGR_HUMAN
	3.768	0.9421	2.35254	Nuclear receptor ROR-alpha	RORA_HUMAN
	4.273	0.8545	2.29529	SEC14-like protein 2	O76054
	4.3	0.6143	2.21343	Lanosterol synthase	ERG7_HUMAN
	4.579	0.6542	2.07982	Sulfotransferase family cytosolic 2B member 1	O00204
	4.223	0.8445	2.07862	Estradiol 17-beta-dehydrogenase 1	P14061
	3.806	0.7612	2.01494	Bile acid receptor	NR1H4_HUMAN
	3.719	0.9297	2.00493	Collagenase 3	MMP13_HUMAN
	3.746	0.7493	2.00172	cAMP-dependent protein kinase catalytic subunit alpha	P00517
	3.702	0.7405	1.99459	Histone deacetylase 8	Q9BY41
	4.416	0.552	1.95225	Retinoic acid receptor gamma	RARG_HUMAN
	4.095	0.6825	1.91865	Trafficking protein particle complex subunit 3	O43617
	4.36	0.6228	1.86551	Mitogen-activated protein kinase 10	MK10_HUMAN
	3.897	0.6496	1.75728	Retinol-binding protein 4	RET4_HUMAN
	3.875	0.775	1.74171	Corticosteroid 11-beta-dehydrogenase isozyme 1	DHI1_HUMAN
	3.903	0.6505	1.6156	Glutathione S-transferase A1	P08263
	4.302	0.717	1.60011	Sex hormone-binding globulin	SHBG_HUMAN
	3.781	0.6302	1.5422	Ephrin type-B receptor 4	EPHB4_HUMAN
	3.955	0.6591	1.53376	Adenosine kinase	P55263

	3.564	0.7129	1.47788	Aldo-keto reductase family 1 member C1	Q04828
	3.661	0.4068	1.46232	Phosphatidylinositol 3-kinase regulatory subunit alpha	P85A_HUMAN
	4.665	0.5184	1.45777	Dual specificity mitogen-activated protein kinase kinase 1	MP2K1_HUMAN
	3.686	0.7371	1.42662	Tyrosine-protein phosphatase non-receptor type 11	PTN11_HUMAN
	3.948	0.4387	1.41335	Growth factor receptor-bound protein 2	GRB2_HUMAN
	3.881	0.5544	1.39454	Cytochrome P450 2C8	CP2C8_HUMAN
	3.649	0.7298	1.36911	Calmodulin	CALM_HUMAN
	3.606	0.5152	1.34892	Protein S100-A9	S10A9_HUMAN
	4.27	0.427	1.32119	Vitamin D3 receptor	VDR_HUMAN
	3.811	0.4764	1.32019	Proto-oncogene tyrosine-protein kinase ABL1	P00519
	4.067	0.3129	1.26083	Endothelial protein C receptor	EPCR_HUMAN
	3.939	0.4924	1.24805	Ferrochelatase, mitochondrial	P22830
	3.837	0.4796	1.24072	Caspase-3	CASP3_HUMAN
	3.637	0.7274	1.23474	Wiskott-Aldrich syndrome protein	WASP_HUMAN
	3.539	0.5899	1.19409	Receptor tyrosine-protein kinase erbB-4	ERBB4_HUMAN
	3.591	0.5129	1.19134	72 kDa type IV collagenase	MMP2_HUMAN
	3.764	0.6274	1.17094	Glutathione-requiring prostaglandin D synthase	PTGD2_HUMAN
	3.496	0.5826	1.1549	Flavin reductase	P30043
	3.601	0.6001	1.12959	Oxysterols receptor LXR-beta	NR1H2_HUMAN
	3.628	0.6047	1.09513	Proto-oncogene tyrosine-protein kinase LCK	LCK_HUMAN
	4.132	0.3756	1.08409	Phosphatidylcholine transfer protein	Q9UKL6
	3.92	0.56	1.0807	Gastrotropin	FABP6_HU

					MAN
	3.329	0.6659	1.06936	Steroid hormone receptor ERR1	P11474
	4.397	0.4397	1.03765	Retinoic acid receptor RXR-beta	RXRB_HUMAN
	3.396	0.566	1.03269	Alpha-1-antitrypsin	A1AT_HUMAN
	5.593	0.5593	1.02925	Cellular retinoic acid-binding protein 2	P29373
	3.955	0.4394	1.0244	Angiopoietin-1 receptor	TIE2_HUMAN
	3.597	0.5139	0.978935	Tyrosine-protein kinase ZAP-70	ZAP70_HUMAN
	3.486	0.581	0.973469	Oxysterols receptor LXR-alpha	NR1H3_HUMAN
	3.301	0.6601	0.969257	Dipeptidyl peptidase 4	DPP4_HUMAN
	3.763	0.5375	0.949712	Hepatocyte nuclear factor 4-gamma	Q14541
	3.573	0.5104	0.931599	Insulin-like growth factor IA	P01343
	3.501	0.8754	0.901845	Vitamin D-binding protein	VTDB_HUMAN
	3.389	0.4236	0.894405	Heat shock protein HSP 90-beta	P08238
	3.693	0.6155	0.855751	Alcohol dehydrogenase class-3	ADHX_HUMAN
	3.508	0.5846	0.848496	Tyrosine-protein kinase ITK/TSK	Q08881
	3.961	0.4401	0.820917	Retinoic acid receptor beta	RARB_HUMAN
	3.44	0.5733	0.818556	Tyrosine-protein kinase JAK3	JAK3_HUMAN
	3.862	0.5518	0.801836	Fatty acid-binding protein, brain	FABP7_HUMAN
**24-ethylcholesta-5,22-dienol**	4.619	0.5133	2.62627	Growth factor receptor-bound protein 2	GRB2_HUMAN
	4.495	0.8991	2.46644	Estradiol 17-beta-dehydrogenase 1	P14061
	3.847	0.9618	2.42295	Nuclear receptor ROR-alpha	RORA_HUMAN
	4.367	0.8734	2.35145	SEC14-like protein 2	O76054
	3.873	0.9683	2.34253	Progesterone receptor	PRGR_HUMAN

	3.873	0.7746	2.30948	Histone deacetylase 8	Q9BY41
	3.827	0.9569	2.19872	Mineralocorticoid receptor	MCR_HUMAN
	4.509	0.6442	1.86243	Sulfotransferase family cytosolic 2B member 1	O00204
	4.125	0.6875	1.86097	Trafficking protein particle complex subunit 3	O43617
	4.074	0.679	1.8509	Glutathione S-transferase A1	P08263
	3.893	0.7785	1.8228	Calmodulin	CALM_HUMAN
	3.945	0.789	1.77562	Corticosteroid 11-beta-dehydrogenase isozyme 1	DHI1_HUMAN
	3.751	0.7503	1.75993	Bile acid receptor	NR1H4_HUMAN
	3.639	0.9097	1.71248	Collagenase 3	MMP13_HUMAN
	5.235	0.6544	1.69424	Retinol-binding protein 4	RET4_HUMAN
	3.663	0.7326	1.6777	cAMP-dependent protein kinase catalytic subunit alpha	P00517
	4.087	0.5839	1.67626	Cytochrome P450 2C8	CP2C8_HUMAN
	4.402	0.7336	1.65	Sex hormone-binding globulin	SHBG_HUMAN
	4.053	0.5066	1.55596	Caspase-3	CASP3_HUMAN
	4.506	0.4096	1.55171	Phosphatidylcholine transfer protein	Q9UKL6
	4.198	0.5247	1.4949	Retinoic acid receptor gamma	RARG_HUMAN
	3.988	0.6646	1.48686	Adenosine kinase	P55263
	3.695	0.6158	1.47343	Peroxisome proliferator-activated receptor alpha	PPARA_HUMAN
	3.559	0.7118	1.44384	Dipeptidyl peptidase 4	DPP4_HUMAN
	4.117	0.5147	1.44308	Ferrochelatase, mitochondrial	P22830
	3.741	0.7482	1.43917	Tyrosine-protein phosphatase non-receptor type 11	PTN11_HUMAN
	3.7	0.4111	1.43896	Phosphatidylinositol 3-kinase regulatory subunit alpha	P85A_HUMAN
	3.672	0.459	1.41474	Heat shock protein HSP 90-beta	P08238

	3.665	0.6108	1.37195	Receptor tyrosine-protein kinase erbB-4	ERBB4_HUMAN
	4.659	0.5177	1.35683	Dual specificity mitogen-activated protein kinase kinase 1	MP2K1_HUMAN
	4.15	0.5928	1.35627	Gastrotropin	FABP6_HUMAN
	3.605	0.6008	1.31294	Flavin reductase	P30043
	4.328	0.4328	1.31145	Vitamin D3 receptor	VDR_HUMAN
	3.801	0.543	1.28512	Tyrosine-protein kinase ZAP-70	ZAP70_HUMAN
	3.697	0.6162	1.26606	Ephrin type-B receptor 4	EPHB4_HUMAN
	3.828	0.638	1.23923	E3 ubiquitin-protein ligase Mdm2	MDM2_HUMAN
	3.536	0.5894	1.23737	Alpha-1-antitrypsin	A1AT_HUMAN
	3.832	0.6387	1.20005	Glutathione-requiring prostaglandin D synthase	PTGD2_HUMAN
	3.792	0.4739	1.1902	Proto-oncogene tyrosine-protein kinase ABL1	P00519
	3.478	0.6955	1.18914	Aldo-keto reductase family 1 member C1	Q04828
	3.592	0.5986	1.17625	NAD(P)H dehydrogenase [quinone] 1	NQO1_HUMAN
	3.907	0.6511	1.1631	Alcohol dehydrogenase class-3	ADHX_HUMAN
	3.615	0.6024	1.15405	Mitogen-activated protein kinase 10	P53779
	4.059	0.3122	1.14939	Endothelial protein C receptor	EPCR_HUMAN
	3.602	0.5146	1.12416	72 kDa type IV collagenase	MMP2_HUMAN
	3.889	0.5556	1.08226	Hepatocyte nuclear factor 4-gamma	Q14541
	3.74	0.5343	1.0575	Lanosterol synthase	ERG7_HUMAN
	3.366	0.6732	1.0565	Glutathione S-transferase P	GSTP1_HUMAN
	4.076	0.5823	1.05647	Fatty acid-binding protein, brain	FABP7_HUMAN

	3.564	0.594	1.04959	Oxysterols receptor LXR-alpha	NR1H3_HUMAN
	3.863	0.4828	1.04111	Retinoic acid receptor alpha	RARA_HUMAN
	3.67	0.6117	1.01578	Tyrosine-protein kinase SYK	KSYK_HUMAN
	3.337	0.6673	0.998714	Steroid hormone receptor ERR1	P11474
	3.603	0.6006	0.947067	Proto-oncogene tyrosine-protein kinase LCK	LCK_HUMAN
	3.52	0.5866	0.909362	Nuclear receptor subfamily 1 group I member 3	NR1I3_HUMAN
	4.355	0.4355	0.905455	Retinoic acid receptor RXR-beta	RXRB_HUMAN
	3.538	0.5897	0.892643	Oxysterols receptor LXR-beta	NR1H2_HUMAN
	3.51	0.5851	0.878404	Tyrosine-protein kinase JAK3	JAK3_HUMAN
	3.5	0.5	0.843112	Interleukin-2	IL2_HUMAN
	3.816	0.5452	0.808539	Fatty acid-binding protein, heart	FABPH_HUMAN
**24-methyl-31-norlanost-9(11)-enol**	4.841	0.6916	2.71808	cAMP-specific 3,5-cyclic phosphodiesterase 4D	PDE4D_HUMAN
	4.259	0.6084	2.48173	Protein S100-A9	S10A9_HUMAN
	4.399	0.6285	2.44267	Renin	RENI_HUMAN
	4	0.6667	2.32419	Ganglioside GM2 activator	P17900
	4.373	0.8746	2.11836	SEC14-like protein 2	O76054
	4.063	0.6772	2.07535	Peroxisome proliferator-activated receptor alpha	PPARA_HUMAN
	3.949	0.7899	1.97276	Bile acid receptor	NR1H4_HUMAN
	4.297	0.7161	1.94139	E3 ubiquitin-protein ligase Mdm2	MDM2_HUMAN
	4.153	0.6922	1.87409	Dipeptidyl peptidase 4	DPP4_HUMAN
	4.251	0.7085	1.87015	Trafficking protein particle complex subunit 3	O43617
	3.955	0.9888	1.85146	Kinesin-like protein KIF11	KIF11_HUMAN

	3.721	0.9303	1.8437	Nuclear receptor ROR-alpha	RORA_HUMAN
	3.979	0.4974	1.84108	Heat shock protein HSP 90-beta	P08238
	4.381	0.6258	1.73298	Hepatocyte nuclear factor 4-gamma	Q14541
	3.929	0.7859	1.72323	Prothrombin	THRB_HUMAN
	4.753	0.4321	1.71569	Serine/threonine-protein phosphatase PP1-gamma catalytic subunit	P36873
	3.709	0.7419	1.70366	Glutathione S-transferase P	GSTP1_HUMAN
	4.208	0.6012	1.6794	Cytochrome P450 2C8	CP2C8_HUMAN
	4.468	0.3437	1.60846	Endothelial protein C receptor	EPCR_HUMAN
	4.979	0.5532	1.56459	Dual specificity mitogen-activated protein kinase kinase 1	MP2K1_HUMAN
	5.278	0.6597	1.56242	Retinol-binding protein 4	RET4_HUMAN
	3.792	0.632	1.52339	Flavin reductase	P30043
	4.014	0.669	1.5034	Glutathione S-transferase A1	P08263
	3.907	0.5581	1.49393	72 kDa type IV collagenase	MMP2_HUMAN
	3.934	0.5619	1.48254	Interleukin-2	IL2_HUMAN
	4.081	0.6802	1.44559	Adenosine kinase	P55263
	4.405	0.4895	1.42435	Angiopoietin-1 receptor	TIE2_HUMAN
	3.87	0.774	1.4153	Wiskott-Aldrich syndrome protein	WASP_HUMAN
	3.616	0.7231	1.39687	Histone deacetylase 8	Q9BY41
	4.014	0.5017	1.39115	Proto-oncogene tyrosine-protein kinase ABL1	P00519
	4.105	0.4561	1.38511	Growth factor receptor-bound protein 2	GRB2_HUMAN
	4.515	0.5017	1.3284	Retinoic acid receptor beta	RARB_HUMAN
	3.917	0.6528	1.30083	Tyrosine-protein kinase SYK	KSYK_HUMAN
	3.775	0.6292	1.29955	Oxysterols receptor LXR-alpha	NR1H3_HU

					MAN
	4.256	0.6079	1.29328	Sulfotransferase family cytosolic 2B member 1	O00204
	3.761	0.7521	1.28423	Calmodulin	CALM_HUMAN
	3.798	0.9494	1.27014	Vitamin D-binding protein	VTDB_HUMAN
	3.881	0.6468	1.26886	Hepatocyte growth factor receptor	MET_HUMAN
	4.441	0.4037	1.26099	Phosphatidylcholine transfer protein	Q9UKL6
	4.513	0.5642	1.25847	Fatty acid-binding protein, heart	FABPH_HUMAN
	3.893	0.4866	1.25672	3-hydroxy-3-methylglutaryl-coenzyme A reductase	HMDH_HUMAN
	4.323	0.6176	1.252	Fatty acid-binding protein, brain	FABP7_HUMAN
	3.681	0.4602	1.19467	Vitamin D3 receptor	VDR_HUMAN
	4.384	0.548	1.15339	Glucocorticoid receptor	GCR_HUMAN
	4.697	0.4697	1.14708	Retinoic acid receptor RXR-beta	RXRB_HUMAN
	3.67	0.4078	1.14294	Phosphatidylinositol 3-kinase regulatory subunit alpha	P85A_HUMAN
	3.604	0.6007	1.13783	Tyrosine-protein phosphatase non-receptor type 1	PTN1_HUMAN
	3.899	0.6499	1.13081	Glutathione-requiring prostaglandin D synthase	PTGD2_HUMAN
	3.686	0.7372	1.0872	Tyrosine-protein phosphatase non-receptor type 11	PTN11_HUMAN
	4.085	0.5836	1.07326	Gastrotropin	FABP6_HUMAN
	3.723	0.7446	1.07273	Corticosteroid 11-beta-dehydrogenase isozyme 1	DHI1_HUMAN
	3.966	0.3606	1.0705	Caspase-1	CASP1_HUMAN
	3.743	0.5347	0.978141	Tyrosine-protein kinase ZAP-70	ZAP70_HUMAN
	3.404	0.6807	0.960505	Steroid hormone receptor ERR1	P11474
	3.769	0.6282	0.95958	[Pyruvate dehydrogenase	PDK2_HU

				[lipoamide]] kinase isozyme 2, mitochondrial	MAN
	3.397	0.4247	0.955421	Heme oxygenase 1	HMOX1_HUMAN
	3.637	0.5196	0.955378	Neutrophil gelatinase-associated lipocalin	NGAL_HUMAN
	3.917	0.4896	0.951895	Ferrochelatase, mitochondrial	P22830
	3.531	0.7062	0.948088	Alpha-tocopherol transfer protein	P49638
	3.634	0.6057	0.896048	Oxysterols receptor LXR-beta	NR1H2_HUMAN
	3.811	0.7623	0.886215	Bile salt sulfotransferase	Q06520
	3.57	0.51	0.87814	Basic fibroblast growth factor receptor 1	FGFR1_HUMAN
	3.75	0.5357	0.875328	Protein kinase C theta type	KPCT_HUMAN
	3.563	0.7125	0.864243	Mineralocorticoid receptor	MCR_HUMAN
	4.094	0.5118	0.854761	Histamine N-methyltransferase	P50135
	3.476	0.3862	0.848878	Bcl-2-like protein 1	Q07817
	5.595	0.5595	0.836732	Cellular retinoic acid-binding protein 2	P29373
	3.832	0.479	0.807156	Retinoic acid receptor alpha	RARA_HUMAN
**24-Methylcholest-5-enyl-3belta-O-glucopyranoside_qt**	4.636	0.4636	2.75318	Retinoic acid receptor gamma	RARG_HUMAN
	4.534	0.5667	2.60253	Retinoic acid receptor RXR-alpha	P19793
	4.419	0.5524	2.4917	Fatty acid-binding protein, brain	FABP7_HUMAN
	4.781	0.683	2.37677	Aldo-keto reductase family 1 member C2	AK1C2_HUMAN
	4.955	0.3812	2.05142	Vitamin D3 receptor	VDR_HUMAN
	3.415	0.5691	2.05106	Coagulation factor X	FA10_HUMAN
	4.791	0.3993	1.89034	Alpha-tocopherol transfer protein	P49638
	3.446	0.5743	1.82616	Flavin reductase	P30043
	3.361	0.6722	1.75821	E3 ubiquitin-protein ligase Mdm2	MDM2_HUMAN
	3.314	0.6627	1.63403	Alcohol dehydrogenase 1C	P00326
	3.329	0.3699	1.59647	Peptidyl-prolyl cis-trans	FKB1B_HU

				isomerase FKBP1B	MAN
	3.355	0.6709	1.56896	Lanosterol synthase	ERG7_HUMAN
	3.429	0.4899	1.47307	Protein S100-A9	S10A9_HUMAN
	3.362	0.6725	1.1951	Sorbitol dehydrogenase	DHSO_HUMAN
	3.342	0.4178	1.14507	Bile acid receptor	Q96RI1
	3.171	0.5285	1.0923	Peroxisome proliferator-activated receptor delta	PPARD_HUMAN
	3.175	0.7938	1.08315	Collagenase 3	MMP13_HUMAN
	3.318	0.553	1.04098	Histone deacetylase 8	HDAC8_HUMAN
	3.39	0.339	1.01809	Ornithine aminotransferase, mitochondrial	OAT_HUMAN
	3.352	0.4189	1.00138	Betaine--homocysteine S-methyltransferase 1	Q93088
	3.192	0.2455	0.996543	Amine oxidase [flavin-containing] A	AOFA_HUMAN
	3.433	0.4904	0.927657	L-serine dehydratase	P20132
	3.35	0.4785	0.88826	Insulin-like growth factor IA	P01343
	3.177	0.6355	0.885145	Epoxide hydrolase 2	HYES_HUMAN
	3.122	0.5204	0.870546	Thyroid hormone receptor beta-2	P37243
	3.145	0.7864	0.865488	Progesterone receptor	PRGR_HUMAN
	3.408	0.3787	0.850232	Interleukin-2	IL2_HUMAN
	3.105	0.6209	0.840729	Macrophage metalloelastase	MMP12_HUMAN
	3.439	0.3439	0.828436	Histamine N-methyltransferase	P50135
	3.135	0.5225	0.822941	Serine hydroxymethyltransferase, cytosolic	GLYC_HUMAN
	2.994	0.9979	0.808337	Mitogen-activated protein kinase 1	MK01_HUMAN
**24-methylenelanost-8-enol**	4.363	0.7272	2.47347	Trafficking protein particle complex subunit 3	O43617
	3.757	0.9392	2.36402	Nuclear receptor ROR-alpha	RORA_HUMAN
	4.218	0.703	2.19337	E3 ubiquitin-protein ligase	MDM2_HU

				Mdm2	MAN
	4.22	0.6028	2.17939	B-Raf proto-oncogene serine/threonine-protein kinase	BRAF1_HUMAN
	4.464	0.5581	2.13773	Ferrochelatase, mitochondrial	P22830
	4.174	0.8349	2.13033	SEC14-like protein 2	O76054
	3.816	0.7633	2.07634	Aldo-keto reductase family 1 member C1	Q04828
	3.812	0.7623	2.06693	Bile acid receptor	NR1H4_HUMAN
	3.619	0.7238	1.89106	Glutathione S-transferase P	GSTP1_HUMAN
	3.925	0.7849	1.85306	Mineralocorticoid receptor	MCR_HUMAN
	4.854	0.5393	1.7202	Dual specificity mitogen-activated protein kinase kinase 1	MP2K1_HUMAN
	3.575	0.5107	1.69588	3-hydroxy-3-methylglutaryl-coenzyme A reductase	HMDH_HUMAN
	3.893	0.4866	1.65538	Stromelysin-1	MMP3_HUMAN
	4.518	0.502	1.64	Retinoic acid receptor beta	RARB_HUMAN
	3.784	0.7567	1.57138	Corticosteroid 11-beta-dehydrogenase isozyme 1	DHI1_HUMAN
	4.118	0.5883	1.55249	Estrogen sulfotransferase	ST1E1_HUMAN
	3.753	0.5361	1.5299	72 kDa type IV collagenase	MMP2_HUMAN
	4.156	0.5937	1.48944	Gastrotropin	FABP6_HUMAN
	3.622	0.7244	1.47788	Alpha-tocopherol transfer protein	P49638
	3.904	0.5576	1.46817	Cytochrome P450 2C8	CP2C8_HUMAN
	3.482	0.6963	1.4469	Histone deacetylase 8	Q9BY41
	4.012	0.5732	1.43371	Hepatocyte nuclear factor 4-gamma	Q14541
	3.659	0.7318	1.42914	Calmodulin	CALM_HUMAN
	3.797	0.5424	1.42866	Insulin-like growth factor IA	P01343
	4.147	0.319	1.42306	Endothelial protein C receptor	EPCR_HUMAN
	3.878	0.6463	1.40746	Glutathione-requiring	PTGD2_HU

				prostaglandin D synthase	MAN
	3.612	0.602	1.39453	Receptor tyrosine-protein kinase erbB-4	ERBB4_HUMAN
	3.829	0.4787	1.38224	Proto-oncogene tyrosine-protein kinase ABL1	P00519
	4.099	0.5856	1.35566	Sulfotransferase family cytosolic 2B member 1	O00204
	3.758	0.5368	1.35538	Retinoic acid receptor gamma	RARG_HUMAN
	4.308	0.5385	1.34176	Glucocorticoid receptor	GCR_HUMAN
	3.371	0.5619	1.29538	cGMP-specific 3,5-cyclic phosphodiesterase	PDE5A_HUMAN
	3.607	0.6011	1.28953	Oxysterols receptor LXR-alpha	NR1H3_HUMAN
	3.889	0.3535	1.27243	Caspase-1	CASP1_HUMAN
	3.83	0.4256	1.26083	Serine/threonine-protein phosphatase PP1-gamma catalytic subunit	P36873
	3.624	0.604	1.21362	Oxysterols receptor LXR-beta	NR1H2_HUMAN
	3.694	0.6157	1.21128	Glutathione S-transferase A1	P08263
	3.745	0.749	1.19473	Estradiol 17-beta-dehydrogenase 1	P14061
	3.571	0.7143	1.18865	Tyrosine-protein phosphatase non-receptor type 11	PTN11_HUMAN
	3.706	0.6176	1.17072	[Pyruvate dehydrogenase [lipoamide]] kinase isozyme 2, mitochondrial	PDK2_HUMAN
	3.585	0.8963	1.15031	Vitamin D-binding protein	VTDB_HUMAN
	3.981	0.6636	1.14378	Sex hormone-binding globulin	SHBG_HUMAN
	3.605	0.6008	1.14221	Dipeptidyl peptidase 4	DPP4_HUMAN
	4.61	0.5763	1.1204	Retinol-binding protein 4	RET4_HUMAN
	3.552	0.592	1.10917	Tyrosine-protein kinase JAK3	JAK3_HUMAN
	3.648	0.6079	1.10146	Tyrosine-protein kinase SYK	KSYK_HU

					MAN
	3.556	0.7112	1.08206	Wiskott-Aldrich syndrome protein	WASP_HUMAN
	3.537	0.5895	1.08189	Nuclear receptor subfamily 1 group I member 3	NR1I3_HUMAN
	3.788	0.4735	1.02897	Retinoic acid receptor alpha	RARA_HUMAN
	3.352	0.5586	0.97003	Alpha-1-antitrypsin	A1AT_HUMAN
	3.828	0.5468	0.968446	Mitogen-activated protein kinase 10	MK10_HUMAN
	3.499	0.4998	0.958747	Interleukin-2	IL2_HUMAN
	3.268	0.5446	0.9539	Ganglioside GM2 activator	P17900
	3.397	0.5662	0.942851	Flavin reductase	P30043
	3.474	0.4964	0.933812	Neutrophil gelatinase-associated lipocalin	NGAL_HUMAN
	4.28	0.428	0.913422	Retinoic acid receptor RXR-beta	RXRB_HUMAN
	3.791	0.4739	0.910837	Peptidyl-prolyl cis-trans isomerase FKBP1A	FKB1A_HUMAN
	3.342	0.5571	0.910332	Leukocyte elastase	ELNE_HUMAN
	3.537	0.5896	0.89636	Hepatocyte growth factor receptor	MET_HUMAN
	3.373	0.4216	0.89173	Heat shock protein HSP 90-beta	P08238
	3.971	0.361	0.861017	Phosphatidylcholine transfer protein	Q9UKL6
	3.39	0.3767	0.844568	Phosphatidylinositol 3-kinase regulatory subunit alpha	P85A_HUMAN
	3.852	0.5503	0.810765	Fatty acid-binding protein, brain	FABP7_HUMAN
**31-norcyclolaudenol**	5.618	0.7022	2.23252	Glucocorticoid receptor	GCR_HUMAN
	4.465	0.893	2.21212	Calmodulin	CALM_HUMAN
	4.537	0.7562	2.14343	Oxysterols receptor LXR-beta	NR1H2_HUMAN
	5.119	0.7313	2.0764	Gastrotropin	FABP6_HUMAN
	4.63	0.7717	2.04419	[Pyruvate dehydrogenase	PDK2_HU

				[lipoamide]] kinase isozyme 2, mitochondrial	MAN
	4.708	0.7847	2.03251	Adenosine kinase	P55263
	5.306	0.4824	1.93329	Phosphatidylcholine transfer protein	Q9UKL6
	4.761	0.6801	1.92861	Thyroid hormone receptor alpha	P10827
	4.157	0.8313	1.8856	Bile acid receptor	NR1H4_HUMAN
	4	0.8	1.86717	Glutathione S-transferase P	GSTP1_HUMAN
	4	1	1.84271	Mitogen-activated protein kinase 14	Q16539
	4	0.8	1.78975	Histone deacetylase 8	Q9BY41
	4.775	0.5968	1.76639	Ferrochelatase, mitochondrial	P22830
	5.211	0.579	1.74554	Retinoic acid receptor beta	RARB_HUMAN
	3.935	0.9838	1.73109	Cell division protein kinase 2	P24941
	3.878	0.9695	1.67753	Peroxisome proliferator-activated receptor gamma	PPARG_HUMAN
	4.505	0.901	1.62828	Bile salt sulfotransferase	Q06520
	3.891	0.9728	1.61342	Estrogen-related receptor gamma	P62508
	4.784	0.6834	1.58479	Sulfotransferase family cytosolic 2B member 1	O00204
	3.903	0.6505	1.57996	Histo-blood group ABO system transferase	P16442
	4.865	0.6949	1.56297	Fatty acid-binding protein, brain	FABP7_HUMAN
	4.677	0.5846	1.55749	Retinoic acid receptor gamma	RARG_HUMAN
	4.914	0.6143	1.54784	Histamine N-methyltransferase	P50135
	3.788	0.9471	1.517	Coagulation factor X	FA10_HUMAN
	5.296	0.5884	1.47341	Dual specificity mitogen-activated protein kinase kinase 1	MP2K1_HUMAN
	3.938	0.6563	1.41629	Alpha-1-antitrypsin	A1AT_HUMAN
	3.919	0.6531	1.39644	Tyrosine-protein phosphatase non-receptor type 1	PTN1_HUMAN
	5.536	0.692	1.39108	Retinol-binding protein 4	RET4_HUMAN
	4.213	0.5267	1.3888	3-hydroxy-3-methylglutaryl-	HMDH_HU

				coenzyme A reductase	MAN
	4.365	0.6235	1.36195	Nuclear receptor subfamily 1 group I member 2	NR1I2_HUMAN
	4.269	0.6098	1.34826	Lanosterol synthase	ERG7_HUMAN
	4	0.8	1.34636	Prothrombin	THRB_HUMAN
	3.912	0.7824	1.29267	Alpha-tocopherol transfer protein	P49638
	4.249	0.7082	1.28678	Glutathione-requiring prostaglandin D synthase	PTGD2_HUMAN
	4.073	0.6788	1.27372	Dipeptidyl peptidase 4	DPP4_HUMAN
	4	0.6667	1.24245	Tyrosine-protein kinase JAK3	JAK3_HUMAN
	4.05	0.5063	1.23774	Vitamin D3 receptor	VDR_HUMAN
	3.989	0.6649	1.22777	Nuclear receptor subfamily 1 group I member 3	NR1I3_HUMAN
	4.122	0.5152	1.17502	Interleukin-2	IL2_HUMAN
	3.935	0.5621	1.14634	Proactivator polypeptide	P07602
	3.907	0.7814	1.13186	Mineralocorticoid receptor	MCR_HUMAN
	4	0.6667	1.11381	Tyrosine-protein kinase ITK/TSK	Q08881
	4.478	0.6397	1.11326	Fatty acid-binding protein, heart	FABPH_HUMAN
	3.633	0.7267	1.11229	E3 ubiquitin-protein ligase Mdm2	MDM2_HUMAN
	3.949	0.9873	1.10976	Vitamin D-binding protein	VTDB_HUMAN
	3.987	0.6645	1.10234	cAMP-specific 3,5-cyclic phosphodiesterase 4D	PDE4D_HUMAN
	3.576	0.8941	1.09928	Liver carboxylesterase 1	EST1_HUMAN
	3.547	0.8867	1.08988	Beta-secretase 1	BACE1_HUMAN
	5.008	0.5564	1.08016	Cellular retinoic acid-binding protein 2	P29373
	4.98	0.498	1.06691	Retinoic acid receptor RXR-beta	RXRB_HUMAN

	4	0.6667	1.05396	Hepatocyte growth factor receptor	MET_HUMAN
	3.573	0.8934	1.02282	Progesterone receptor	PRGR_HUMAN
	3.947	0.4934	1.00245	Stromelysin-1	MMP3_HUMAN
	3.977	0.6628	0.997181	Glutathione S-transferase A1	P08263
	3.898	0.7796	0.992914	Corticosteroid 11-beta-dehydrogenase isozyme 1	DHI1_HUMAN
	3.579	0.7159	0.990347	Aldo-keto reductase family 1 member C3	AK1C3_HUMAN
	4.321	0.3324	0.954336	Endothelial protein C receptor	EPCR_HUMAN
	4.149	0.461	0.933011	Mast/stem cell growth factor receptor	KIT_HUMAN
	3.965	0.7929	0.882567	Estradiol 17-beta-dehydrogenase 1	P14061
	3.895	0.5564	0.864838	Tyrosine-protein kinase ZAP-70	ZAP70_HUMAN
	3.586	0.7172	0.863035	Aldo-keto reductase family 1 member C1	Q04828
	3.769	0.6281	0.861573	Oxysterols receptor LXR-alpha	NR1H3_HUMAN
	4.051	0.4501	0.843621	Growth factor receptor-bound protein 2	GRB2_HUMAN
	4.201	0.7002	0.832671	Sex hormone-binding globulin	SHBG_HUMAN
**31-norlanost-9(11)-enol**	4.344	0.543	2.77699	Heat shock protein HSP 90-beta	P08238
	4.367	0.5459	2.31395	Interleukin-2	IL2_HUMAN
	4.193	0.5991	2.19126	Renin	RENI_HUMAN
	4.308	0.7181	2.18661	[Pyruvate dehydrogenase [lipoamide]] kinase isozyme 2, mitochondrial	PDK2_HUMAN
	3.962	0.7925	2.16722	Bile acid receptor	NR1H4_HUMAN
	4.273	0.7122	2.06597	Trafficking protein particle complex subunit 3	O43617
	3.955	0.7909	1.96263	Prothrombin	THRB_HUMAN

	3.702	0.9255	1.95061	Nuclear receptor ROR-alpha	RORA_HUMAN
	4.58	0.3523	1.93993	Endothelial protein C receptor	EPCR_HUMAN
	3.734	0.9334	1.89841	Mitogen-activated protein kinase 14	Q16539
	4.562	0.6517	1.87993	Sulfotransferase family cytosolic 2B member 1	O00204
	3.841	0.6401	1.8078	Poly [ADP-ribose] polymerase 1	P09874
	3.905	0.4881	1.77752	3-hydroxy-3-methylglutaryl-coenzyme A reductase	HMDH_HUMAN
	3.842	0.9606	1.75258	Kinesin-like protein KIF11	KIF11_HUMAN
	4.223	0.6033	1.74573	cAMP-specific 3,5-cyclic phosphodiesterase 4D	PDE4D_HUMAN
	4.706	0.5229	1.72027	Retinoic acid receptor beta	RARB_HUMAN
	5.074	0.5074	1.71536	Retinoic acid receptor RXR-beta	RXRB_HUMAN
	3.819	0.6365	1.69508	Oxysterols receptor LXR-beta	NR1H2_HUMAN
	4.065	0.813	1.69098	SEC14-like protein 2	O76054
	3.683	0.4603	1.66275	Heme oxygenase 1	HMOX1_HUMAN
	4.659	0.5823	1.66194	Glucocorticoid receptor	GCR_HUMAN
	4.09	0.5842	1.60834	Cytochrome P450 2C8	CP2C8_HUMAN
	3.843	0.6405	1.59395	Oxysterols receptor LXR-alpha	NR1H3_HUMAN
	3.867	0.9667	1.59271	Vitamin D-binding protein	VTDB_HUMAN
	4.574	0.4158	1.59128	Phosphatidylcholine transfer protein	Q9UKL6
	3.637	0.7274	1.5738	Steroid hormone receptor ERR1	P11474
	4.142	0.6903	1.52862	Alcohol dehydrogenase class-3	ADHX_HUMAN
	3.755	0.6258	1.52528	Peroxisome proliferator-activated receptor alpha	PPARA_HUMAN
	3.843	0.549	1.50108	72 kDa type IV collagenase	MMP2_HUMAN

	3.841	0.7683	1.49932	Wiskott-Aldrich syndrome protein	WASP_HUMAN
	3.816	0.636	1.47857	Nuclear receptor subfamily 1 group I member 3	NR1I3_HUMAN
	3.922	0.6537	1.46303	Glutathione S-transferase A1	P08263
	3.702	0.617	1.45824	Flavin reductase	P30043
	4.158	0.5198	1.44507	Ferrochelatase, mitochondrial	P22830
	3.881	0.5545	1.38931	Retinoic acid receptor gamma	RARG_HUMAN
	3.578	0.7155	1.38241	cAMP-dependent protein kinase catalytic subunit alpha	P00517
	3.728	0.7457	1.35158	Calmodulin	CALM_HUMAN
	4.061	0.5801	1.31863	Hepatocyte nuclear factor 4-gamma	Q14541
	3.851	0.6418	1.30538	Tyrosine-protein kinase SYK	KSYK_HUMAN
	3.802	0.4752	1.26494	Stromelysin-1	MMP3_HUMAN
	3.463	0.6926	1.21533	Glutathione S-transferase P	GSTP1_HUMAN
	3.87	0.645	1.17636	Adenosine kinase	P55263
	4.538	0.5042	1.15511	Dual specificity mitogen-activated protein kinase kinase 1	MP2K1_HUMAN
	3.881	0.7762	1.14352	Bile salt sulfotransferase	Q06520
	3.666	0.611	1.1422	Tyrosine-protein kinase JAK3	JAK3_HUMAN
	3.695	0.4619	1.12389	Vitamin D3 receptor	VDR_HUMAN
	4.033	0.5762	1.10923	Gastrotropin	FABP6_HUMAN
	3.588	0.5125	1.10824	Protein S100-A9	S10A9_HUMAN
	3.51	0.5851	1.10582	Alpha-1-antitrypsin	A1AT_HUMAN
	3.442	0.6884	1.09218	Histone deacetylase 8	Q9BY41
	3.842	0.4269	1.07013	Serine/threonine-protein phosphatase PP1-gamma catalytic subunit	P36873
	3.862	0.4291	1.0562	Growth factor receptor-bound protein 2	GRB2_HUMAN

	3.377	0.6755	1.04121	E3 ubiquitin-protein ligase Mdm2	MDM2_HUMAN
	4.057	0.4508	1.03348	Angiopoietin-1 receptor	TIE2_HUMAN
	3.631	0.6051	1.02865	Retinol-binding protein 4	RET4_HUMAN
	3.735	0.4668	1.01849	Proto-oncogene tyrosine-protein kinase ABL1	P00519
	3.492	0.6984	0.978512	Alpha-tocopherol transfer protein	P49638
	3.717	0.7433	0.947652	Estradiol 17-beta-dehydrogenase 1	P14061
	3.923	0.5604	0.946604	Mitogen-activated protein kinase 10	MK10_HUMAN
	3.623	0.5176	0.864196	Tyrosine-protein kinase ZAP-70	ZAP70_HUMAN
	5.499	0.5499	0.863123	Cellular retinoic acid-binding protein 2	P29373
	3.595	0.5991	0.858888	Tyrosine-protein kinase ITK/TSK	Q08881
	3.564	0.7129	0.857779	Corticosteroid 11-beta-dehydrogenase isozyme 1	DHI1_HUMAN
	3.524	0.7048	0.849626	Tyrosine-protein phosphatase non-receptor type 11	PTN11_HUMAN
	3.67	0.5242	0.842425	Protein kinase C theta type	KPCT_HUMAN
	3.486	0.498	0.823198	Basic fibroblast growth factor receptor 1	FGFR1_HUMAN
	3.841	0.4801	0.819841	Peptidyl-prolyl cis-trans isomerase FKBP1A	FKB1A_HUMAN
	3.302	0.5504	0.819361	Ganglioside GM2 activator	P17900
	3.716	0.5309	0.804874	Nuclear receptor subfamily 1 group I member 2	NR1I2_HUMAN
**31-norlanosterol**	5.061	0.723	3.17901	Lanosterol synthase	ERG7_HUMAN
	4.514	0.5643	2.52853	Vitamin D3 receptor	VDR_HUMAN
	4.587	0.7645	2.45819	Trafficking protein particle complex subunit 3	O43617
	4.966	0.382	2.36835	Endothelial protein C receptor	EPCR_HUMAN
	5.636	0.6263	2.31898	Dual specificity mitogen-	MP2K1_HU

				activated protein kinase kinase 1	MAN
	3.971	0.9927	2.29424	Progesterone receptor	PRGR_HUMAN
	3.946	0.7893	2.08373	cAMP-dependent protein kinase catalytic subunit alpha	P00517
	4.346	0.8691	2.04844	SEC14-like protein 2	O76054
	3.925	0.7851	1.95641	Mineralocorticoid receptor	MCR_HUMAN
	4.374	0.6248	1.95547	Cytochrome P450 2C8	CP2C8_HUMAN
	4	0.6667	1.91565	Peroxisome proliferator-activated receptor alpha	PPARA_HUMAN
	5.503	0.6879	1.77204	Retinol-binding protein 4	RET4_HUMAN
	4.466	0.638	1.76464	Estrogen sulfotransferase	ST1E1_HUMAN
	3.825	0.765	1.67803	Bile acid receptor	NR1H4_HUMAN
	4.783	0.5314	1.66578	Retinoic acid receptor beta	RARB_HUMAN
	3.646	0.9114	1.64699	Nuclear receptor ROR-alpha	RORA_HUMAN
	3.789	0.7577	1.63443	Vascular endothelial growth factor receptor 2	P35968
	3.868	0.4835	1.59848	Heat shock protein HSP 90-beta	P08238
	4.394	0.5492	1.58726	Retinoic acid receptor gamma	RARG_HUMAN
	3.911	0.6519	1.57891	Oxysterols receptor LXR-alpha	NR1H3_HUMAN
	4.198	0.4665	1.54284	Growth factor receptor-bound protein 2	GRB2_HUMAN
	3.806	0.6344	1.54217	Flavin reductase	P30043
	3.677	0.7353	1.53664	Histone deacetylase 8	Q9BY41
	3.82	0.764	1.53468	Alpha-tocopherol transfer protein	P49638
	3.612	0.7223	1.44806	Glutathione S-transferase P	GSTP1_HUMAN
	4.21	0.5263	1.42249	Retinoic acid receptor alpha	RARA_HUMAN
	4.081	0.5102	1.37379	Caspase-3	CASP3_HUMAN
	3.605	0.721	1.37295	Steroid hormone receptor ERR1	P11474

	4.199	0.5249	1.36943	Ferrochelatase, mitochondrial	P22830
	4.867	0.4867	1.32447	Retinoic acid receptor RXR-beta	RXRB_HUMAN
	3.806	0.5437	1.30348	72 kDa type IV collagenase	MMP2_HUMAN
	3.814	0.9536	1.29334	Vitamin D-binding protein	VTDB_HUMAN
	3.818	0.7635	1.28989	Wiskott-Aldrich syndrome protein	WASP_HUMAN
	6.292	0.6292	1.28147	Cellular retinoic acid-binding protein 2	P29373
	3.987	0.6645	1.26769	Glutathione-requiring prostaglandin D synthase	PTGD2_HUMAN
	3.53	0.8825	1.26298	Mitogen-activated protein kinase 14	Q16539
	4.208	0.6012	1.24486	Gastrotropin	FABP6_HUMAN
	4.429	0.4027	1.22736	Phosphatidylcholine transfer protein	Q9UKL6
	3.796	0.7592	1.21358	Corticosteroid 11-beta-dehydrogenase isozyme 1	DHI1_HUMAN
	3.702	0.4113	1.20345	Phosphatidylinositol 3-kinase regulatory subunit alpha	P85A_HUMAN
	3.852	0.6421	1.19787	Hepatocyte growth factor receptor	MET_HUMAN
	3.632	0.6053	1.18942	Poly [ADP-ribose] polymerase 1	P09874
	3.907	0.7814	1.16177	Estradiol 17-beta-dehydrogenase 1	P14061
	3.824	0.6373	1.12181	Glutathione S-transferase A1	P08263
	4	0.6667	1.11692	Alcohol dehydrogenase class-3	ADHX_HUMAN
	3.816	0.6361	1.0927	Tyrosine-protein kinase SYK	KSYK_HUMAN
	3.609	0.6015	1.09195	Tyrosine-protein phosphatase non-receptor type 1	PTN1_HUMAN
	3.667	0.7334	1.03442	Tyrosine-protein phosphatase non-receptor type 11	PTN11_HUMAN
	3.607	0.5153	1.01232	Protein S100-A9	S10A9_HUMAN
	3.671	0.5244	0.980914	Interleukin-2	IL2_HUMAN

	3.624	0.7248	0.970122	Calmodulin	CALM_HUMAN
	3.849	0.4277	0.941614	Serine/threonine-protein phosphatase PP1-gamma catalytic subunit	P36873
	4.162	0.5203	0.940518	Histamine N-methyltransferase	P50135
	3.836	0.7672	0.920744	Bile salt sulfotransferase	Q06520
	4.051	0.4502	0.902438	Angiopoietin-1 receptor	TIE2_HUMAN
	3.888	0.5555	0.881143	Hepatocyte nuclear factor 4-gamma	Q14541
	3.959	0.5655	0.85066	Sulfotransferase family cytosolic 2B member 1	O00204
	3.773	0.6288	0.850271	Adenosine kinase	P55263
	3.421	0.6842	0.841032	Serine/threonine-protein kinase Chk1	CHK1_HUMAN
	3.767	0.4186	0.828254	Cathepsin B	CATB_HUMAN
	3.422	0.5703	0.81609	Alpha-1-antitrypsin	A1AT_HUMAN
**Atropine**	3.556	0.4445	4.51205	Eukaryotic translation initiation factor 4E	P06730
	3.354	0.4791	3.8744	Dihydrofolate reductase	DYR_HUMAN
	3.687	0.6146	2.37246	Phosphoenolpyruvate carboxykinase, cytosolic [GTP]	P35558
	3.448	0.3831	2.0066	5(3)-deoxyribonucleotidase, mitochondrial	Q9NPB1
	2.966	0.7414	1.9018	Cathepsin B	CATB_HUMAN
	2.914	0.7284	1.77799	Galectin-7	LEG7_HUMAN
	2.933	0.9777	1.77356	Peptidyl-prolyl cis-trans isomerase A	P62937
	2.941	0.7353	1.76386	Carnitine O-acetyltransferase	CACP_HUMAN
	2.952	0.2684	1.65668	Histone-lysine N-methyltransferase SETD8	SETD8_HUMAN
	2.98	0.7449	1.63124	Proto-oncogene tyrosine-protein kinase Src	SRC_HUMAN
	2.916	0.7291	1.60151	Ig lambda chain V-II region	P01709

				MGC	
	2.921	0.7302	1.59683	Cell division protein kinase 2	P24941
	2.907	0.7269	1.55304	Stromelysin-1	MMP3_HUMAN
	2.868	0.717	1.55122	Aldose reductase	ALDR_HUMAN
	3.876	0.3876	1.52787	Sulfotransferase family cytosolic 2B member 1	O00204
	2.907	0.9691	1.52206	Oxysterols receptor LXR-beta	NR1H2_HUMAN
	2.899	0.3624	1.35829	Dipeptidyl peptidase 4	DPP4_HUMAN
	2.873	0.7181	1.34996	Prothrombin	THRB_HUMAN
	2.954	0.3693	1.32569	Signal transducer and activator of transcription 1-alpha/beta	STAT1_HUMAN
	2.841	0.7101	1.31656	Coagulation factor X	FA10_HUMAN
	3.824	0.3824	1.27547	Neprilysin	NEP_HUMAN
	2.84	0.5681	1.26701	Urokinase-type plasminogen activator	UROK_HUMAN
	2.987	0.3733	1.2636	Coagulation factor XI	FA11_HUMAN
	2.956	0.4223	1.26258	cAMP-dependent protein kinase, alpha-catalytic subunit	P00517
	2.902	0.4146	1.22077	Endoplasmic reticulum mannosyl-oligosaccharide 1,2-alpha-mannosidase	Q9UKM7
	2.949	0.3277	1.18953	Histone acetyltransferase PCAF	Q92831
	2.933	0.419	1.18716	Renin	RENI_HUMAN
	2.934	0.4191	1.17317	Spermidine synthase	P19623
	2.823	0.5646	1.16432	Cathepsin K	CATK_HUMAN
	2.95	0.59	1.13826	Tyrosine-protein phosphatase non-receptor type 1	PTN1_HUMAN
	2.903	0.7258	1.11879	Serum amyloid P-component	P02743
	2.84	0.5681	1.06841	Superoxide dismutase [Mn], mitochondrial	P04179
	2.943	0.5886	1.03777	Ornithine carbamoyltransferase,	P00480

				mitochondrial	
	2.847	0.5694	1.03476	Macrophage metalloelastase	MMP12_HUMAN
	2.76	0.69	1.03022	Carbonic anhydrase 2	CAH2_HUMAN
	2.913	0.4855	1.02053	Dihydroorotate dehydrogenase, mitochondrial	PYRD_HUMAN
	2.775	0.6938	1.01103	Complement factor D	CFAD_HUMAN
	2.785	0.4642	1.00429	Fatty acid-binding protein, adipocyte	FABP4_HUMAN
	2.849	0.4748	0.983223	Peroxisome proliferator-activated receptor gamma	PPARG_HUMAN
	2.838	0.4054	0.962224	Arginase-2, mitochondrial	ARGI2_HUMAN
	2.884	0.3204	0.940296	Pancreatic alpha-amylase	AMYP_HUMAN
	3.178	0.3178	0.93682	ADAM 33	ADA33_HUMAN
	2.718	0.9062	0.910828	Cholinesterase	CHLE_HUMAN
	2.741	0.4569	0.907249	Glycogen synthase kinase-3 beta	GSK3B_HUMAN
	2.839	0.3549	0.873073	Beta-secretase 1	P56817
	2.945	0.3682	0.870922	Betaine--homocysteine S-methyltransferase 1	Q93088
	2.758	0.6896	0.869062	Protein farnesyltransferase/geranylgeranyltransferase type-1 subunit alpha	FNTA_HUMAN
	2.848	0.4747	0.864099	Eosinophil cationic protein	P12724
	2.825	0.4709	0.85758	Thymidylate synthase	TYSY_HUMAN
	2.982	0.3728	0.85732	ADP-ribosylation factor 4	P18085
	2.719	0.6797	0.825916	Casein kinase I isoform gamma-2	KC1G2_HUMAN
**campesterol**	4.633	0.9266	2.66174	Estradiol 17-beta-dehydrogenase 1	P14061
	3.929	0.9823	2.55778	Nuclear receptor ROR-alpha	RORA_HUMAN
	4.543	0.5048	2.41179	Growth factor receptor-bound protein 2	GRB2_HUMAN

	4.393	0.8787	2.33989	SEC14-like protein 2	O76054
	3.822	0.9556	2.12912	Progesterone receptor	PRGR_HUMAN
	3.935	0.7869	2.1248	Bile acid receptor	NR1H4_HUMAN
	3.783	0.9458	2.04746	Mineralocorticoid receptor	MCR_HUMAN
	4.318	0.7197	2.00872	Glutathione-requiring prostaglandin D synthase	PTGD2_HUMAN
	3.753	0.7506	1.92168	Histone deacetylase 8	Q9BY41
	4.227	0.6039	1.89956	Lanosterol synthase	ERG7_HUMAN
	3.941	0.6568	1.83502	Oxysterols receptor LXR-alpha	NR1H3_HUMAN
	3.86	0.965	1.81437	Kinesin-like protein KIF11	KIF11_HUMAN
	4.127	0.6878	1.8063	Trafficking protein particle complex subunit 3	O43617
	4.488	0.6412	1.78123	Sulfotransferase family cytosolic 2B member 1	O00204
	4.283	0.5354	1.71945	Retinoic acid receptor alpha	RARA_HUMAN
	3.832	0.7665	1.6746	Prothrombin	THRB_HUMAN
	4.446	0.7409	1.66715	Sex hormone-binding globulin	SHBG_HUMAN
	4.119	0.5149	1.62447	Caspase-3	CASP3_HUMAN
	3.851	0.9628	1.57575	Vitamin D-binding protein	VTDB_HUMAN
	3.745	0.7489	1.57474	Androgen receptor	ANDR_HUMAN
	5.126	0.6407	1.53916	Retinol-binding protein 4	RET4_HUMAN
	3.861	0.7722	1.52653	Corticosteroid 11-beta-dehydrogenase isozyme 1	DHI1_HUMAN
	3.603	0.7206	1.51953	Steroid hormone receptor ERR1	P11474
	4.239	0.5299	1.51044	Retinoic acid receptor gamma	RARG_HUMAN
	4.166	0.5208	1.47204	Ferrochelatase, mitochondrial	P22830
	4.754	0.5282	1.43304	Dual specificity mitogen-	MP2K1_HU

				activated protein kinase kinase 1	MAN
	4.091	0.5844	1.38773	Hepatocyte nuclear factor 4-gamma	Q14541
	3.936	0.5623	1.3395	Cytochrome P450 2C8	CP2C8_HUMAN
	4.135	0.5907	1.31887	Mitogen-activated protein kinase 10	MK10_HUMAN
	3.5	0.5834	1.31697	Ganglioside GM2 activator	P17900
	3.922	0.6536	1.29686	Adenosine kinase	P55263
	4.156	0.3197	1.25843	Endothelial protein C receptor	EPCR_HUMAN
	3.455	0.8639	1.23811	Collagenase 3	MMP13_HUMAN
	3.637	0.4041	1.22405	Phosphatidylinositol 3-kinase regulatory subunit alpha	P85A_HUMAN
	3.675	0.735	1.21955	Tyrosine-protein phosphatase non-receptor type 11	PTN11_HUMAN
	3.661	0.7322	1.2177	Calmodulin	CALM_HUMAN
	3.703	0.7405	1.20975	Wiskott-Aldrich syndrome protein	WASP_HUMAN
	3.792	0.474	1.2056	3-hydroxy-3-methylglutaryl-coenzyme A reductase	HMDH_HUMAN
	3.777	0.6295	1.18777	Glutathione S-transferase A1	P08263
	4.059	0.5798	1.16426	Gastrotropin	FABP6_HUMAN
	3.475	0.695	1.15679	cAMP-dependent protein kinase catalytic subunit alpha	P00517
	3.447	0.6894	1.14067	Dipeptidyl peptidase 4	DPP4_HUMAN
	3.629	0.5184	1.12152	72 kDa type IV collagenase	MMP2_HUMAN
	3.673	0.6122	1.12101	Oxysterols receptor LXR-beta	NR1H2_HUMAN
	3.574	0.5956	1.10785	Receptor tyrosine-protein kinase erbB-4	ERBB4_HUMAN
	3.739	0.6231	1.09717	Tyrosine-protein kinase SYK	KSYK_HUMAN
	3.517	0.5862	1.08765	Poly [ADP-ribose] polymerase 1	P09874
	3.686	0.6144	1.05711	Proto-oncogene tyrosine-protein kinase LCK	LCK_HUMAN

	4.103	0.5861	1.0507	Fatty acid-binding protein, brain	FABP7_HUMAN
	3.525	0.5876	1.04797	Flavin reductase	P30043
	3.302	0.8255	1.03947	Bile salt sulfotransferase	Q06520
	3.547	0.5067	1.034	Protein S100-A9	S10A9_HUMAN
	3.664	0.6107	1.03027	Tyrosine-protein kinase ITK/TSK	Q08881
	3.863	0.6439	1.02551	Alcohol dehydrogenase class-3	ADHX_HUMAN
	3.727	0.4659	1.02042	Proto-oncogene tyrosine-protein kinase ABL1	P00519
	3.667	0.5238	0.967487	Tyrosine-protein kinase ZAP-70	ZAP70_HUMAN
	3.342	0.8355	0.964636	Mitogen-activated protein kinase 14	Q16539
	3.568	0.5946	0.944967	Tyrosine-protein kinase JAK3	JAK3_HUMAN
	3.808	0.4231	0.902243	Mast/stem cell growth factor receptor	KIT_HUMAN
	5.544	0.5544	0.900073	Cellular retinoic acid-binding protein 2	P29373
	3.539	0.5898	0.889477	Nuclear receptor subfamily 1 group I member 3	NR1I3_HUMAN
	3.535	0.5891	0.880579	Ephrin type-B receptor 4	EPHB4_HUMAN
	3.542	0.506	0.870491	Interleukin-2	IL2_HUMAN
	3.436	0.4295	0.846212	Heat shock protein HSP 90-beta	P08238
	3.438	0.5731	0.801752	Peroxisome proliferator-activated receptor alpha	PPARA_HUMAN
**Cycloartenol**	3.484	0.5807	2.5178	Nuclear receptor subfamily 1 group I member 3	NR1I3_HUMAN
	3.63	0.4537	2.33584	Caspase-3	CASP3_HUMAN
	3.474	0.4963	2.30011	72 kDa type IV collagenase	MMP2_HUMAN
	3.807	0.2928	2.21527	Endothelial protein C receptor	EPCR_HUMAN
	3.575	0.3972	2.14867	Growth factor receptor-bound protein 2	GRB2_HUMAN

	3.402	0.567	1.87454	Trafficking protein particle complex subunit 3	O43617
	3.451	0.3834	1.86372	Serine/threonine-protein phosphatase PP1-gamma catalytic subunit	P36873
	3.519	0.5027	1.81061	Hepatocyte nuclear factor 4-gamma	Q14541
	3.35	0.5584	1.80509	Tyrosine-protein kinase SYK	KSYK_HUMAN
	3.261	0.5436	1.78231	Tyrosine-protein phosphatase non-receptor type 1	PTN1_HUMAN
	2.959	0.9863	1.70913	Steryl-sulfatase	STS_HUMAN
	2.944	0.9812	1.64268	Apolipoprotein A-II	APOA2_HUMAN
	3.306	0.5509	1.63063	[Pyruvate dehydrogenase [lipoamide]] kinase isozyme 2, mitochondrial	PDK2_HUMAN
	3.934	0.3934	1.62687	Nuclear receptor subfamily 1 group I member 2	NR1I2_HUMAN
	3.531	0.5045	1.61676	Gastrotropin	FABP6_HUMAN
	2.994	0.5989	1.554	Glutathione S-transferase P	GSTP1_HUMAN
	2.975	0.595	1.5448	Epidermal growth factor receptor	EGFR_HUMAN
	3.256	0.6513	1.49716	SEC14-like protein 2	O76054
	2.987	0.4978	1.46066	Histo-blood group ABO system transferase	P16442
	3.167	0.5278	1.45933	Integrin alpha-L	ITAL_HUMAN
	2.851	0.9502	1.457	Transthyretin	TTHY_HUMAN
	2.918	0.5836	1.43716	Renin	RENI_HUMAN
	2.995	0.4992	1.39549	Insulin-like growth factor IA	P01343
	3.405	0.4256	1.39364	Phospholipase A2, membrane associated	P14555
	2.877	0.9591	1.39119	Amine oxidase [flavin-containing] B	AOFB_HUMAN
	3.534	0.4417	1.37165	Histamine N-methyltransferase	P50135

	2.85	0.9499	1.35567	Serum albumin	ALBU_HUMAN
	3.566	0.4457	1.30484	Glucocorticoid receptor	GCR_HUMAN
	2.909	0.7273	1.30375	Cell division protein kinase 2	P24941
	2.878	0.7195	1.2254	Mitogen-activated protein kinase 8	MK08_HUMAN
	3.329	0.4756	1.19123	Mitogen-activated protein kinase 10	MK10_HUMAN
	3.567	0.4458	1.18939	Retinoic acid receptor RXR-alpha	RXRA_HUMAN
	2.847	0.5693	1.09764	Androgen receptor	ANDR_HUMAN
	2.766	0.9221	1.0919	Prothrombin	THRB_HUMAN
	2.907	0.5813	1.07097	Proto-oncogene tyrosine-protein kinase Src	SRC_HUMAN
	3.59	0.3989	1.03292	Dual specificity mitogen-activated protein kinase kinase 1	MP2K1_HUMAN
	2.922	0.487	1.00399	Poly [ADP-ribose] polymerase 1	P09874
	2.973	0.5947	0.967932	Calmodulin	CALM_HUMAN
	2.926	0.3658	0.953792	Heat shock protein HSP 90-beta	P08238
	2.962	0.5925	0.90149	Tyrosine-protein phosphatase non-receptor type 11	PTN11_HUMAN
	2.784	0.6959	0.899418	Nuclear receptor ROR-alpha	RORA_HUMAN
	2.706	0.9018	0.890307	Kinesin-like protein KIF11	KIF11_HUMAN
	2.704	0.5409	0.858343	Stromelysin-1	MMP3_HUMAN
	3.246	0.4637	0.845893	Fatty acid-binding protein, heart	FABPH_HUMAN
	2.894	0.4824	0.841731	Flavin reductase	P30043
	2.897	0.4829	0.832013	NAD(P)H dehydrogenase [quinone] 1	NQO1_HUMAN
	2.865	0.5731	0.827054	Serine/threonine-protein kinase Chk1	CHK1_HUMAN
	2.904	0.4149	0.803339	3-phosphoinositide-dependent protein kinase 1	PDPK1_HUMAN
	2.892	0.4819	0.801156	Peroxisome proliferator-activated	PPARA_HU

				receptor alpha	MAN
	2.944	0.4206	0.800629	Mitogen-activated protein kinase 1	MK01_HUMAN
**Cycloeucalenol**	3.806	0.4757	2.79645	Caspase-3	CASP3_HUMAN
	3.845	0.5493	2.60713	Hepatocyte nuclear factor 4-gamma	Q14541
	3.414	0.8534	2.41308	Vitamin D-binding protein	VTDB_HUMAN
	3.423	0.4891	2.28964	Protein farnesyltransferase/geranylgeranyltransferase type-1 subunit alpha	FNTA_HUMAN
	3.873	0.5533	2.18227	Fatty acid-binding protein, brain	FABP7_HUMAN
	3.727	0.5324	2.04541	Gastrotropin	FABP6_HUMAN
	2.961	0.9871	1.8733	Integrin alpha-L	ITAL_HUMAN
	3.221	0.6442	1.84893	Calmodulin	CALM_HUMAN
	3.154	0.3942	1.72109	Heat shock protein HSP 90-beta	P08238
	2.998	0.5996	1.68401	Glutathione S-transferase P	GSTP1_HUMAN
	2.932	0.7331	1.67713	Phenylethanolamine N-methyltransferase	PNMT_HUMAN
	3.563	0.4453	1.65674	Heat shock protein HSP 90-alpha	HS90A_HUMAN
	3.564	0.2742	1.65603	Endothelial protein C receptor	EPCR_HUMAN
	2.843	0.9478	1.63792	Peptidyl-prolyl cis-trans isomerase FKBP1A	FKB1A_HUMAN
	3.652	0.4565	1.62422	Histamine N-methyltransferase	P50135
	3.164	0.5273	1.58851	Nuclear receptor subfamily 1 group I member 3	NR1I3_HUMAN
	2.927	0.7317	1.58821	Troponin C, slow skeletal and cardiac muscles	P63316
	2.862	0.954	1.58807	Caspase-7	CASP7_HUMAN
	2.974	0.4957	1.52767	Histo-blood group ABO system transferase	P16442
	3.141	0.4488	1.48603	72 kDa type IV collagenase	MMP2_HU

					MAN
	3.504	0.438	1.47662	Retinoic acid receptor RXR-alpha	RXRA_HUMAN
	3.062	0.4375	1.43456	Protein S100-A9	S10A9_HUMAN
	2.916	0.7291	1.33396	Progesterone receptor	PRGR_HUMAN
	3.231	0.359	1.29776	Serine/threonine-protein phosphatase PP1-gamma catalytic subunit	P36873
	2.952	0.5904	1.26507	Bile acid receptor	NR1H4_HUMAN
	2.913	0.4855	1.23145	Insulin-like growth factor IA	P01343
	2.745	0.6862	1.21944	Heat shock cognate 71 kDa protein	P11142
	3.132	0.5221	1.21292	Trafficking protein particle complex subunit 3	O43617
	2.734	0.9112	1.15668	Serum albumin	ALBU_HUMAN
	2.77	0.6925	1.14164	Coagulation factor X	FA10_HUMAN
	3.126	0.4466	1.12454	Lanosterol synthase	ERG7_HUMAN
	3.625	0.3625	1.08222	Nuclear receptor subfamily 1 group I member 2	NR1I2_HUMAN
	3.19	0.5317	1.0798	Alcohol dehydrogenase class-3	ADHX_HUMAN
	2.832	0.4719	1.06694	Renin	RENI_HUMAN
	3.589	0.3988	1.03501	Dual specificity mitogen-activated protein kinase kinase 1	MP2K1_HUMAN
	2.956	0.4927	0.979595	Estrogen-related receptor gamma	ERR3_HUMAN
	2.722	0.3889	0.948941	Medium-chain specific acyl-CoA dehydrogenase, mitochondrial	ACADM_HUMAN
	2.931	0.5862	0.941044	Prothrombin	THRB_HUMAN
	3.005	0.4293	0.93745	Retinoic acid receptor gamma	RARG_HUMAN
	2.716	0.5431	0.931372	Cell division protein kinase 2	P24941
	3.141	0.3926	0.929767	Ferrochelatase, mitochondrial	P22830

	2.998	0.5997	0.874966	SEC14-like protein 2	O76054
	2.864	0.2603	0.870251	FK506-binding protein 3	FKBP3_HUMAN
	3.178	0.3973	0.870223	Phospholipase A2, membrane associated	P14555
	3.237	0.4624	0.87009	Fatty acid-binding protein, heart	FABPH_HUMAN
	2.715	0.6787	0.868726	Dihydroorotate dehydrogenase, mitochondrial	PYRD_HUMAN
	3.011	0.4301	0.809252	Cytochrome P450 2C8	CP2C8_HUMAN
	2.647	0.8824	0.80723	Transthyretin	TTHY_HUMAN
	2.83	0.566	0.805289	Sex hormone-binding globulin	SHBG_HUMAN
	2.845	0.4741	0.804296	Flavin reductase	P30043
**Denudatin B**	4.43	0.5538	2.92413	Glutathione S-transferase A1	P08263
	3.932	0.6553	2.7131	Oxysterols receptor LXR-alpha	NR1H3_HUMAN
	3.753	0.7505	2.54761	Catenin alpha-1	CTNA1_HUMAN
	3.796	0.6326	2.40038	Cytochrome P450 2C9	CP2C9_HUMAN
	3.571	0.8927	2.38959	cAMP-specific 3,5-cyclic phosphodiesterase 4B	PDE4B_HUMAN
	3.903	0.4878	2.3141	Vitamin D3 receptor	VDR_HUMAN
	3.629	0.9073	2.30104	Estrogen-related receptor gamma	P62508
	3.584	0.3982	2.29901	Peptidyl-prolyl cis-trans isomerase FKBP1B	FKB1B_HUMAN
	3.598	0.514	2.22197	Histo-blood group ABO system transferase	P16442
	3.555	0.711	2.16199	Serine/threonine-protein kinase 6	O14965
	3.717	0.4647	2.15551	Heat shock protein HSP 90-beta	P08238
	3.866	0.5523	2.13035	Insulin-like growth factor IA	P01343
	3.96	0.66	2.09735	Glutathione-requiring prostaglandin D synthase	PTGD2_HUMAN
	3.709	0.7418	2.05261	Sorbitol dehydrogenase	DHSO_HUMAN
	3.839	0.6399	1.95883	Trafficking protein particle complex subunit 3	O43617

	3.685	0.9212	1.94614	Vitamin D-binding protein	VTDB_HUMAN
	4.564	0.5071	1.82914	Cellular retinoic acid-binding protein 2	P29373
	3.499	0.4999	1.78443	Dual specificity protein kinase CLK1	CLK1_HUMAN
	3.576	0.7152	1.77814	Estradiol 17-beta-dehydrogenase 1	P14061
	3.84	0.3491	1.70111	Caspase-1	CASP1_HUMAN
	3.508	0.5846	1.66228	Alpha-1-antitrypsin	A1AT_HUMAN
	3.765	0.5379	1.66087	Cytochrome P450 2C8	CP2C8_HUMAN
	3.52	0.5866	1.61505	Oxysterols receptor LXR-beta	NR1H2_HUMAN
	3.788	0.5411	1.60723	Nuclear receptor subfamily 1 group I member 2	NR1I2_HUMAN
	3.725	0.4139	1.51572	Leukotriene A-4 hydrolase	LKHA4_HUMAN
	3.825	0.4782	1.50867	Ferrochelatase, mitochondrial	P22830
	4.089	0.5111	1.50569	Glucocorticoid receptor	GCR_HUMAN
	3.577	0.5111	1.49032	Interleukin-2	IL2_HUMAN
	3.749	0.5356	1.45909	Thyroid hormone receptor alpha	P10827
	3.247	0.8119	1.4391	Retinoic acid receptor RXR-alpha	RXRA_HUMAN
	3.865	0.5521	1.40904	Sulfotransferase family cytosolic 2B member 1	O00204
	3.237	0.8092	1.3345	Sex hormone-binding globulin	SHBG_HUMAN
	3.528	0.5879	1.31776	Tyrosine-protein kinase ITK/TSK	Q08881
	3.76	0.5371	1.27571	Fatty acid-binding protein, heart	FABPH_HUMAN
	3.31	0.331	1.20234	Hydroxyacyl-coenzyme A dehydrogenase, mitochondrial	Q16836
	3.257	0.8142	1.19291	Mitogen-activated protein kinase 14	Q16539
	3.318	0.6636	1.18801	Poly [ADP-ribose] polymerase 1	P09874

	3.654	0.522	1.12006	Estrogen sulfotransferase	ST1E1_HUMAN
	3.51	0.4388	1.10607	Neprilysin	NEP_HUMAN
	3.626	0.4533	1.09738	Retinoic acid receptor alpha	RARA_HUMAN
	3.778	0.5397	1.08819	Fatty acid-binding protein, brain	FABP7_HUMAN
	3.68	0.5257	1.06614	Gastrotropin	FABP6_HUMAN
	3.594	0.5989	1.06201	Alcohol dehydrogenase class-3	ADHX_HUMAN
	3.342	0.557	1.06104	Peroxisome proliferator-activated receptor alpha	PPARA_HUMAN
	3.778	0.6296	1.04648	Phosphoenolpyruvate carboxykinase, cytosolic [GTP]	P35558
	3.802	0.4752	1.02167	Protein-glutamine gamma-glutamyltransferase E	TGM3_HUMAN
	3.984	0.4426	0.99517	Dual specificity mitogen-activated protein kinase kinase 1	MP2K1_HUMAN
	3.272	0.5454	0.97807	Leukocyte elastase	ELNE_HUMAN
	3.367	0.3741	0.970792	Glutathione S-transferase Mu 1	GSTM1_HUMAN
	3.524	0.3916	0.943358	Glutathione S-transferase A3	Q16772
**Ethyl linolenate**	4.099	0.6831	2.73197	Insulin-like growth factor IA	P01343
	4.049	0.6749	2.2901	Peroxisome proliferator-activated receptor alpha	PPARA_HUMAN
	4.355	0.6222	2.26794	Lanosterol synthase	ERG7_HUMAN
	4.49	0.3454	1.96144	Endothelial protein C receptor	EPCR_HUMAN
	3.842	0.7685	1.94413	Estrogen receptor	ESR1_HUMAN
	4.552	0.6504	1.92855	Fatty acid-binding protein, brain	FABP7_HUMAN
	3.81	0.762	1.80818	Tyrosine-protein kinase ITK/TSK	Q08881
	3.841	0.6402	1.79201	Flavin reductase	P30043
	3.879	0.431	1.77769	Phosphatidylinositol 3-kinase regulatory subunit alpha	P85A_HUMAN

	4.072	0.6786	1.75172	Trafficking protein particle complex subunit 3	O43617
	4.189	0.6981	1.71712	Alcohol dehydrogenase class-3	ADHX_HUMAN
	3.666	0.9165	1.70367	Mitogen-activated protein kinase 14	Q16539
	4.027	0.8054	1.67133	SEC14-like protein 2	O76054
	3.628	0.9071	1.66482	Nuclear receptor ROR-alpha	RORA_HUMAN
	4.535	0.5039	1.63631	Retinoic acid receptor beta	RARB_HUMAN
	4.066	0.5809	1.62715	Cytochrome P450 2C8	CP2C8_HUMAN
	3.696	0.7392	1.60356	cAMP-dependent protein kinase catalytic subunit alpha	P00517
	3.912	0.5588	1.59176	Interleukin-2	IL2_HUMAN
	3.84	0.64	1.52261	Tyrosine-protein kinase JAK3	JAK3_HUMAN
	4.281	0.6115	1.51064	Fatty acid-binding protein, heart	FABPH_HUMAN
	3.92	0.56	1.48633	Retinoic acid receptor gamma	RARG_HUMAN
	3.615	0.6025	1.47703	Ganglioside GM2 activator	P17900
	4.407	0.4006	1.47603	Phosphatidylcholine transfer protein	Q9UKL6
	3.754	0.4693	1.47393	Heat shock protein HSP 90-beta	P08238
	3.822	0.9554	1.46975	Vitamin D-binding protein	VTDB_HUMAN
	3.819	0.7638	1.46901	Mineralocorticoid receptor	MCR_HUMAN
	4.075	0.5821	1.40666	Hepatocyte nuclear factor 4-gamma	Q14541
	3.798	0.5426	1.40023	72 kDa type IV collagenase	MMP2_HUMAN
	4.672	0.5191	1.38093	Cellular retinoic acid-binding protein 2	P29373
	3.761	0.5373	1.35657	Proactivator polypeptide	P07602
	3.485	0.8712	1.35049	Peroxisome proliferator-activated receptor delta	Q03181
	3.922	0.7844	1.34752	Estradiol 17-beta-dehydrogenase	P14061

				1	
	3.49	0.698	1.30621	Estrogen receptor beta	Q92731
	3.833	0.6389	1.30035	Hepatocyte growth factor receptor	MET_HUMAN
	3.845	0.5493	1.29253	Tyrosine-protein kinase ZAP-70	ZAP70_HUMAN
	4.523	0.5025	1.27072	Dual specificity mitogen-activated protein kinase kinase 1	MP2K1_HUMAN
	3.825	0.6376	1.2564	Glutathione S-transferase A1	P08263
	3.757	0.6262	1.25589	Integrin alpha-L	ITAL_HUMAN
	3.758	0.7516	1.24687	Corticosteroid 11-beta-dehydrogenase isozyme 1	DHI1_HUMAN
	3.776	0.6294	1.23261	cAMP-specific 3,5-cyclic phosphodiesterase 4D	PDE4D_HUMAN
	3.704	0.7408	1.2084	Tyrosine-protein phosphatase non-receptor type 11	PTN11_HUMAN
	3.631	0.7262	1.19953	Alpha-tocopherol transfer protein	P49638
	3.519	0.7039	1.16705	Tyrosine-protein kinase SYK	KSYK_HUMAN
	3.622	0.6037	1.16324	Cytochrome P450 2C9	CP2C9_HUMAN
	3.833	0.5476	1.12867	Protein kinase C theta type	KPCT_HUMAN
	3.625	0.4531	1.12366	Vitamin D3 receptor	VDR_HUMAN
	3.468	0.6936	1.11422	E3 ubiquitin-protein ligase Mdm2	MDM2_HUMAN
	3.905	0.5578	1.11416	Thyroid hormone receptor alpha	P10827
	3.558	0.7117	1.10086	Bile acid receptor	NR1H4_HUMAN
	3.875	0.4305	1.09107	Proto-oncogene tyrosine-protein kinase ABL1	P00519
	3.587	0.3261	1.07293	FK506-binding protein 3	FKBP3_HUMAN
	3.913	0.4891	1.06912	Ferrochelatase, mitochondrial	P22830
	3.671	0.6119	1.04868	Retinol-binding protein 4	RET4_HUMAN
	3.896	0.4871	1.04256	Retinoic acid receptor alpha	RARA_HUMAN
	4.158	0.5198	1.02639	Glucocorticoid receptor	GCR_HUM

					AN
	3.616	0.7233	1.00233	Calmodulin	CALM_HUMAN
	4.39	0.439	1.00224	Retinoic acid receptor RXR-beta	RXRB_HUMAN
	3.511	0.5852	0.991203	Alpha-1-antitrypsin	A1AT_HUMAN
	3.616	0.5165	0.948807	Neutrophil gelatinase-associated lipocalin	NGAL_HUMAN
	3.765	0.6274	0.939705	Adenosine kinase	P55263
	3.69	0.5272	0.938884	Serine--pyruvate aminotransferase	SPYA_HUMAN
	3.475	0.6949	0.936721	Poly [ADP-ribose] polymerase 1	P09874
	3.768	0.4187	0.894738	Serine/threonine-protein phosphatase PP1-gamma catalytic subunit	P36873
	3.595	0.719	0.851367	Wiskott-Aldrich syndrome protein	WASP_HUMAN
	3.397	0.8493	0.840306	ADAM 17	ADA17_HUMAN
	3.853	0.5504	0.825037	Gastrotropin	FABP6_HUMAN
**Ethyl oleate (NF)**	4.665	0.6664	2.95052	Cytochrome P450 2C8	CP2C8_HUMAN
	4.492	0.7487	2.71594	Trafficking protein particle complex subunit 3	O43617
	4.456	0.7426	2.33543	Alcohol dehydrogenase class-3	ADHX_HUMAN
	4.734	0.6763	2.28966	Fatty acid-binding protein, brain	FABP7_HUMAN
	3.87	0.9676	2.02051	ADAM 17	ADA17_HUMAN
	4.28	0.3891	2.00662	Caspase-1	CASP1_HUMAN
	4.153	0.8307	2.00605	SEC14-like protein 2	O76054
	4.232	0.7053	1.99455	Glutathione-requiring prostaglandin D synthase	PTGD2_HUMAN
	3.782	0.7564	1.98304	Corticosteroid 11-beta-dehydrogenase isozyme 1	P28845
	4.471	0.3439	1.95979	Endothelial protein C receptor	EPCR_HUMAN

	3.674	0.9186	1.89642	Nuclear receptor ROR-alpha	RORA_HUMAN
	3.896	0.487	1.83666	Vitamin D3 receptor	VDR_HUMAN
	5.159	0.6448	1.80021	Retinol-binding protein 4	RET4_HUMAN
	3.703	0.4629	1.72861	Heme oxygenase 1	HMOX1_HUMAN
	4.142	0.5916	1.68171	Thyroid hormone receptor alpha	P10827
	3.654	0.9136	1.67146	Progesterone receptor	PRGR_HUMAN
	4.189	0.5236	1.6232	Ferrochelatase, mitochondrial	P22830
	3.867	0.5525	1.60961	72 kDa type IV collagenase	MMP2_HUMAN
	3.695	0.739	1.59986	Vascular endothelial growth factor receptor 2	P35968
	3.816	0.7632	1.58735	Tyrosine-protein phosphatase non-receptor type 11	PTN11_HUMAN
	4.127	0.5896	1.56317	Hepatocyte nuclear factor 4-gamma	Q14541
	3.746	0.6243	1.54467	Peroxisome proliferator-activated receptor alpha	PPARA_HUMAN
	3.686	0.7372	1.53715	Bile acid receptor	NR1H4_HUMAN
	3.658	0.7316	1.50973	Aldo-keto reductase family 1 member C1	Q04828
	4.092	0.5115	1.48813	Retinoic acid receptor alpha	RARA_HUMAN
	3.882	0.6469	1.48704	Hepatocyte growth factor receptor	MET_HUMAN
	3.956	0.5651	1.47175	Lanosterol synthase	ERG7_HUMAN
	3.83	0.5471	1.47123	Interleukin-2	IL2_HUMAN
	3.688	0.7377	1.45221	Sex hormone-binding globulin	SHBG_HUMAN
	3.833	0.4791	1.44411	Betaine--homocysteine S-methyltransferase 1	Q93088
	3.501	0.8752	1.39557	Coagulation factor X	FA10_HUMAN
	3.585	0.717	1.38526	cAMP-dependent protein kinase	P00517

				catalytic subunit alpha	
	3.558	0.7116	1.38033	Steroid hormone receptor ERR1	P11474
	3.773	0.6289	1.36754	Integrin alpha-L	ITAL_HUMAN
	3.659	0.6098	1.36484	Flavin reductase	P30043
	3.531	0.5885	1.33688	Ganglioside GM2 activator	P17900
	5.857	0.5857	1.33198	Cellular retinoic acid-binding protein 2	P29373
	3.605	0.6009	1.29724	Alpha-1-antitrypsin	A1AT_HUMAN
	3.793	0.5419	1.2636	Retinoic acid receptor gamma	RARG_HUMAN
	4.064	0.5806	1.25692	Gastrotropin	FABP6_HUMAN
	4.253	0.3866	1.24714	Phosphatidylcholine transfer protein	Q9UKL6
	3.709	0.9273	1.23736	Vitamin D-binding protein	VTDB_HUMAN
	3.679	0.6132	1.18804	Tyrosine-protein kinase JAK3	JAK3_HUMAN
	3.551	0.5918	1.14713	Leukocyte elastase	ELNE_HUMAN
	3.604	0.7207	1.0827	Sorbitol dehydrogenase	DHSO_HUMAN
	3.427	0.8567	1.0801	Mitogen-activated protein kinase 14	Q16539
	3.824	0.5462	1.05777	Nuclear receptor subfamily 1 group I member 2	NR1I2_HUMAN
	3.712	0.6186	1.04924	Tyrosine-protein kinase SYK	KSYK_HUMAN
	3.396	0.5659	1.04482	cGMP-specific 3,5-cyclic phosphodiesterase	PDE5A_HUMAN
	3.827	0.4253	1.04411	Growth factor receptor-bound protein 2	GRB2_HUMAN
	3.572	0.3969	1.03269	Phosphatidylinositol 3-kinase regulatory subunit alpha	P85A_HUMAN
	3.668	0.6113	1.03242	Tyrosine-protein kinase ITK/TSK	Q08881
	3.688	0.5269	1.00802	Tyrosine-protein kinase ZAP-70	ZAP70_HUMAN
	3.325	0.665	0.991656	Estrogen receptor beta	Q92731

	3.735	0.5336	0.990519	Tyrosine-protein kinase JAK2	JAK2_HUMAN
	4.299	0.4777	0.978797	Dual specificity mitogen-activated protein kinase kinase 1	MP2K1_HUMAN
	3.798	0.5425	0.96656	Tyrosine-protein kinase HCK	HCK_HUMAN
	3.711	0.5301	0.938114	Protein kinase C theta type	KPCT_HUMAN
	3.656	0.5223	0.933626	Serine--pyruvate aminotransferase	SPYA_HUMAN
	4.317	0.4317	0.929643	Retinoic acid receptor RXR-beta	RXRB_HUMAN
	3.59	0.718	0.924051	Wiskott-Aldrich syndrome protein	WASP_HUMAN
	3.42	0.5699	0.898297	Insulin-like growth factor IA	P01343
	3.868	0.5525	0.881978	Sulfotransferase family cytosolic 2B member 1	O00204
	3.625	0.6042	0.861503	Glutathione S-transferase A1	P08263
	4.026	0.4473	0.85726	Retinoic acid receptor beta	RARB_HUMAN
	3.526	0.7052	0.851513	Calmodulin	CALM_HUMAN
	3.482	0.5803	0.839282	Receptor tyrosine-protein kinase erbB-4	ERBB4_HUMAN
	3.518	0.5863	0.827023	Oxysterols receptor LXR-alpha	NR1H3_HUMAN
	3.935	0.6558	0.819932	Phosphoenolpyruvate carboxykinase, cytosolic [GTP]	P35558
**Fucosterol**	4.563	0.9125	2.80135	SEC14-like protein 2	O76054
	4.634	0.9268	2.7953	Estradiol 17-beta-dehydrogenase 1	P14061
	4.499	0.7498	2.78733	Glutathione S-transferase A1	P08263
	3.892	0.7783	2.42625	Histone deacetylase 8	Q9BY41
	3.822	0.9556	2.27279	Progesterone receptor	PRGR_HUMAN
	3.721	0.9303	2.15721	Nuclear receptor ROR-alpha	RORA_HUMAN
	3.78	0.9451	2.15134	Mineralocorticoid receptor	MCR_HUMAN
	4.165	0.6941	1.99454	Trafficking protein particle complex subunit 3	O43617

	4.48	0.64	1.87187	Sulfotransferase family cytosolic 2B member 1	O00204
	3.757	0.7515	1.83028	Bile acid receptor	NR1H4_HUMAN
	5.305	0.6632	1.82133	Retinol-binding protein 4	RET4_HUMAN
	3.69	0.7381	1.79763	cAMP-dependent protein kinase catalytic subunit alpha	P00517
	4.134	0.689	1.79041	Glutathione-requiring prostaglandin D synthase	PTGD2_HUMAN
	4.251	0.6073	1.78356	Hepatocyte nuclear factor 4-gamma	Q14541
	4.446	0.741	1.76457	Sex hormone-binding globulin	SHBG_HUMAN
	3.809	0.7619	1.75308	Prothrombin	THRB_HUMAN
	3.599	0.8998	1.71261	Mitogen-activated protein kinase 14	Q16539
	3.808	0.7616	1.68238	Calmodulin	CALM_HUMAN
	3.722	0.7445	1.62985	Androgen receptor	ANDR_HUMAN
	4.239	0.5299	1.61035	Retinoic acid receptor gamma	RARG_HUMAN
	4.181	0.5226	1.59436	Ferrochelatase, mitochondrial	P22830
	3.731	0.4145	1.56658	Phosphatidylinositol 3-kinase regulatory subunit alpha	P85A_HUMAN
	3.981	0.5687	1.52967	Cytochrome P450 2C8	CP2C8_HUMAN
	3.792	0.7585	1.49312	Corticosteroid 11-beta-dehydrogenase isozyme 1	DHI1_HUMAN
	3.508	0.8771	1.46313	Collagenase 3	MMP13_HUMAN
	3.706	0.6176	1.42273	Oxysterols receptor LXR-alpha	NR1H3_HUMAN
	3.71	0.742	1.42054	Tyrosine-protein phosphatase non-receptor type 11	PTN11_HUMAN
	3.911	0.6518	1.38603	Adenosine kinase	P55263
	3.612	0.6019	1.3827	Flavin reductase	P30043
	3.929	0.4912	1.36666	Caspase-3	CASP3_HUMAN

	3.529	0.7058	1.34419	Aldo-keto reductase family 1 member C1	Q04828
	4.315	0.4315	1.33911	Vitamin D3 receptor	VDR_HUMAN
	4.104	0.5862	1.32976	Gastrotropin	FABP6_HUMAN
	3.527	0.7054	1.31376	Serine/threonine-protein kinase Chk1	CHK1_HUMAN
	3.664	0.5234	1.2809	72 kDa type IV collagenase	MMP2_HUMAN
	4.079	0.3138	1.22992	Endothelial protein C receptor	EPCR_HUMAN
	3.557	0.4446	1.21096	Heat shock protein HSP 90-beta	P08238
	3.648	0.608	1.176	Oxysterols receptor LXR-beta	NR1H2_HUMAN
	3.554	0.5923	1.17138	Receptor tyrosine-protein kinase erbB-4	ERBB4_HUMAN
	3.657	0.5224	1.15494	Protein farnesyltransferase/geranylgeranyltransferase type-1 subunit alpha	FNTA_HUMAN
	4.452	0.4946	1.14288	Dual specificity mitogen-activated protein kinase kinase 1	MP2K1_HUMAN
	3.302	0.8254	1.13966	Bile salt sulfotransferase	Q06520
	3.601	0.6001	1.11438	Ephrin type-B receptor 4	EPHB4_HUMAN
	3.559	0.5084	0.998922	Interleukin-2	IL2_HUMAN
	3.807	0.4759	0.986142	Retinoic acid receptor alpha	RARA_HUMAN
	3.321	0.6642	0.982093	Glutathione S-transferase P	GSTP1_HUMAN
	3.786	0.6311	0.981895	Alcohol dehydrogenase class-3	ADHX_HUMAN
	3.653	0.4567	0.978732	Proto-oncogene tyrosine-protein kinase ABL1	P00519
	5.504	0.5504	0.933987	Cellular retinoic acid-binding protein 2	P29373
	3.6	0.6	0.916695	Tyrosine-protein kinase SYK	KSYK_HUMAN
	3.526	0.8814	0.902583	Vitamin D-binding protein	VTDB_HUMAN

	3.952	0.5646	0.867685	Lactoylglutathione lyase	LGUL_HUMAN
	3.412	0.5686	0.857526	NAD(P)H dehydrogenase [quinone] 1	NQO1_HUMAN
	3.311	0.8279	0.842497	Estrogen receptor	ESR1_HUMAN
	3.329	0.5548	0.840394	Alpha-1-antitrypsin	A1AT_HUMAN
	4.257	0.4257	0.825894	Retinoic acid receptor RXR-beta	RXRB_HUMAN
	3.362	0.6725	0.814543	Alpha-tocopherol transfer protein	P49638
**Kadsurenone**	4.517	0.5647	3.81561	Vitamin D3 receptor	VDR_HUMAN
	4.468	0.4062	3.23504	Caspase-1	CASP1_HUMAN
	3.822	0.546	2.82912	Histo-blood group ABO system transferase	P16442
	3.982	0.7964	2.58248	Corticosteroid 11-beta-dehydrogenase isozyme 1	DHI1_HUMAN
	3.636	0.909	2.46722	Sex hormone-binding globulin	SHBG_HUMAN
	4.128	0.688	2.40025	Glutathione-requiring prostaglandin D synthase	PTGD2_HUMAN
	3.776	0.6294	2.22691	Cytochrome P450 2C9	CP2C9_HUMAN
	3.669	0.7338	2.10364	Aldo-keto reductase family 1 member C1	Q04828
	3.553	0.8882	2.01445	Mitogen-activated protein kinase 14	Q16539
	3.512	0.8779	1.98878	Insulin-like growth factor 1 receptor	P08069
	3.701	0.7401	1.90972	Sorbitol dehydrogenase	DHSO_HUMAN
	3.82	0.5456	1.89891	Insulin-like growth factor IA	P01343
	3.629	0.5185	1.84804	Deoxycytidine kinase	DCK_HUMAN
	3.854	0.4282	1.84788	Neutrophil gelatinase-associated lipocalin	NGAL_HUMAN
	3.68	0.6133	1.83683	Oxysterols receptor LXR-alpha	NR1H3_HUMAN
	3.594	0.5134	1.76154	cAMP-dependent protein kinase,	P00517

				alpha-catalytic subunit	
	3.772	0.4715	1.72721	Neprilysin	NEP_HUMAN
	3.781	0.6302	1.71057	Trafficking protein particle complex subunit 3	O43617
	3.842	0.7683	1.69173	Bile salt sulfotransferase	Q06520
	3.55	0.5917	1.67749	Leukocyte elastase	ELNE_HUMAN
	3.873	0.5533	1.66677	Thyroid hormone receptor alpha	P10827
	3.682	0.5259	1.65488	Interleukin-2	IL2_HUMAN
	3.933	0.5619	1.63877	Estrogen sulfotransferase	ST1E1_HUMAN
	3.408	0.8519	1.51526	Collagenase 3	MMP13_HUMAN
	3.649	0.5213	1.44528	Retinoic acid receptor gamma	RARG_HUMAN
	3.47	0.5784	1.36346	Oxysterols receptor LXR-beta	NR1H2_HUMAN
	3.372	0.6745	1.35198	Catenin alpha-1	CTNA1_HUMAN
	3.304	0.8261	1.34586	Estrogen receptor	ESR1_HUMAN
	3.718	0.5312	1.33403	Nuclear receptor subfamily 1 group I member 2	NR1I2_HUMAN
	3.56	0.5933	1.22033	Hepatocyte growth factor receptor	MET_HUMAN
	3.259	0.8148	1.21907	Mineralocorticoid receptor	MCR_HUMAN
	3.336	0.8341	1.1848	ADAM 17	ADA17_HUMAN
	3.268	0.8169	1.17304	cAMP-specific 3,5-cyclic phosphodiesterase 4B	PDE4B_HUMAN
	3.479	0.5799	1.13376	Retinol-binding protein 4	RET4_HUMAN
	3.455	0.5759	1.10575	Phosphoenolpyruvate carboxykinase, cytosolic [GTP]	P35558
	3.843	0.4804	1.08108	Histamine N-methyltransferase	P50135
	3.379	0.4224	1.07072	Heat shock protein HSP 90-beta	P08238
	3.633	0.4541	1.05566	Glucose-6-phosphate isomerase	G6PI_HUMAN

	3.314	0.5523	1.05185	Serine hydroxymethyltransferase, cytosolic	GLYC_HUMAN
	3.451	0.5752	1.0474	Triosephosphate isomerase	TPIS_HUMAN
	3.358	0.6716	1.04266	Alpha-tocopherol transfer protein	P49638
	4.048	0.4498	1.03537	Dual specificity mitogen-activated protein kinase kinase 1	MP2K1_HUMAN
	4.996	0.4996	1.0053	Cellular retinoic acid-binding protein 2	P29373
	3.56	0.3956	1.00196	Nitric oxide synthase, inducible	NOS2_HUMAN
	3.547	0.5912	0.985885	Adenosine kinase	P55263
	3.633	0.3633	0.963671	Glutathione S-transferase Mu 1	GSTM1_HUMAN
	3.224	0.6448	0.950866	Macrophage metalloelastase	MMP12_HUMAN
	4.027	0.4027	0.926269	Retinoic acid receptor RXR-beta	RXRB_HUMAN
	3.45	0.3833	0.924547	E-selectin	LYAM2_HUMAN
	3.417	0.4271	0.917453	Chymase	CMA1_HUMAN
	3.411	0.4264	0.910241	Betaine--homocysteine S-methyltransferase 1	Q93088
	3.571	0.4464	0.87403	Retinoic acid receptor alpha	RARA_HUMAN
	3.612	0.4515	0.847344	Glucokinase	HXK4_HUMAN
	3.183	0.3978	0.826918	Heme oxygenase 1	HMOX1_HUMAN
	3.425	0.6851	0.816335	SEC14-like protein 2	O76054
	3.534	0.5891	0.80976	Alcohol dehydrogenase class-3	ADHX_HUMAN
**Karanjin**	4.027	0.5753	4.09216	Tyrosine-protein kinase HCK	HCK_HUMAN
	3.654	0.4567	3.81185	Coagulation factor XI	FA11_HUMAN
	3.446	0.6892	3.80512	Carbonyl reductase [NADPH] 1	CBR1_HUMAN
	3.596	0.5994	3.27056	Proto-oncogene tyrosine-protein kinase LCK	LCK_HUMAN

	3.467	0.4333	2.7748	Betaine--homocysteine S-methyltransferase 1	Q93088
	3.154	0.6307	2.38835	Glycogen synthase kinase-3 beta	P49841
	3.496	0.3885	2.03511	5(3)-deoxyribonucleotidase, mitochondrial	Q9NPB1
	3.377	0.4221	1.97482	Glucose-6-phosphate isomerase	G6PI_HUMAN
	3.554	0.5077	1.95661	Cystathionine beta-synthase	P35520
	2.968	0.4946	1.89318	Fatty acid-binding protein, adipocyte	FABP4_HUMAN
	3.117	0.5196	1.85843	Dihydroorotate dehydrogenase, mitochondrial	PYRD_HUMAN
	3.566	0.4457	1.85572	Protein-glutamine gamma-glutamyltransferase E	TGM3_HUMAN
	3.551	0.3551	1.65401	3 histone mRNA exonuclease 1	Q8IV48
	2.916	0.5832	1.65145	Heat shock protein HSP 90-alpha	HS90A_HUMAN
	2.934	0.7334	1.61618	cAMP-specific 3,5-cyclic phosphodiesterase 4D	PDE4D_HUMAN
	2.946	0.7365	1.61394	Mitogen-activated protein kinase 14	Q16539
	2.912	0.728	1.58973	B-Raf proto-oncogene serine/threonine-protein kinase	BRAF1_HUMAN
	2.943	0.7359	1.54589	Protein farnesyltransferase/geranylgeranyltransferase type-1 subunit alpha	FNTA_HUMAN
	2.865	0.9551	1.50248	Glutathione S-transferase P	GSTP1_HUMAN
	2.89	0.7226	1.49786	Cyclin-A2	CCNA2_HUMAN
	2.96	0.4934	1.48098	Thymidylate synthase	TYSY_HUMAN
	2.868	0.5735	1.46582	Cyclin-T1	CCNT1_HUMAN
	2.888	0.7219	1.45527	Macrophage migration inhibitory factor	MIF_HUMAN
	2.851	0.9503	1.42776	Mitogen-activated protein kinase 10	MK10_HUMAN
	3.204	0.4577	1.4244	Nuclear receptor subfamily 1 group I member 2	NR1I2_HUMAN
	2.85	0.9501	1.40814	Carbonic anhydrase 2	CAH2_HU

					MAN
	2.891	0.5782	1.40777	Methionine aminopeptidase 2	AMPM2_HUMAN
	2.892	0.7231	1.39078	Mineralocorticoid receptor	MCR_HUMAN
	2.846	0.9488	1.37653	Mitogen-activated protein kinase 1	MK01_HUMAN
	2.878	0.5757	1.35538	Neutrophil collagenase	MMP8_HUMAN
	2.917	0.5833	1.3115	Vascular endothelial growth factor receptor 2	P35968
	2.894	0.7236	1.30816	Transthyretin	TTHY_HUMAN
	2.831	0.9435	1.26344	Mitogen-activated protein kinase 8	MK08_HUMAN
	2.987	0.4979	1.25292	Receptor tyrosine-protein kinase erbB-4	ERBB4_HUMAN
	2.84	0.71	1.23583	Sex hormone-binding globulin	SHBG_HUMAN
	2.838	0.7096	1.22644	Peroxisome proliferator-activated receptor delta	Q03181
	2.8	0.7001	1.19084	Cathepsin B	CATB_HUMAN
	2.852	0.5703	1.17052	Cell division protein kinase 6	CDK6_HUMAN
	2.813	0.5627	1.15861	Cathepsin K	CATK_HUMAN
	2.85	0.7125	1.15779	Peroxisome proliferator-activated receptor gamma	PPARG_HUMAN
	2.8	0.9334	1.14412	Estrogen receptor	ESR1_HUMAN
	2.813	0.5627	1.13621	cAMP-dependent protein kinase catalytic subunit alpha	P00517
	2.78	0.9266	1.11937	Complement factor B	CFAB_HUMAN
	2.857	0.4761	1.11783	Coagulation factor X	FA10_HUMAN
	2.795	0.6987	1.08911	Angiogenin	ANGI_HUMAN
	2.834	0.4724	1.08678	Alpha-amylase 1	P04745
	2.867	0.7168	1.07043	Proto-oncogene tyrosine-protein	SRC_HUM

				kinase Src	AN
	2.956	0.4223	1.0451	Matrix metalloproteinase-9	MMP9_HUMAN
	2.885	0.3606	1.02069	Vitamin D3 receptor	VDR_HUMAN
	2.915	0.265	1.00576	FK506-binding protein 3	FKBP3_HUMAN
	2.832	0.5663	0.985134	Hepatocyte growth factor receptor	P08581
	2.822	0.3136	0.96644	Pancreatic alpha-amylase	AMYP_HUMAN
	2.888	0.5776	0.951895	Serine/threonine-protein kinase Chk1	CHK1_HUMAN
	2.82	0.564	0.947982	Aldo-keto reductase family 1 member C3	AK1C3_HUMAN
	2.877	0.3596	0.947498	Signal transducer and activator of transcription 1-alpha/beta	STAT1_HUMAN
	2.931	0.3663	0.934186	Cell division protein kinase 7	P50613
	2.75	0.6876	0.892215	Dipeptidyl peptidase 4	DPP4_HUMAN
	2.777	0.5553	0.888981	Phenylalanine-4-hydroxylase	PH4H_HUMAN
	2.744	0.6861	0.873378	Androgen receptor	ANDR_HUMAN
	2.786	0.6966	0.851587	ADAM 17	ADA17_HUMAN
	2.978	0.4254	0.83174	S-methyl-5-thioadenosine phosphorylase	Q13126
	2.719	0.6797	0.829469	Complement factor D	CFAD_HUMAN
	3.629	0.3629	0.824824	Neprilysin	NEP_HUMAN
	2.814	0.5628	0.800302	Corticosteroid 11-beta-dehydrogenase isozyme 1	P28845
**LAN**	4.542	0.9085	3.29745	Aldo-keto reductase family 1 member C1	Q04828
	4.937	0.7053	3.04636	Lanosterol synthase	ERG7_HUMAN
	4.431	0.6329	2.589	Renin	RENI_HUMAN
	4.641	0.663	2.51593	Cytochrome P450 2C8	CP2C8_HU

					MAN
	4.471	0.7451	2.35577	E3 ubiquitin-protein ligase Mdm2	MDM2_HUMAN
	4.455	0.8911	2.34343	SEC14-like protein 2	O76054
	3.857	0.9644	2.30149	Nuclear receptor ROR-alpha	RORA_HUMAN
	5.975	0.7469	2.2942	Retinol-binding protein 4	RET4_HUMAN
	3.905	0.6509	2.21675	Ganglioside GM2 activator	P17900
	3.872	0.7745	2.04263	cAMP-dependent protein kinase catalytic subunit alpha	P00517
	4.409	0.5511	1.945	Retinoic acid receptor gamma	RARG_HUMAN
	3.886	0.7771	1.94049	Bile acid receptor	NR1H4_HUMAN
	4.225	0.7042	1.90163	Trafficking protein particle complex subunit 3	O43617
	4.462	0.5578	1.84738	Ferrochelatase, mitochondrial	P22830
	4.256	0.532	1.79378	Caspase-3	CASP3_HUMAN
	3.9	0.9751	1.62199	Vitamin D-binding protein	VTDB_HUMAN
	4.692	0.5864	1.61792	Glucocorticoid receptor	GCR_HUMAN
	3.834	0.7667	1.61295	Prothrombin	THRB_HUMAN
	4.756	0.5945	1.5992	Tyrosine-protein phosphatase non-receptor type 1	PTN1_HUMAN
	3.846	0.7691	1.55203	Tyrosine-protein phosphatase non-receptor type 11	PTN11_HUMAN
	3.902	0.7805	1.55034	Corticosteroid 11-beta-dehydrogenase isozyme 1	DHI1_HUMAN
	3.946	0.4933	1.5182	Proto-oncogene tyrosine-protein kinase ABL1	P00519
	3.845	0.5493	1.47151	72 kDa type IV collagenase	MMP2_HUMAN
	3.794	0.7587	1.46911	Calmodulin	CALM_HUMAN
	3.776	0.7552	1.44311	Estradiol 17-beta-dehydrogenase 1	P14061
	4.122	0.687	1.42964	Alcohol dehydrogenase class-3	ADHX_HU

					MAN
	3.793	0.7586	1.40314	Mineralocorticoid receptor	MCR_HUMAN
	4.287	0.6124	1.39987	Sulfotransferase family cytosolic 2B member 1	O00204
	3.891	0.6484	1.35879	Glutathione S-transferase A1	P08263
	3.756	0.5366	1.34954	Serine/threonine-protein kinase 6	O14965
	3.768	0.6279	1.34277	Nuclear receptor subfamily 1 group I member 3	NR1I3_HUMAN
	3.529	0.7058	1.33672	Steroid hormone receptor ERR1	P11474
	4.103	0.5862	1.33625	Hepatocyte nuclear factor 4-gamma	Q14541
	3.665	0.7331	1.33441	Alpha-tocopherol transfer protein	P49638
	3.863	0.6438	1.33124	Hepatocyte growth factor receptor	MET_HUMAN
	3.693	0.6154	1.32902	Receptor tyrosine-protein kinase erbB-4	ERBB4_HUMAN
	3.551	0.5919	1.32498	Retinoic acid receptor RXR-alpha	RXRA_HUMAN
	4.207	0.601	1.32374	Gastrotropin	FABP6_HUMAN
	3.959	0.6599	1.31396	Glutathione-requiring prostaglandin D synthase	PTGD2_HUMAN
	4.726	0.5251	1.30582	Dual specificity mitogen-activated protein kinase kinase 1	MP2K1_HUMAN
	3.494	0.6988	1.30003	Glutathione S-transferase P	GSTP1_HUMAN
	3.973	0.6621	1.29491	Sex hormone-binding globulin	SHBG_HUMAN
	4.215	0.3243	1.27793	Endothelial protein C receptor	EPCR_HUMAN
	4.764	0.4764	1.26368	Retinoic acid receptor RXR-beta	RXRB_HUMAN
	4.236	0.6052	1.18511	Fatty acid-binding protein, brain	FABP7_HUMAN
	3.498	0.2691	1.17436	Amine oxidase [flavin-containing] A	AOFA_HUMAN
	4.082	0.5832	1.16241	Mitogen-activated protein kinase 10	MK10_HUMAN
	3.939	0.4376	1.16212	Growth factor receptor-bound protein 2	GRB2_HUMAN

	3.503	0.7006	1.1391	Poly [ADP-ribose] polymerase 1	P09874
	4.329	0.481	1.13614	Retinoic acid receptor beta	RARB_HUMAN
	3.645	0.6075	1.13206	Oxysterols receptor LXR-alpha	NR1H3_HUMAN
	3.543	0.3936	1.12605	Bcl-2-like protein 1	Q07817
	3.474	0.579	1.10861	Insulin-like growth factor IA	P01343
	3.681	0.5258	1.10614	Interleukin-2	IL2_HUMAN
	3.421	0.6843	1.05133	Histone deacetylase 8	Q9BY41
	3.529	0.5881	1.03374	Flavin reductase	P30043
	3.613	0.5162	1.02152	Neutrophil gelatinase-associated lipocalin	NGAL_HUMAN
	3.462	0.577	1.01223	Alpha-1-antitrypsin	A1AT_HUMAN
	4.213	0.383	0.997048	Phosphatidylcholine transfer protein	Q9UKL6
	3.341	0.4772	0.929073	3-hydroxy-3-methylglutaryl-coenzyme A reductase	HMDH_HUMAN
	3.588	0.598	0.860887	cAMP-specific 3,5-cyclic phosphodiesterase 4D	PDE4D_HUMAN
	3.743	0.7485	0.854952	Bile salt sulfotransferase	Q06520
	3.552	0.592	0.838784	Oxysterols receptor LXR-beta	NR1H2_HUMAN
	3.547	0.7095	0.835192	Wiskott-Aldrich syndrome protein	WASP_HUMAN
	3.467	0.4953	0.832579	Protein S100-A9	S10A9_HUMAN
	3.346	0.6692	0.805078	Vascular endothelial growth factor receptor 2	P35968
**lanost-8-en-3beta-ol**	4.543	0.9085	3.40766	Aldo-keto reductase family 1 member C1	Q04828
	4.931	0.7044	3.143	Lanosterol synthase	ERG7_HUMAN
	4.69	0.6699	2.70006	Cytochrome P450 2C8	CP2C8_HUMAN
	3.927	0.9816	2.56554	Nuclear receptor ROR-alpha	RORA_HUMAN
	4.454	0.8909	2.43385	SEC14-like protein 2	O76054
	4.298	0.614	2.41714	Renin	RENI_HUMAN

	4.328	0.7214	2.17376	E3 ubiquitin-protein ligase Mdm2	MDM2_HUMAN
	4.16	0.6933	2.11162	Retinol-binding protein 4	RET4_HUMAN
	3.787	0.6312	2.02914	Ganglioside GM2 activator	P17900
	4.462	0.5578	1.92402	Ferrochelatase, mitochondrial	P22830
	4.184	0.6974	1.90784	Trafficking protein particle complex subunit 3	O43617
	3.813	0.7626	1.858	Bile acid receptor	NR1H4_HUMAN
	4.215	0.5269	1.80141	Caspase-3	CASP3_HUMAN
	4.278	0.713	1.78657	Alcohol dehydrogenase class-3	ADHX_HUMAN
	3.939	0.7878	1.70702	Corticosteroid 11-beta-dehydrogenase isozyme 1	DHI1_HUMAN
	3.835	0.4261	1.70594	Phosphatidylinositol 3-kinase regulatory subunit alpha	P85A_HUMAN
	3.895	0.9737	1.69753	Vitamin D-binding protein	VTDB_HUMAN
	4.763	0.5954	1.68122	Tyrosine-protein phosphatase non-receptor type 1	PTN1_HUMAN
	4.353	0.6219	1.67105	Mitogen-activated protein kinase 10	MK10_HUMAN
	3.906	0.558	1.64506	72 kDa type IV collagenase	MMP2_HUMAN
	3.885	0.555	1.64166	Macrophage metalloelastase	MMP12_HUMAN
	3.978	0.4973	1.64087	Proto-oncogene tyrosine-protein kinase ABL1	P00519
	4.644	0.5805	1.62722	Glucocorticoid receptor	GCR_HUMAN
	3.616	0.7231	1.58102	Steroid hormone receptor ERR1	P11474
	3.706	0.6176	1.52015	Flavin reductase	P30043
	3.776	0.7552	1.51232	Estradiol 17-beta-dehydrogenase 1	P14061
	3.568	0.7137	1.48583	Histone deacetylase 8	Q9BY41
	3.531	0.8828	1.46935	Mitogen-activated protein kinase 14	Q16539
	3.886	0.6477	1.42127	Glutathione S-transferase A1	P08263
	3.759	0.7518	1.40444	Mineralocorticoid receptor	MCR_HUM

					AN
	3.733	0.6221	1.39217	Oxysterols receptor LXR-alpha	NR1H3_HUMAN
	4.207	0.601	1.39071	Gastrotropin	FABP6_HUMAN
	4.229	0.6042	1.38198	Sulfotransferase family cytosolic 2B member 1	O00204
	4.792	0.4792	1.3612	Retinoic acid receptor RXR-beta	RXRB_HUMAN
	3.734	0.6223	1.3431	Nuclear receptor subfamily 1 group I member 3	NR1I3_HUMAN
	3.476	0.6953	1.32548	Glutathione S-transferase P	GSTP1_HUMAN
	3.621	0.7243	1.30905	Alpha-tocopherol transfer protein	P49638
	3.699	0.5284	1.28812	Neutrophil gelatinase-associated lipocalin	NGAL_HUMAN
	3.804	0.6339	1.28541	Hepatocyte growth factor receptor	MET_HUMAN
	3.921	0.6534	1.27093	Sex hormone-binding globulin	SHBG_HUMAN
	4.35	0.3955	1.25781	Phosphatidylcholine transfer protein	Q9UKL6
	4.62	0.5133	1.24555	Dual specificity mitogen-activated protein kinase kinase 1	MP2K1_HUMAN
	3.513	0.7027	1.22155	Poly [ADP-ribose] polymerase 1	P09874
	3.646	0.7293	1.21588	Calmodulin	CALM_HUMAN
	4.13	0.3177	1.21311	Endothelial protein C receptor	EPCR_HUMAN
	3.696	0.528	1.19513	Interleukin-2	IL2_HUMAN
	4.303	0.4781	1.16391	Retinoic acid receptor beta	RARB_HUMAN
	3.821	0.6368	1.14335	Glutathione-requiring prostaglandin D synthase	PTGD2_HUMAN
	3.616	0.7232	1.11837	Tyrosine-protein phosphatase non-receptor type 11	PTN11_HUMAN
	3.919	0.4898	1.09609	Retinoic acid receptor alpha	RARA_HUMAN
	3.998	0.4997	1.09019	Peptidyl-prolyl cis-trans isomerase FKBP1A	FKB1A_HUMAN

	3.805	0.7611	1.03234	Bile salt sulfotransferase	Q06520
	3.515	0.5021	1.00254	Protein S100-A9	S10A9_HUMAN
	3.537	0.5053	1.00164	Serine/threonine-protein kinase 6	O14965
	3.337	0.4767	0.97617	3-hydroxy-3-methylglutaryl-coenzyme A reductase	HMDH_HUMAN
	4.08	0.51	0.961189	Histamine N-methyltransferase	P50135
	3.377	0.6753	0.927537	Vascular endothelial growth factor receptor 2	P35968
	4.01	0.5728	0.90684	Fatty acid-binding protein, brain	FABP7_HUMAN
	3.552	0.592	0.899387	Oxysterols receptor LXR-beta	NR1H2_HUMAN
	3.356	0.5593	0.853989	Alpha-1-antitrypsin	A1AT_HUMAN
**lanosta-8,24-dien-3-ol,3-acetate**	3.752	0.4169	2.96609	Serine/threonine-protein phosphatase PP1-gamma catalytic subunit	P36873
	3.834	0.4793	2.20822	Fatty acid-binding protein, brain	FABP7_HUMAN
	3.352	0.419	2.08702	Proto-oncogene tyrosine-protein kinase ABL1	P00519
	3.608	0.2775	1.93798	Endothelial protein C receptor	EPCR_HUMAN
	3.22	0.5366	1.86492	Nuclear receptor subfamily 1 group I member 3	NR1I3_HUMAN
	2.921	0.9736	1.80989	Proto-oncogene serine/threonine-protein kinase Pim-1	P11309
	3.642	0.4553	1.78216	Histamine N-methyltransferase	P50135
	2.997	0.5995	1.71758	Glutathione S-transferase P	GSTP1_HUMAN
	3.256	0.5427	1.70014	Tyrosine-protein kinase SYK	KSYK_HUMAN
	3.191	0.5318	1.6899	Integrin alpha-L	ITAL_HUMAN
	2.92	0.9735	1.6799	Apolipoprotein A-II	APOA2_HUMAN
	3.327	0.4159	1.67404	Caspase-3	CASP3_HUMAN
	3.844	0.3844	1.63642	Nuclear receptor subfamily 1 group I member 2	NR1I2_HUMAN

	2.953	0.7382	1.62648	Mitogen-activated protein kinase 8	MK08_HUMAN
	3.12	0.4458	1.58113	Proactivator polypeptide	P07602
	3.177	0.5296	1.57933	Dipeptidyl peptidase 4	DPP4_HUMAN
	3.324	0.4748	1.47485	Hepatocyte nuclear factor 4-gamma	Q14541
	3.129	0.3912	1.43595	Vitamin D3 receptor	VDR_HUMAN
	2.966	0.4943	1.42968	Insulin-like growth factor IA	P01343
	3.348	0.4783	1.38869	Mitogen-activated protein kinase 10	MK10_HUMAN
	3.07	0.4385	1.35599	72 kDa type IV collagenase	MMP2_HUMAN
	3.23	0.3589	1.34077	Growth factor receptor-bound protein 2	GRB2_HUMAN
	3.152	0.5254	1.33132	[Pyruvate dehydrogenase [lipoamide]] kinase isozyme 2, mitochondrial	PDK2_HUMAN
	2.87	0.7175	1.28288	Cell division protein kinase 2	P24941
	3.226	0.4033	1.28012	Glutathione S-transferase A1	P08263
	3.354	0.4791	1.2519	Fatty acid-binding protein, heart	FABPH_HUMAN
	2.778	0.926	1.24505	Carbonic anhydrase 2	CAH2_HUMAN
	2.951	0.4918	1.2305	Aldo-keto reductase family 1 member C3	AK1C3_HUMAN
	2.761	0.9202	1.14502	Amine oxidase [flavin-containing] B	AOFB_HUMAN
	3.572	0.3969	1.13821	Dual specificity mitogen-activated protein kinase kinase 1	MP2K1_HUMAN
	2.98	0.596	1.1374	Prothrombin	THRB_HUMAN
	2.753	0.9176	1.13381	Thyroid hormone receptor beta	P10828
	2.908	0.4846	1.07251	Poly [ADP-ribose] polymerase 1	P09874
	2.846	0.7115	1.05908	Annexin A5	ANXA5_HUMAN
	2.835	0.567	1.04644	NONE	NONE
	3.042	0.507	1.03514	Androgen receptor	ANDR_HUMAN
	2.95	0.5901	1.01651	Calmodulin	CALM_HU

					MAN
	2.973	0.5947	0.998166	Wiskott-Aldrich syndrome protein	WASP_HUMAN
	3.537	0.3537	0.995722	Retinoic acid receptor RXR-beta	RXRB_HUMAN
	2.925	0.3251	0.988087	Phosphatidylinositol 3-kinase regulatory subunit alpha	P85A_HUMAN
	2.869	0.5737	0.985361	Bile acid receptor	NR1H4_HUMAN
	2.739	0.6848	0.982272	Troponin C, slow skeletal and cardiac muscles	P63316
	2.835	0.5669	0.976191	cAMP-dependent protein kinase catalytic subunit alpha	P00517
	2.82	0.7051	0.964711	Proto-oncogene tyrosine-protein kinase Src	SRC_HUMAN
	2.817	0.4695	0.937549	Histo-blood group ABO system transferase	P16442
	2.864	0.5727	0.92392	Serine/threonine-protein kinase Chk1	CHK1_HUMAN
	2.892	0.4819	0.914901	Peroxisome proliferator-activated receptor alpha	PPARA_HUMAN
	2.928	0.488	0.884293	Tyrosine-protein kinase JAK3	JAK3_HUMAN
	2.681	0.8937	0.876517	Kinesin-like protein KIF11	KIF11_HUMAN
	3.146	0.4494	0.867844	Gastrotropin	FABP6_HUMAN
	3.578	0.5112	0.866549	3-phosphoinositide-dependent protein kinase 1	PDPK1_HUMAN
	2.774	0.3963	0.849004	3-hydroxy-3-methylglutaryl-coenzyme A reductase	HMDH_HUMAN
	2.873	0.4789	0.842933	Glycogen synthase kinase-3 beta	GSK3B_HUMAN
	3.275	0.4093	0.840451	Glucocorticoid receptor	GCR_HUMAN
	2.651	0.8836	0.812997	Serum albumin	ALBU_HUMAN
	2.899	0.5797	0.807841	Tyrosine-protein phosphatase non-receptor type 11	PTN11_HUMAN
	2.958	0.4226	0.802951	MAP kinase-activated protein kinase 2	P49137

matrine	3.552	0.7104	4.15263	Estradiol 17-beta-dehydrogenase 1	P14061
	2.968	0.4947	2.31826	Prothrombin	THRB_HUMAN
	2.899	0.7248	1.80421	Protein farnesyltransferase/geranylgeranyltransferase type-1 subunit alpha	FNTA_HUMAN
	2.894	0.7236	1.78874	Estrogen-related receptor gamma	P62508
	2.937	0.5875	1.60863	Alpha-tocopherol transfer protein	P49638
	2.995	0.4278	1.50438	Retinoic acid receptor RXR-alpha	RXRA_HUMAN
	2.997	0.4282	1.42576	Retinoic acid receptor gamma	RARG_HUMAN
	2.802	0.5604	1.36842	Carbonic anhydrase 2	CAH2_HUMAN
	2.879	0.3599	1.33379	Heat shock protein HSP 90-beta	P08238
	2.901	0.4144	1.33333	3-phosphoinositide-dependent protein kinase 1	PDPK1_HUMAN
	2.852	0.4754	1.2888	Thyroid hormone receptor beta	P10828
	2.842	0.5683	1.26878	Mitogen-activated protein kinase 14	Q16539
	2.882	0.4804	1.17495	Nuclear receptor subfamily 1 group I member 3	NR1I3_HUMAN
	2.897	0.4139	1.15201	Peroxisome proliferator-activated receptor gamma	PPARG_HUMAN
	2.877	0.3596	1.07151	Vitamin D3 receptor	VDR_HUMAN
	2.674	0.6686	1.03163	Dipeptidyl peptidase 4	DPP4_HUMAN
	2.773	0.3962	1.01161	Baculoviral IAP repeat-containing protein 4	XIAP_HUMAN
	2.929	0.4882	0.975905	Adenosine kinase	P55263
	2.687	0.5375	0.966677	Neutrophil collagenase	MMP8_HUMAN
	2.815	0.4692	0.931216	Oxysterols receptor LXR-alpha	NR1H3_HUMAN
	2.779	0.4632	0.824458	Corticosteroid 11-beta-dehydrogenase isozyme 1	DHI1_HUMAN
**sophranol**	2.88	0.2619	2.01287	Histone-lysine N-methyltransferase SETD8	SETD8_HUMAN
	3.083	0.3854	1.94076	Stromelysin-1	MMP3_HU

					MAN
	2.815	0.7037	1.86202	Mitogen-activated protein kinase 8	MK08_HUMAN
	2.803	0.5606	1.73391	Neutrophil collagenase	MMP8_HUMAN
	2.769	0.5538	1.65756	Heat shock protein HSP 90-alpha	HS90A_HUMAN
	2.81	0.5621	1.54379	Phenylalanine-4-hydroxylase	PH4H_HUMAN
	2.944	0.4205	1.43014	cAMP-specific 3,5-cyclic phosphodiesterase 4B	PDE4B_HUMAN
	2.965	0.4235	1.36631	Tyrosine-protein phosphatase non-receptor type 1	PTN1_HUMAN
	2.722	0.6806	1.34382	Dipeptidyl peptidase 4	DPP4_HUMAN
	2.786	0.398	1.30371	Proto-oncogene serine/threonine-protein kinase Pim-1	PIM1_HUMAN
	2.804	0.5609	1.26797	Cell division protein kinase 2	P24941
	2.828	0.3535	1.17544	Bile acid receptor	Q96RI1
	2.974	0.4249	1.16084	Tyrosine-protein kinase HCK	HCK_HUMAN
	2.752	0.3931	1.08608	Corticosteroid 11-beta-dehydrogenase isozyme 1	DHI1_HUMAN
	2.771	0.4618	1.03532	Cytochrome P450 2C9	CP2C9_HUMAN
	2.69	0.5381	0.896974	Fibroblast growth factor receptor 2	P21802
	2.563	0.6409	0.826476	NONE	NONE
	2.83	0.3144	0.803871	Peptidyl-prolyl cis-trans isomerase FKBP1A	FKB1A_HUMAN
**Mandenol**	4.632	0.9263	3.19019	SEC14-like protein 2	O76054
	4.584	0.764	3.03863	Trafficking protein particle complex subunit 3	O43617
	4.171	0.5958	2.32681	Interleukin-2	IL2_HUMAN
	4	0.8	2.17117	Tyrosine-protein phosphatase non-receptor type 11	PTN11_HUMAN
	4.229	0.6041	2.14019	Lanosterol synthase	ERG7_HUMAN
	3.91	0.6516	2.07653	Peroxisome proliferator-activated receptor alpha	PPARA_HUMAN

	4.462	0.3432	2.04204	Endothelial protein C receptor	EPCR_HUMAN
	3.943	0.9857	1.98574	Vitamin D-binding protein	VTDB_HUMAN
	5.139	0.6424	1.86869	Retinol-binding protein 4	RET4_HUMAN
	4.098	0.5854	1.83419	Cytochrome P450 2C8	CP2C8_HUMAN
	3.788	0.3443	1.80974	FK506-binding protein 3	FKBP3_HUMAN
	3.693	0.6155	1.8092	cGMP-specific 3,5-cyclic phosphodiesterase	PDE5A_HUMAN
	3.83	0.766	1.73756	Calmodulin	CALM_HUMAN
	4.163	0.5947	1.72257	Hepatocyte nuclear factor 4-gamma	Q14541
	4.025	0.6709	1.65188	Glutathione-requiring prostaglandin D synthase	PTGD2_HUMAN
	3.637	0.7273	1.63867	Steroid hormone receptor ERR1	P11474
	3.963	0.6604	1.61461	E3 ubiquitin-protein ligase Mdm2	MDM2_HUMAN
	3.843	0.6406	1.54706	Dipeptidyl peptidase 4	DPP4_HUMAN
	4.694	0.5216	1.51528	Cellular retinoic acid-binding protein 2	P29373
	3.694	0.7388	1.50112	Alpha-tocopherol transfer protein	P49638
	3.555	0.5926	1.46256	Ganglioside GM2 activator	P17900
	3.624	0.7248	1.43812	Epoxide hydrolase 2	HYES_HUMAN
	3.658	0.6097	1.43272	Flavin reductase	P30043
	4.322	0.4803	1.4024	Retinoic acid receptor beta	RARB_HUMAN
	3.975	0.6625	1.38769	Alcohol dehydrogenase class-3	ADHX_HUMAN
	3.707	0.6179	1.38329	Oxysterols receptor LXR-alpha	NR1H3_HUMAN
	4.18	0.5971	1.363	Fatty acid-binding protein, brain	FABP7_HUMAN
	3.766	0.6277	1.35101	Tyrosine-protein kinase ITK/TSK	Q08881
	4	0.5715	1.34492	Cytochrome P450 2C9	CP2C9_HU

					MAN
	3.827	0.6378	1.33665	[Pyruvate dehydrogenase [lipoamide]] kinase isozyme 2, mitochondrial	PDK2_HUMAN
	3.389	0.8472	1.33148	Macrophage migration inhibitory factor	MIF_HUMAN
	3.708	0.5297	1.33005	72 kDa type IV collagenase	MMP2_HUMAN
	3.728	0.7456	1.31739	Corticosteroid 11-beta-dehydrogenase isozyme 1	DHI1_HUMAN
	3.721	0.6201	1.30866	Integrin alpha-L	ITAL_HUMAN
	4.221	0.3837	1.26853	Phosphatidylcholine transfer protein	Q9UKL6
	3.379	0.8446	1.23299	Epidermal growth factor receptor	EGFR_HUMAN
	3.632	0.7264	1.21911	Prothrombin	THRB_HUMAN
	3.825	0.6375	1.21391	Adenosine kinase	P55263
	3.751	0.5358	1.21145	Tyrosine-protein kinase ZAP-70	ZAP70_HUMAN
	3.481	0.6962	1.19657	Histone deacetylase 8	Q9BY41
	3.791	0.7581	1.19199	Estradiol 17-beta-dehydrogenase 1	P14061
	3.652	0.6087	1.18319	Tyrosine-protein kinase JAK3	JAK3_HUMAN
	3.913	0.4891	1.18204	Ferrochelatase, mitochondrial	P22830
	3.794	0.5421	1.17022	Protein kinase C theta type	KPCT_HUMAN
	4.355	0.4839	1.1275	Dual specificity mitogen-activated protein kinase kinase 1	MP2K1_HUMAN
	3.621	0.5173	1.12507	Proactivator polypeptide	P07602
	3.693	0.5276	1.09857	Retinoic acid receptor gamma	RARG_HUMAN
	3.498	0.583	1.08997	Alpha-1-antitrypsin	A1AT_HUMAN
	4.129	0.5162	1.08455	Glucocorticoid receptor	GCR_HUMAN
	3.862	0.5516	1.05718	Estrogen sulfotransferase	ST1E1_HUMAN
	3.5	0.3889	1.03845	Bcl-2-like protein 1	Q07817

	3.969	0.5671	1.02723	Fatty acid-binding protein, heart	FABPH_HUMAN
	3.317	0.6633	0.99214	Serine/threonine-protein kinase PAK 7	PAK7_HUMAN
	3.432	0.5721	0.981136	Insulin-like growth factor IA	P01343
	3.374	0.8436	0.975719	Mitogen-activated protein kinase 14	Q16539
	3.38	0.8451	0.919183	ADAM 17	ADA17_HUMAN
	3.508	0.3898	0.91542	Phosphatidylinositol 3-kinase regulatory subunit alpha	P85A_HUMAN
	3.499	0.4373	0.902622	Glutamate carboxypeptidase 2	FOLH1_HUMAN
	3.428	0.6856	0.888958	Bile acid receptor	NR1H4_HUMAN
	3.463	0.4328	0.864115	Vitamin D3 receptor	VDR_HUMAN
	3.926	0.6543	0.862154	Phosphoenolpyruvate carboxykinase, cytosolic [GTP]	P35558
	3.813	0.5447	0.83976	Sulfotransferase family cytosolic 2B member 1	O00204
	3.445	0.4307	0.834454	Heat shock protein HSP 90-beta	P08238
	3.699	0.5285	0.818897	Tyrosine-protein kinase HCK	HCK_HUMAN
**(E,E)-1-ethyl octadeca-3,13-dienoate**	4.818	0.6883	3.24485	Cytochrome P450 2C8	CP2C8_HUMAN
	4.157	0.6929	2.73545	Flavin reductase	P30043
	4.411	0.7351	2.51873	Trafficking protein particle complex subunit 3	O43617
	4.464	0.6378	2.51782	Lanosterol synthase	ERG7_HUMAN
	3.871	0.9677	2.30543	Mitogen-activated protein kinase 14	Q16539
	4.188	0.5983	2.27289	72 kDa type IV collagenase	MMP2_HUMAN
	4.391	0.4879	2.06672	Proto-oncogene tyrosine-protein kinase ABL1	P00519
	3.801	0.9502	2.03595	Collagenase 3	MMP13_HUMAN
	4.138	0.8275	1.95689	SEC14-like protein 2	O76054
	4.493	0.6419	1.93399	Fatty acid-binding protein, heart	FABPH_HU

					MAN
	3.812	0.953	1.83657	ADAM 17	ADA17_HUMAN
	3.65	0.9124	1.8187	Nuclear receptor ROR-alpha	RORA_HUMAN
	3.672	0.7343	1.79951	Serine/threonine-protein kinase PAK 7	PAK7_HUMAN
	4.235	0.605	1.76541	Hepatocyte nuclear factor 4-gamma	Q14541
	3.682	0.6136	1.744	Ganglioside GM2 activator	P17900
	3.77	0.4189	1.70888	Bcl-2-like protein 1	Q07817
	4.151	0.593	1.68503	Thyroid hormone receptor alpha	P10827
	4.547	0.5053	1.66472	Retinoic acid receptor beta	RARB_HUMAN
	3.683	0.7366	1.64709	cAMP-dependent protein kinase catalytic subunit alpha	P00517
	4.485	0.4077	1.62366	Phosphatidylcholine transfer protein	Q9UKL6
	4.295	0.3304	1.61273	Endothelial protein C receptor	EPCR_HUMAN
	3.837	0.7674	1.56173	Mineralocorticoid receptor	MCR_HUMAN
	3.698	0.7397	1.56165	Bile acid receptor	NR1H4_HUMAN
	3.856	0.6426	1.55581	Integrin alpha-L	ITAL_HUMAN
	3.807	0.7614	1.55302	Tyrosine-protein phosphatase non-receptor type 11	PTN11_HUMAN
	3.785	0.7571	1.41512	Wiskott-Aldrich syndrome protein	WASP_HUMAN
	3.628	0.7257	1.41142	Poly [ADP-ribose] polymerase 1	P09874
	3.901	0.7802	1.35136	Estradiol 17-beta-dehydrogenase 1	P14061
	3.529	0.7058	1.33203	Heat shock protein HSP 90-alpha	HS90A_HUMAN
	3.746	0.9366	1.33085	Vitamin D-binding protein	VTDB_HUMAN
	3.613	0.6022	1.31138	Alpha-1-antitrypsin	A1AT_HUMAN
	4.01	0.4455	1.29754	Mast/stem cell growth factor receptor	KIT_HUMAN

	3.845	0.6408	1.29592	[Pyruvate dehydrogenase [lipoamide]] kinase isozyme 2, mitochondrial	PDK2_HUMAN
	3.719	0.6198	1.22654	Retinol-binding protein 4	RET4_HUMAN
	3.844	0.6406	1.19962	Glutathione-requiring prostaglandin D synthase	PTGD2_HUMAN
	3.625	0.6041	1.19457	Receptor tyrosine-protein kinase erbB-4	ERBB4_HUMAN
	4.509	0.4509	1.17668	Retinoic acid receptor RXR-beta	RXRB_HUMAN
	3.702	0.7404	1.1759	Corticosteroid 11-beta-dehydrogenase isozyme 1	DHI1_HUMAN
	3.521	0.7042	1.16907	cAMP-specific 3,5-cyclic phosphodiesterase 4D	PDE4D_HUMAN
	3.736	0.5337	1.12824	Retinoic acid receptor gamma	RARG_HUMAN
	3.452	0.6904	1.11485	Steroid hormone receptor ERR1	P11474
	3.652	0.6087	1.11332	Tyrosine-protein kinase JAK3	JAK3_HUMAN
	3.681	0.4601	1.0948	Betaine--homocysteine S-methyltransferase 1	Q93088
	5.558	0.5558	1.0784	Cellular retinoic acid-binding protein 2	P29373
	3.854	0.6423	1.04337	Alcohol dehydrogenase class-3	ADHX_HUMAN
	3.756	0.5366	1.02875	Tyrosine-protein kinase JAK2	JAK2_HUMAN
	3.549	0.5915	1.01149	Peroxisome proliferator-activated receptor alpha	PPARA_HUMAN
	3.458	0.5763	1.0034	Insulin-like growth factor IA	P01343
	3.55	0.5071	0.981997	Peptidyl-prolyl cis-trans isomerase FKBP1A	FKB1A_HUMAN
	3.612	0.516	0.98078	Interleukin-2	IL2_HUMAN
	4.014	0.5734	0.972549	Fatty acid-binding protein, brain	FABP7_HUMAN
	3.67	0.6116	0.959584	Glutathione S-transferase A1	P08263
	4.28	0.4755	0.939564	Dual specificity mitogen-activated protein kinase kinase 1	MP2K1_HUMAN
	3.559	0.7118	0.93475	Calmodulin	CALM_HU

					MAN
	3.359	0.6717	0.928261	Alcohol dehydrogenase 1C	P00326
	3.743	0.4159	0.899302	Serine/threonine-protein phosphatase PP1-gamma catalytic subunit	P36873
	3.473	0.6945	0.888726	Alpha-tocopherol transfer protein	P49638
	3.755	0.4173	0.87453	Growth factor receptor-bound protein 2	GRB2_HUMAN
	3.534	0.589	0.826336	Nuclear receptor subfamily 1 group I member 3	NR1I3_HUMAN
	3.685	0.6141	0.821474	Adenosine kinase	P55263
	3.829	0.547	0.807191	Sulfotransferase family cytosolic 2B member 1	O00204
**2,6,10,14,18-pentamethylicosa-2,6,10,14,18-pentaene**	4.965	0.8275	2.43115	Aldose reductase	ALDR_HUMAN
	5.048	0.7211	2.33241	Lanosterol synthase	ERG7_HUMAN
	5.366	0.4127	2.2261	Endothelial protein C receptor	EPCR_HUMAN
	4.529	0.7549	2.2142	ADAM 17	ADA17_HUMAN
	5.609	0.6232	2.08708	Retinoic acid receptor beta	RARB_HUMAN
	4.455	0.7425	2.04791	Flavin reductase	P30043
	4.667	0.9334	1.82003	SEC14-like protein 2	O76054
	4.738	0.6769	1.8034	Cytochrome P450 2C8	CP2C8_HUMAN
	4.603	0.7672	1.69089	Trafficking protein particle complex subunit 3	O43617
	4.78	0.5975	1.56401	Ferrochelatase, mitochondrial	P22830
	4.825	0.6032	1.54977	Retinoic acid receptor gamma	RARG_HUMAN
	4.616	0.577	1.54456	Caspase-3	CASP3_HUMAN
	5.043	0.6304	1.53413	Histamine N-methyltransferase	P50135
	3.969	0.9922	1.4581	Nuclear receptor ROR-alpha	RORA_HUMAN
	4.235	0.5293	1.45204	Vitamin D3 receptor	VDR_HUMAN
	5.695	0.7119	1.44573	Retinol-binding protein 4	RET4_HUMAN

	4	0.8	1.41275	Glutathione S-transferase P	GSTP1_HUMAN
	4.241	0.7069	1.37087	Mitogen-activated protein kinase 10	P53779
	4.479	0.7465	1.36323	Adenosine kinase	P55263
	5.018	0.6273	1.29193	Fatty acid-binding protein, heart	FABPH_HUMAN
	4.568	0.6526	1.2882	Hepatocyte nuclear factor 4-gamma	Q14541
	4.05	0.81	1.27325	Bile acid receptor	NR1H4_HUMAN
	4.909	0.4463	1.25393	Phosphatidylcholine transfer protein	Q9UKL6
	5.285	0.5285	1.24747	Retinoic acid receptor RXR-beta	RXRB_HUMAN
	3.89	0.6484	1.23574	cGMP-specific 3,5-cyclic phosphodiesterase	PDE5A_HUMAN
	3.926	0.7852	1.21443	Steroid hormone receptor ERR1	P11474
	4.692	0.5213	1.17934	Angiopoietin-1 receptor	TIE2_HUMAN
	4.623	0.6604	1.16972	Sulfotransferase family cytosolic 2B member 1	O00204
	4.696	0.6708	1.12347	Fatty acid-binding protein, brain	FABP7_HUMAN
	3.853	0.7706	1.12049	E3 ubiquitin-protein ligase Mdm2	MDM2_HUMAN
	4.1	0.6833	1.10774	Oxysterols receptor LXR-alpha	NR1H3_HUMAN
	4.308	0.7181	1.09699	Glutathione-requiring prostaglandin D synthase	PTGD2_HUMAN
	4.534	0.6477	1.07865	Gastrotropin	FABP6_HUMAN
	4.095	0.5851	1.06567	Proactivator polypeptide	P07602
	4	0.6667	1.06493	Tyrosine-protein phosphatase non-receptor type 1	PTN1_HUMAN
	4	1	1.05811	Kinesin-like protein KIF11	KIF11_HUMAN
	3.859	0.6432	1.05639	Renin	RENI_HUMAN
	3.855	0.771	1.03877	Dipeptidyl peptidase 4	DPP4_HUMAN

	3.868	0.7736	1.03683	Histone deacetylase 8	Q9BY41
	5.013	0.557	1.03393	Dual specificity mitogen-activated protein kinase kinase 1	MP2K1_HUMAN
	4.191	0.6985	1.03015	Glutathione S-transferase A1	P08263
	3.891	0.7782	1.02868	Aldo-keto reductase family 1 member C1	Q04828
	3.957	0.4397	1.02687	Bcl-2-like protein 1	Q07817
	3.823	0.6372	1.00811	Ganglioside GM2 activator	P17900
	3.941	0.6568	0.994957	Poly [ADP-ribose] polymerase 1	P09874
	3.796	0.7591	0.972135	Hepatocyte growth factor receptor	P08581
	4.024	0.8048	0.96202	Calmodulin	CALM_HUMAN
	3.75	0.9375	0.94585	Cell division protein kinase 2	P24941
	4.151	0.6918	0.944491	Tyrosine-protein kinase SYK	KSYK_HUMAN
	3.839	0.7678	0.941935	cAMP-dependent protein kinase catalytic subunit alpha	P00517
	3.868	0.7735	0.940861	Proto-oncogene tyrosine-protein kinase Src	SRC_HUMAN
	4	0.5714	0.936342	72 kDa type IV collagenase	MMP2_HUMAN
	3.826	0.2943	0.919763	Amine oxidase [flavin-containing] A	AOFA_HUMAN
	3.954	0.659	0.919326	Peroxisome proliferator-activated receptor alpha	PPARA_HUMAN
	4	0.8	0.909102	Prothrombin	THRB_HUMAN
	3.844	0.7688	0.904953	Vascular endothelial growth factor receptor 2	P35968
	4	0.8	0.882343	Tyrosine-protein phosphatase non-receptor type 11	PTN11_HUMAN
	4.005	0.6676	0.877299	Nuclear receptor subfamily 1 group I member 3	NR1I3_HUMAN
	4.215	0.4684	0.846869	Serine/threonine-protein phosphatase PP1-gamma catalytic subunit	P36873
	3.993	0.6656	0.844623	Tyrosine-protein kinase JAK3	JAK3_HUMAN
	4.474	0.5592	0.811581	Basic fibroblast growth factor receptor 1	FGFR1_HUMAN

	4	0.8	0.801312	Corticosteroid 11-beta-dehydrogenase isozyme 1	DHI1_HUMAN
**3,4-Dehydrolycopen-16-al**	5.804	0.6449	2.76454	Angiopoietin-1 receptor	TIE2_HUMAN
	5.835	0.7294	2.70344	Histamine N-methyltransferase	P50135
	5.027	0.7181	2.46478	Lanosterol synthase	ERG7_HUMAN
	4.494	0.8987	2.45705	Bile acid receptor	NR1H4_HUMAN
	4.625	0.7708	2.45004	Beta-secretase 1	P56817
	5	0.7143	2.40551	Cytochrome P450 2C8	CP2C8_HUMAN
	4.869	0.6086	2.39689	Interleukin-2	IL2_HUMAN
	5.344	0.411	2.3268	Endothelial protein C receptor	EPCR_HUMAN
	4.527	0.9055	2.31964	Alpha-tocopherol transfer protein	P49638
	4.697	0.9393	2.06565	SEC14-like protein 2	O76054
	4.565	0.7609	2.01373	Dipeptidyl peptidase 4	DPP4_HUMAN
	4.41	0.735	1.91133	Nuclear receptor subfamily 1 group I member 3	NR1I3_HUMAN
	4.545	0.6492	1.87598	Retinoic acid receptor gamma	RARG_HUMAN
	4.584	0.764	1.85634	Trafficking protein particle complex subunit 3	O43617
	4	1	1.71871	Mitogen-activated protein kinase 14	Q16539
	3.992	0.7985	1.71561	Glutathione S-transferase P	GSTP1_HUMAN
	3.843	0.9607	1.6852	Proto-oncogene tyrosine-protein kinase Src	SRC_HUMAN
	4.56	0.5066	1.66533	Serine/threonine-protein phosphatase PP1-gamma catalytic subunit	P36873
	4.479	0.7464	1.65484	E3 ubiquitin-protein ligase Mdm2	MDM2_HUMAN
	3.998	0.7997	1.65357	Histone deacetylase 8	Q9BY41
	3.917	0.9793	1.64924	Coagulation factor X	FA10_HUMAN
	4.316	0.7194	1.63752	Retinol-binding protein 4	RET4_HU

					MAN
	4.113	0.6855	1.59383	Flavin reductase	P30043
	3.994	0.7987	1.568	cAMP-dependent protein kinase catalytic subunit alpha	P00517
	5.05	0.5611	1.52196	Retinoic acid receptor beta	RARB_HUMAN
	3.974	0.7948	1.49084	Methionine aminopeptidase 2	AMPM2_HUMAN
	3.875	0.6458	1.422	Ganglioside GM2 activator	P17900
	4.926	0.4479	1.41607	Phosphatidylcholine transfer protein	Q9UKL6
	4.269	0.7115	1.40141	Glutathione S-transferase A1	P08263
	4.491	0.6416	1.35661	Hepatocyte nuclear factor 4-gamma	Q14541
	3.856	0.7712	1.35467	Steroid hormone receptor ERR1	P11474
	3.957	0.6595	1.34158	Alpha-1-antitrypsin	A1AT_HUMAN
	3.973	0.6622	1.34148	Poly [ADP-ribose] polymerase 1	P09874
	4	1	1.33736	Kinesin-like protein KIF11	KIF11_HUMAN
	3.843	0.6405	1.33644	Renin	RENI_HUMAN
	3.772	0.6287	1.29547	cGMP-specific 3,5-cyclic phosphodiesterase	PDE5A_HUMAN
	4.451	0.4945	1.28091	Mast/stem cell growth factor receptor	KIT_HUMAN
	4.585	0.655	1.27627	Sulfotransferase family cytosolic 2B member 1	O00204
	3.849	0.7698	1.20337	Vascular endothelial growth factor receptor 2	P35968
	4.079	0.5098	1.19268	Proto-oncogene tyrosine-protein kinase ABL1	P00519
	4.376	0.547	1.16686	Ferrochelatase, mitochondrial	P22830
	4	0.8	1.16465	Tyrosine-protein phosphatase non-receptor type 11	PTN11_HUMAN
	4	0.5714	1.16268	72 kDa type IV collagenase	MMP2_HUMAN
	3.794	0.7588	1.14988	Tyrosine-protein kinase ITK/TSK	Q08881
	3.965	0.7929	1.12555	Calmodulin	CALM_HUMAN

	3.97	0.6617	1.12433	Oxysterols receptor LXR-alpha	NR1H3_HUMAN
	4.989	0.5544	1.12107	Dual specificity mitogen-activated protein kinase kinase 1	MP2K1_HUMAN
	5.064	0.5064	1.11941	Retinoic acid receptor RXR-beta	RXRB_HUMAN
	3.991	0.6651	1.11247	Tyrosine-protein kinase JAK3	JAK3_HUMAN
	4.565	0.6521	1.10476	Fatty acid-binding protein, brain	FABP7_HUMAN
	4	0.8	1.0935	Wiskott-Aldrich syndrome protein	WASP_HUMAN
	4	1	1.08988	Vitamin D-binding protein	VTDB_HUMAN
	3.659	0.9147	1.08932	Nuclear receptor ROR-alpha	RORA_HUMAN
	3.745	0.9363	1.08098	ADAM 17	ADA17_HUMAN
	3.96	0.5657	1.07329	Proactivator polypeptide	P07602
	4.402	0.6289	1.06656	Gastrotropin	FABP6_HUMAN
	3.828	0.4253	1.05687	Bcl-2-like protein 1	Q07817
	3.877	0.6462	1.0449	Receptor tyrosine-protein kinase erbB-4	ERBB4_HUMAN
	4	0.6667	1.01931	cAMP-specific 3,5-cyclic phosphodiesterase 4D	PDE4D_HUMAN
	3.743	0.6238	1.0181	Insulin-like growth factor IA	P01343
	4.112	0.6853	0.998186	Glutathione-requiring prostaglandin D synthase	PTGD2_HUMAN
	3.841	0.4801	0.992762	Vitamin D3 receptor	VDR_HUMAN
	3.852	0.428	0.959674	Phosphatidylinositol 3-kinase regulatory subunit alpha	P85A_HUMAN
	4.894	0.5438	0.929238	Cellular retinoic acid-binding protein 2	P29373
	3.684	0.7368	0.914864	Aldo-keto reductase family 1 member C1	Q04828
	3.954	0.4943	0.913042	Stromelysin-1	MMP3_HUMAN
	3.85	0.7699	0.892013	Prothrombin	THRB_HUMAN

	3.765	0.4706	0.874449	Heat shock protein HSP 90-beta	P08238
	3.774	0.6291	0.846135	Peroxisome proliferator-activated receptor alpha	PPARA_HUMAN
	4	0.8	0.842615	Estradiol 17-beta-dehydrogenase 1	P14061
	3.772	0.5388	0.820057	Protein S100-A9	S10A9_HUMAN
	4.032	0.672	0.816553	Adenosine kinase	P55263
	3.859	0.7718	0.803441	Corticosteroid 11-beta-dehydrogenase isozyme 1	DHI1_HUMAN
**4,24-methyllophenol**	4.191	0.8382	2.61616	Aldo-keto reductase family 1 member C1	Q04828
	4.871	0.6088	2.53577	Ferrochelatase, mitochondrial	P22830
	3.917	0.9791	2.46587	Nuclear receptor ROR-alpha	RORA_HUMAN
	4.439	0.7398	2.46414	Glutathione S-transferase A1	P08263
	4.557	0.5696	2.40653	Caspase-3	CASP3_HUMAN
	4.792	0.599	2.33039	Retinoic acid receptor gamma	RARG_HUMAN
	4.428	0.738	2.31717	Trafficking protein particle complex subunit 3	O43617
	5.123	0.5692	2.25275	Retinoic acid receptor beta	RARB_HUMAN
	4.127	0.6878	2.19352	Oxysterols receptor LXR-alpha	NR1H3_HUMAN
	4.674	0.6677	2.07741	Gastrotropin	FABP6_HUMAN
	4.24	0.6057	1.87112	Lanosterol synthase	ERG7_HUMAN
	4.238	0.6054	1.84653	Cytochrome P450 2C8	CP2C8_HUMAN
	3.78	0.756	1.8377	cAMP-dependent protein kinase catalytic subunit alpha	P00517
	4.218	0.703	1.82573	Adenosine kinase	P55263
	4.531	0.6473	1.79676	Sulfotransferase family cytosolic 2B member 1	O00204
	3.673	0.6121	1.7847	cGMP-specific 3,5-cyclic phosphodiesterase	PDE5A_HUMAN
	3.829	0.6382	1.66773	Peroxisome proliferator-activated receptor alpha	PPARA_HUMAN

	3.651	0.7302	1.65377	Dipeptidyl peptidase 4	DPP4_HUMAN
	3.746	0.7493	1.62954	Bile acid receptor	NR1H4_HUMAN
	4.614	0.4195	1.61333	Phosphatidylcholine transfer protein	Q9UKL6
	3.775	0.6292	1.60591	Flavin reductase	P30043
	3.874	0.5534	1.53244	72 kDa type IV collagenase	MMP2_HUMAN
	3.588	0.7177	1.51798	Glutathione S-transferase P	GSTP1_HUMAN
	3.568	0.8921	1.50494	Cell division protein kinase 2	P24941
	4.418	0.6311	1.50104	Fatty acid-binding protein, brain	FABP7_HUMAN
	4.002	0.6669	1.48081	E3 ubiquitin-protein ligase Mdm2	MDM2_HUMAN
	3.829	0.7658	1.47394	Mineralocorticoid receptor	MCR_HUMAN
	5.105	0.6382	1.47271	Retinol-binding protein 4	RET4_HUMAN
	3.529	0.8822	1.46099	Peroxisome proliferator-activated receptor gamma	PPARG_HUMAN
	3.585	0.5975	1.42615	Histo-blood group ABO system transferase	P16442
	3.482	0.8706	1.3841	Estradiol 17-beta-dehydrogenase 1	P14061
	3.791	0.9477	1.37659	Vitamin D-binding protein	VTDB_HUMAN
	3.744	0.7488	1.36055	Calmodulin	CALM_HUMAN
	3.539	0.7078	1.3509	cAMP-specific 3,5-cyclic phosphodiesterase 4D	PDE4D_HUMAN
	3.706	0.4117	1.33715	Phosphatidylinositol 3-kinase regulatory subunit alpha	P85A_HUMAN
	4.231	0.3255	1.33502	Endothelial protein C receptor	EPCR_HUMAN
	3.858	0.7717	1.26554	SEC14-like protein 2	O76054
	3.749	0.7497	1.26527	Wiskott-Aldrich syndrome protein	WASP_HUMAN
	4.307	0.5384	1.26066	Histamine N-methyltransferase	P50135
	3.687	0.7374	1.25682	Prothrombin	THRB_HU

					MAN
	3.475	0.5792	1.21551	Ganglioside GM2 activator	P17900
	3.445	0.689	1.13828	Steroid hormone receptor ERR1	P11474
	3.696	0.462	1.10348	Vitamin D3 receptor	VDR_HUMAN
	3.712	0.6186	1.0901	Tyrosine-protein kinase ITK/TSK	Q08881
	3.819	0.5455	1.08644	Protein kinase C theta type	KPCT_HUMAN
	4.498	0.4998	1.07663	Dual specificity mitogen-activated protein kinase kinase 1	MP2K1_HUMAN
	3.939	0.5626	1.07547	Hepatocyte nuclear factor 4-gamma	Q14541
	3.748	0.6247	1.0724	Tyrosine-protein kinase SYK	KSYK_HUMAN
	3.81	0.635	1.06955	Glutathione-requiring prostaglandin D synthase	PTGD2_HUMAN
	3.738	0.534	1.06449	Tyrosine-protein kinase ZAP-70	ZAP70_HUMAN
	3.349	0.8372	1.0592	Coagulation factor X	FA10_HUMAN
	3.654	0.7307	1.03092	Corticosteroid 11-beta-dehydrogenase isozyme 1	DHI1_HUMAN
	3.64	0.6067	1.00845	Oxysterols receptor LXR-beta	NR1H2_HUMAN
	3.428	0.5714	0.97203	Insulin-like growth factor IA	P01343
	3.528	0.504	0.947722	Protein S100-A9	S10A9_HUMAN
	3.595	0.5136	0.936724	Interleukin-2	IL2_HUMAN
	3.962	0.6603	0.930248	Sex hormone-binding globulin	SHBG_HUMAN
	4.423	0.4423	0.911311	Retinoic acid receptor RXR-beta	RXRB_HUMAN
	3.665	0.4581	0.90065	3-hydroxy-3-methylglutaryl-coenzyme A reductase	HMDH_HUMAN
	4.12	0.515	0.878892	Glucocorticoid receptor	GCR_HUMAN
	3.803	0.6338	0.872643	Alcohol dehydrogenase class-3	ADHX_HUMAN
	3.738	0.4153	0.839713	Serine/threonine-protein	P36873

				phosphatase PP1-gamma catalytic subunit	
	3.53	0.5883	0.829981	Nuclear receptor subfamily 1 group I member 3	NR1I3_HUMAN
	3.796	0.5423	0.829827	Cytochrome P450 2C9	CP2C9_HUMAN
	3.955	0.565	0.828489	Fatty acid-binding protein, heart	FABPH_HUMAN
**14b-pregnane**	3.721	0.7442	3.83603	Glutathione S-transferase P	GSTP1_HUMAN
	4.306	0.5382	3.07497	Retinoic acid receptor RXR-alpha	RXRA_HUMAN
	3.65	0.9124	2.7799	Vitamin D-binding protein	VTDB_HUMAN
	3.919	0.3014	2.22854	Endothelial protein C receptor	EPCR_HUMAN
	3.643	0.6072	2.11363	Adenosine kinase	P55263
	3.319	0.6638	1.942	Aldo-keto reductase family 1 member C1	Q04828
	3.729	0.5327	1.93126	Fatty acid-binding protein, heart	FABPH_HUMAN
	3.627	0.4534	1.70448	Ferrochelatase, mitochondrial	P22830
	3.636	0.4546	1.35593	Basic fibroblast growth factor receptor 1	FGFR1_HUMAN
	3.504	0.438	1.33493	Retinoic acid receptor gamma	RARG_HUMAN
	2.998	0.9994	1.21757	Bone morphogenetic protein 2	BMP2_HUMAN
	3.556	0.5081	1.21402	Fatty acid-binding protein, brain	FABP7_HUMAN
	3.622	0.3293	1.20299	Phosphatidylcholine transfer protein	Q9UKL6
	3.838	0.3838	1.19123	Retinoic acid receptor RXR-beta	RXRB_HUMAN
	3.832	0.4258	1.16636	Cellular retinoic acid-binding protein 2	P29373
	2.953	0.7384	1.16	Carbonic anhydrase 2	CAH2_HUMAN
	3.26	0.4657	1.13588	Cyclin-A2	CCNA2_HUMAN
	3.45	0.4929	1.11539	Sulfotransferase family cytosolic	O00204

				2B member 1	
	2.924	0.9747	1.10927	Caspase-7	CASP7_HUMAN
	2.968	0.742	1.09106	Serine/threonine-protein phosphatase 5	P53041
	3.312	0.4731	1.05566	Nuclear receptor subfamily 1 group I member 2	NR1I2_HUMAN
	3.497	0.4371	1.0188	Histamine N-methyltransferase	P50135
	2.964	0.5927	1.01011	Placenta growth factor	PLGF_HUMAN
	2.914	0.7285	1.00075	Proto-oncogene tyrosine-protein kinase Src	SRC_HUMAN
	2.883	0.9609	0.994327	Aldo-keto reductase family 1 member C2	AK1C2_HUMAN
	2.94	0.7349	0.991843	Estradiol 17-beta-dehydrogenase 1	P14061
	3.379	0.4827	0.980308	Gastrotropin	FABP6_HUMAN
	3.237	0.5394	0.967897	Glutathione-requiring prostaglandin D synthase	PTGD2_HUMAN
	2.958	0.7394	0.963437	Progesterone receptor	PRGR_HUMAN
	3.317	0.4739	0.959753	Hepatocyte nuclear factor 4-gamma	Q14541
	3.515	0.4394	0.951383	Glucocorticoid receptor	GCR_HUMAN
	2.899	0.5798	0.924215	Methionine aminopeptidase 2	AMPM2_HUMAN
	3.156	0.3945	0.909206	Stromelysin-1	MMP3_HUMAN
	2.917	0.4168	0.886134	Coagulation factor VII	FA7_HUMAN
	3.455	0.3141	0.867841	Serine/threonine-protein phosphatase PP1-gamma catalytic subunit	P36873
	3.614	0.4016	0.862332	Dual specificity mitogen-activated protein kinase kinase 1	MP2K1_HUMAN
	2.948	0.7371	0.860793	Amine oxidase [flavin-containing] B	AOFB_HUMAN
	2.976	0.5951	0.854236	Corticosteroid 11-beta-dehydrogenase isozyme 1	P28845

	2.941	0.5882	0.835559	E3 ubiquitin-protein ligase Mdm2	MDM2_HUMAN
	2.848	0.9492	0.818161	Steryl-sulfatase	STS_HUMAN
	3.443	0.3826	0.806166	Retinoic acid receptor beta	RARB_HUMAN
	2.825	0.9417	0.801665	Serum albumin	ALBU_HUMAN
**4alpha-methyl-24-ethylcholesta-7,24-dienol**	4.362	0.8723	2.60782	Mineralocorticoid receptor	MCR_HUMAN
	3.838	0.9595	2.48842	Nuclear receptor ROR-alpha	RORA_HUMAN
	4.321	0.7202	2.30157	Trafficking protein particle complex subunit 3	O43617
	4.186	0.8372	2.06942	SEC14-like protein 2	O76054
	4.601	0.6573	2.05749	Sulfotransferase family cytosolic 2B member 1	O00204
	3.664	0.7328	2.03298	Alcohol dehydrogenase 1C	P00326
	3.886	0.7772	1.97368	Prothrombin	THRB_HUMAN
	3.659	0.9148	1.86391	Progesterone receptor	PRGR_HUMAN
	4.134	0.5906	1.85024	Lanosterol synthase	ERG7_HUMAN
	4.128	0.8256	1.84856	Estradiol 17-beta-dehydrogenase 1	P14061
	4.331	0.5414	1.84284	Ferrochelatase, mitochondrial	P22830
	3.918	0.7836	1.79218	Corticosteroid 11-beta-dehydrogenase isozyme 1	DHI1_HUMAN
	3.85	0.6417	1.64625	Ephrin type-B receptor 4	EPHB4_HUMAN
	3.778	0.7555	1.63513	Calmodulin	CALM_HUMAN
	3.647	0.7294	1.59389	Bile acid receptor	NR1H4_HUMAN
	3.72	0.465	1.5917	Heat shock protein HSP 90-beta	P08238
	3.998	0.5711	1.57524	Cytochrome P450 2C8	CP2C8_HUMAN
	3.986	0.6644	1.53593	Glutathione-requiring prostaglandin D synthase	PTGD2_HUMAN
	3.939	0.4924	1.51864	Proto-oncogene tyrosine-protein	P00519

				kinase ABL1	
	3.522	0.7044	1.43629	Dipeptidyl peptidase 4	DPP4_HUMAN
	3.696	0.6159	1.42512	Oxysterols receptor LXR-alpha	NR1H3_HUMAN
	3.722	0.5317	1.42223	Macrophage metalloelastase	MMP12_HUMAN
	4.132	0.5903	1.3833	Gastrotropin	FABP6_HUMAN
	4.716	0.4716	1.3823	Retinoic acid receptor RXR-beta	RXRB_HUMAN
	3.699	0.6165	1.37979	Nuclear receptor subfamily 1 group I member 3	NR1I3_HUMAN
	3.635	0.4039	1.36568	Phosphatidylinositol 3-kinase regulatory subunit alpha	P85A_HUMAN
	3.524	0.7049	1.36257	Aldo-keto reductase family 1 member C1	Q04828
	3.447	0.8617	1.35679	Collagenase 3	MMP13_HUMAN
	3.708	0.5297	1.31695	Interleukin-2	IL2_HUMAN
	4.547	0.5053	1.26263	Dual specificity mitogen-activated protein kinase kinase 1	MP2K1_HUMAN
	3.98	0.5686	1.25573	Estrogen sulfotransferase	ST1E1_HUMAN
	3.374	0.5623	1.2447	cGMP-specific 3,5-cyclic phosphodiesterase	PDE5A_HUMAN
	3.389	0.5648	1.24219	Peroxisome proliferator-activated receptor delta	PPARD_HUMAN
	4.02	0.5743	1.23524	Mitogen-activated protein kinase 10	MK10_HUMAN
	3.94	0.5628	1.23479	Hepatocyte nuclear factor 4-gamma	Q14541
	3.617	0.5166	1.2149	72 kDa type IV collagenase	MMP2_HUMAN
	3.602	0.7205	1.19488	Tyrosine-protein phosphatase non-receptor type 11	PTN11_HUMAN
	4.226	0.3841	1.18586	Phosphatidylcholine transfer protein	Q9UKL6
	3.514	0.5856	1.17179	Flavin reductase	P30043
	3.535	0.4418	1.1617	Vitamin D3 receptor	VDR_HUM

					AN
	3.55	0.5916	1.15969	NAD(P)H dehydrogenase [quinone] 1	NQO1_HUMAN
	4.693	0.5866	1.15131	Retinol-binding protein 4	RET4_HUMAN
	5.818	0.5818	1.14382	Cellular retinoic acid-binding protein 2	P29373
	3.677	0.6128	1.1126	Glutathione S-transferase A1	P08263
	3.595	0.8987	1.10456	Vitamin D-binding protein	VTDB_HUMAN
	3.347	0.6694	1.09361	Glutathione S-transferase P	GSTP1_HUMAN
	3.618	0.5168	1.09249	Protein farnesyltransferase/geranylgeranyltransferase type-1 subunit alpha	FNTA_HUMAN
	4.162	0.4624	1.0706	Retinoic acid receptor beta	RARB_HUMAN
	3.827	0.4252	1.03818	Mast/stem cell growth factor receptor	KIT_HUMAN
	3.355	0.6709	1.01381	cAMP-dependent protein kinase catalytic subunit alpha	P00517
	3.701	0.7401	0.936468	Bile salt sulfotransferase	Q06520
	3.434	0.5724	0.933787	Receptor tyrosine-protein kinase erbB-4	ERBB4_HUMAN
	3.356	0.5594	0.930556	Alpha-1-antitrypsin	A1AT_HUMAN
	3.518	0.5864	0.921651	Oxysterols receptor LXR-beta	NR1H2_HUMAN
	3.315	0.8287	0.890967	Estrogen receptor	ESR1_HUMAN
	3.823	0.4779	0.890012	Phospholipase A2, membrane associated	P14555
	3.856	0.2966	0.871367	Endothelial protein C receptor	EPCR_HUMAN
	3.844	0.4271	0.823609	Angiopoietin-1 receptor	TIE2_HUMAN
	3.226	0.5376	0.802127	Ganglioside GM2 activator	P17900
**4alpha,14alpha,24-trimethylcholesta-8,24-dienol**	5.33	0.6663	3.11013	Ferrochelatase, mitochondrial	P22830
	4.366	0.5458	2.56843	Vitamin D3 receptor	VDR_HUMAN
	3.981	0.9952	2.53047	Nuclear receptor ROR-alpha	RORA_HU

					MAN
	5.027	0.7181	2.4992	Gastrotropin	FABP6_HUMAN
	4.294	0.7156	2.45042	Oxysterols receptor LXR-alpha	NR1H3_HUMAN
	4.264	0.6091	2.19507	Renin	RENI_HUMAN
	4.457	0.6367	2.15947	Lanosterol synthase	ERG7_HUMAN
	4.134	0.8267	2.14691	Calmodulin	CALM_HUMAN
	3.851	0.9628	1.98313	Transthyretin	TTHY_HUMAN
	3.986	0.6644	1.9384	Oxysterols receptor LXR-beta	NR1H2_HUMAN
	3.933	0.5619	1.79352	Protein S100-A9	S10A9_HUMAN
	4.86	0.6075	1.74551	Fatty acid-binding protein, heart	FABPH_HUMAN
	3.877	0.9693	1.72313	Kinesin-like protein KIF11	KIF11_HUMAN
	4.024	0.6706	1.71045	Retinol-binding protein 4	RET4_HUMAN
	4.123	0.6872	1.68385	[Pyruvate dehydrogenase [lipoamide]] kinase isozyme 2, mitochondrial	PDK2_HUMAN
	4.117	0.8233	1.67344	SEC14-like protein 2	O76054
	4.302	0.6146	1.61975	Hepatocyte nuclear factor 4-gamma	Q14541
	5.058	0.5058	1.56142	Retinoic acid receptor RXR-beta	RXRB_HUMAN
	4.508	0.6441	1.54803	Fatty acid-binding protein, brain	FABP7_HUMAN
	3.718	0.7436	1.54495	Aldo-keto reductase family 1 member C1	Q04828
	3.738	0.7475	1.54449	Bile acid receptor	NR1H4_HUMAN
	4.368	0.546	1.54441	Peptidyl-prolyl cis-trans isomerase FKBP1A	FKB1A_HUMAN
	3.89	0.5557	1.49811	72 kDa type IV collagenase	MMP2_HUMAN

	3.624	0.6039	1.48067	Histo-blood group ABO system transferase	P16442
	3.876	0.7752	1.46328	Mineralocorticoid receptor	MCR_HUMAN
	4	0.6667	1.44525	Trafficking protein particle complex subunit 3	O43617
	3.792	0.7584	1.44146	Prothrombin	THRB_HUMAN
	3.737	0.7474	1.42061	Alpha-tocopherol transfer protein	P49638
	4.333	0.3333	1.41455	Endothelial protein C receptor	EPCR_HUMAN
	3.624	0.7247	1.41137	cAMP-dependent protein kinase catalytic subunit alpha	P00517
	3.914	0.4892	1.38549	Stromelysin-1	MMP3_HUMAN
	4.536	0.504	1.36798	Retinoic acid receptor beta	RARB_HUMAN
	3.921	0.6535	1.35913	Glutathione S-transferase A1	P08263
	4.494	0.4086	1.35028	Phosphatidylcholine transfer protein	Q9UKL6
	3.962	0.5659	1.27355	Cytochrome P450 2C8	CP2C8_HUMAN
	4.724	0.5249	1.26827	Dual specificity mitogen-activated protein kinase kinase 1	MP2K1_HUMAN
	3.865	0.5521	1.24307	Tyrosine-protein kinase ZAP-70	ZAP70_HUMAN
	3.664	0.6106	1.22638	Peroxisome proliferator-activated receptor alpha	PPARA_HUMAN
	3.75	0.9375	1.20854	Vitamin D-binding protein	VTDB_HUMAN
	3.713	0.6188	1.16027	Tyrosine-protein kinase JAK3	JAK3_HUMAN
	3.699	0.6165	1.13749	Nuclear receptor subfamily 1 group I member 3	NR1I3_HUMAN
	3.467	0.6934	1.12701	NAD(P)H dehydrogenase [quinone] 1	NQO1_HUMAN
	4.108	0.5869	1.10097	Sulfotransferase family cytosolic 2B member 1	O00204
	3.776	0.5395	1.1	Retinoic acid receptor gamma	RARG_HUMAN
	3.46	0.692	1.09501	Dipeptidyl peptidase 4	DPP4_HUM

					AN
	3.852	0.5503	1.08596	Protein kinase C theta type	KPCT_HUMAN
	3.667	0.6112	1.05539	Ephrin type-B receptor 4	EPHB4_HUMAN
	3.646	0.7292	1.05026	Tyrosine-protein phosphatase non-receptor type 11	PTN11_HUMAN
	3.665	0.7331	0.999346	Corticosteroid 11-beta-dehydrogenase isozyme 1	DHI1_HUMAN
	3.634	0.7269	0.956824	Wiskott-Aldrich syndrome protein	WASP_HUMAN
	3.756	0.7511	0.943521	Estradiol 17-beta-dehydrogenase 1	P14061
	4.205	0.5256	0.930676	Glucocorticoid receptor	GCR_HUMAN
	3.364	0.5607	0.921107	Ganglioside GM2 activator	P17900
	3.656	0.6094	0.914365	Tyrosine-protein kinase ITK/TSK	Q08881
	3.492	0.4365	0.88944	Heat shock protein HSP 90-beta	P08238
	3.495	0.5825	0.886207	Tyrosine-protein phosphatase non-receptor type 1	PTN1_HUMAN
	3.378	0.8446	0.876459	Progesterone receptor	PRGR_HUMAN
	3.488	0.5814	0.870463	Flavin reductase	P30043
	3.367	0.6733	0.846478	Histone deacetylase 8	Q9BY41
	3.845	0.5492	0.837947	Estrogen sulfotransferase	ST1E1_HUMAN
	3.64	0.6066	0.832776	Hepatocyte growth factor receptor	MET_HUMAN
	3.382	0.5637	0.826747	Insulin-like growth factor IA	P01343
	3.769	0.5385	0.82538	Nuclear receptor subfamily 1 group I member 2	NR1I2_HUMAN
	3.802	0.5432	0.824358	Thyroid hormone receptor alpha	P10827
	3.754	0.4171	0.813726	Serine/threonine-protein phosphatase PP1-gamma catalytic subunit	P36873
	3.549	0.507	0.812263	Neutrophil gelatinase-associated lipocalin	NGAL_HUMAN
**4alpha,24-dimethylcholesta-7,24-dienol**	3.953	0.9883	2.77026	Nuclear receptor ROR-alpha	RORA_HUMAN
	4.088	0.6813	2.30725	Oxysterols receptor LXR-alpha	NR1H3_HU

					MAN
	4.266	0.8533	2.10781	Estradiol 17-beta-dehydrogenase 1	P14061
	3.687	0.7373	2.06189	Alcohol dehydrogenase 1C	P00326
	3.852	0.7704	2.06118	Aldo-keto reductase family 1 member C1	Q04828
	4.233	0.6047	2.0339	Lanosterol synthase	ERG7_HUMAN
	4.577	0.6538	2.03303	Sulfotransferase family cytosolic 2B member 1	O00204
	4.167	0.8335	2.02997	SEC14-like protein 2	O76054
	3.71	0.6183	1.9444	Ganglioside GM2 activator	P17900
	3.801	0.7601	1.93644	Bile acid receptor	NR1H4_HUMAN
	3.969	0.6614	1.85014	Retinol-binding protein 4	RET4_HUMAN
	3.611	0.9028	1.79288	Mineralocorticoid receptor	MCR_HUMAN
	4.057	0.6762	1.76725	E3 ubiquitin-protein ligase Mdm2	MDM2_HUMAN
	3.913	0.559	1.75378	72 kDa type IV collagenase	MMP2_HUMAN
	4.272	0.5341	1.74978	Ferrochelatase, mitochondrial	P22830
	3.743	0.6238	1.74441	Tyrosine-protein phosphatase non-receptor type 1	PTN1_HUMAN
	3.863	0.7725	1.65304	Corticosteroid 11-beta-dehydrogenase isozyme 1	DHI1_HUMAN
	3.978	0.6629	1.63658	Trafficking protein particle complex subunit 3	O43617
	3.582	0.5971	1.61758	Renin	RENI_HUMAN
	3.579	0.8948	1.6117	Progesterone receptor	PRGR_HUMAN
	3.566	0.8916	1.59059	Collagenase 3	MMP13_HUMAN
	3.797	0.9493	1.57558	Vitamin D-binding protein	VTDB_HUMAN
	3.809	0.6348	1.5436	Ephrin type-B receptor 4	EPHB4_HUMAN
	4.29	0.715	1.54072	Sex hormone-binding globulin	SHBG_HUMAN

	4.114	0.5142	1.53607	Retinoic acid receptor alpha	RARA_HUMAN
	3.522	0.7045	1.51421	Glutathione S-transferase P	GSTP1_HUMAN
	3.727	0.7453	1.49003	Calmodulin	CALM_HUMAN
	3.705	0.741	1.48299	Prothrombin	THRB_HUMAN
	4.689	0.521	1.45256	Dual specificity mitogen-activated protein kinase kinase 1	MP2K1_HUMAN
	4.085	0.5836	1.42356	Estrogen sulfotransferase	ST1E1_HUMAN
	4.148	0.5926	1.40586	Gastrotropin	FABP6_HUMAN
	3.907	0.5582	1.39184	Cytochrome P450 2C8	CP2C8_HUMAN
	3.813	0.5447	1.38035	Retinoic acid receptor gamma	RARG_HUMAN
	3.71	0.53	1.2979	Interleukin-2	IL2_HUMAN
	4.298	0.3907	1.29625	Phosphatidylcholine transfer protein	Q9UKL6
	3.773	0.6288	1.28736	Glutathione S-transferase A1	P08263
	3.975	0.5678	1.28714	Hepatocyte nuclear factor 4-gamma	Q14541
	4.267	0.4267	1.27668	Vitamin D3 receptor	VDR_HUMAN
	3.333	0.8332	1.23699	Troponin C, slow skeletal and cardiac muscles	P63316
	4.28	0.4755	1.23699	Retinoic acid receptor beta	RARB_HUMAN
	3.632	0.7264	1.23333	Tyrosine-protein phosphatase non-receptor type 11	PTN11_HUMAN
	4.196	0.3814	1.16575	Serine/threonine-protein phosphatase PP1-gamma catalytic subunit	P36873
	4.104	0.5863	1.15247	Fatty acid-binding protein, brain	FABP7_HUMAN
	4.009	0.3084	1.11616	Endothelial protein C receptor	EPCR_HUMAN
	3.515	0.4394	1.11342	Heat shock protein HSP 90-beta	P08238

	3.498	0.8745	1.08991	Kinesin-like protein KIF11	KIF11_HUMAN
	3.668	0.6114	1.0548	Tyrosine-protein kinase SYK	KSYK_HUMAN
	3.476	0.5794	1.0364	Flavin reductase	P30043
	3.601	0.5144	1.02115	Protein farnesyltransferase/geranylgeranyltransferase type-1 subunit alpha	FNTA_HUMAN
	3.642	0.5202	1.01466	Tyrosine-protein kinase ZAP-70	ZAP70_HUMAN
	3.769	0.4188	0.99552	Growth factor receptor-bound protein 2	GRB2_HUMAN
	5.574	0.5574	0.995124	Cellular retinoic acid-binding protein 2	P29373
	3.551	0.7103	0.972897	Wiskott-Aldrich syndrome protein	WASP_HUMAN
	3.614	0.4517	0.904167	Proto-oncogene tyrosine-protein kinase ABL1	P00519
	3.509	0.5848	0.867275	Oxysterols receptor LXR-beta	NR1H2_HUMAN
	3.598	0.5996	0.818196	Glutathione-requiring prostaglandin D synthase	PTGD2_HUMAN
	3.361	0.6722	0.803683	Alpha-tocopherol transfer protein	P49638
**6-Fluoroindole-7-Dehydrocholesterol**	4.533	0.7555	2.72441	Trafficking protein particle complex subunit 3	O43617
	3.924	0.981	2.69583	Nuclear receptor ROR-alpha	RORA_HUMAN
	4.439	0.7399	2.46717	Adenosine kinase	P55263
	4.439	0.6342	2.41211	Cytochrome P450 2C8	CP2C8_HUMAN
	3.883	0.6472	2.37424	Ganglioside GM2 activator	P17900
	4.134	0.8268	1.96749	SEC14-like protein 2	O76054
	3.792	0.7584	1.91588	Bile acid receptor	NR1H4_HUMAN
	3.979	0.6631	1.87319	Retinol-binding protein 4	RET4_HUMAN
	5.003	0.5559	1.8576	Dual specificity mitogen-activated protein kinase kinase 1	MP2K1_HUMAN
	3.675	0.9188	1.84156	Collagenase 3	MMP13_HUMAN
	3.9	0.65	1.81041	Nuclear receptor subfamily 1	NR1I3_HU

				group I member 3	MAN
	3.651	0.9129	1.80818	Progesterone receptor	PRGR_HUMAN
	3.996	0.5708	1.75265	Retinoic acid receptor gamma	RARG_HUMAN
	4.369	0.6241	1.71223	Sulfotransferase family cytosolic 2B member 1	O00204
	3.841	0.9603	1.68748	Vitamin D-binding protein	VTDB_HUMAN
	3.809	0.5441	1.64175	Proto-oncogene tyrosine-protein kinase LCK	LCK_HUMAN
	3.853	0.5505	1.63191	Basic fibroblast growth factor receptor 1	FGFR1_HUMAN
	3.758	0.6263	1.6311	Receptor tyrosine-protein kinase erbB-4	ERBB4_HUMAN
	3.77	0.5386	1.62825	Tyrosine-protein phosphatase non-receptor type 1	PTN1_HUMAN
	3.536	0.7073	1.5515	Glutathione S-transferase P	GSTP1_HUMAN
	3.613	0.7225	1.52679	Aldo-keto reductase family 1 member C1	Q04828
	4.432	0.4432	1.51896	Vitamin D3 receptor	VDR_HUMAN
	3.844	0.6406	1.4368	Glutathione S-transferase A1	P08263
	3.761	0.7522	1.4245	Corticosteroid 11-beta-dehydrogenase isozyme 1	DHI1_HUMAN
	4.19	0.6984	1.3939	Sex hormone-binding globulin	SHBG_HUMAN
	3.843	0.7687	1.30172	Estradiol 17-beta-dehydrogenase 1	P14061
	3.88	0.4311	1.27712	Serine/threonine-protein phosphatase PP1-gamma catalytic subunit	P36873
	3.649	0.5212	1.25038	72 kDa type IV collagenase	MMP2_HUMAN
	4.03	0.5758	1.24675	Mitogen-activated protein kinase 10	MK10_HUMAN
	3.536	0.5893	1.23593	Poly [ADP-ribose] polymerase 1	P09874
	3.951	0.4938	1.22427	Ferrochelatase, mitochondrial	P22830
	3.511	0.5852	1.22054	Alpha-1-antitrypsin	A1AT_HUMAN

	3.34	0.8351	1.21657	Mineralocorticoid receptor	MCR_HUMAN
	3.723	0.5318	1.18716	Insulin-like growth factor IA	P01343
	3.785	0.5407	1.18624	Lanosterol synthase	ERG7_HUMAN
	3.598	0.7196	1.15234	Tyrosine-protein phosphatase non-receptor type 11	PTN11_HUMAN
	3.963	0.5662	1.10757	Gastrotropin	FABP6_HUMAN
	3.605	0.6008	1.07855	Oxysterols receptor LXR-beta	NR1H2_HUMAN
	3.852	0.5503	1.0637	Hepatocyte nuclear factor 4-gamma	Q14541
	3.976	0.3058	1.06215	Endothelial protein C receptor	EPCR_HUMAN
	3.48	0.58	1.04553	Flavin reductase	P30043
	4.121	0.4579	1.01216	Retinoic acid receptor beta	RARB_HUMAN
	3.516	0.586	0.97964	Oxysterols receptor LXR-alpha	NR1H3_HUMAN
	3.312	0.6623	0.968733	Steroid hormone receptor ERR1	P11474
	5.512	0.5512	0.954851	Cellular retinoic acid-binding protein 2	P29373
	3.319	0.8296	0.927031	ADAM 17	ADA17_HUMAN
	3.463	0.3848	0.918682	Phosphatidylinositol 3-kinase regulatory subunit alpha	P85A_HUMAN
	3.463	0.4948	0.907722	Serine/threonine-protein kinase 6	O14965
	3.629	0.6049	0.876361	E3 ubiquitin-protein ligase Mdm2	MDM2_HUMAN
	3.934	0.562	0.876244	Fatty acid-binding protein, brain	FABP7_HUMAN
	4.266	0.4266	0.844636	Retinoic acid receptor RXR-beta	RXRB_HUMAN
	3.3	0.66	0.827588	cAMP-dependent protein kinase catalytic subunit alpha	P00517
	3.247	0.6494	0.825318	Hepatocyte growth factor receptor	P08581
	3.365	0.6731	0.821592	Phospholipase A2, membrane associated	P14555
	3.568	0.446	0.818643	Proto-oncogene tyrosine-protein	P00519

				kinase ABL1	
	3.715	0.5308	0.800218	Estrogen sulfotransferase	ST1E1_HUMAN
**daucosterol_qt**	4.237	0.8475	3.34326	Histone deacetylase 8	Q9BY41
	4.466	0.7444	3.19072	Oxysterols receptor LXR-alpha	NR1H3_HUMAN
	4.431	0.7385	2.55004	E3 ubiquitin-protein ligase Mdm2	MDM2_HUMAN
	4.202	0.8404	2.09313	SEC14-like protein 2	O76054
	4.226	0.7043	2.02377	Adenosine kinase	P55263
	5.096	0.5662	1.99267	Dual specificity mitogen-activated protein kinase kinase 1	MP2K1_HUMAN
	4.111	0.6852	1.9812	Glutathione S-transferase A1	P08263
	4.786	0.5318	1.97351	Retinoic acid receptor beta	RARB_HUMAN
	4.147	0.6911	1.95767	Trafficking protein particle complex subunit 3	O43617
	4.708	0.428	1.93756	Phosphatidylcholine transfer protein	Q9UKL6
	3.946	0.6577	1.89243	Nuclear receptor subfamily 1 group I member 3	NR1I3_HUMAN
	4.342	0.5427	1.86474	Ferrochelatase, mitochondrial	P22830
	4.479	0.6399	1.77027	Fatty acid-binding protein, brain	FABP7_HUMAN
	4.118	0.6863	1.75785	Glutathione-requiring prostaglandin D synthase	PTGD2_HUMAN
	4.584	0.4584	1.74351	Vitamin D3 receptor	VDR_HUMAN
	3.766	0.6277	1.73355	Flavin reductase	P30043
	3.597	0.5996	1.72213	cGMP-specific 3,5-cyclic phosphodiesterase	PDE5A_HUMAN
	3.651	0.7302	1.65402	Dipeptidyl peptidase 4	DPP4_HUMAN
	4.317	0.3321	1.63593	Endothelial protein C receptor	EPCR_HUMAN
	3.509	0.8772	1.54416	Nuclear receptor ROR-alpha	RORA_HUMAN
	3.723	0.5318	1.53092	Protein S100-A9	S10A9_HUMAN
	3.809	0.6348	1.51854	Ephrin type-B receptor 4	EPHB4_HUMAN

	3.81	0.635	1.49388	Retinol-binding protein 4	RET4_HUMAN
	3.74	0.7481	1.48859	Calmodulin	CALM_HUMAN
	3.608	0.7216	1.47849	Aldo-keto reductase family 1 member C1	Q04828
	3.897	0.6495	1.4615	[Pyruvate dehydrogenase [lipoamide]] kinase isozyme 2, mitochondrial	PDK2_HUMAN
	4.432	0.5539	1.46073	Glucocorticoid receptor	GCR_HUMAN
	3.763	0.9407	1.45762	Vitamin D-binding protein	VTDB_HUMAN
	3.683	0.4604	1.4521	Heat shock protein HSP 90-beta	P08238
	3.742	0.6236	1.45022	Mitogen-activated protein kinase 10	P53779
	3.848	0.5497	1.42265	Insulin-like growth factor IA	P01343
	3.932	0.5618	1.42189	Cytochrome P450 2C8	CP2C8_HUMAN
	3.724	0.5321	1.3666	72 kDa type IV collagenase	MMP2_HUMAN
	4.407	0.5508	1.35724	Fatty acid-binding protein, heart	FABPH_HUMAN
	3.459	0.8647	1.35037	Cell division protein kinase 2	P24941
	4.668	0.4668	1.3457	Retinoic acid receptor RXR-beta	RXRB_HUMAN
	3.781	0.4727	1.32016	Stromelysin-1	MMP3_HUMAN
	3.564	0.7128	1.31862	Bile acid receptor	NR1H4_HUMAN
	4.078	0.5825	1.28271	Gastrotropin	FABP6_HUMAN
	3.468	0.6936	1.27674	Steroid hormone receptor ERR1	P11474
	3.71	0.742	1.27661	Corticosteroid 11-beta-dehydrogenase isozyme 1	DHI1_HUMAN
	3.831	0.7661	1.2555	Estradiol 17-beta-dehydrogenase 1	P14061
	3.895	0.5565	1.23954	Nuclear receptor subfamily 1 group I member 2	NR1I2_HUMAN
	3.741	0.5345	1.20729	Retinoic acid receptor gamma	RARG_HUMAN

	3.878	0.4309	1.19489	Growth factor receptor-bound protein 2	GRB2_HUMAN
	3.573	0.5955	1.18775	Peroxisome proliferator-activated receptor alpha	PPARA_HUMAN
	3.593	0.7186	1.14746	Prothrombin	THRB_HUMAN
	3.64	0.728	1.14206	Wiskott-Aldrich syndrome protein	WASP_HUMAN
	3.571	0.3968	1.13792	Phosphatidylinositol 3-kinase regulatory subunit alpha	P85A_HUMAN
	3.642	0.5203	1.12962	Interleukin-2	IL2_HUMAN
	3.345	0.8362	1.12752	Coagulation factor X	FA10_HUMAN
	3.313	0.8283	1.11163	Mineralocorticoid receptor	MCR_HUMAN
	4.115	0.5144	1.10249	Histamine N-methyltransferase	P50135
	3.622	0.6037	1.08018	Oxysterols receptor LXR-beta	NR1H2_HUMAN
	3.372	0.6743	1.0492	Glutathione S-transferase P	GSTP1_HUMAN
	3.715	0.5307	1.02585	Lanosterol synthase	ERG7_HUMAN
	3.538	0.7076	0.966425	Tyrosine-protein phosphatase non-receptor type 11	PTN11_HUMAN
	3.59	0.5129	0.95564	Protein farnesyltransferase/geranylgeranyltransferase type-1 subunit alpha	FNTA_HUMAN
	3.37	0.6739	0.89053	Methionine aminopeptidase 2	AMPM2_HUMAN
	3.679	0.4088	0.87056	Cathepsin B	CATB_HUMAN
	3.734	0.6224	0.855771	Alcohol dehydrogenase class-3	ADHX_HUMAN
**(-)-Tabernemontanine**	3.697	0.6162	4.36369	NONE	NONE
	3.609	0.7218	4.05271	Basic fibroblast growth factor receptor 1	FGFR1_HUMAN
	3.212	0.8031	3.06226	Annexin A5	ANXA5_HUMAN
	3.437	0.4297	2.44903	Proto-oncogene tyrosine-protein kinase ABL1	P00519

	3.097	0.4424	2.40941	3-hydroxy-3-methylglutaryl-coenzyme A reductase	HMDH_HUMAN
	3.515	0.4393	2.36502	Retinoic acid receptor alpha	RARA_HUMAN
	3.369	0.3063	2.08293	Caspase-1	CASP1_HUMAN
	2.974	0.7434	2.03611	Serine/threonine-protein kinase PLK1	P53350
	2.932	0.5863	1.91071	Glycogen synthase kinase-3 beta	GSK3B_HUMAN
	3.172	0.3172	1.78951	tRNA (cytosine-5-)-methyltransferase	O14717
	2.939	0.5877	1.76003	Serine protease hepsin	P05981
	2.886	0.9619	1.74899	P-selectin	LYAM3_HUMAN
	2.864	0.4773	1.73929	B transferase	Q9NY01
	2.907	0.5814	1.73291	Aldo-keto reductase family 1 member C3	AK1C3_HUMAN
	2.873	0.5746	1.66159	Neutrophil collagenase	MMP8_HUMAN
	2.89	0.578	1.55969	Macrophage metalloelastase	MMP12_HUMAN
	2.776	0.9254	1.51656	Probable ATP-dependent RNA helicase DDX6	DDX6_HUMAN
	2.82	0.7049	1.45462	Serine/threonine-protein kinase Chk1	CHK1_HUMAN
	2.804	0.9348	1.41636	Cyclin-dependent kinase 5 activator 1	CD5R1_HUMAN
	2.886	0.4123	1.40996	Baculoviral IAP repeat-containing protein 4	P98170
	2.806	0.3507	1.40483	Platelet glycoprotein Ib alpha chain	P07359
	2.892	0.482	1.39754	Insulin-like growth factor IA	P01343
	2.786	0.4644	1.39573	Fatty acid-binding protein, adipocyte	FABP4_HUMAN
	2.853	0.4755	1.38995	Fructose-bisphosphate aldolase A	ALDOA_HUMAN
	2.874	0.7184	1.38206	Glycine amidinotransferase, mitochondrial	GATM_HUMAN
	2.877	0.4796	1.36998	Eosinophil cationic protein	P12724
	2.845	0.4742	1.35313	Baculoviral IAP repeat-	Q96CA5

				containing protein 7	
	2.783	0.6957	1.33984	Heat shock protein HSP 90-alpha	HS90A_HUMAN
	3.568	0.5098	1.29087	Alcohol dehydrogenase class-3	ADHX_HUMAN
	3.001	0.5002	1.28204	Prostatic acid phosphatase	PPAP_HUMAN
	2.884	0.3204	1.27816	Pancreatic alpha-amylase	AMYP_HUMAN
	2.805	0.4674	1.24734	cGMP-specific 3,5-cyclic phosphodiesterase	PDE5A_HUMAN
	2.858	0.3573	1.24127	Beta-secretase 1	BACE1_HUMAN
	2.727	0.6817	1.20921	Cathepsin B	CATB_HUMAN
	2.82	0.564	1.18751	Fibroblast growth factor receptor 2	P21802
	2.788	0.5576	1.16331	Androgen receptor	ANDR_HUMAN
	2.81	0.562	1.14276	Tyrosine-protein kinase SYK	KSYK_HUMAN
	2.847	0.4067	1.14273	Dual specificity protein kinase CLK1	CLK1_HUMAN
	2.742	0.6856	1.12898	Prothrombin	THRB_HUMAN
	2.812	0.4017	1.11308	ADP-ribosyl cyclase 2	BST1_HUMAN
	2.818	0.5636	1.09368	cAMP-dependent protein kinase catalytic subunit alpha	P00517
	2.863	0.2603	1.07583	FK506-binding protein 3	FKBP3_HUMAN
	2.88	0.4115	1.06479	Nicotinamide mononucleotide adenylyltransferase 1	Q9HAN9
	2.857	0.4762	1.05618	Cytochrome P450 2C9	CP2C9_HUMAN
	2.883	0.3604	1.05617	Peptidyl-prolyl cis-trans isomerase FKBP1A	FKB1A_HUMAN
	2.77	0.554	1.04743	Cyclin-A2	CCNA2_HUMAN
	2.91	0.4851	1.04409	Ephrin type-B receptor 4	EPHB4_HUMAN

	2.737	0.6842	1.03548	Caspase-7	CASP7_HUMAN
	2.962	0.3291	1.02864	Lactoylglutathione lyase	LGUL_HUMAN
	2.819	0.3132	1.01931	Caspase-3	CASP3_HUMAN
	2.774	0.5549	1.00987	Lanosterol synthase	ERG7_HUMAN
	3.221	0.2928	1.00402	Serine/threonine-protein phosphatase PP1-gamma catalytic subunit	P36873
	2.741	0.6853	0.99639	cAMP-specific 3,5-cyclic phosphodiesterase 4B	PDE4B_HUMAN
	2.8	0.56	0.968496	Kinesin-like protein KIF11	KIF11_HUMAN
	2.723	0.3404	0.947762	Phosphatidylinositol 3-kinase regulatory subunit alpha	P85A_HUMAN
	2.815	0.4692	0.938664	Insulin-like growth factor 1 receptor	IGF1R_HUMAN
	2.869	0.5738	0.931797	Serum albumin	ALBU_HUMAN
	2.759	0.5518	0.920381	Epidermal growth factor receptor	EGFR_HUMAN
	2.89	0.578	0.916265	Corticosteroid 11-beta-dehydrogenase isozyme 1	DHI1_HUMAN
	2.959	0.4227	0.909112	B-Raf proto-oncogene serine/threonine-protein kinase	BRAF1_HUMAN
	2.707	0.3384	0.90801	Coagulation factor XI	FA11_HUMAN
	2.708	0.4513	0.902328	Alpha-amylase 1	P04745
	2.777	0.5554	0.867773	Mitogen-activated protein kinase 14	Q16539
	2.898	0.483	0.866525	Vascular endothelial growth factor receptor 2	VGFR2_HUMAN
	2.881	0.3602	0.859488	Chymase	CMA1_HUMAN
	2.737	0.6844	0.858849	Kynurenine--oxoglutarate transaminase 1	KAT1_HUMAN
	2.827	0.4038	0.854886	Thymidylate kinase	KTHY_HUMAN
	2.765	0.395	0.853693	Cathepsin F	Q9UBX1

	2.904	0.4148	0.842733	Serine--pyruvate aminotransferase	SPYA_HUMAN
	2.712	0.5424	0.806698	Phosphatidylinositol-4,5-bisphosphate 3-kinase catalytic subunit gamma isoform	PK3CG_HUMAN
**(-)-Caryophyllene oxide**	4.355	0.6221	3.39981	Glutathione S-transferase P	GSTP1_HUMAN
	3.665	0.5236	2.46403	Protein farnesyltransferase/geranylgeranyltransferase type-1 subunit alpha	FNTA_HUMAN
	3.585	0.5976	2.27309	Nuclear receptor subfamily 1 group I member 3	NR1I3_HUMAN
	3.645	0.4557	2.16241	Stromelysin-1	MMP3_HUMAN
	4.115	0.5144	2.06365	Glucocorticoid receptor	GCR_HUMAN
	3.564	0.5091	1.91139	Retinoic acid receptor gamma	RARG_HUMAN
	3.429	0.4899	1.60924	Peroxisome proliferator-activated receptor gamma	PPARG_HUMAN
	3.257	0.4071	1.48496	Vitamin D3 receptor	VDR_HUMAN
	3.65	0.5214	1.44	Sulfotransferase family cytosolic 2B member 1	O00204
	3.59	0.4487	1.37038	Peptidyl-prolyl cis-trans isomerase FKBP1A	FKB1A_HUMAN
	3.539	0.5056	1.34846	Estrogen sulfotransferase	ST1E1_HUMAN
	2.997	0.9991	1.33362	Aldo-keto reductase family 1 member C2	AK1C2_HUMAN
	3.201	0.5335	1.26976	Oxysterols receptor LXR-beta	NR1H2_HUMAN
	2.998	0.9994	1.2526	Integrin alpha-L	ITAL_HUMAN
	3.673	0.3339	1.2146	Serine/threonine-protein phosphatase PP1-gamma catalytic subunit	P36873
	3.184	0.6367	1.20441	Alpha-tocopherol transfer protein	P49638
	3.654	0.406	1.12589	Retinoic acid receptor beta	RARB_HUMAN
	2.999	0.7497	1.07985	Bile salt sulfotransferase	Q06520

	3.321	0.5534	1.06624	Glutathione-requiring prostaglandin D synthase	PTGD2_HUMAN
	3.82	0.382	1.05081	Retinoic acid receptor RXR-beta	RXRB_HUMAN
	2.951	0.7377	1.04907	Retinoic acid receptor RXR-alpha	RXRA_HUMAN
	2.98	0.7451	1.04781	Progesterone receptor	PRGR_HUMAN
	3.252	0.542	1.00364	Glutathione S-transferase A1	P08263
	2.962	0.7404	1.0014	Group 10 secretory phospholipase A2	O15496
	2.987	0.7468	0.986953	Estrogen receptor	ESR1_HUMAN
	2.992	0.5983	0.977997	Cell division protein kinase 2	P24941
	2.957	0.7393	0.975276	Sex hormone-binding globulin	SHBG_HUMAN
	2.966	0.7416	0.956508	Estradiol 17-beta-dehydrogenase 1	P14061
	2.955	0.5911	0.955974	Estrogen receptor beta	Q92731
	2.996	0.5992	0.934575	Tyrosine-protein phosphatase non-receptor type 1	P18031
	2.897	0.7243	0.888776	NONE	NONE
	2.879	0.9598	0.878174	Cytochrome P450 19A1	P11511
	2.871	0.9569	0.858466	Phosphatidylinositol-4,5-bisphosphate 3-kinase catalytic subunit gamma isoform	PK3CG_HUMAN
	2.905	0.7262	0.83293	Proto-oncogene tyrosine-protein kinase Src	SRC_HUMAN
	2.884	0.9614	0.821444	Caspase-7	CASP7_HUMAN
	3.644	0.4049	0.814563	Dual specificity mitogen-activated protein kinase kinase 1	MP2K1_HUMAN
	2.845	0.9483	0.811606	Serum albumin	ALBU_HUMAN
	2.969	0.7423	0.811216	Transthyretin	TTHY_HUMAN
**lanost-8-enol**	5.228	0.7469	3.47996	Lanosterol synthase	ERG7_HUMAN
	5.294	0.6618	3.03785	Ferrochelatase, mitochondrial	P22830
	5.082	0.7261	2.56619	Gastrotropin	FABP6_HUMAN

	4.587	0.7645	2.48647	Trafficking protein particle complex subunit 3	O43617
	4.324	0.5405	2.48139	Vitamin D3 receptor	VDR_HUMAN
	3.921	0.9803	2.38681	Nuclear receptor ROR-alpha	RORA_HUMAN
	3.954	0.9885	2.20409	Transthyretin	TTHY_HUMAN
	3.925	0.785	1.96287	Bile acid receptor	NR1H4_HUMAN
	3.787	0.6311	1.88612	Ganglioside GM2 activator	P17900
	3.925	0.5607	1.77782	Protein S100-A9	S10A9_HUMAN
	3.929	0.7858	1.77064	Prothrombin	THRB_HUMAN
	4.171	0.8341	1.76913	SEC14-like protein 2	O76054
	3.841	0.6402	1.68962	Flavin reductase	P30043
	4.453	0.6361	1.58861	Sulfotransferase family cytosolic 2B member 1	O00204
	4.128	0.5897	1.56258	Cytochrome P450 2C8	CP2C8_HUMAN
	4.643	0.4221	1.55167	Phosphatidylcholine transfer protein	Q9UKL6
	3.962	0.4953	1.49543	Proto-oncogene tyrosine-protein kinase ABL1	P00519
	4.683	0.5853	1.49501	Fatty acid-binding protein, heart	FABPH_HUMAN
	3.98	0.5686	1.47921	Retinoic acid receptor gamma	RARG_HUMAN
	3.826	0.6376	1.45792	Oxysterols receptor LXR-alpha	NR1H3_HUMAN
	4.965	0.4965	1.44645	Retinoic acid receptor RXR-beta	RXRB_HUMAN
	3.576	0.8939	1.44613	Mitogen-activated protein kinase 14	Q16539
	3.873	0.7747	1.43166	Corticosteroid 11-beta-dehydrogenase isozyme 1	DHI1_HUMAN
	3.772	0.4191	1.42517	Phosphatidylinositol 3-kinase regulatory subunit alpha	P85A_HUMAN
	3.735	0.747	1.42193	Alpha-tocopherol transfer protein	P49638
	3.797	0.7593	1.4151	Calmodulin	CALM_HU

					MAN
	3.697	0.4108	1.41227	Bcl-2-like protein 1	Q07817
	3.62	0.7241	1.41075	cAMP-dependent protein kinase catalytic subunit alpha	P00517
	3.698	0.6164	1.4002	Poly [ADP-ribose] polymerase 1	P09874
	4.171	0.5959	1.39771	Hepatocyte nuclear factor 4-gamma	Q14541
	3.934	0.6556	1.3961	Histone deacetylase 8	HDAC8_HUMAN
	3.813	0.7626	1.35507	Mineralocorticoid receptor	MCR_HUMAN
	3.8	0.6333	1.35162	Nuclear receptor subfamily 1 group I member 3	NR1I3_HUMAN
	3.657	0.6095	1.34376	Alpha-1-antitrypsin	A1AT_HUMAN
	3.552	0.7103	1.33192	Steroid hormone receptor ERR1	P11474
	4.757	0.5285	1.29873	Dual specificity mitogen-activated protein kinase kinase 1	MP2K1_HUMAN
	3.783	0.9459	1.28948	Vitamin D-binding protein	VTDB_HUMAN
	3.792	0.632	1.26786	Oxysterols receptor LXR-beta	NR1H2_HUMAN
	3.864	0.6439	1.25283	Glutathione S-transferase A1	P08263
	3.664	0.458	1.24531	Heat shock protein HSP 90-beta	P08238
	4.956	0.6196	1.22453	Retinol-binding protein 4	RET4_HUMAN
	4.38	0.3982	1.2096	Serine/threonine-protein phosphatase PP1-gamma catalytic subunit	P36873
	3.474	0.6948	1.18938	Glutathione S-transferase P	GSTP1_HUMAN
	3.636	0.606	1.17214	Peroxisome proliferator-activated receptor alpha	PPARA_HUMAN
	3.707	0.5296	1.17154	Neutrophil gelatinase-associated lipocalin	NGAL_HUMAN
	3.513	0.2702	1.15369	Amine oxidase [flavin-containing] A	AOFA_HUMAN
	3.687	0.5267	1.14817	72 kDa type IV collagenase	MMP2_HUMAN
	3.526	0.5877	1.13912	Serine/threonine-protein kinase Chk1	CHK1_HUMAN

	4.355	0.4838	1.12678	Retinoic acid receptor beta	RARB_HUMAN
	3.481	0.6962	1.06897	Aldo-keto reductase family 1 member C1	Q04828
	3.903	0.4337	1.04185	Growth factor receptor-bound protein 2	GRB2_HUMAN
	3.632	0.7263	1.02759	Tyrosine-protein phosphatase non-receptor type 11	PTN11_HUMAN
	3.963	0.5662	1.02131	Estrogen sulfotransferase	ST1E1_HUMAN
	3.278	0.8196	1.01497	Androgen receptor	ANDR_HUMAN
	3.615	0.5164	1.00858	Basic fibroblast growth factor receptor 1	FGFR1_HUMAN
	4.144	0.5919	1.00048	Fatty acid-binding protein, brain	FABP7_HUMAN
	3.761	0.7523	0.9565	Estradiol 17-beta-dehydrogenase 1	P14061
	3.865	0.6442	0.926928	Alcohol dehydrogenase class-3	ADHX_HUMAN
	3.605	0.7211	0.90348	Wiskott-Aldrich syndrome protein	WASP_HUMAN
	3.645	0.6075	0.8988	Tyrosine-protein kinase ITK/TSK	Q08881
	3.732	0.622	0.891734	E3 ubiquitin-protein ligase Mdm2	MDM2_HUMAN
	3.369	0.5615	0.869351	Histo-blood group ABO system transferase	P16442
	3.682	0.6136	0.839754	[Pyruvate dehydrogenase [lipoamide]] kinase isozyme 2, mitochondrial	PDK2_HUMAN
	3.694	0.5277	0.822754	Protein kinase C theta type	KPCT_HUMAN
	3.69	0.615	0.818662	Glutathione-requiring prostaglandin D synthase	PTGD2_HUMAN
	3.938	0.3029	0.802891	Endothelial protein C receptor	EPCR_HUMAN
**Lophenol**	3.915	0.9786	2.61283	Nuclear receptor ROR-alpha	RORA_HUMAN
	4.408	0.8816	2.45019	SEC14-like protein 2	O76054
	4.411	0.7351	2.42424	Trafficking protein particle complex subunit 3	O43617

	4	0.8	2.3742	Vascular endothelial growth factor receptor 2	P35968
	4.386	0.8773	2.28183	Estradiol 17-beta-dehydrogenase 1	P14061
	4.407	0.5509	2.26239	Caspase-3	CASP3_HUMAN
	3.805	0.6341	2.13066	Ganglioside GM2 activator	P17900
	4.605	0.6579	2.02638	Sulfotransferase family cytosolic 2B member 1	O00204
	3.679	0.7358	1.9913	Alcohol dehydrogenase 1C	P00326
	3.705	0.9262	1.95378	Mineralocorticoid receptor	MCR_HUMAN
	4.176	0.5966	1.87758	Lanosterol synthase	ERG7_HUMAN
	4.173	0.5962	1.85105	Cytochrome P450 2C8	CP2C8_HUMAN
	4.361	0.5451	1.84924	Ferrochelatase, mitochondrial	P22830
	3.711	0.7422	1.80236	cAMP-dependent protein kinase catalytic subunit alpha	P00517
	3.658	0.9146	1.77955	Progesterone receptor	PRGR_HUMAN
	3.811	0.4234	1.71045	Phosphatidylinositol 3-kinase regulatory subunit alpha	P85A_HUMAN
	3.767	0.7535	1.67896	Androgen receptor	ANDR_HUMAN
	3.81	0.5443	1.67729	Tyrosine-protein phosphatase non-receptor type 1	PTN1_HUMAN
	3.726	0.621	1.58504	Oxysterols receptor LXR-beta	NR1H2_HUMAN
	3.586	0.7171	1.5497	Steroid hormone receptor ERR1	P11474
	4.236	0.6052	1.50562	Gastrotropin	FABP6_HUMAN
	4.761	0.529	1.50004	Dual specificity mitogen-activated protein kinase kinase 1	MP2K1_HUMAN
	3.604	0.7207	1.46966	Aldo-keto reductase family 1 member C1	Q04828
	3.671	0.7343	1.45358	Alpha-tocopherol transfer protein	P49638
	4.256	0.7094	1.44996	Sex hormone-binding globulin	SHBG_HUMAN
	3.507	0.8768	1.42047	Collagenase 3	MMP13_HUMAN

	3.604	0.7207	1.41824	Bile acid receptor	NR1H4_HUMAN
	3.509	0.5848	1.39239	Renin	RENI_HUMAN
	3.719	0.9298	1.3342	Vitamin D-binding protein	VTDB_HUMAN
	4.053	0.579	1.32983	Estrogen sulfotransferase	ST1E1_HUMAN
	3.705	0.5293	1.32242	72 kDa type IV collagenase	MMP2_HUMAN
	4.873	0.6092	1.32078	Retinol-binding protein 4	RET4_HUMAN
	3.983	0.5689	1.26066	Hepatocyte nuclear factor 4-gamma	Q14541
	3.765	0.6275	1.23094	Glutathione S-transferase A1	P08263
	3.693	0.5276	1.22776	Interleukin-2	IL2_HUMAN
	3.846	0.6411	1.21481	Adenosine kinase	P55263
	3.583	0.5972	1.19562	Receptor tyrosine-protein kinase erbB-4	ERBB4_HUMAN
	3.626	0.7251	1.17887	Tyrosine-protein phosphatase non-receptor type 11	PTN11_HUMAN
	3.457	0.2659	1.16595	Amine oxidase [flavin-containing] A	AOFA_HUMAN
	3.708	0.5297	1.13128	Retinoic acid receptor gamma	RARG_HUMAN
	3.295	0.8238	1.1269	Troponin C, slow skeletal and cardiac muscles	P63316
	5.777	0.5777	1.10365	Cellular retinoic acid-binding protein 2	P29373
	3.8	0.76	1.07031	Bile salt sulfotransferase	Q06520
	4.147	0.4147	1.06668	Vitamin D3 receptor	VDR_HUMAN
	3.571	0.5101	1.043	Macrophage metalloelastase	MMP12_HUMAN
	3.691	0.4614	1.01375	Proto-oncogene tyrosine-protein kinase ABL1	P00519
	3.589	0.7179	0.999792	Corticosteroid 11-beta-dehydrogenase isozyme 1	DHI1_HUMAN
	4.122	0.458	0.976261	Retinoic acid receptor beta	RARB_HUMAN

	3.796	0.6327	0.963583	Alcohol dehydrogenase class-3	ADHX_HUMAN
	3.884	0.5549	0.955001	Mitogen-activated protein kinase 10	MK10_HUMAN
	3.321	0.6642	0.938033	Prothrombin	THRB_HUMAN
	3.671	0.6119	0.919301	Glutathione-requiring prostaglandin D synthase	PTGD2_HUMAN
	3.887	0.299	0.874417	Endothelial protein C receptor	EPCR_HUMAN
	3.797	0.7594	0.871199	S-methyl-5-thioadenosine phosphorylase	Q13126
	3.585	0.5122	0.867959	Tyrosine-protein kinase ZAP-70	ZAP70_HUMAN
	3.46	0.5767	0.817229	Oxysterols receptor LXR-alpha	NR1H3_HUMAN

Table S3 Target proteins' corresponding pathways predicted by KEGG and PubMed

hsa01100 Metabolic pathways - Homo sapiens (human) (23)
hsa05200 Pathways in cancer - Homo sapiens (human) (13)
hsa04151 PI3K-Akt signaling pathway - Homo sapiens (human) (11)
hsa04014 Ras signaling pathway - Homo sapiens (human) (9)
hsa04068 FoxO signaling pathway - Homo sapiens (human) (7)
hsa04914 Progesterone-mediated oocyte maturation - Homo sapiens (human) (7)
hsa04510 Focal adhesion - Homo sapiens (human) (7)
hsa01521 EGFR tyrosine kinase inhibitor resistance - Homo sapiens (human) (7)
hsa04910 Insulin signaling pathway - Homo sapiens (human) (6)
hsa05418 Fluid shear stress and atherosclerosis - Homo sapiens (human) (6)
hsa04010 MAPK signaling pathway - Homo sapiens (human) (6)
hsa05145 Toxoplasmosis - Homo sapiens (human) (6)
hsa00980 Metabolism of xenobiotics by cytochrome P450 - Homo sapiens (human) (6)
hsa04110 Cell cycle - Homo sapiens (human) (5)
hsa04210 Apoptosis - Homo sapiens (human) (5)
hsa04114 Oocyte meiosis - Homo sapiens (human) (5)
hsa05205 Proteoglycans in cancer - Homo sapiens (human) (5)
hsa00270 Cysteine and methionine metabolism - Homo sapiens (human) (5)
hsa04217 Necroptosis - Homo sapiens (human) (5)
hsa05203 Viral carcinogenesis - Homo sapiens (human) (5)

hsa04621 NOD-like receptor signaling pathway - Homo sapiens (human) (5)
hsa05204 Chemical carcinogenesis - Homo sapiens (human) (5)
hsa04919 Thyroid hormone signaling pathway - Homo sapiens (human) (5)
hsa00140 Steroid hormone biosynthesis - Homo sapiens (human) (5)
hsa04015 Rap1 signaling pathway - Homo sapiens (human) (5)
hsa05222 Small cell lung cancer - Homo sapiens (human) (5)
hsa04722 Neurotrophin signaling pathway - Homo sapiens (human) (5)
hsa05215 Prostate cancer - Homo sapiens (human) (5)
hsa04931 Insulin resistance - Homo sapiens (human) (5)
hsa05169 Epstein-Barr virus infection - Homo sapiens (human) (5)
hsa05166 HTLV-I infection - Homo sapiens (human) (4)
hsa05160 Hepatitis C - Homo sapiens (human) (4)
hsa04215 Apoptosis - multiple species - Homo sapiens (human) (4)
hsa04657 IL-17 signaling pathway - Homo sapiens (human) (4)
hsa05167 Kaposi's sarcoma-associated herpesvirus infection - Homo sapiens (human) (4)
hsa04917 Prolactin signaling pathway - Homo sapiens (human) (4)
hsa04141 Protein processing in endoplasmic reticulum - Homo sapiens (human) (4)
hsa01524 Platinum drug resistance - Homo sapiens (human) (4)
hsa04728 Dopaminergic synapse - Homo sapiens (human) (4)
hsa05202 Transcriptional misregulation in cancer - Homo sapiens (human) (4)
hsa04550 Signaling pathways regulating pluripotency of stem cells - Homo sapiens (human) (4)
hsa05164 Influenza A - Homo sapiens (human) (4)
hsa04659 Th17 cell differentiation - Homo sapiens (human) (4)
hsa04218 Cellular senescence - Homo sapiens (human) (4)
hsa01522 Endocrine resistance - Homo sapiens (human) (4)
hsa04144 Endocytosis - Homo sapiens (human) (3)
hsa04660 T cell receptor signaling pathway - Homo sapiens (human) (3)
hsa05120 Epithelial cell signaling in Helicobacter pylori infection - Homo sapiens (human) (3)
hsa04370 VEGF signaling pathway - Homo sapiens (human) (3)
hsa04360 Axon guidance - Homo sapiens (human) (3)
hsa04261 Adrenergic signaling in cardiomyocytes - Homo sapiens (human) (3)
hsa05162 Measles - Homo sapiens (human) (3)
hsa04150 mTOR signaling pathway - Homo sapiens (human) (3)
hsa05168 Herpes simplex infection - Homo sapiens (human) (3)
hsa03320 PPAR signaling pathway - Homo sapiens (human) (3)
hsa04064 NF-kappa B signaling pathway - Homo sapiens (human) (3)
hsa04213 Longevity regulating pathway - multiple species - Homo sapiens (human) (3)
hsa05206 MicroRNAs in cancer - Homo sapiens (human) (3)
hsa00480 Glutathione metabolism - Homo sapiens (human) (3)

hsa04976 Bile secretion - Homo sapiens (human) (3)
hsa01230 Biosynthesis of amino acids - Homo sapiens (human) (3)
hsa04611 Platelet activation - Homo sapiens (human) (3)
hsa04211 Longevity regulating pathway - Homo sapiens (human) (3)
hsa00260 Glycine, serine and threonine metabolism - Homo sapiens (human) (3)
hsa05224 Breast cancer - Homo sapiens (human) (3)
hsa04932 Non-alcoholic fatty liver disease (NAFLD) - Homo sapiens (human) (3)
hsa04140 Autophagy - animal - Homo sapiens (human) (3)
hsa04920 Adipocytokine signaling pathway - Homo sapiens (human) (3)
hsa00982 Drug metabolism - cytochrome P450 - Homo sapiens (human) (3)
hsa04933 AGE-RAGE signaling pathway in diabetic complications - Homo sapiens (human) (3)
hsa04913 Ovarian steroidogenesis - Homo sapiens (human) (3)
hsa04310 Wnt signaling pathway - Homo sapiens (human) (3)
hsa04750 Inflammatory mediator regulation of TRP channels - Homo sapiens (human) (3)
hsa04142 Lysosome - Homo sapiens (human) (3)
hsa04012 ErbB signaling pathway - Homo sapiens (human) (3)
hsa04915 Estrogen signaling pathway - Homo sapiens (human) (3)
hsa04520 Adherens junction - Homo sapiens (human) (3)
hsa04270 Vascular smooth muscle contraction - Homo sapiens (human) (3)
hsa05131 Shigellosis - Homo sapiens (human) (3)
hsa05165 Human papillomavirus infection - Homo sapiens (human) (3)
hsa05221 Acute myeloid leukemia - Homo sapiens (human) (2)
hsa05010 Alzheimer's disease - Homo sapiens (human) (2)
hsa04024 cAMP signaling pathway - Homo sapiens (human) (2)
hsa04912 GnRH signaling pathway - Homo sapiens (human) (2)
hsa05034 Alcoholism - Homo sapiens (human) (2)
hsa04062 Chemokine signaling pathway - Homo sapiens (human) (2)
hsa04668 TNF signaling pathway - Homo sapiens (human) (2)
hsa04390 Hippo signaling pathway - Homo sapiens (human) (2)
hsa04152 AMPK signaling pathway - Homo sapiens (human) (2)
hsa05132 Salmonella infection - Homo sapiens (human) (2)
hsa00591 Linoleic acid metabolism - Homo sapiens (human) (2)
hsa00565 Ether lipid metabolism - Homo sapiens (human) (2)
hsa04080 Neuroactive ligand-receptor interaction - Homo sapiens (human) (2)
hsa05212 Pancreatic cancer - Homo sapiens (human) (2)
hsa04723 Retrograde endocannabinoid signaling - Homo sapiens (human) (2)
hsa04620 Toll-like receptor signaling pathway - Homo sapiens (human) (2)
hsa00860 Porphyrin and chlorophyll metabolism - Homo sapiens (human) (2)
hsa04060 Cytokine-cytokine receptor interaction - Homo sapiens (human) (2)

hsa04071 Sphingolipid signaling pathway - Homo sapiens (human) (2)
hsa04120 Ubiquitin mediated proteolysis - Homo sapiens (human) (2)
hsa05133 Pertussis - Homo sapiens (human) (2)
hsa04380 Osteoclast differentiation - Homo sapiens (human) (2)
hsa00230 Purine metabolism - Homo sapiens (human) (2)
hsa00590 Arachidonic acid metabolism - Homo sapiens (human) (2)
hsa04630 Jak-STAT signaling pathway - Homo sapiens (human) (2)
hsa04137 Mitophagy - animal - Homo sapiens (human) (2)
hsa00592 alpha-Linolenic acid metabolism - Homo sapiens (human) (2)
hsa00760 Nicotinate and nicotinamide metabolism - Homo sapiens (human) (2)
hsa00010 Glycolysis / Gluconeogenesis - Homo sapiens (human) (2)
hsa05161 Hepatitis B - Homo sapiens (human) (2)
hsa04972 Pancreatic secretion - Homo sapiens (human) (2)
hsa00071 Fatty acid degradation - Homo sapiens (human) (2)
hsa00564 Glycerophospholipid metabolism - Homo sapiens (human) (2)
hsa05220 Chronic myeloid leukemia - Homo sapiens (human) (2)
hsa04658 Th1 and Th2 cell differentiation - Homo sapiens (human) (2)
hsa04664 Fc epsilon RI signaling pathway - Homo sapiens (human) (2)
hsa04670 Leukocyte transendothelial migration - Homo sapiens (human) (2)
hsa04975 Fat digestion and absorption - Homo sapiens (human) (2)
hsa05142 Chagas disease (American trypanosomiasis) - Homo sapiens (human) (2)
hsa05152 Tuberculosis - Homo sapiens (human) (2)
hsa05014 Amyotrophic lateral sclerosis (ALS) - Homo sapiens (human) (2)
hsa04066 HIF-1 signaling pathway - Homo sapiens (human) (2)
hsa05210 Colorectal cancer - Homo sapiens (human) (2)
hsa04926 Relaxin signaling pathway - Homo sapiens (human) (2)
hsa05230 Central carbon metabolism in cancer - Homo sapiens (human) (2)
hsa04810 Regulation of actin cytoskeleton - Homo sapiens (human) (2)
hsa05218 Melanoma - Homo sapiens (human) (2)
hsa04612 Antigen processing and presentation - Homo sapiens (human) (2)
hsa04622 RIG-I-like receptor signaling pathway - Homo sapiens (human) (2)
hsa00380 Tryptophan metabolism - Homo sapiens (human) (1)
hsa05211 Renal cell carcinoma - Homo sapiens (human) (1)
hsa00500 Starch and sucrose metabolism - Homo sapiens (human) (1)
hsa04115 p53 signaling pathway - Homo sapiens (human) (1)
hsa00350 Tyrosine metabolism - Homo sapiens (human) (1)
hsa04260 Cardiac muscle contraction - Homo sapiens (human) (1)
hsa04950 Maturity onset diabetes of the young - Homo sapiens (human) (1)
hsa04640 Hematopoietic cell lineage - Homo sapiens (human) (1)

hsa05146 Amoebiasis - Homo sapiens (human) (1)
hsa05016 Huntington's disease - Homo sapiens (human) (1)
hsa00220 Arginine biosynthesis - Homo sapiens (human) (1)
hsa00534 Glycosaminoglycan biosynthesis - heparan sulfate / heparin - Homo sapiens (human) (1)
hsa00970 Aminoacyl-tRNA biosynthesis - Homo sapiens (human) (1)
hsa04020 Calcium signaling pathway - Homo sapiens (human) (1)
hsa04964 Proximal tubule bicarbonate reclamation - Homo sapiens (human) (1)
hsa01200 Carbon metabolism - Homo sapiens (human) (1)
hsa04610 Complement and coagulation cascades - Homo sapiens (human) (1)
hsa00330 Arginine and proline metabolism - Homo sapiens (human) (1)
hsa05144 Malaria - Homo sapiens (human) (1)
hsa00650 Butanoate metabolism - Homo sapiens (human) (1)
hsa00020 Citrate cycle (TCA cycle) - Homo sapiens (human) (1)
hsa03040 Spliceosome - Homo sapiens (human) (1)
hsa05100 Bacterial invasion of epithelial cells - Homo sapiens (human) (1)
hsa05214 Glioma - Homo sapiens (human) (1)
hsa04340 Hedgehog signaling pathway - Homo sapiens (human) (1)
hsa00340 Histidine metabolism - Homo sapiens (human) (1)
hsa00290 Valine, leucine and isoleucine biosynthesis - Homo sapiens (human) (1)
hsa05020 Prion diseases - Homo sapiens (human) (1)
hsa05213 Endometrial cancer - Homo sapiens (human) (1)
hsa04930 Type II diabetes mellitus - Homo sapiens (human) (1)
hsa03420 Nucleotide excision repair - Homo sapiens (human) (1)
hsa00510 N-Glycan biosynthesis - Homo sapiens (human) (1)
hsa00620 Pyruvate metabolism - Homo sapiens (human) (1)
hsa04916 Melanogenesis - Homo sapiens (human) (1)
hsa00062 Fatty acid elongation - Homo sapiens (human) (1)
hsa05134 Legionellosis - Homo sapiens (human) (1)
hsa04530 Tight junction - Homo sapiens (human) (1)
hsa03022 Basal transcription factors - Homo sapiens (human) (1)
hsa05140 Leishmaniasis - Homo sapiens (human) (1)
hsa04720 Long-term potentiation - Homo sapiens (human) (1)
hsa04662 B cell receptor signaling pathway - Homo sapiens (human) (1)
hsa05130 Pathogenic Escherichia coli infection - Homo sapiens (human) (1)
hsa03410 Base excision repair - Homo sapiens (human) (1)
hsa00740 Riboflavin metabolism - Homo sapiens (human) (1)
hsa05223 Non-small cell lung cancer - Homo sapiens (human) (1)
hsa04512 ECM-receptor interaction - Homo sapiens (human) (1)
hsa04922 Glucagon signaling pathway - Homo sapiens (human) (1)

hsa00410 beta-Alanine metabolism - Homo sapiens (human) (1)
hsa05414 Dilated cardiomyopathy (DCM) - Homo sapiens (human) (1)
hsa05031 Amphetamine addiction - Homo sapiens (human) (1)
hsa00280 Valine, leucine and isoleucine degradation - Homo sapiens (human) (1)
hsa00240 Pyrimidine metabolism - Homo sapiens (human) (1)
hsa04921 Oxytocin signaling pathway - Homo sapiens (human) (1)
hsa04022 cGMP-PKG signaling pathway - Homo sapiens (human) (1)
hsa04970 Salivary secretion - Homo sapiens (human) (1)
hsa00310 Lysine degradation - Homo sapiens (human) (1)
hsa05231 Choline metabolism in cancer - Homo sapiens (human) (1)
hsa03015 mRNA surveillance pathway - Homo sapiens (human) (1)
hsa05216 Thyroid cancer - Homo sapiens (human) (1)
hsa05310 Asthma - Homo sapiens (human) (1)
hsa00601 Glycosphingolipid biosynthesis - lacto and neolacto series - Homo sapiens (human) (1)
hsa05416 Viral myocarditis - Homo sapiens (human) (1)
hsa04730 Long-term depression - Homo sapiens (human) (1)
hsa05217 Basal cell carcinoma - Homo sapiens (human) (1)
hsa00830 Retinol metabolism - Homo sapiens (human) (1)
hsa03013 RNA transport - Homo sapiens (human) (1)
hsa01212 Fatty acid metabolism - Homo sapiens (human) (1)
hsa04973 Carbohydrate digestion and absorption - Homo sapiens (human) (1)
hsa04146 Peroxisome - Homo sapiens (human) (1)
hsa05322 Systemic lupus erythematosus - Homo sapiens (human) (1)
hsa05410 Hypertrophic cardiomyopathy (HCM) - Homo sapiens (human) (1)
hsa04330 Notch signaling pathway - Homo sapiens (human) (1)
Androgen receptor
cAMP-specific 3,5-cyclic phosphodiesterase 4D
Cholinergic synapse
Endocrine and other factor-regulated calcium reabsorption
Estrogen-related receptor gamma,testis isoform
Fatty acid biosynthesis
GABAergic synapse
Glyoxylate and dicarboxylate metabolism
Neuroactive ligand-receptor interacion
Regulation of lipolysis in adipocytes
Renin secretion
Secondary bile acid biosynthesis
Serotonergic synapse
Thyroid hormone synthesis

Vasopressin-regulated water reabsorption
Alanine,aspartate and glutamate metabolism
Nitrogen metabolism
Pantothenate and CoA biosynthesis
Phenylalanine, tyrosine and tryptophan biosynthesis
Primary bile acid biosynthesis
Purine metabolism
Taurine and hypotaurine metabolism
glycerolipid metabolism
glycolysis or gluconeogenesis
phenylalanine, tyrosine and tryptophan bisoynthesis
Phenylalanine metabolism
Biosynthesis of unsaturated fatty
Sphingphosphplipid metabolism
phosphatidylcholine biosynthesis
Superpathway of methionine degradation
serotonin receptor signaliing
pruine nucleotides de novo biosynthesis II
tyrosine synthesis

Table S4 Herb-active compound-target protein-pathway network of YGP-treated KYDS rats

**Herb**	**Target proteins' number**	**Active compound**	**Target proteins in KEGG**	**Target proteins in YGP**	**Uniprot ID**	**Pathway**
**FZ (*Radix Aconiti Lateralis Preparata*)**	6	11,14-eicosadienoic acid	L-serine/L-threonine ammonia-lyase [EC:4.3.1.17 4.3.1.19]	L-serine dehydratase	P20132	hsa00260 Glycine, serine and threonine metabolism - Homo sapiens (human) (2)
			betaine-homocysteine S-methyltransferase [EC:2.1.1.5]	Betaine--homocysteine S-methyltransferase 1	Q93088	
			alcohol dehydrogenase 1/7 [EC:1.1.1.1]	Alcohol dehydrogenase 1C	P00326	hsa00010 Glycolysis / Gluconeogenesis - Homo sapiens (human) (1)
			L-serine/L-threonine ammonia-lyase [EC:4.3.1.17 4.3.1.19]	L-serine dehydratase	P20132	hsa00290 Valine, leucine and isoleucine biosynthesis - Homo sapiens (human) (1)

		Karanjin	cystathionine beta-synthase [EC:4.2.1.22]	Cystathionine beta-synthase	P35520	hsa00260 Glycine, serine and threonine metabolism - Homo sapiens (human) (2)
			betaine-homocysteine S-methyltransferase [EC:2.1.1.5]	Betaine--homocysteine S-methyltransferase 1	Q93088	
SY (***Rhizoma Dioscoreae***)	9	24-Methylcholest-5-enyl-3belta-O-glucopyranoside_qt	L-serine/L-threonine ammonia-lyase [EC:4.3.1.17 4.3.1.19]	L-serine dehydratase	P20132	hsa00260 Glycine, serine and threonine metabolism - Homo sapiens (human) (2)
			betaine-homocysteine S-methyltransferase [EC:2.1.1.5]	Betaine--homocysteine S-methyltransferase 1	Q93088	
			L-serine/L-threonine ammonia-lyase [EC:4.3.1.17 4.3.1.19]	L-serine dehydratase	P20132	hsa00290 Valine, leucine and isoleucine biosynthesis - Homo sapiens (human) (1)
			alcohol dehydrogenase 1/7 [EC:1.1.1.1]	Alcohol dehydrogenase 1C	P00326	hsa00010 Glycolysis / Gluconeogenesis - Homo sapiens (human) (1)
		Denudatin B	phosphoenolpyruvate carboxykinase (GTP) [EC:4.1.1.32]	Phosphoenolpyruvate carboxykinase, cytosolic [GTP]	ADHX_HUMAN	hsa00010 Glycolysis / Gluconeogenesis - Homo sapiens (human) (1)
			phosphoenolpyruvate carboxykinase (GTP) [EC:4.1.1.32]	Phosphoenolpyruvate carboxykinase, cytosolic [GTP]	P35558	hsa00620 Pyruvate metabolism - Homo sapiens (human) (1)
		Kadsurenone	phosphoenolpyruvate carboxykinase (GTP) [EC:4.1.1.32]	Phosphoenolpyruvate carboxykinase, cytosolic [GTP]	P35558	hsa00620 Pyruvate metabolism - Homo sapiens (human) (1)
			betaine-homocysteine S-methyltransferase [EC:2.1.1.5]	Betaine--homocysteine S-methyltransferase 1	Q93088	hsa00260 Glycine, serine and threonine metabolism - Homo sapiens (human) (1)
			phosphoenolpyruvate carboxykinase (GTP) [EC:4.1.1.32]	Phosphoenolpyruvate carboxykinase, cytosolic [GTP]	ADHX_HUMAN	hsa00010 Glycolysis / Gluconeogenesis - Homo sapiens (human) (1)
**SZY (*Macrocarpium Officinale*)**	6	Ethyl oleate (NF)	betaine-homocysteine S-methyltransferase [EC:2.1.1.5]	Betaine--homocysteine S-methyltransferase 1	Q93088	hsa00260 Glycine, serine and threonine metabolism - Homo sapiens (human) (1)
			phosphoenolpyruvate carboxykinase (GTP)	Phosphoenolpyruvate carboxykinase, cytosolic	P35558	hsa00620 Pyruvate metabolism - Homo

			[EC:4.1.1.32]	[GTP]		sapiens (human) (1)
			phosphoenolpyruvate carboxykinase (GTP) [EC:4.1.1.32]	Phosphoenolpyruvate carboxykinase, cytosolic [GTP]	ADHX_HUMAN	hsa00010 Glycolysis / Gluconeogenesis - Homo sapiens (human) (1)
		Mandenol	phosphoenolpyruvate carboxykinase (GTP) [EC:4.1.1.32]	Phosphoenolpyruvate carboxykinase, cytosolic [GTP]	P35558	hsa00620 Pyruvate metabolism - Homo sapiens (human) (1)
			phosphoenolpyruvate carboxykinase (GTP) [EC:4.1.1.32]	Phosphoenolpyruvate carboxykinase, cytosolic [GTP]	ADHX_HUMAN	hsa00010 Glycolysis / Gluconeogenesis - Homo sapiens (human) (1)
		Tetrahydroalstonine	betaine-homocysteine S-methyltransferase [EC:2.1.1.5]	Betaine--homocysteine S-methyltransferase 1	Q93088	hsa00260 Glycine, serine and threonine metabolism - Homo sapiens (human) (1)
**GQ (*Lycium Barbarum*)**	15	(E,E)-1-ethyl octadeca-3,13-dienoate	alcohol dehydrogenase 1/7 [EC:1.1.1.1]	Alcohol dehydrogenase class-3	ADHX_HUMAN	hsa00010 Glycolysis / Gluconeogenesis - Homo sapiens (human) (1)
			betaine-homocysteine S-methyltransferase [EC:2.1.1.5]	Betaine--homocysteine S-methyltransferase 1	Q93088	hsa00260 Glycine, serine and threonine metabolism - Homo sapiens (human) (1)
		Atropine	betaine-homocysteine S-methyltransferase [EC:2.1.1.5]	Betaine--homocysteine S-methyltransferase 1	Q93088	hsa00260 Glycine, serine and threonine metabolism - Homo sapiens (human) (1)
			phosphoenolpyruvate carboxykinase (GTP) [EC:4.1.1.32]	Phosphoenolpyruvate carboxykinase, cytosolic [GTP]	P35558	hsa00620 Pyruvate metabolism - Homo sapiens (human) (1)
			phosphoenolpyruvate carboxykinase (GTP) [EC:4.1.1.32]	Phosphoenolpyruvate carboxykinase, cytosolic [GTP]	P00326	hsa00010 Glycolysis / Gluconeogenesis - Homo sapiens (human) (1)
			spermidine synthase [EC:2.5.1.16]	Spermidine synthase	P19623	hsa00330 Arginine and proline metabolism - Homo sapiens (human) (1)
		Mandenol	phosphoenolpyruvate carboxykinase (GTP) [EC:4.1.1.32]	Phosphoenolpyruvate carboxykinase, cytosolic [GTP]	P35558	hsa00620 Pyruvate metabolism - Homo sapiens (human) (1)

			phosphoenolpyruvate carboxykinase (GTP) [EC:4.1.1.32]	Phosphoenolpyruvate carboxykinase, cytosolic [GTP]	ADHX_HUMAN	hsa00010 Glycolysis / Gluconeogenesis - Homo sapiens (human) (1)
		4alpha,24-dimethylcholesta-7,24-dienol	alcohol dehydrogenase 1/7 [EC:1.1.1.1]	Alcohol dehydrogenase 1C	P00326	hsa00010 Glycolysis / Gluconeogenesis - Homo sapiens (human) (1)
		4alpha-methyl-24-ethylcholesta-7,24-dienol	alcohol dehydrogenase 1/7 [EC:1.1.1.1]	Alcohol dehydrogenase 1C	P00326	hsa00010 Glycolysis / Gluconeogenesis - Homo sapiens (human) (1)
		24-methylidenelophenol	alcohol dehydrogenase 1/7 [EC:1.1.1.1]	Alcohol dehydrogenase class-3	ADHX_HUMAN	hsa00010 Glycolysis / Gluconeogenesis - Homo sapiens (human) (1)
		Obtusifoliol	alcohol dehydrogenase 1/7 [EC:1.1.1.1]	Alcohol dehydrogenase 1C	P00326	hsa00010 Glycolysis / Gluconeogenesis - Homo sapiens (human) (1)
		Lophenol	alcohol dehydrogenase 1/7 [EC:1.1.1.1]	Alcohol dehydrogenase 1C	P00326	hsa00010 Glycolysis / Gluconeogenesis - Homo sapiens (human) (1)
		Sitosterol alpha1	alcohol dehydrogenase 1/7 [EC:1.1.1.1]	Alcohol dehydrogenase class-3	ADHX_HUMAN	hsa00010 Glycolysis / Gluconeogenesis - Homo sapiens (human) (1)
		betaine	cystathionine beta-synthase [EC:4.2.1.22]	Cystathionine beta-synthase	P35520	hsa00260 Glycine, serine and threonine metabolism - Homo sapiens (human) (1)
**RG (*Cinnamomi cortex*)**	1	Styrone	tyrosyl-tRNA synthetase [EC:6.1.1.1]	Tyrosyl-tRNA synthetase, cytoplasmic	P54577	hsa00970 Aminoacyl-tRNA biosynthesis - Homo sapiens (human) (1)
_qt: the residue of saccharide after desaccharify (i.e., aglycone)						
